# *In vivo* fitness of *sul* gene-dependent sulfonamide-resistant *Escherichia coli* in the mammalian gut

**DOI:** 10.1128/msystems.00836-24

**Published:** 2024-08-14

**Authors:** Han Jiang, Yuzhi Dong, Xue Jiao, Biao Tang, Tao Feng, Ping Li, Jiehong Fang

**Affiliations:** 1 Key Laboratory of Specialty Agri-products Quality and Hazard Controlling Technology of Zhejiang Province, College of Life Sciences, China Jiliang University, Hangzhou, Zhejiang, China; 2 School of Life Science, Hangzhou Institute for Advanced Study, University of Chinese Academy of Sciences, Hangzhou, Zhejiang, China; 3 Key Laboratory for Food Microbial Technology of Zhejiang Province, Zhejiang Gongshang University, Hangzhou, Zhejiang, China; University of California Irvine, Irvine, California, USA

**Keywords:** sulfonamide resistance gene, fitness cost, *in vivo*, quantitative proteomic method, *Escherichia coli*

## Abstract

The widespread sulfonamide resistance genes *sul1*, *sul2*, and *sul3* in food and gut bacteria have attracted considerable attention. In this study, we assessed the *in vivo* fitness of *sul* gene-dependent sulfonamide-resistant *Escherichia coli*, using a murine model. High fitness costs were incurred for *sul1* and *sul3* gene-dependent *E. coli* strains *in vivo*. A fitness advantage was found in three of the eight mice after intragastric administration of *sul2* gene-dependent *E. coli* strains. We isolated three compensatory mutant strains (CMSs) independently from three mice that outcompeted the parent strain P2 *in vivo*. Whole-genome sequencing revealed seven identical single nucleotide polymorphism (SNP) mutations in the three CMSs compared with strain P2, an additional SNP mutation in strain S2-2, and two additional SNP mutations in strain S2-3. Furthermore, tandem mass tag-based quantitative proteomic analysis revealed abundant differentially expressed proteins (DEPs) in the CMSs compared with P2. Of these, seven key fitness-related DEPs distributed in two-component systems, galactose and tryptophan metabolism pathways, were verified using parallel reaction monitoring analysis. The DEPs in the CMSs influenced bacterial motility, environmental stress tolerance, colonization ability, carbohydrate utilization, cell morphology maintenance, and chemotaxis to restore fitness costs and adapt to the mammalian gut environment.

**IMPORTANCE:**

Sulfonamides are traditional synthetic antimicrobial agents used in clinical and veterinary medical settings. Their long-term excessive overuse has resulted in widespread microbial resistance, limiting their application for medical interventions. Resistance to sulfonamides is primarily conferred by the alternative genes *sul1*, *sul2*, and *sul3* encoding dihydropteroate synthase in bacteria. Studying the potential fitness cost of these *sul* genes is crucial for understanding the evolution and transmission of sulfonamide-resistant bacteria. *In vitro* studies have been conducted on the fitness cost of *sul* genes in bacteria. In this study, we provide critical insights into bacterial adaptation and transmission using an *in vivo* approach.

## INTRODUCTION

Sulfonamides are important synthetic antimicrobial agents that are used extensively in clinical and veterinary medicine in developing countries; they have a broad antibacterial spectrum, exhibit stable properties, and are cost-effective. However, their extended and inappropriate usage has led to widespread sulfonamide resistance in bacteria found in food animals and retail meat intended for human consumption. Gradually, this has reduced their efficacy in medical interventions for humans (1–3). Bacteria develop resistance to sulfonamides by two main mechanisms, which are the mutation of the *folP* gene that encodes dihydropteroate synthase (DHPS) and the acquisition of an alternative DHPS gene, named *sul* genes, whose products have a lower affinity for sulfonamides (4). The most prevalent *sul* genes identified on both chromosomes and plasmids are *sul1*, *sul2*, and *sul3* (5). However, plasmids harboring *sul* genes are more readily disseminated among bacterial populations through conjugation or transformation, thereby propagating *sul* genes globally (2, 5–8). The *sul1*, *sul2*, and *sul3* genes are highly conserved, but their base sequences exhibit approximately 50% homology with each other (9, 10).

Antibiotic resistance frequently correlates with reduced competitiveness when compared to antibiotic-sensitive strains in the absence of antibiotics. Such fitness cost depends on the specific antimicrobial resistance gene (ARG) mutation, the genetic background of the strain, and whether it can be ameliorated by compensatory mutations (11–13). The factors driving antibiotic resistance evolution *in vitro* have been characterized extensively; however, few studies have explored its evolution *in vivo*, which significantly restricts the in-depth understanding of its prevalence and transmission (13). An early seminal study by Gagneux et al., reveals that laboratory mutants of *Mycobacterium tuberculosis* with rifampin resistance typically suffer from decreased competitive fitness due to a unique resistance mutation and the genetic profile of the strain. In contrast, prolonged patient treatment can result in multidrug-resistant strains with no fitness defect (11). Another *in vivo* study showed that compensatory mutations selected *in vivo* effectively mitigate the fitness cost associated with mosaic *penA* alleles, which confer ceftriaxone resistance in *Neisseria gonorrhoeae*. Such mutations primarily enhance metabolism to address the fitness deficit (14). In addition, the presence of MgrB-dependent colistin resistance in *Klebsiella pneumoniae* has been linked to a biological cost in gut colonization. Nevertheless, this drawback is offset by enhanced viability outside the host, facilitating increased host-to-host transmission (15). In the case of *sul* genes discussed in the present study, the *in vitro* fitness cost of *sul* genes in *Escherichia coli* has already been studied, and *sul3* gene-carrying *E. coli* reportedly has a specific fitness cost in *in vitro* competition tests. However, this cost was recovered under sub-inhibitory concentrations of sulfisoxazole (10). Therefore, elucidating the *in vivo* fitness of *sul* gene-dependent sulfonamide-resistant *E. coli* can provide insights into bacterial adaptation and transmission.

**Fig 1:**
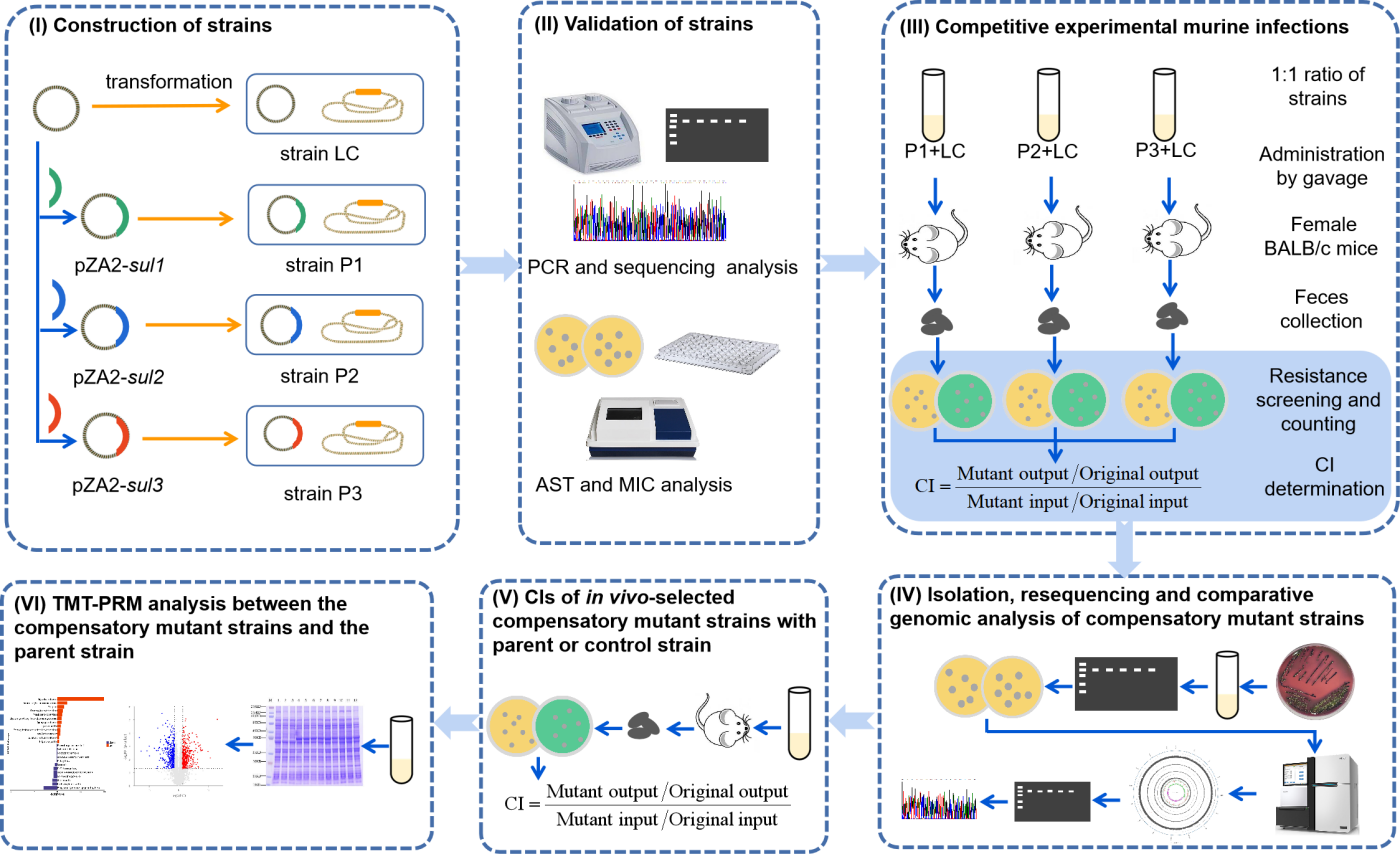
Overview of the experimental design.

In this study, we assessed the potential fitness costs of *sul1*, *sul2*, and *sul3* gene-dependent sulfonamide resistance in *E. coli in vivo*. The experimental design is illustrated in Fig. 1. Briefly, *sul1*, *sul2*, and *sul3* genes are inserted into a pZA2 constitutive expression vector and transformed into *E. coli* MG1656 *araB::cat*. A prior investigation revealed that the plasmid pZA2 with a 6.2 kbp backbone minimally slowed *E. coli* MG1656 growth by 0.8%, and utilizing *E. coli* MG1656 carrying the plasmid pZA2 emerged as one of the optimal selections for probing the fitness cost of functional elements within *E. coli* (16). Moreover, previous studies have demonstrated that *araB* inactivation is fitness neutral in a murine model, and *E. coli* growth is not significantly influenced by the chloramphenicol-resistance gene marker (*cat*) (17, 18). Subsequently, relative fitness was measured using an *in vivo* test of female mice bacterial competition. Furthermore, three compensatory mutant strains (CMSs) carrying the *sul2* gene were isolated from infected mice that exhibited increased fitness, and their genomes were sequenced. Finally, fitness changes in the CMSs at the protein expression level were explored using a tandem mass tag (TMT)-based quantitative proteomic method and parallel reaction monitoring (PRM) analysis.

## RESULTS

### Characterization of sulfonamide-resistant test strains and sulfonamide-sensitive control strain

Before investigating the fitness cost, we confirmed the presence of *sul1, sul2,* or *sul3* in *E. coli* MG1656 *araB::cat* using polymerase chain reaction (PCR) (Fig. S1a). Furthermore, whole-genome sequencing (WGS) showed that the pZA2 and pZA2-*suls* recombinant plasmids had gene sequences consistent with the theoretical sequences. Antimicrobial susceptibility test (AST) results showed that sulfonamide-sensitive control strain (hereafter referred to as LC) was sensitive to ampicillin (antibiotic-sensitive control) and sulfisoxazole (representing any currently available sulfonamide preparation) but resistant to kanamycin (pZA2 plasmid carrying kanamycin resistance gene *aph(3′)-Ia*) and chloramphenicol (strain MG1656 carrying chloramphenicol-resistance gene *catA1*) (19). In addition, sulfonamide-resistant test strains (hereafter referred to as P1, P2, and P3) were sensitive to ampicillin and resistant to kanamycin, chloramphenicol, and sulfisoxazole (Fig. S1b). The minimum inhibitory concentration (MIC) for sulfisoxazole in strain LC was 0.25 mg/mL, and 4.0 mg/mL for strains P1, P2, and P3, each. According to the Clinical and Laboratory Standards Institute (CLSI) guideline, a sulfonamide-resistant strain is determined at MIC > 512 µg/mL (Fig. S1c).

### *In vivo* competition assays and CMS selection

**Fig 2:**
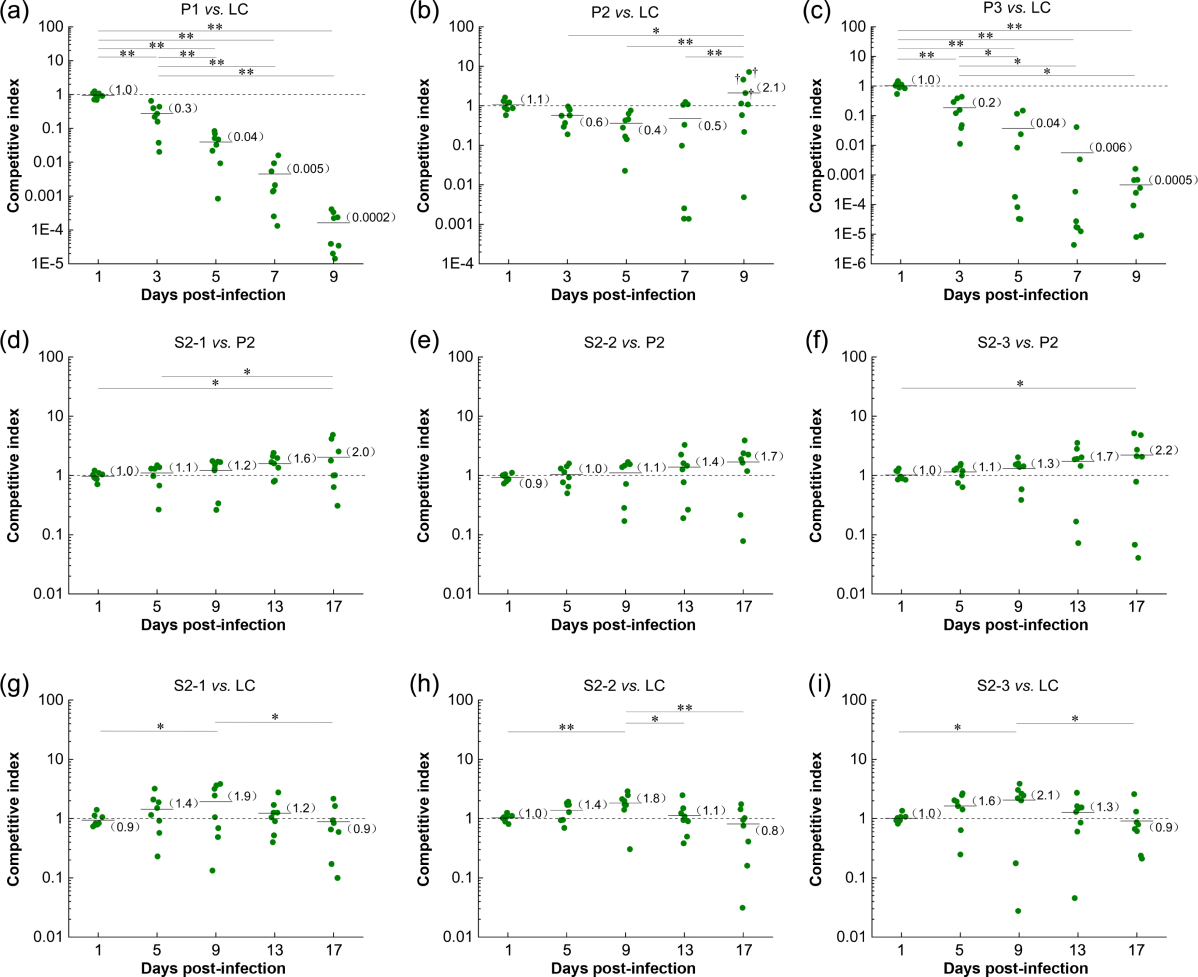
*In vivo* competition experiments. (a–c) *In vivo* competition indices (CIs) for the sulfonamide-resistant test strains P1, P2, and P3 vs sulfonamide-sensitive control strain LC. (d–f) *In vivo* CIs for CMSs S2-1, S2-2, and S2-3 vs parent strain P2. (g–i) *In vivo* CIs for CMSs S2-1, S2-2, and S2-3 vs strain LC; *P < 0.05, **P < 0.01. Each dot denotes the CI of an individual mouse, with eight mice in each group. The short solid line represents the arithmetic mean (also shown in parentheses), and the horizontal dashed line represents CI of 1. The detection limit considers four CFUs when calculating the CI since the strains do not grow on the LB agar plate. † indicates mice where possible CMSs were observed *in vivo*. The analysis was conducted on the isolates collected from the mice feces. The data were obtained from three independent experiments.

*In vivo* competition assays were performed using strains P1, P2, or P3 and LC to determine whether the *sul* genes conferring sulfonamide resistance were associated with fitness costs in *E. coli* (Fig. 2a through c). The colony forming unit (CFU) data for each strain in individual mice, from which the competitive index (CI) is derived, is presented in Table S1. The mean CI was <1 on day 3 and decreased sharply daily. We observed significantly lower mean CIs (*P* < 0.01) for strains P1 and P3 in competition with strain LC over the entire 9 days of infection, suggesting higher fitness costs for *sul1* and *sul3* gene-dependent sulfonamide resistance in *E. coli in vivo* (Fig. 2a and c). The mean CI of strain P2 in competition with strain LC decreased in the first 5 days; however, it recovered gradually from day 7. In addition, CI > 1, indicating fitness advantages, occurred on day 9 in three of the eight mice after intragastric administration, showing significant high fitness recovery compared with day 7 (*P* < 0.01; Fig. 2b; CIs showing this shift are indicated by †). The three mice are labeled P2-V, P2-VI, and P2-VII, and the detailed CFU data are presented in Table S1 in bold. We suspected that during infection in these three mice, strain P2 spontaneously acquired compensatory mutations that improved fitness. We prepared frozen stocks of well-isolated single colonies from kanamycin-, chloramphenicol-, and sulfisoxazole-containing Luria–Bertani (LB) agar plates and subjected them to further phenotypic and genotypic analyses. One representative suspected CMS from mice P2-V, mice P2-VI, and mice P2-VII, respectively, were selected for subsequent investigation (hereafter referred to as CMSs S2-1, S2-2, and S2-3).

### Identification of CMSs and competitive coinfection of CMSs with strain P2 or strain LC

First, based on the sulfisoxazole susceptibility test and MIC results, CMSs S2-1, S2-2, and S2-3 showed no changes compared to strain P2 (Fig. S2a and b). In addition, PCR identification and WGS showed that they were all *E. coli* strains containing *sul2, catA1,* and *aph(3′)-Ia* resistance genes (Fig. S2c and d). The growth curves in LB broth showed that the three CMSs grew significantly faster than strain P2 (*P* < 0.05) but at a rate similar to strain LC (Fig. S2e).

Second, after detecting single nucleotide polymorphism (SNP) and annotation, we observed seven identical SNP mutations in the three CMSs compared with strain P2, an additional SNP mutation in strain S2-2, and two additional SNP mutations in strain S2-3 (detailed information in Table 1). Of these, a potential fitness response Cytosine to Adenine mutation was found at position 3,822,476 of the genome, converting the 26th alanine residue in the original translation of the *spoT* gene to glutamic acid (A26E).

**TABLE 1:** SNP mutations in the three CMSs relative to the parent strain P2

Bacterial strain	Gene name	Product or function	Mutation	Gene position (in nucleotides)	Genome position
S2-1, S2-2, S2-3	*lacI*	DNA-binding transcriptional repressor LacI	L114R, A→C	341	367,170
	*folD*	Bifunctional methylenetetrahydrofolate dehydrogenase/methenyltetrahydrofolate cyclohydrolase	S47L, G→A	140	557,602
	*ybfP*	Lipoprotein YbfP	G80E, G→A	239	715,650
	*insH6*	IS5 family transposase and trans-activator	K274R, T→C	821	2,066,465
	*yqeF*	Putative acyltransferase	A375V, G→A	1,124	2,984,469
	*spoT*	Bifunctional (p)ppGpp synthase/hydrolase SpoT	A26E, C→A	77	3,822,476
	*glpK*	Glycerol kinase	G411S, C→T	1,231	4,115,992
S2-2	*glk*	Glucokinase	T316T, G→A	948	2,508,479
S2-3	*ybaM*	DUF2496 domain-containing protein YbaM	A13V, G→A	38	490,234
	*ptrB*	Oligopeptidase B	S469N, C→T	1,406	1,927,434

Third, according to the mean CIs, the three CMSs showed an approximately twofold increase in fitness on the 17th day of infection relative to strain P2 (Fig. 2d through f). These three CMSs outcompeted strain LC, with an approximately twofold increase in fitness on the 9th day of infection, followed by a decrease on the 13th day and a slight fitness cost relative to strain LC on the 17th day (Fig. 2g through i).

### Comparative proteomics of total proteins between the CMSs and strain P2

**Fig 3:**
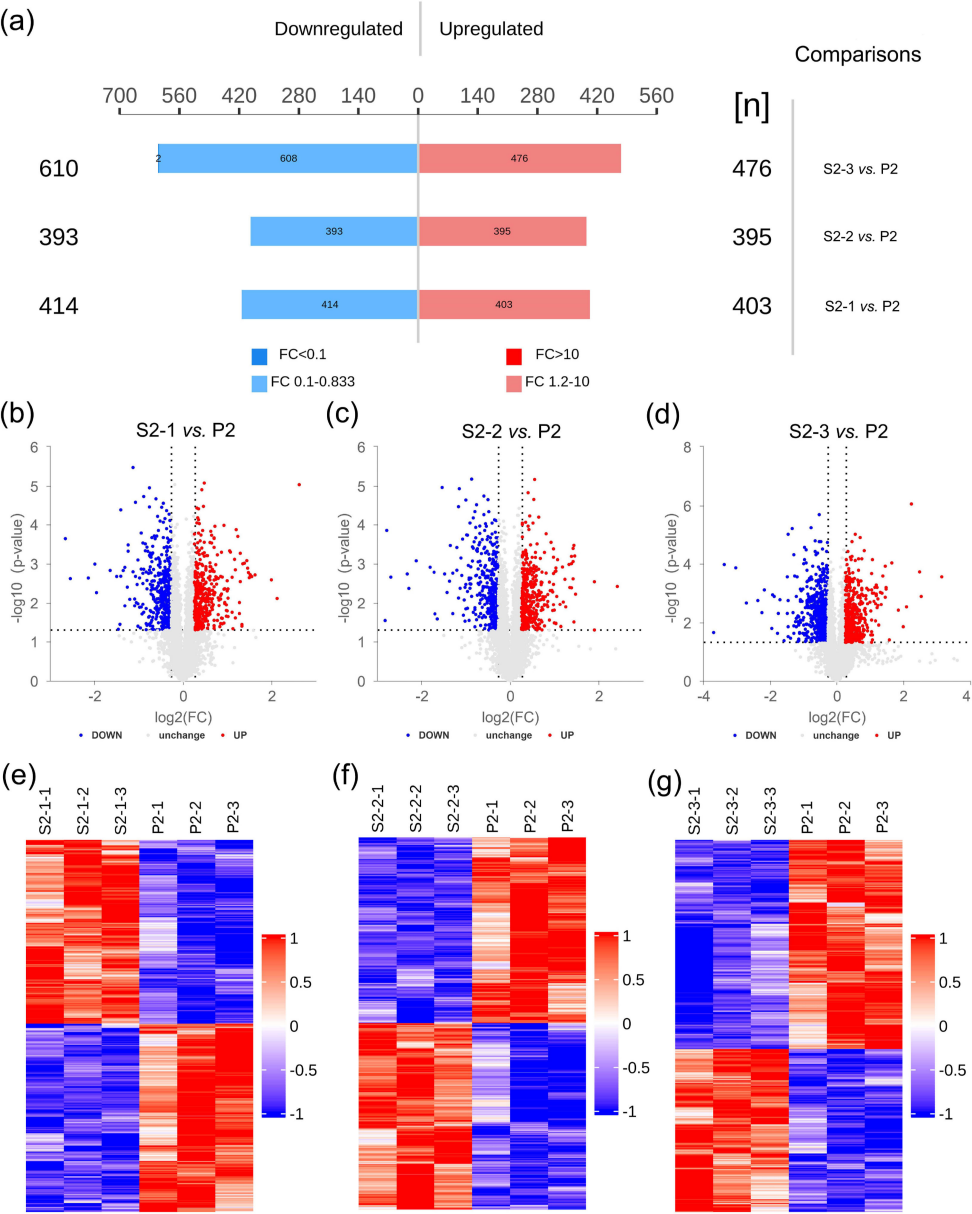
Quantitative proteomics analysis of the CMSs S2-1, S2-2, and S2-3 and parent strain P2. (**a**) A number of proteins undergo obvious upregulation and downregulation. (b–d) Volcano plot analysis of DEPs. (**b**) S2-1 vs P2; (**c**) S2-2 vs P2; (**d**) S2-3 vs P2. The *x*-axis represents expression changes (logarithmic transformation with a base of 2), and the *y*-axis denotes the statistical significance between the two groups (logarithmic transformation with a base of 10). Red dots: upregulated DEPs, blue dots: downregulated DEPs, gray dots: no significant differences in expression. (e–g) Heatmaps of DEP hierarchical clustering analysis between CMSs S2-1, S2-2, and S2-3 with strain P2. The experiments were repeated thrice.

Globally, the TMT-labeled quantitative proteomics analysis identified 4,726 quantifiable proteins from 31,603 peptides. Of these, 817 were differentially expressed between strains S2-1 and P2 (403 upregulated and 414 downregulated). Similarly, 788 (395 upregulated and 393 downregulated) and 1,086 (476 upregulated and 610 downregulated) proteins were altered in strains S2-2 and S2-3, respectively, compared with strain P2 (Fig. 3a; Table S2). These are also shown in the volcano plots [Fig. 3b and d; red, blue, and gray points indicate upregulated, downregulated, and non-significant differentially expressed proteins (DEPs), respectively] and heatmaps (Fig. 3e through g), which indicated an overview of all significant DEPs between CMSs and strain P2.

### Functional annotations of DEPs

**Fig 4:**
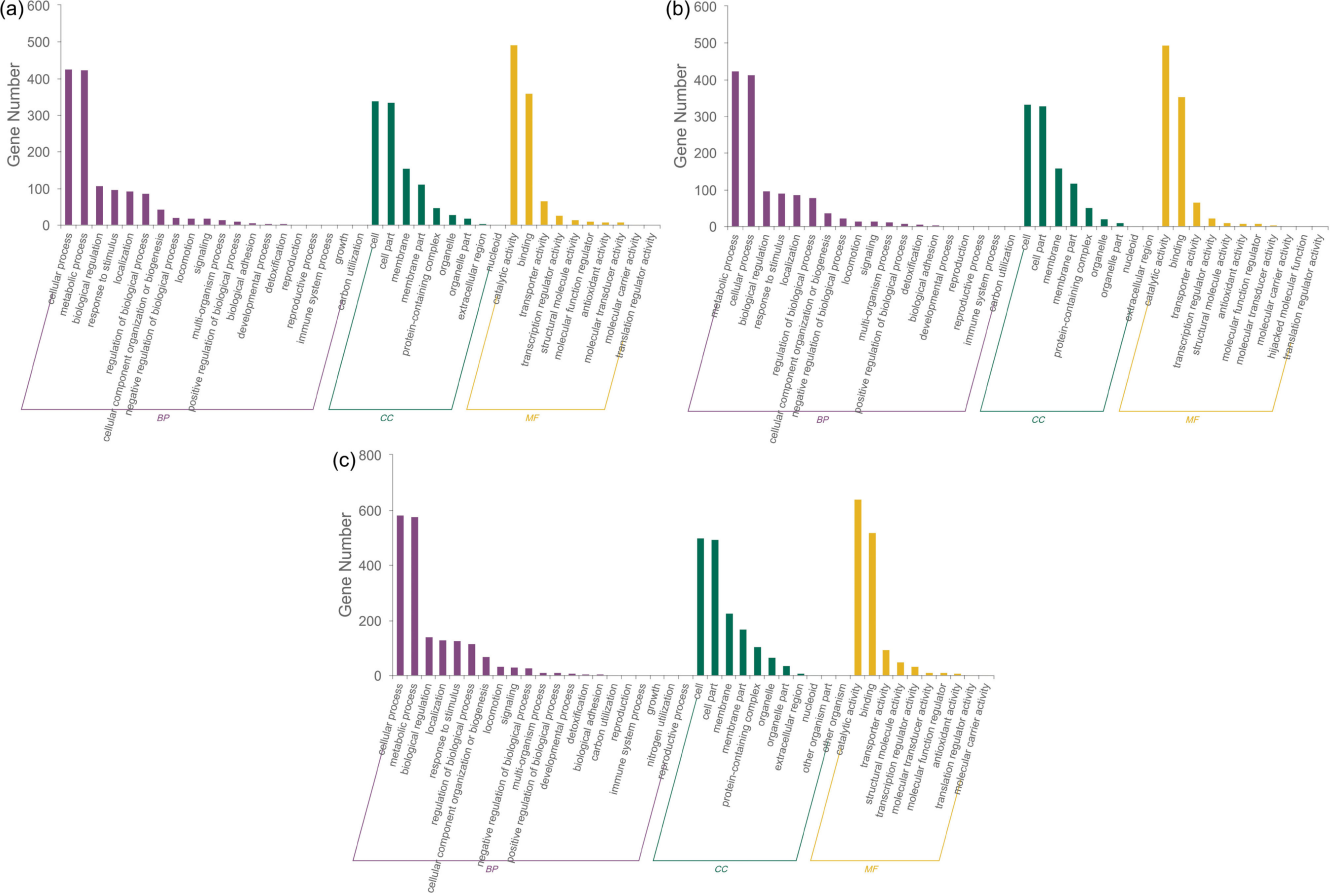
GO enrichment analysis of DEPs. (**a**) CMS S2-1 vs parent strain P2. (**b**) S2-2 vs P2. (**c**) S2-3 vs P2. The *x*-axis indicates DEPs belonging to the BP, CC, and MF terms, and the *y*-axis indicates the number of genes under each functional category.

We analyzed the identified DEPs in terms of biological processes (BP), cellular components (CC), and molecular functions (MF). Subsequent Gene Ontology (GO) functional enrichment analysis confirmed the functional categories of DEP enrichment using Fisher’s exact test (*P* < 0.05). As shown in Fig. 4a through c, the primary DEP-enriched categories in the 20 BPs were cellular and metabolic processes; of the nine CCs, the main terms were cell and cell parts; of the 10 MFs, the most enriched terms were catalytic and binding functions. The DEP information for the top 20 enriched GO terms in the BP, CC, and MF of the three CMSs is summarized in Tables S3 to S5, respectively.

**Fig 5:**
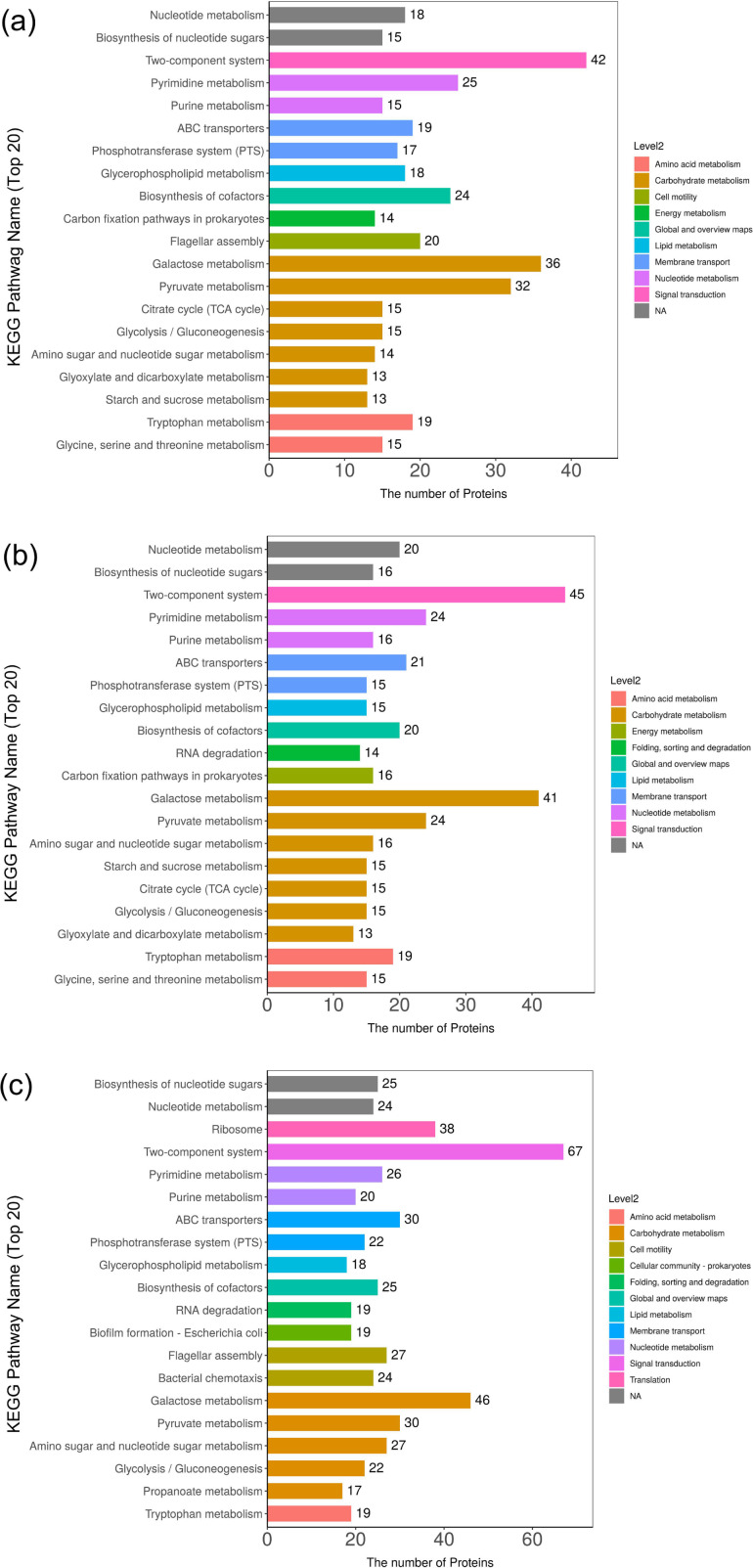
Top 20 KEGG pathways with most DEPs. (**a**) CMS S2-1 vs parent strain P2. (**b**) S2-2 vs P2. (**c**) S2-3 vs P2. The *x*-axis indicates the number of DEPs, and the *y*-axis indicates the name of the pathway involving DEPs.

In addition, DEPs were subjected to pathway enrichment analysis using the Kyoto Encyclopedia of Genes and Genomes (KEGG) database, together with the integration of their metabolic pathways. The DEPs between strains S2-1 and P2, S2-2 and P2, and S2-3 and P2 were related to 98, 97, and 118 KEGG pathways, respectively. Figures S3a through c demonstrate the top 20 pathways with a *P*-value < 0.05. Larger, rich factors report greater enrichment, and larger bubbles report more DEPs. In addition, a redder bubble indicates a smaller *P*-value; in this case, the corresponding metabolic pathway enrichment exhibits a higher significance. Thus, the DEPs involved in galactose and tryptophan metabolism merit further research. In addition, for CMSs S2-1, S2-2, and S2-3, a total of 42, 45, and 67 DEPs were involved in a two-component system, and 36, 41, and 46 DEPs with galactose metabolism, respectively (Fig. 5a through c). These two metabolic pathways had the highest number of DEPs among the three CMSs (supporting information: Table S6). Thus, critical proteins related to two components, galactose and tryptophan metabolisms merit further attention.

### PRM quantification of candidate proteins

Based on the TMT results, eight essential proteins were selected for further PRM analysis (Table 2): four two-component system-related proteins (sensor histidine protein kinase/phosphatase, PhoQ, A0A376MVG2; Rcs stress response system protein, RcsF, A0A377E1X8; response regulator transcription factor, RcsB, A0A029HTA9; RNA polymerase sigma factor, FliA, A0A7T2N4B4), one protein related to galactose metabolism (uridine diphosphate-glucose pyrophosphorylase 2, UGP2, A0A3Y1V1S8), two tryptophan metabolism-related proteins (tryptophanase, TnaA, A0A8B4PN44; tryptophan permease, TnaB, A0A376J8Y2), and one protein related to sulfonamide-resistant DHPS 2 (Sul2, A0A3G4RTB5). Overall, PRM and TMT yielded almost consistent results (Table 2), verifying the reliability of the TMT quantitative proteomics.

**TABLE 2:** Quantification result comparison between TMT and PRM for the eight candidate proteins

Protein name	Description	exp/con^*b*^ (TMT)^*c*^			Average multiple^*b*^	exp/con (PRM)^*c*^			Average multiple^*c*^
		S2-1/P2	S2-2/P2	S2-3/P2		S2-1/P2	S2-2/P2	S2-3/P2	
A0A376MVG2	Sensor histidine protein kinase/phosphatase (PhoQ)	1.4494	1.4691	1.4027	1.4404	1.5618	1.5143	1.6204	1.5655
A0A377E1X8	Rcs stress response system protein (RcsF)	1.5265	1.4710	1.4697	1.4891	1.5063	1.5034	1.4034	1.4710
A0A029HTA9	Response regulator transcription factor (RcsB)	0.8145^*a*^	0.7752	0.7232	0.7710	0.8173^*a*^	0.5251	0.4489	0.5235
A0A7T2N4B4	The RNA polymerase sigma factor (Fli A)	1.3047	1.3832	1.2704	1.3194	1.5835	1.7565	1.5256	1.6218
A0A3Y1V1S8	UDP-glucose pyrophosphorylase 2 (UGP2)	2.2617	2.0372	2.2697	2.1895	2.0345	2.2096	1.8676	2.0372
A0A8B4PN44	Tryptophanase (TnaA)	6.1354	5.3432	5.8626	5.7804	4.9608	3.8826	6.5890	5.1441
A0A376J8Y2	Tryptophan permease (TnaB)	2.0189	1.9296	2.0205	1.9897	2.0189	1.9296	2.0204	1.9896
A0A3G4RTB5	Sulfonamide-resistant dihydropteroate synthase 2 (Sul2)	0.9004^*a*^	0.8465^*a*^	0.8281^*a*^	0.8583^*a*^	1.0000^*a*^	1.0608^*a*^	1.0367^*a*^	1.0325^*a*^

^*a*^ TMT or PRM results are not significantly up- or downregulated (0.8–1.2-fold change).

^*b*^ exp/con means experimental group/control group.

^*c*^ Average of three biological and technical replicates, each.

### Analysis of DEP variation in response to fitness recovery

The two-component system is the largest family of multistep signal transduction pathways that regulate cellular processes and is commonly utilized by bacteria for sensing and adapting to changing environments (20). In this study, we considered four essential proteins in this two-component system. In the PhoQ/PhoP two-component system, PhoQ expression was significantly upregulated (*P* < 0.05). In the Rsc two-component system, RcsF expression was significantly upregulated, whereas RcsB was significantly downregulated (except in strain S2-1; *P* < 0.05). RNA polymerase sigma factor FliA expression was also significantly upregulated (*P* < 0.05); the QseB/QseC two-component system regulates this protein.

Galactose metabolism involves the conversion of galactose to glucose, lactose, and other sugar intermediates used in metabolic processes. In the present study, UGP2 expression was significantly upregulated (*P* < 0.05). Galactose can be converted to Uridine 5′ diphospho-glucose (UDPG) by UGP2 catalysis and plays a vital role in carbohydrate metabolism, including biosynthesis (21). Hence, increased UGP2 expression may influence carbohydrate utilization by regulating galactose metabolism for host bacteria to adapt to their environment.

The most significantly upregulated (>5-fold) protein was TnaA (*P* < 0.05), which belongs to the tryptophan metabolic pathway. Furthermore, the expression of the corresponding protein, TnaB, was significantly upregulated (*P* < 0.05). During tryptophan metabolism, TnaA and TnaB contribute to indole synthesis. By accumulating higher indole concentrations, bacteria can regulate their chemotaxis and adaptability, increasing the colonization level and adaptability of the host in the intestine for improved bacterial survival (22, 23).

In addition, Sul2 expression was not significantly upregulated or downregulated in TMT or PRM analysis.

## DISCUSSION

In this study, we focused on the *in vivo* fitness of *sul* gene-dependent sulfonamide-resistant *E. coli* in the mammalian gut. Target gene- or element-carrying plasmid construction in genetically engineered bacteria and CI calculations between two competing strains are frequently used to evaluate the fitness cost of bacteria (10, 14, 24). Moreover, the plasmid pZA2 and the *E. coli* strain MG1656 used in our study have been used in many published fitness cost studies (16, 24). In our study, only *sul2* gene-dependent strains obtained an average fitness advantage on day 9 compared with the sulfonamide-sensitive control strain. In a previous study using LB medium, *sul1* and *sul2* gene-dependent sulfonamide resistance in *E. coli* (*E. coli* BL21: pET23a-*sul1/sul2*) exhibited fitness advantages *in vitro*, which differed from our *in vivo* results (10). This could be attributed to the variation in fitness effects across host genetic backgrounds and external environments (25). Estimated fitness effects *in vitro* differ substantially from those *in vivo*, and everyday laboratory environments might not ideally predict single antibiotic resistance mutation costs or their combined effects (26).

In addition, a seminal review by San Millan (12) summarized that ARG-carrying plasmids in each clone may initially impose a cost, which decreases over time, potentially resulting in a host compensatory evolution event. Based on this perspective, we selected three putative *sul2* gene-dependent CMSs. Upon inspecting the CFUs in each mouse at each time point, after 5 days of competition, some mice had increased frequency of CMSs S2-1, S2-2, and S2-3 clones, whereas the others had reduced frequency. Thus, in some mice, CMSs might compensate for the cost of *sul* resistance; however, a comprehensive analysis of all data suggests that it might be more consistent with general adaptation to the mammalian gut. The short-term cost and long-term within-host evolution of antibiotic resistance exhibit strong personalization and are greatly affected by microbiota composition (26).

In addition, WGS has enabled the identification of mutation trajectories and, in some cases, the correlation of mutations to phenotypes (27). Thus, we identified SNPs in the three CMSs using comparative genomics. The phenomenon of *de novo* evolution of seven identical SNP mutations was observed in three independent strains. It is likely that the seven mutations were already present in the ancestral strain at low frequency and increased in frequency in three of the eight mice to observe such levels of genetic parallelism. In addition, we found a possible fitness response SpoT A26E mutation. Notably, some studies have shown that SpoT mutants, independent of or combined with other mutants, stimulate (p)ppGpp hydrolase activity and regulate physiological activities, such as the metabolism and expression of virulence factors that affect environmental adaptability (28–30). Hence, additional information is required to measure the fitness effect of a mutation in the ancestral background to claim that the spoT mutation is compensatory.

Furthermore, proteomics is a highly suitable tool for studying the adaptive evolution of bacteria from a system-level perspective and highlighting the key players responsible for the adaptive phenotype (31, 32). In this study, we found several upregulated and downregulated proteins in the three CMSs relative to strain P2. The key DEPs were mainly distributed in the two-component system, galactose and tryptophan metabolic pathways, influencing various fitness-related changes in biological functions. In particular, PhoQ expression was significantly upregulated in the three CMSs. PhoQ is a response regulator DNA-binding protein of the two-component (PhoQ/PhoP) system. It acts as an external environmental receptor for Enterobacteria to respond to several stimuli in their mammalian hosts (33). PhoQ senses primary stimuli associated with the host (34, 35). PhoQ-mediated osmosensing enhances bacterial fitness in hyperosmotic environments and can assist in virulence regulation (33). Moreover, the Rcs system, a non-orthodox two-component system of Enterobacteriaceae, integrates environmental signals, regulates gene expression, and alters the physiological behavior of Enterobacteriaceae (36). In the Rcs two-component system, the transcription factor RcsF is a bacterial outer membrane pore protein; upregulated RcsF expression increases bacterial adhesion, invasion, anti-phagocytic survival, biofilm formation, and *in vivo* colonization while reducing bacterial motility (37). RcsB is the core component of the input and output of the complex regulatory network of the Rcs two-component system (38). In the present study, significantly downregulated RcsB expression positively regulates bacterial motility and compensates for the decrease in motility caused by the upregulation of RcsF, demonstrating strong environmental adaptation (39, 40). FliA is also known as a flagellar-specific transcription factor σ28, regulated by the QseB/QseC two-component system (41). FliA upregulation can strengthen flagellin synthesis, improving cell mobility (42, 43). Also, it positively regulates bacterial chemotaxis (44), i.e., the ability of bacteria to sense chemical gradients in the environment and control the movement of flagella and other locomotory organs to achieve proximity or distance to these compounds. This can help bacteria adapt to rapid environmental changes, strive under limited nutrition, and occupy favorable habitats in the ecosystem, enhancing adaptability (45). Regarding the galactose metabolic pathway, UGP2 was significantly upregulated in the three CMSs, increasing UDPG, polysaccharide, and cell wall synthesis to maintain normal cell morphology more effectively and improve their environmental adaptability (21). In addition, DEPs in the galactose metabolic pathway are often associated with glycogen and cell wall synthesis (46). Thus, CMSs may fine-tune DEPs related to glycogen and cell wall synthesis to improve their adaptability to the intestine through carbohydrate utilization and cell morphology maintenance. Regarding the tryptophan metabolic pathway, indole is a critical signaling molecule for *N*-heterocyclic aromatic hydrocarbons, which have a vital impact on the biological functions of biofilm formation, quorum sensing, and the adaptability of antimicrobial-resistant microorganisms in the intestine (47). It can also be used as a signaling molecule to regulate bacterial signal transduction, adhesion ability, and flagella formation (48, 49). Furthermore, indole has a biphasic chemotactic effect on intestinal bacteria. It retains bacteria that have adapted to high indole concentrations through an attractant response and removes bacteria that have not adapted through a repellent response (22). The most significantly upregulated protein was TnaA, which converts tryptophan to indole (50). The significantly upregulated TnaB also helps transport extracellular tryptophan into the cell, stimulates *tnaA* gene expression, and further catalyzes the conversion of tryptophan into indole, thus improving bacterial adaptability (51). Moreover, although the results identified many DEPs in the three CMSs compared with strain P2, *Sul2* expression was not significantly upregulated or downregulated, consistent with the unchanged sulfisoxazole MIC for the CMSs and strain P2. Finally, compared to strain P2, the growth of the CMSs *in vitro* recovered well, further confirming restored fitness. Nevertheless, compared to strain LC, both *in vivo* competition and growth results implied that the sulfonamide-sensitive control strain gradually gains a fitness advantage in the absence of antibiotic pressure over time.

In summary, this study reveals that high fitness costs are incurred by *sul1* and *sul3* gene-dependent *E. coli* strains *in vivo*. However, a fitness advantage was found in three of the eight mice after the intragastric administration of *sul2* gene-dependent *E. coli* on day 9. Three CMSs that outcompeted strain P2 *in vivo* were independently isolated from the three mice. WGS revealed that the *spoT* (77 C→A) mutation site of the CMS is potentially linked to the fitness response of *E. coli*. Furthermore, the results of proteomics analysis revealed the potential bacterial adaptation as follows: first, CMSs can mainly enhance their bacterial motility, environmental stress tolerance, and colonization ability by regulating the expression levels of key proteins in the two-component systems. Second, CMSs may fine-tune DEPs related to glycogen and cell wall synthesis to improve their adaptability to the intestine through carbohydrate utilization and cell morphology maintenance. Finally, by accumulating elevated indole concentrations, CMSs can modulate their chemotaxis, thereby increasing the colonization level and adaptability within the intestine of hosts, ultimately enhancing bacterial survival.

Our study had some limitations. First, although we used the plasmid and strain of *E. coli* employed in previous fitness cost investigations (14, 24, 52), they contain antibiotic-resistance genes that may affect the bacterial response to antimicrobial agents. Future studies should consider the implications for multiple drug resistance and possible shared mechanisms. In addition, reducing the overgrowth of commensal flora in the study mice may have had unintended effects, given the influence of the microbiota on bacterial responses to antibiotic pressure, which warrants further consideration. Ultimately, building upon the findings from comparative genomics and proteomics, future investigations could explore the following: (i) the claim that the spoT mutation is compensatory, necessitating further investigation to contextualize the mutation within the ancestral background and assess its fitness effect; (ii) mechanisms through which elevating TnaA and TnaB expression might augment indole levels within bacterial cells, enhancing the adaptability of *sul* genes responsible for sulfonamide resistance in *E. coli*; (iii) and the feasibility of selecting more CMSs from varied periods, to explore the bacterial adaptation further.

## MATERIALS AND METHODS

### Genetically engineered strains and plasmid construction

The primers and oligonucleotides used for cloning *sul* genes, plasmid recombination, and gene editing of the strain are listed in Table S7. The genetically engineered strains and plasmids used in this study are listed in Table S8.

We used PCR to clone the *sul* genes using 2× Platinum SuperFi II PCR Master Mix (Thermo Fisher Scientific, MA, USA) with *sul*-positive genomic DNA as the template to construct the recombinant plasmids. The PCR amplification products harboring the restriction endonuclease KpnI/XhoI restriction sites were ligated into the parent plasmid pZA2 according to the restriction digestion protocol (New England Biolabs, MA, USA). A chloramphenicol-resistance gene, *cat*, was inserted into the *araB* site of the *E. coli* MG1656 chromosome to construct a chloramphenicol-resistant *E. coli* MG1656 *araB::cat* strain using the CRISPR-Cas9 system from our previous study (53). Plasmids were transferred to *E. coli* MG1656 *araB::cat* through heat shock transformation. All genetically engineered *E. coli* strains were confirmed by PCR amplification, and the products were sequenced at Novogene Biotech using bidirectional sequencing. The PCR components contain 10 µL 2× master mix, 1 µL 10 µM forward primer, 1 µL 10 µM reverse primer, 1 µL genomic DNA (about 5–100 ng), and proper volume nuclease-free water (up to a total of 20 µL). The PCR reaction programs are as follows: initial denaturation 98°C 30 s; denaturation 98°C 5 s, annealing 60°C 10 s, extension 72°C 30 s, 30 cycles; final extension 72°C 5 min.

### Sulfisoxazole susceptibility, MIC, and growth curve analysis

We used the disk diffusion method for the AST on genetically engineered *E. coli* strains. According to CLSI guidelines, we determined the MIC of sulfisoxazole in genetically engineered *E. coli* strains using the broth dilution approach (19). For growth curve analysis, bacterial colonies cultured on LB agar plates were harvested and inoculated into an LB liquid medium. We assessed bacterial growth by measuring the OD_600_ hourly for 24 h.

### Competitive experimental murine infections and screening of CMSs

The *in vivo* competition experiment included female BALB/c mice (5–6 weeks old). Each mouse was single-housed with an individual cage for the fecal pallet collection. They were pretreated as previously described (14, 24, 53). Briefly, 5 g/L streptomycin sulfate salt and 5 g/L glucose (for improving taste) was added to the drinking water for 72 h to reduce the overgrowth of commensal flora, followed by 24 h of purified drinking water to completely clear streptomycin from the *in vivo* system before inoculation. Pairwise growth-competitive strains were also generated. Strains P1, P2, and P3 were independently cultured to achieve 10^6^ CFU and mixed with 10^6^ CFU of strain LC at a 1:1 (vol/vol) ratio. We administered 100 µL of *E. coli* mixture with pairwise growth competition strains to eight mice by oral gavage. Fecal samples were collected, homogenized in 1 mL of phosphate buffer saline (PBS), and serially diluted. Equal volumes of suspensions were cultured on LB agar with the corresponding antibiotics (50 mg/L kanamycin, 25 mg/L chloramphenicol, and 1,024 mg/L sulfisoxazole for strains P1, P2, and P3; 50 mg/L kanamycin and 25 mg/L chloramphenicol for strain LC) to count the numbers of test and control strains (CFUs per gram feces). The CI calculation was performed using equation 1:


(1) $$CI=\frac{mutant\;output/original\;output}{mutant\;input/original\;input}$$

where “mutant” represents P1, P2, and P3, and “original” represents LC; CI = 1 represents equal fitness between the test and control strains; CI > 1 represents greater fitness of the test strains, and CI < 1 denotes reduced fitness of the test strains. Each experiment was repeated thrice for each suspension.

Putative CMSs were observed when the CI was significant >1 (*P* < 0.05). These clones were screened, and their growth curve, sulfisoxazole susceptibility, and MIC were analyzed as described above. Molecular identification of specific resistance genes [*suls, catA1,* and *aph(3′)-Ia*] of the putative CMSs was performed using PCR. One representative CMS from P2-V (CMS S2-1), P2-VI (CMS S2-2), and P2-VII (CMS S2-3) mice was collected, respectively, for further research (detailed information in Table S1).

### Resequencing of the CMSs and comparative genomic analysis

We prepared the genomic DNA of CMSs S2-1, S2-2, and S2-3 using the Dneasy Tissue Kit (Qiagen, Hilden, Germany). The NEBNext Ultra DNA Library Prep Kit for Illumina (New England Biolabs) was used to generate sequencing libraries as per the manufacturer’s recommendations (1 µg DNA/sample). WGS of each strain was conducted on an Illumina NovaSeq PE150 instrument. The original high-throughput sequencing data were converted into raw sequenced data using CASAVA base calling (FASTQ format).

The sample reads were aligned with the genome sequence of the reference strain *E. coli* MG1655 (NC_000913.3) to obtain variation information for CMSs. Alignment was conducted using BWA (version: 0.7.8) and SAMTOOLS (version: 0.1.18) software. The variation map of the whole genome, which showed read coverage and the distribution of SNPs and InDels (insertion and deletion of small fragments, <50 bp), was drawn using Circos (version: 0.64). PCR amplified all SNP and InDels, and the products were sequenced by Novogene Biotech using bidirectional sequencing.

### Experimental competitive infections in mice using CMSs with P2 or LC

The CIs of three confirmed CMSs with strains P2 or LC were analyzed. Notably, to separate the amounts of CMSs from strain P2 (all strains had the same resistance genes), the *spoT* (77 C→A) mutation site of the CMS gene was confirmed by Sanger sequencing of the PCR amplified products.

### Protein extraction, digestion, and TMT labeling

After an 8-h culture in LB medium, CMSs and P2 cells were centrifuged for 10 min at 8,000 × *g* and washed with PBS (pH 7.4) thrice. We utilized SDT buffer (4% sodium dodecyl sulfate, 100 mM Tris-HCl, and 1 mM dithiothreitol, pH 7.6) for protein extraction and cell lysis. Quantification and digestion of proteins were performed as described previously (54). The peptides from each sample underwent desalting treatment on Empore solid phase extraction (SPE) Cartridges C18 (Sigma–Aldrich, MO, USA), vacuum centrifugation, concentration, and reconstitution in 0.1% (vol/vol) formic acid (40 µL). We estimated the peptide content using the ultraviolet spectral density at 280 nm of a 0.1% (g/L) solution with an extinction coefficient of 1.1. Per the manufacturer’s instructions, TMT reagent was used to label each sample’s 100 µg peptide mixture.

### Labeled peptide fractionation using a high pH reversed-phase

A high pH reversed-phase peptide fractionation kit (Thermo Scientific) was used for labeled peptide fractionation. The reconstituted dried peptide mixture was acidified in a 0.1% trifluoroacetic acid solution, followed by loading onto a balanced spin column for reversed-phase fractionation at high pH. The peptides were separated into 10 distinct fractions. Empore SPE C18 (Sigma–Aldrich) was used to desalinize the collected fractions. The peptides were re-dissolved in 12 µL of 0.1% formic acid, and their concentration was estimated at OD_280_.

### Liquid chromatography–tandem mass spectrometry analysis

The peptides were loaded onto a reverse-phase trap column (Acclaim PepMap100, 100 µm × 2 cm, nanoViper C18; Thermo Scientific) connected to the C18 reversed-phase analytical column (Easy Column, 10 cm long, 75 µm inner diameter, 3 µm resin; Thermo Scientific) in buffer A (0.1% formic acid) and separated with a linear gradient of buffer B (84% acetonitrile and 0.1% formic acid) at a flow rate of 300 nL/min. Liquid chromatography–tandem mass spectrometry (LC–MS/MS) analysis was performed using a Q-Exactive mass spectrometer coupled to an Easy nLC instrument for 60/90 min (Thermo Scientific). The MS detection was a survey scan (300–1,800 *m/z*) with a maximum injection time of 10 ms.

### Identification and bioinformatics analysis

The raw MS data were searched using the Mascot engine (Matrix Science, London, UK; version 2.2) embedded in Proteome Discoverer 1.4 software for identification and quantitative analysis. All raw mass data were processed using a false discovery rate <0.01 at the peptide level. The protein ratios were calculated as the median of unique protein peptides. All peptide ratios were normalized using the median protein ratio. The median protein ratio was 1 after normalization.

The proteins with ratios (CMSs to P2) >1.2- or <0.8-fold change with *P*-value < 0.05 were considered upregulated or downregulated DEPs, respectively. The National Center for Biotechnology Information (NCBI) BLAST+ client software (ncbi-blast-2.2.28+-win32.exe), together with InterProScan, served to locally search protein sequences of differentially expressed genes for homologous sequences, followed by the mapping of GO terms and sequence annotation using the software program Blast2GO. We then analyzed the studied proteins using BLAST from the online KEGG database (http://geneontology.org/) to retrieve their KEGG orthology identifications and map them to pathways in KEGG.

### PRM analysis

The DEPs obtained using TMT analysis were selected for further targeted quantification using LC–PRM-MS on a Q-Exactive HF mass spectrometer with an Easy nLC 1200 system. One microgram of equivalent digested peptides was taken from each sample and mixed with 20 fmol standard peptides (PRTC: TASEFDSAIAQDK) for PRM analysis. Raw data were analyzed, and normal peak areas were calculated using the software Skyline 3.5.0 (55).

### Statistical analysis

Origin Pro 2021 software (OriginLab Corporation, MA, USA) was used for statistical analysis. The results were compared using a one-way analysis of variance, followed by the Kruskal–Wallis test. Student’s *t*-test was used to assess the significance of differences among the strains. *P* < 0.05 was considered to be statistically significant.

## Data Availability

The mass spectrometry proteomics data have been deposited to the ProteomeXchange Consortium (https://proteomecentral.proteomexchange.org) via the iProX partner repository with the data set identifier PXD053633. The raw sequencing data of the genomic DNA generated are publicly available in the BioProject database (accession numbers: PRJNA967149, PRJNA967153, and PRJNA967155).

## SUPPLEMENTAL MATERIAL

Table S1 msystems.00836-24-s0001.docx The data from which the competitive index is calculated.

Table S2 msystems.00836-24-s0002.docx Information on differentially expressed proteins of compensatory mutant strains S2-1, S2-2, and S2-3.

Table S3 msystems.00836-24-s0003.docx Differentially expressed protein information related to biological process ontology of compensatory mutant strains S2-1, S2-2, and S2-3.

Table S4 msystems.00836-24-s0004.docx Differentially expressed protein information related to cellular component ontology of compensatory mutant strains S2-1, S2-2, and S2-3.

Supplemental tables msystems.00836-24-s0005.docx Table S5 and TS6.

Tables S7 and S8 msystems.00836-24-s0006.docx Primers, strains, and plasmids.

Legends msystems.00836-24-s0007.docx Supplemental figure and table legends.

Figure S1 msystems.00836-24-s0008.tif Characterization of sulfonamide-resistant test strains P1, P2, and P3 and sulfonamide-sensitive control strain LC.

Figure S2 msystems.00836-24-s0009.tif Characterization of CMS S2-1, S2-2, and S2-3 and parent strain P2.

Figure S3 msystems.00836-24-s0010.tif Top 20 terms from the Kyoto Encyclopedia of Genes and Genomes pathway enrichment analysis of differentially expressed proteins (DEPs).

ASM does not own the copyrights to Supplemental Material that may be linked to, or accessed through, an article. The authors have granted ASM a non-exclusive, world-wide license to publish the Supplemental Material files. Please contact the corresponding author directly for reuse.
