# Supplementary material for: In vivo fitness of sul gene-dependent sulfonamide-resistant Escherichia coli in the mammalian gut
Source: mSystems. 2024 Aug 14;9(9):e00836-24. doi: 10.1128/msystems.00836-24 (PMC11406977; doi:10.1128/msystems.00836-24)
Supplement: Table S1 — The data from which the competitive index is calculated. [file msystems.00836-24-s0001.docx]

**Table S1 The data of the CFU per gram feces of each strain in each mouse from which the competitive index is calculated**

|  | Days | 1 d | | | 3 d | | | 5 d | | | 7 d | | | 9 d | | |
| --- | --- | --- | --- | --- | --- | --- | --- | --- | --- | --- | --- | --- | --- | --- | --- | --- |
|  | Mouse ID | P1 | LC | CI | P1 | LC | CI | P1 | LC | CI | P1 | LC | CI | P1 | LC | CI |
| P1 vs. LC | I | 1.73E+06 | 1.38E+06 | 1.2536 | 4.00E+04 | 1.96E+06 | 0.0204 | 4.00E+02 | 4.57E+05 | 0.0009 | 4.00E+00 | 2.88E+03 | 0.0014 | 4.00E+00 | 9.80E+03 | 0.000408 |
|  | II | 7.20E+05 | 1.04E+06 | 0.6923 | 4.00E+04 | 1.02E+05 | 0.3922 | 1.30E+03 | 1.57E+04 | 0.0828 | 6.00E+01 | 3.70E+03 | 0.0162 | 4.00E+00 | 1.19E+04 | 0.000336 |
|  | III | 4.50E+05 | 4.30E+05 | 1.0465 | 3.50E+05 | 5.40E+05 | 0.6481 | 5.00E+03 | 1.00E+05 | 0.0500 | 4.00E+00 | 2.79E+03 | 0.0014 | 4.00E+00 | 1.05E+05 | 0.000038 |
|  | IV | 1.34E+06 | 1.48E+06 | 0.9054 | 1.30E+05 | 3.51E+06 | 0.0370 | 6.00E+03 | 1.98E+05 | 0.0303 | 4.00E+00 | 1.59E+04 | 0.0003 | 4.00E+00 | 1.79E+04 | 0.000224 |
|  | V | 3.60E+05 | 3.10E+05 | 1.1613 | 2.20E+05 | 5.00E+05 | 0.4400 | 9.00E+03 | 1.89E+05 | 0.0476 | 7.00E+00 | 3.39E+03 | 0.0021 | 4.00E+00 | 2.85E+05 | 0.000014 |
|  | VI | 1.33E+06 | 1.24E+06 | 1.0726 | 4.20E+05 | 1.98E+06 | 0.2121 | 4.00E+03 | 3.82E+05 | 0.0105 | 4.00E+00 | 3.05E+04 | 0.0001 | 4.00E+00 | 1.96E+05 | 0.000020 |
|  | VII | 6.20E+05 | 7.20E+05 | 0.8611 | 3.60E+05 | 1.32E+06 | 0.2727 | 1.60E+04 | 2.31E+05 | 0.0693 | 9.00E+01 | 1.67E+04 | 0.0054 | 4.00E+00 | 1.18E+05 | 0.000034 |
|  | VIII | 8.50E+05 | 1.20E+06 | 0.7083 | 3.00E+05 | 1.91E+06 | 0.1571 | 7.00E+03 | 3.21E+05 | 0.0218 | 5.00E+01 | 5.35E+03 | 0.0093 | 4.00E+00 | 1.70E+04 | 0.000235 |
| CI mean | |  |  | 1.0 |  |  | 0.3 |  |  | 0.04 |  |  | 0.005 |  |  | 0.0002 |
|  | Days | 1 d | | | 3 d | | | 5 d | | | 7 d | | | 9 d | | |
|  | Mouse ID | P2 | LC | CI | P2 | LC | CI | P2 | LC | CI | P2 | LC | CI | P2 | LC | CI |
| P2 vs. LC | I | 1.54E+05 | 9.50E+04 | 1.6211 | 6.70E+04 | 1.17E+05 | 0.5726 | 6.30E+04 | 1.38E+05 | 0.4565 | 1.48E+04 | 1.40E+04 | 1.0571 | 8.70E+03 | 1.50E+04 | 0.5800 |
|  | II | 4.40E+04 | 5.40E+04 | 0.8148 | 3.50E+04 | 1.19E+05 | 0.2941 | 4.00E+03 | 1.77E+05 | 0.0226 | 2.00E+02 | 1.45E+05 | 0.0014 | 4.00E+02 | 8.40E+04 | 0.0048 |
|  | III | 1.00E+05 | 1.73E+05 | 0.5780 | 6.90E+04 | 1.23E+05 | 0.5610 | 2.60E+04 | 1.59E+05 | 0.1635 | 1.00E+02 | 7.19E+04 | 0.0014 | 1.43E+04 | 1.25E+04 | 1.1440 |
|  | IV | 6.80E+04 | 7.80E+04 | 0.8718 | 1.31E+05 | 1.51E+05 | 0.8675 | 3.30E+04 | 2.32E+05 | 0.1422 | 4.00E+02 | 1.59E+05 | 0.0025 | 3.00E+03 | 1.38E+04 | 0.2174 |
|  | **V** | **1.32E+05** | **1.00E+05** | **1.3200** | **8.90E+04** | **1.12E+05** | **0.7946** | **8.60E+04** | **1.36E+05** | **0.6324** | **1.64E+05** | **1.31E+05** | **1.2519** | **1.64E+04** | **7.80E+03** | **2.1026** |
|  | **VI** | **7.90E+05** | **6.50E+05** | **1.2154** | **8.00E+04** | **2.18E+05** | **0.3670** | **1.14E+05** | **1.50E+05** | **0.7600** | **1.10E+05** | **1.01E+05** | **1.0891** | **2.07E+04** | **4.50E+03** | **4.6000** |
|  | **VII** | **1.29E+05** | **1.09E+05** | **1.1835** | **3.00E+04** | **1.58E+05** | **0.1899** | **1.11E+05** | **2.59E+05** | **0.4286** | **4.00E+03** | **4.10E+04** | **0.0976** | **1.15E+04** | **1.60E+03** | **7.1875** |
|  | VIII | 6.70E+04 | 7.40E+04 | 0.9054 | 1.08E+05 | 1.12E+05 | 0.9643 | 6.10E+04 | 2.17E+05 | 0.2811 | 4.50E+04 | 1.36E+05 | 0.3309 | 5.40E+03 | 5.00E+03 | 1.0800 |
| CI mean | |  |  | 1.1 |  |  | 0.6 |  |  | 0.4 |  |  | 0.5 |  |  | 2.1 |
|  | Days | 1 d | | | 3 d | | | 5 d | | | 7 d | | | 9 d | | |
|  | Mouse ID | P3 | LC | CI | P3 | LC | CI | P3 | LC | CI | P3 | LC | CI | P3 | LC | CI |
| P3 vs. LC | I | 5.50E+05 | 1.03E+06 | 0.533981 | 8.20E+05 | 1.90E+06 | 0.431579 | 2.36E+03 | 2.83E+05 | 0.008350 | 7.00E+00 | 2.57E+04 | 0.000272 | 4.00E+00 | 6.10E+03 | 0.000656 |
|  | II | 1.14E+06 | 1.28E+06 | 0.890625 | 2.00E+05 | 1.66E+06 | 0.120482 | 2.70E+01 | 8.20E+05 | 0.000033 | 4.00E+00 | 3.42E+05 | 0.000012 | 4.00E+00 | 1.60E+04 | 0.000250 |
|  | III | 5.60E+05 | 3.80E+05 | 1.473684 | 1.90E+05 | 6.60E+05 | 0.287879 | 4.20E+04 | 2.83E+05 | 0.148410 | 4.10E+02 | 9.59E+03 | 0.042753 | 4.00E+00 | 4.68E+05 | 0.000009 |
|  | IV | 1.28E+05 | 1.54E+05 | 0.831169 | 1.30E+05 | 3.39E+06 | 0.038348 | 8.00E+00 | 2.51E+05 | 0.000032 | 5.00E+00 | 1.84E+05 | 0.000027 | 4.00E+00 | 4.10E+04 | 0.000098 |
|  | V | 6.20E+04 | 6.00E+04 | 1.033333 | 2.40E+04 | 5.01E+05 | 0.047904 | 3.20E+01 | 3.88E+05 | 0.000082 | 9.00E+00 | 5.19E+05 | 0.000017 | 4.00E+00 | 5.90E+03 | 0.000678 |
|  | VI | 7.20E+04 | 6.40E+04 | 1.125000 | 7.20E+04 | 1.88E+05 | 0.382979 | 6.90E+03 | 2.91E+05 | 0.023703 | 4.00E+00 | 2.31E+05 | 0.000017 | 4.00E+00 | 2.50E+03 | 0.001603 |
|  | VII | 4.60E+04 | 4.30E+04 | 1.069767 | 3.10E+04 | 1.98E+05 | 0.156566 | 8.80E+01 | 4.82E+05 | 0.000183 | 4.00E+00 | 9.84E+05 | 0.000004 | 4.00E+00 | 1.10E+04 | 0.000364 |
|  | VIII | 6.10E+04 | 5.10E+04 | 1.196078 | 2.40E+04 | 2.19E+06 | 0.010979 | 1.80E+04 | 1.52E+05 | 0.118421 | 9.00E+01 | 2.69E+04 | 0.003344 | 4.00E+00 | 5.15E+05 | 0.000008 |
| CI mean | |  |  | 1.0 |  |  | 0.2 |  |  | 0.04 |  |  | 0.006 |  |  | 0.0005 |
|  | Days | 1 d | | | 5 d | | | 9 d | | | 13 d | | | 17 d | | |
|  | Mouse ID | S2-1 | P2 | CI | S2-1 | P2 | CI | S2-1 | P2 | CI | S2-1 | P2 | CI | S2-1 | P2 | CI |
| S2-1 vs. P2 | I | 27 | 22 | 1.2273 | 36 | 26 | 1.3846 | 67 | 39 | 1.7179 | 38 | 18 | 2.1111 | 37 | 9 | 4.1111 |
|  | II | 26 | 30 | 0.8667 | 43 | 44 | 0.9773 | 54 | 44 | 1.2273 | 45 | 33 | 1.3636 | 33 | 33 | 1.0000 |
|  | III | 61 | 69 | 0.8841 | 103 | 79 | 1.3038 | 94 | 72 | 1.3056 | 61 | 25 | 2.4400 | 95 | 53 | 1.7925 |
|  | IV | 102 | 99 | 1.0303 | 43 | 161 | 0.2671 | 55 | 209 | 0.2632 | 109 | 139 | 0.7842 | 71 | 111 | 0.6396 |
|  | V | 42 | 41 | 1.0244 | 48 | 72 | 0.6667 | 36 | 108 | 0.3333 | 78 | 96 | 0.8125 | 50 | 49 | 1.0204 |
|  | VI | 26 | 23 | 1.1304 | 36 | 28 | 1.2857 | 45 | 27 | 1.6667 | 42 | 22 | 1.9091 | 46 | 10 | 4.6000 |
|  | VII | 36 | 51 | 0.7059 | 46 | 31 | 1.4839 | 34 | 22 | 1.5455 | 33 | 20 | 1.6500 | 62 | 24 | 2.5833 |
|  | VIII | 31 | 34 | 0.9118 | 34 | 24 | 1.4167 | 35 | 20 | 1.7500 | 52 | 32 | 1.6250 | 18 | 62 | 0.2903 |
| CI mean | |  |  | 1.0 |  |  | 1.1 |  |  | 1.2 |  |  | 1.6 |  |  | 2.0 |
|  | Days | 1 d | | | 5 d | | | 9 d | | | 13 d | | | 17 d | | |
|  | Mouse ID | S2-2 | P2 | CI | S2-2 | P2 | CI | S2-2 | P2 | CI | S2-2 | P2 | CI | S2-2 | P2 | CI |
| S2-2 vs. P2 | I | 49 | 50 | 0.9800 | 26 | 28 | 0.9286 | 35 | 23 | 1.5217 | 59 | 47 | 1.2553 | 45 | 24 | 1.8750 |
|  | II | 29 | 37 | 0.7838 | 32 | 42 | 0.7619 | 28 | 99 | 0.2828 | 32 | 121 | 0.2645 | 12 | 154 | 0.0779 |
|  | III | 33 | 32 | 1.0313 | 51 | 39 | 1.3077 | 32 | 189 | 0.1693 | 14 | 71 | 0.1972 | 32 | 148 | 0.2162 |
|  | IV | 66 | 58 | 1.1379 | 97 | 149 | 0.6510 | 87 | 121 | 0.7190 | 112 | 70 | 1.6000 | 65 | 28 | 2.3214 |
|  | V | 39 | 38 | 1.0263 | 87 | 55 | 1.5818 | 35 | 21 | 1.6667 | 55 | 17 | 3.2353 | 113 | 29 | 3.8966 |
|  | VI | 112 | 131 | 0.8550 | 86 | 76 | 1.1316 | 124 | 80 | 1.5500 | 115 | 79 | 1.4557 | 94 | 58 | 1.6207 |
|  | VII | 26 | 29 | 0.8966 | 44 | 31 | 1.4194 | 50 | 34 | 1.4706 | 48 | 21 | 2.2857 | 79 | 35 | 2.2571 |
|  | VIII | 18 | 24 | 0.7500 | 15 | 30 | 0.5000 | 40 | 28 | 1.4286 | 23 | 30 | 0.7667 | 32 | 27 | 1.1852 |
| CI mean | |  |  | 0.9 |  |  | 1.0 |  |  | 1.1 |  |  | 1.4 |  |  | 1.7 |
|  | Days | 1 d | | | 5 d | | | 9 d | | | 13 d | | | 17 d | | |
|  | Mouse ID | S2-3 | P2 | CI | S2-3 | P2 | CI | S2-3 | P2 | CI | S2-3 | P2 | CI | S2-3 | P2 | CI |
| S2-3 vs. P2 | I | 29 | 33 | 0.8788 | 21 | 33 | 0.6364 | 19 | 49 | 0.3878 | 6 | 36 | 0.1667 | 3 | 45 | 0.0667 |
|  | II | 36 | 39 | 0.9231 | 58 | 44 | 1.3182 | 56 | 38 | 1.4737 | 57 | 28 | 2.0357 | 44 | 21 | 2.0952 |
|  | III | 72 | 55 | 1.3091 | 102 | 83 | 1.2289 | 152 | 74 | 2.0541 | 56 | 20 | 2.8000 | 57 | 11 | 5.1818 |
|  | IV | 41 | 49 | 0.8367 | 41 | 41 | 1.0000 | 36 | 26 | 1.3846 | 35 | 18 | 1.9444 | 27 | 10 | 2.7000 |
|  | V | 78 | 66 | 1.1818 | 55 | 47 | 1.1702 | 29 | 50 | 0.5800 | 4 | 55 | 0.0727 | 2 | 49 | 0.0408 |
|  | VI | 40 | 45 | 0.8889 | 26 | 35 | 0.7429 | 27 | 19 | 1.4211 | 26 | 18 | 1.4444 | 14 | 18 | 0.7778 |
|  | VII | 37 | 39 | 0.9487 | 50 | 34 | 1.4706 | 39 | 24 | 1.6250 | 39 | 21 | 1.8571 | 29 | 14 | 2.0714 |
|  | VIII | 56 | 48 | 1.1667 | 46 | 29 | 1.5862 | 34 | 22 | 1.5455 | 39 | 11 | 3.5455 | 29 | 6 | 4.8333 |
| CI mean | |  |  | 1.0 |  |  | 1.1 |  |  | 1.3 |  |  | 1.7 |  |  | 2.2 |
|  | Days | 1 d | | | 5 d | | | 9 d | | | 13 d | | | 17 d | | |
|  | Mouse ID | S2-1 | LC | CI | S2-1 | LC | CI | S2-1 | LC | CI | S2-1 | LC | CI | S2-1 | LC | CI |
| S2-1 vs. LC | I | 1.35E+05 | 1.19E+05 | 1.1345 | 5.50E+05 | 6.00E+05 | 0.9167 | 5.20E+05 | 1.07E+06 | 0.4860 | 7.60E+04 | 1.88E+05 | 0.4043 | 1.60E+05 | 9.30E+05 | 0.1720 |
|  | II | 1.20E+05 | 8.50E+04 | 1.4118 | 6.60E+05 | 3.20E+05 | 2.0625 | 6.90E+05 | 2.20E+05 | 3.1364 | 8.80E+04 | 3.20E+04 | 2.7500 | 1.12E+05 | 5.20E+04 | 2.1538 |
|  | III | 5.40E+05 | 6.50E+05 | 0.8308 | 1.29E+05 | 1.12E+05 | 1.1518 | 1.49E+06 | 6.10E+05 | 2.4426 | 1.02E+05 | 8.10E+04 | 1.2593 | 5.00E+05 | 7.60E+05 | 0.6579 |
|  | IV | 1.08E+05 | 1.02E+05 | 1.0588 | 1.05E+05 | 5.60E+04 | 1.8750 | 9.10E+05 | 2.50E+05 | 3.6400 | 1.30E+05 | 7.60E+04 | 1.7105 | 1.10E+05 | 1.31E+05 | 0.8397 |
|  | V | 7.60E+05 | 1.04E+06 | 0.7308 | 3.00E+05 | 5.20E+05 | 0.5769 | 2.90E+05 | 4.20E+05 | 0.6905 | 4.70E+04 | 5.20E+04 | 0.9038 | 6.50E+05 | 4.00E+05 | 1.6250 |
|  | VI | 6.50E+05 | 7.80E+05 | 0.8333 | 5.10E+05 | 1.60E+05 | 3.1875 | 1.15E+05 | 3.00E+04 | 3.8333 | 5.90E+04 | 4.70E+04 | 1.2553 | 4.20E+05 | 4.50E+05 | 0.9333 |
|  | VII | 3.90E+05 | 4.90E+05 | 0.7959 | 9.20E+05 | 6.10E+05 | 1.5082 | 5.50E+05 | 5.10E+05 | 1.0784 | 7.30E+04 | 7.00E+04 | 1.0429 | 3.40E+05 | 5.70E+05 | 0.5965 |
|  | VIII | 5.20E+05 | 7.00E+05 | 0.7429 | 1.40E+05 | 6.00E+05 | 0.2333 | 1.20E+05 | 9.00E+05 | 0.1333 | 3.30E+05 | 6.30E+05 | 0.5238 | 8.00E+04 | 8.10E+05 | 0.0988 |
| CI mean | |  |  | 0.9 |  |  | 1.4 |  |  | 1.9 |  |  | 1.2 |  |  | 0.9 |
|  | Days | 1 d | | | 5 d | | | 9 d | | | 13 d | | | 17 d | | |
|  | Mouse ID | S2-2 | LC | CI | S2-2 | LC | CI | S2-2 | LC | CI | S2-2 | LC | CI | S2-2 | LC | CI |
| S2-2 vs. LC | I | 4.90E+05 | 4.90E+05 | 1.0000 | 1.01E+06 | 5.90E+05 | 1.7119 | 1.02E+05 | 3.60E+04 | 2.8333 | 6.70E+05 | 2.70E+05 | 2.4815 | 4.70E+05 | 2.70E+05 | 1.7407 |
|  | II | 6.00E+05 | 5.60E+05 | 1.0714 | 4.20E+05 | 4.40E+05 | 0.9545 | 6.70E+05 | 4.70E+05 | 1.4255 | 3.50E+05 | 3.90E+05 | 0.8974 | 9.20E+04 | 9.60E+04 | 0.9583 |
|  | III | 1.11E+05 | 1.24E+05 | 0.8952 | 1.46E+05 | 1.56E+05 | 0.9359 | 1.92E+05 | 9.70E+04 | 1.9794 | 8.50E+05 | 5.70E+05 | 1.4912 | 4.80E+05 | 3.30E+05 | 1.4545 |
|  | IV | 4.10E+05 | 3.70E+05 | 1.1081 | 6.70E+05 | 3.50E+05 | 1.9143 | 5.20E+05 | 3.10E+05 | 1.6774 | 4.50E+05 | 4.80E+05 | 0.9375 | 6.20E+05 | 8.20E+05 | 0.7561 |
|  | V | 7.40E+05 | 7.20E+05 | 1.0278 | 1.05E+05 | 8.10E+04 | 1.2963 | 7.20E+05 | 3.00E+05 | 2.4000 | 1.25E+05 | 1.15E+05 | 1.0870 | 4.80E+05 | 4.60E+05 | 1.0435 |
|  | VI | 5.90E+05 | 7.30E+05 | 0.8082 | 4.00E+05 | 5.80E+05 | 0.6897 | 3.00E+05 | 9.80E+05 | 0.3061 | 4.40E+05 | 8.90E+05 | 0.4944 | 7.00E+04 | 2.25E+06 | 0.0311 |
|  | VII | 8.60E+05 | 6.80E+05 | 1.2647 | 9.30E+05 | 5.10E+05 | 1.8235 | 5.00E+05 | 2.90E+05 | 1.7241 | 6.60E+05 | 5.40E+05 | 1.2222 | 6.00E+05 | 1.48E+06 | 0.4054 |
|  | VIII | 1.36E+05 | 1.28E+05 | 1.0625 | 1.36E+05 | 7.90E+04 | 1.7215 | 9.10E+05 | 4.30E+05 | 2.1163 | 2.40E+05 | 6.20E+05 | 0.3871 | 1.50E+05 | 8.90E+05 | 0.1685 |
| CI mean | |  |  | 1.0 |  |  | 1.4 |  |  | 1.8 |  |  | 1.1 |  |  | 0.8 |
|  | Days | 1 d | | | 5 d | | | 9 d | | | 13 d | | | 17 d | | |
|  | Mouse ID | S2-3 | LC | CI | S2-3 | LC | CI | S2-3 | LC | CI | S2-3 | LC | CI | S2-3 | LC | CI |
| S2-3 vs. LC | I | 5.60E+05 | 6.90E+05 | 0.8116 | 5.80E+05 | 4.10E+05 | 1.4146 | 1.03E+05 | 5.10E+04 | 2.0196 | 4.80E+05 | 3.70E+05 | 1.2973 | 3.70E+05 | 5.60E+05 | 0.6607 |
|  | II | 3.30E+05 | 3.50E+05 | 0.9429 | 5.60E+05 | 2.90E+05 | 1.9310 | 8.20E+05 | 3.70E+05 | 2.2162 | 5.80E+05 | 3.80E+05 | 1.5263 | 6.50E+05 | 7.50E+05 | 0.8667 |
|  | III | 4.00E+05 | 4.50E+05 | 0.8889 | 3.60E+05 | 1.80E+05 | 2.0000 | 7.90E+05 | 2.60E+05 | 3.0385 | 6.00E+05 | 2.20E+05 | 2.7273 | 6.40E+05 | 2.50E+05 | 2.5600 |
|  | IV | 1.05E+05 | 9.80E+04 | 1.0714 | 1.15E+05 | 4.60E+04 | 2.5000 | 1.16E+05 | 3.00E+04 | 3.8667 | 1.62E+05 | 1.00E+05 | 1.6200 | 3.30E+05 | 5.40E+05 | 0.6111 |
|  | V | 7.00E+05 | 7.60E+05 | 0.9211 | 1.25E+05 | 4.70E+04 | 2.6596 | 6.70E+05 | 2.50E+05 | 2.6800 | 6.30E+05 | 4.30E+05 | 1.4651 | 9.40E+04 | 1.18E+05 | 0.7966 |
|  | VI | 1.30E+05 | 9.60E+04 | 1.3542 | 1.17E+05 | 7.10E+04 | 1.6479 | 9.10E+05 | 3.70E+05 | 2.4595 | 7.10E+05 | 8.30E+05 | 0.8554 | 5.90E+05 | 4.40E+05 | 1.3409 |
|  | VII | 3.90E+05 | 3.60E+05 | 1.0833 | 1.60E+05 | 6.40E+05 | 0.2500 | 4.00E+03 | 1.40E+05 | 0.0286 | 8.00E+04 | 1.72E+06 | 0.0465 | 2.10E+05 | 9.90E+05 | 0.2121 |
|  | VIII | 5.30E+05 | 5.10E+05 | 1.0392 | 3.80E+05 | 5.90E+05 | 0.6441 | 1.00E+05 | 5.60E+05 | 0.1786 | 4.80E+05 | 8.10E+05 | 0.5926 | 2.20E+05 | 9.30E+05 | 0.2366 |
| CI mean | |  |  | 1.0 |  |  | 1.6 |  |  | 2.1 |  |  | 1.3 |  |  | 0.9 |

Note：In the competitive assay of “S2-1 *vs.* P2”, “S2-2 *vs.* P2” and “S2-3 *vs.* P2”, the suspensions of *E. coli* mixture were diluted 10^4^-10^5^, and the CFUs of CMSs (S2-1, S2-2 or S2-3) and the parent strain P2 in each mouse were confirmed by Sanger sequencing based the *spoT* (77 C→A) mutation site, respectively.
