# Supplementary material for: In vivo fitness of sul gene-dependent sulfonamide-resistant Escherichia coli in the mammalian gut
Source: mSystems. 2024 Aug 14;9(9):e00836-24. doi: 10.1128/msystems.00836-24 (PMC11406977; doi:10.1128/msystems.00836-24)
Supplement: Table S2 — Information on differentially expressed proteins of compensatory mutant strains S2-1, S2-2, and S2-3. [file msystems.00836-24-s0002.docx]

**Table S2** Information on differentially expressed proteins of compensatory mutant strains S2-1, S2-2, and S2-3

| **Strain** | **Accession** | **Gene name** | **Description** | **Fold change** | ***P*-value** | **Up/**  **Down** |
| --- | --- | --- | --- | --- | --- | --- |
| S2-1 | A0A8B4PN44 | *tnaA* | Tryptophanase OS = *Escherichia coli* OX = 562 GN = tnaA PE = 4 SV = 1 | 6.135404581 | 9.43108E-06 | Up |
|  | A0A771BBG3 | *tnaA* | Tryptophanase OS = *Escherichia coli* OX = 562 GN = tnaA PE = 4 SV = 1 | 4.354970213 | 0.007784007 | Up |
|  | A0A2X7H313 | *tnaA* | Tryptophanase OS = *Escherichia coli* OX = 562 GN = tnaA PE = 3 SV = 1 | 3.980636105 | 0.002590371 | Up |
|  | A0A7L5V678 | *glpT* | Glycerol-3-phosphate transporter OS = *Escherichia coli* OX = 562 GN = glpT PE = 3 SV = 1 | 3.088374493 | 0.001934294 | Up |
|  | A0A418U310 | *tnaA* | Tryptophanase OS = *Escherichia coli* OX = 562 GN = tnaA PE = 3 SV = 1 | 2.946518264 | 0.002135957 | Up |
|  | A0A376JKT8 | *wrbA_3* | Putative flavoprotein OS = *Escherichia coli* OX = 562 GN = wrbA_3 PE = 4 SV = 1 | 2.843198467 | 0.001904386 | Up |
|  | A0A7A3AYV6 | *hemX* | Uroporphyrinogen-III C-methyltransferase OS = *Escherichia coli* OX = 562 GN = hemX PE = 4 SV = 1 | 2.83634429 | 0.002283085 | Up |
|  | A0A6M0PU76 | *rplI* | 50S ribosomal protein L9 OS = *Escherichia coli* OX = 562 GN = rplI PE = 3 SV = 1 | 2.776524011 | 0.001683163 | Up |
|  | U9XXA4 | *xseB* | Exodeoxyribonuclease 7 small subunit OS = *Escherichia coli* 113290 OX = 1268976 GN = xseB PE = 3 SV = 1 | 2.757016126 | 0.001300749 | Up |
|  | A0A7U9FZE8 | *tnaA* | Tryptophanase OS = *Escherichia coli* TA143 OX = 656437 GN = tnaA PE = 3 SV = 1 | 2.708922 | 0.000833018 | Up |
|  | A0A7U9ASB9 | *tnaA* | Tryptophanase OS = *Escherichia coli* TA206 OX = 656440 GN = tnaA PE = 3 SV = 1 | 2.613764712 | 0.000972112 | Up |
|  | A0A7T8PQW7 | *waaO* | Lipopolysaccharide 3-alpha-galactosyltransferase OS = *Escherichia coli* OX = 562 GN = waaO PE = 4 SV = 1 | 2.541207264 | 0.001003319 | Up |
|  | A0A853RYC6 | BHF52_02880 | Tryptophanase OS = *Escherichia coli* OX = 562 GN = BHF52_02880 PE = 4 SV = 1 | 2.513252504 | 0.039797956 | Up |
|  | A0A376L5P7 | *ompW_1* | Outer membrane protein W OS = *Escherichia coli* OX = 562 GN = ompW_1 PE = 4 SV = 1 | 2.501470552 | 0.03637135 | Up |
|  | A0A6N8PVJ3 | *ackA* | Acetate kinase OS = *Escherichia coli* OX = 562 GN = ackA PE = 3 SV = 1 | 2.459343882 | 0.000539967 | Up |
|  | A0A826R875 | *envC* | Murein hydrolase activator EnvC OS = *Escherichia coli* OX = 562 GN = envC PE = 4 SV = 1 | 2.449932275 | 0.002875896 | Up |
|  | A0A6D0IRU3 | *ibpB* | Small heat shock protein IbpB OS = *Escherichia coli* OX = 562 GN = ibpB PE = 3 SV = 1 | 2.426859942 | 0.009879466 | Up |
|  | A0A7D7DG69 | *zraP* | Zinc resistance-associated protein OS = *Escherichia coli* OX = 562 GN = zraP PE = 3 SV = 1 | 2.415876227 | 0.019182517 | Up |
|  | A0A828MYB1 | *ibpA* | Heat shock chaperone IbpA OS = *Escherichia coli* OX = 562 GN = ibpA PE = 4 SV = 1 | 2.345561462 | 0.000199156 | Up |
|  | A0A777SAD9 | *tnaA* | Tryptophanase OS = *Escherichia coli* OX = 562 GN = tnaA PE = 3 SV = 1 | 2.288181378 | 0.000133613 | Up |
|  | A0A3Y1V1S8 | *galF* | Alpha-D-glucosyl-1-phosphate uridylyltransferase OS = *Escherichia coli* OX = 562 GN = galF PE = 3 SV = 1 | 2.261670613 | 0.001432909 | Up |
|  | A0A2S8JZY6 | *asnA* | Aspartate--ammonia ligase OS = *Escherichia coli* OX = 562 GN = asnA PE = 3 SV = 1 | 2.25857901 | 0.024795795 | Up |
|  | A0A6L9DL34 | FZC17_12545 | Pirin family protein OS = *Escherichia coli* OX = 562 GN = FZC17_12545 PE = 3 SV = 1 | 2.231792033 | 0.00077266 | Up |
|  | A0A8A5J2H5 | JSU10_11650 | LysR family transcriptional regulator OS = *Escherichia coli* H20 OX = 2810409 GN = JSU10_11650 PE = 4 SV = 1 | 2.189499099 | 0.046106042 | Up |
|  | A0A271QYN7 | BIZ41_14720 | Glutamate synthase large subunit OS = *Escherichia coli* OX = 562 GN = BIZ41_14720 PE = 3 SV = 1 | 2.150654968 | 0.002221849 | Up |
|  | A0A7B3MKM9 | HLZ50_09340 | DUF892 family protein OS = *Escherichia coli* OX = 562 GN = HLZ50_09340 PE = 4 SV = 1 | 2.142229221 | 0.017758545 | Up |
|  | A0A6M0PVJ7 | *pyrG* | CTP synthase OS = *Escherichia coli* OX = 562 GN = pyrG PE = 3 SV = 1 | 2.092063421 | 0.000503546 | Up |
|  | A0A6N9MUA6 | *glpQ* | Glycerophosphodiester phosphodiesterase OS = *Escherichia coli* OX = 562 GN = glpQ PE = 4 SV = 1 | 2.068963094 | 0.00046644 | Up |
|  | A0A376J8Y2 | *tnaB* | Aromatic amino acid permease OS = *Escherichia coli* OX = 562 GN = tnaB PE = 3 SV = 1 | 2.018941084 | 0.007637725 | Up |
|  | W9AMC1 | *tnaA* | Tryptophanase OS = *Escherichia coli* O25b : H4-ST131 OX = 941322 GN = tnaA PE = 3 SV = 1 | 2.015809663 | 0.022841234 | Up |
|  | A0A7H9LNK0 | *cadA* | Lysine decarboxylase CadA OS = *Escherichia coli* OX = 562 GN = cadA PE = 3 SV = 1 | 2.008677358 | 0.00441928 | Up |
|  | A0A829JIU5 | G938_01661 | Sulfatase YdeN OS = *Escherichia coli* UMEA 3200-1 OX = 1281213 GN = G938_01661 PE = 4 SV = 1 | 1.99129831 | 0.00273141 | Up |
|  | A0A418H5N2 | *ybtA* | Yersiniabactin transcriptional regulator YbtA (Fragment) OS = *Escherichia coli* OX = 562 GN = ybtA PE = 4 SV = 1 | 1.987177405 | 0.016396439 | Up |
|  | A0A826JV02 | *fadD* | Long-chain-fatty-acid--CoA ligase FadD OS = *Escherichia coli* OX = 562 GN = fadD PE = 4 SV = 1 | 1.984774707 | 0.004989453 | Up |
|  | A0A377CV50 | *mazE* | Antitoxin MazE OS = *Escherichia coli* OX = 562 GN = mazE PE = 4 SV = 1 | 1.981197811 | 0.015980765 | Up |
|  | A0A2K3TM93 | C2M16_24085 | Uncharacterized protein OS = *Escherichia coli* OX = 562 GN = C2M16_24085 PE = 4 SV = 1 | 1.976583764 | 0.011417312 | Up |
|  | A0A5B1FAQ8 | *glpT* | GlpT protein OS = *Escherichia coli* OX = 562 GN = glpT PE = 3 SV = 1 | 1.974307429 | 0.022355333 | Up |
|  | A0A376S463 | *tnaA* | Tryptophanase OS = *Escherichia coli* OX = 562 GN = tnaA PE = 4 SV = 1 | 1.931456737 | 0.010575201 | Up |
|  | A0A7U9FZE7 | ECMG_00118 | Glutamate decarboxylase beta (GAD-beta) OS = *Escherichia coli* TA143 OX = 656437 GN = ECMG_00118 PE = 4 SV = 1 | 1.926752343 | 0.004373562 | Up |
|  | A0A828FSI3 | *cybC* | Cytochrome b562 OS = *Escherichia coli* OX = 562 GN = cybC PE = 4 SV = 1 | 1.912924152 | 0.009849001 | Up |
|  | A0A1E5M202 | *ydfZ* | Selenium carrying protein OS = *Escherichia coli* OX = 562 GN = ydfZ PE = 4 SV = 1 | 1.908209925 | 0.033388672 | Up |
|  | A0A2A3VUL7 | *tnaA* | Tryptophanase OS = *Escherichia coli* OX = 562 GN = tnaA PE = 3 SV = 1 | 1.905709436 | 0.000103715 | Up |
|  | A0A8B5PFJ8 | *tnaA* | Tryptophanase OS = *Escherichia coli* OX = 562 GN = tnaA PE = 4 SV = 1 | 1.897916623 | 0.004503666 | Up |
|  | A0A6L4XMK4 | GP710_03455 | Fumarate hydratase class I OS = *Escherichia coli* OX = 562 GN = GP710_03455 PE = 3 SV = 1 | 1.85006505 | 0.002197858 | Up |
|  | A0A7Z1F0N1 | APX88_03850 | Fumarate hydratase class I OS = *Escherichia coli* OX = 562 GN = APX88_03850 PE = 3 SV = 1 | 1.839626245 | 0.025305896 | Up |
|  | A0A7H2C767 | *clpB* | Chaperone protein ClpB OS = *Escherichia coli* (strain K12) OX = 83333 GN = clpB PE = 3 SV = 1 | 1.837537422 | 0.035036157 | Up |
|  | A0A7U2BDF2 | *ompW* | Outer membrane protein OmpW OS = *Escherichia coli* OX = 562 GN = ompW PE = 4 SV = 1 | 1.822420706 | 0.024959577 | Up |
|  | A0A7I8Z834 | *glpQ* | GlpQ protein OS = *Escherichia coli* OX = 562 GN = glpQ PE = 4 SV = 1 | 1.820323053 | 0.000507927 | Up |
|  | A0A6C9IM12 | GKF86_27255 | SAM-dependent methyltransferase OS = *Escherichia coli* OX = 562 GN = GKF86_27255 PE = 4 SV = 1 | 1.814411573 | 0.005959874 | Up |
|  | A8A6H0 | *tnaA* | Tryptophanase OS = *Escherichia coli* O9 : H4 (strain HS) OX = 331112 GN = tnaA PE = 3 SV = 1 | 1.803030142 | 0.008039792 | Up |
|  | Q46774 | *glpQ* | Glycerophosphoryl diester phosphodiesterase (Fragment) OS = *Escherichia coli* OX = 562 GN = glpQ PE = 4 SV = 1 | 1.799850306 | 0.006494417 | Up |
|  | A0A855W463 | DB282_27280 | Protease (Fragment) OS = *Escherichia coli* OX = 562 GN = DB282_27280 PE = 4 SV = 1 | 1.796614735 | 0.034186831 | Up |
|  | A0A376NYE6 | *nrfA* | Cytochrome c-552 OS = *Escherichia coli* OX = 562 GN = nrfA PE = 3 SV = 1 | 1.792087138 | 0.00211672 | Up |
|  | A0A377BSF6 | *ompW_1* | Outer membrane protein W OS = *Escherichia coli* OX = 562 GN = ompW_1 PE = 4 SV = 1 | 1.77432183 | 0.040984478 | Up |
|  | A0A6D0Y0N5 | G3M51_04305 | DUF4765 family protein OS = *Escherichia coli* OX = 562 GN = G3M51_04305 PE = 4 SV = 1 | 1.757703878 | 0.011866567 | Up |
|  | A0A827KW76 | *hybB* | Ni/Fe-hydrogenase cytochrome b subunit OS = *Escherichia coli* OX = 562 GN = hybB PE = 4 SV = 1 | 1.745846888 | 0.008560235 | Up |
|  | A0A6C9LHP4 | *yedE* | Selenium metabolism membrane protein YedE/FdhT OS = *Escherichia coli* OX = 562 GN = yedE PE = 3 SV = 1 | 1.739727303 | 0.002383908 | Up |
|  | U9YDR6 | HMPREF1589_01601 | Malate synthase OS = *Escherichia coli* 113290 OX = 1268976 GN = HMPREF1589_01601 PE = 3 SV = 1 | 1.738442531 | 0.020353825 | Up |
|  | A0A6N6YDL1 | *glpQ* | Glycerophosphodiester phosphodiesterase OS = *Escherichia coli* OX = 562 GN = glpQ PE = 4 SV = 1 | 1.729333534 | 0.007499508 | Up |
|  | A0A6D0C6T7 | *cadA* | Lysine decarboxylase CadA OS = *Escherichia coli* OX = 562 GN = cadA PE = 3 SV = 1 | 1.714455431 | 0.028706349 | Up |
|  | A0A826SG38 | *glpD* | Glycerol-3-phosphate dehydrogenase OS = *Escherichia coli* OX = 562 GN = glpD PE = 4 SV = 1 | 1.707730885 | 0.000592284 | Up |
|  | A0A6M0PUJ1 | G4V03_08605 | Fumarate hydratase class I OS = *Escherichia coli* OX = 562 GN = G4V03_08605 PE = 3 SV = 1 | 1.698823222 | 0.002233753 | Up |
|  | A0A2P6IUD5 | *rfaF* | ADP-heptose--LPS heptosyltransferase RfaF OS = *Escherichia coli* OX = 562 GN = rfaF PE = 4 SV = 1 | 1.697972882 | 0.003336992 | Up |
|  | A0A5P0J560 | *uspF* | Universal stress protein UspF OS = *Escherichia coli* OX = 562 GN = uspF PE = 3 SV = 1 | 1.677946418 | 0.001057857 | Up |
|  | A0A5B9GFD8 | unknow | Glycosyltransferase OS = *Escherichia coli* OX = 562 PE = 4 SV = 1 | 1.677363381 | 0.002517435 | Up |
|  | A0A777TQ89 | *feoB* | Ferrous iron transport protein B OS = *Escherichia coli* OX = 562 GN = feoB PE = 3 SV = 1 | 1.658496444 | 0.002151037 | Up |
|  | A0A7U9QDI6 | *torS* | Histidine kinase OS = *Escherichia coli* O145 : H28 OX = 1078034 GN = torS PE = 4 SV = 1 | 1.642937283 | 0.000109576 | Up |
|  | F4SG82 | ECHG_02138 | Glycerophosphoryl diester phosphodiesterase GlpQ OS = *Escherichia coli* H736 OX = 656414 GN = ECHG_02138 PE = 4 SV = 1 | 1.638533196 | 0.00551111 | Up |
|  | A0A3Q0N2H5 | CR539_20030 | Hydrogenase 2 large subunit OS = *Escherichia coli* OX = 562 GN = CR539_20030 PE = 3 SV = 1 | 1.636432616 | 0.028469132 | Up |
|  | A0A6C9EEH0 | GKG27_26075 | Dipeptidase (Fragment) OS = *Escherichia coli* OX = 562 GN = GKG27_26075 PE = 3 SV = 1 | 1.635706516 | 0.003906056 | Up |
|  | D7Y8S5 | *fucO* | Lactaldehyde reductase OS = *Escherichia coli* (strain MS 115-1) OX = 749537 GN = fucO PE = 4 SV = 1 | 1.63427473 | 0.00021758 | Up |
|  | A0A6G4BZV9 | *tnaA* | Tryptophanase OS = *Escherichia coli* OX = 562 GN = tnaA PE = 3 SV = 1 | 1.633630563 | 0.000225222 | Up |
|  | P0ACD8 | *hyaB* | Hydrogenase-1 large chain OS = *Escherichia coli* (strain K12) OX = 83333 GN = hyaB PE = 1 SV = 1 | 1.633156668 | 0.039013219 | Up |
|  | A0A828JSI0 | EH88_005446 | Acetyltransferase OS = *Escherichia coli* OX = 562 GN = EH88_005446 PE = 4 SV = 1 | 1.631765875 | 0.006277066 | Up |
|  | A0A827L415 | BRV34_001673 | NAD(P)/FAD-dependent oxidoreductase OS = *Escherichia coli* OX = 562 GN = BRV34_001673 PE = 4 SV = 1 | 1.627704006 | 0.002614073 | Up |
|  | A0A0A1A5H6 | *rihC* | Non-specific ribonucleoside hydrolase RihC OS = *Escherichia coli* OX = 562 GN = rihC PE = 3 SV = 1 | 1.624944449 | 0.000381299 | Up |
|  | A0A1X3LUN7 | EAZG_00482 | Citrate lyase alpha chain OS = *Escherichia coli* TA249 OX = 656441 GN = EAZG_00482 PE = 4 SV = 1 | 1.623748653 | 0.001586981 | Up |
|  | A0A7L5V1W3 | *mscK* | Mechanosensitive channel MscK OS = *Escherichia coli* OX = 562 GN = mscK PE = 3 SV = 1 | 1.623302277 | 0.045146051 | Up |
|  | A0A2U9A1S8 | *ompC* | Outer membrane porin protein OmpC (Fragment) OS = *Escherichia coli* OX = 562 GN = ompC PE = 3 SV = 1 | 1.620438609 | 0.005362647 | Up |
|  | A0A8A8NRS0 | *gntP* | Gluconate permease GntP OS = *Escherichia coli* OX = 562 GN = gntP PE = 4 SV = 1 | 1.614774316 | 0.006062597 | Up |
|  | A0A6L4XL24 | *secA* | Protein translocase subunit SecA OS = *Escherichia coli* OX = 562 GN = secA PE = 2 SV = 1 | 1.608284829 | 0.011304296 | Up |
|  | A0A5D8QXC9 | *ompT* | Omptin family outer membrane protease OmpT (Fragment) OS = *Escherichia coli* OX = 562 GN = ompT PE = 4 SV = 1 | 1.594430933 | 0.00154192 | Up |
|  | A0A377BSB0 | *tnaA_1* | Tryptophanase OS = *Escherichia coli* OX = 562 GN = tnaA_1 PE = 4 SV = 1 | 1.589334128 | 0.00477781 | Up |
|  | A0A0K4EBA5 | *ccpA* | Transcriptional regulator YcjW OS = *Escherichia coli* OX = 562 GN = ccpA PE = 4 SV = 1 | 1.588226795 | 0.015143526 | Up |
|  | W1ETT4 | unknow | Shikimate kinase I OS = *Escherichia coli* ISC7 OX = 1432555 PE = 4 SV = 1 | 1.585224678 | 0.007893082 | Up |
|  | A0A376I8B9 | *cydA* | Cytochrome d ubiquinol oxidase subunit 1 OS = *Escherichia coli* OX = 562 GN = cydA PE = 3 SV = 1 | 1.577116171 | 0.012610622 | Up |
|  | A0A843MJ33 | *nfuA* | Iron-sulfur cluster biogenesis protein NfuA OS = *Escherichia coli* OX = 562 GN = nfuA PE = 4 SV = 1 | 1.575203644 | 0.00139125 | Up |
|  | A0A3K0JQE8 | D9J61_18285 | FAD-binding protein OS = *Escherichia coli* OX = 562 GN = D9J61_18285 PE = 4 SV = 1 | 1.570268021 | 0.000293955 | Up |
|  | A0A377AU17 | *cbpA_2* | Chaperone modulatory protein CbpM OS = *Escherichia coli* OX = 562 GN = cbpA_2 PE = 3 SV = 1 | 1.56991039 | 0.000185944 | Up |
|  | K4XKM3 | *tnaA* | Tryptophanase/L-cysteine desulfhydrase, PLP-dependent OS = *Escherichia coli* O111 : H11 str. CVM9455 OX = 1165939 GN = tnaA PE = 4 SV = 1 | 1.569323319 | 0.009726487 | Up |
|  | A0A6D0FG95 | *flgM* | Anti-sigma-28 factor OS = *Escherichia coli* OX = 562 GN = flgM PE = 3 SV = 1 | 1.566567306 | 0.008208433 | Up |
|  | A0A1Q6BB69 | *hemL* | Glutamate-1-semialdehyde 2,1-aminomutase OS = *Escherichia coli* OX = 562 GN = hemL PE = 3 SV = 1 | 1.565890868 | 0.013415763 | Up |
|  | Q5IIG4 | *aspC* | Aspartate amino transferase (Fragment) OS = *Escherichia coli* OX = 562 GN = aspC PE = 4 SV = 1 | 1.5617861 | 0.00328659 | Up |
|  | A0A0K3PZ85 | *rmuC* | DNA recombination protein RmuC OS = *Escherichia coli* OX = 562 GN = rmuC PE = 3 SV = 1 | 1.545881246 | 0.000147694 | Up |
|  | A0A660HE83 | A9X72_14540 | Uncharacterized protein OS = *Escherichia coli* OX = 562 GN = A9X72_14540 PE = 4 SV = 1 | 1.543053677 | 0.001195244 | Up |
|  | A0A376S994 | *rmlB_1* | dTDP-D-glucose 4,6-dehydratase rmlB OS = *Escherichia coli* OX = 562 GN = rmlB_1 PE = 4 SV = 1 | 1.534483709 | 0.00213284 | Up |
|  | A0A2T3THJ9 | C7B02_06005 | Monooxygenase OS = *Escherichia coli* OX = 562 GN = C7B02_06005 PE = 4 SV = 1 | 1.533565573 | 0.006114463 | Up |
|  | A0A376HT67 | *gor* | Glutathione reductase OS = *Escherichia coli* OX = 562 GN = gor PE = 3 SV = 1 | 1.532825311 | 0.01986729 | Up |
|  | A0A777HIK4 | *glpT* | Glycerol-3-phosphate transporter OS = *Escherichia coli* OX = 562 GN = glpT PE = 3 SV = 1 | 1.527943451 | 0.004976291 | Up |
|  | A0A377E1X8 | *rcsF* | RcsF--phosphorelay glucose and zinc sensor OS = *Escherichia coli* OX = 562 GN = rcsF PE = 4 SV = 1 | 1.526511902 | 0.002103595 | Up |
|  | A0A827E0G2 | CX692_001784 | Tryptophanase OS = *Escherichia coli* OX = 562 GN = CX692_001784 PE = 4 SV = 1 | 1.526001496 | 0.007890407 | Up |
|  | W1WA20 | Q609_ECAC02517G0001 | Uncharacterized protein (Fragment) OS = *Escherichia coli* DORA_A_5_14_21 OX = 1403943 GN = Q609_ECAC02517G0001 PE = 4 SV = 1 | 1.52559946 | 0.002593972 | Up |
|  | I2UGI9 | EC40522_5562 | tRNA ligases class II (D, K and N) OS = *Escherichia coli* 4.0522 OX = 869681 GN = EC40522_5562 PE = 4 SV = 1 | 1.525070484 | 0.000285232 | Up |
|  | A0A826ZD52 | *hdhA* | 7-alpha-hydroxysteroid dehydrogenase OS = *Escherichia coli* OX = 562 GN = hdhA PE = 4 SV = 1 | 1.525029153 | 0.001184867 | Up |
|  | A0A6D0USM6 | *glpQ* | Glycerophosphodiester phosphodiesterase (Fragment) OS = *Escherichia coli* OX = 562 GN = glpQ PE = 4 SV = 1 | 1.524093038 | 0.001564884 | Up |
|  | A0A843ZG16 | E4K54_28035 | GTP-binding protein (Fragment) OS = *Escherichia coli* OX = 562 GN = E4K54_28035 PE = 4 SV = 1 | 1.52351065 | 0.016932739 | Up |
|  | Q56UC7 | unknow | Host specificity protein (Fragment) OS = *Escherichia coli* OX = 562 PE = 4 SV = 1 | 1.522675183 | 0.000950081 | Up |
|  | W1F624 | unknow | Uptake hydrogenase large subunit OS = *Escherichia coli* ISC7 OX = 1432555 PE = 3 SV = 1 | 1.51006108 | 0.005942977 | Up |
|  | A0A3L0VV29 | D9F05_05105 | Glycerophosphodiester phosphodiesterase OS = *Escherichia coli* OX = 562 GN = D9F05_05105 PE = 4 SV = 1 | 1.507348108 | 0.011509592 | Up |
|  | A0A1X3I3W6 | *lysS* | Lysine--tRNA ligase OS = *Escherichia coli* M056 OX = 656415 GN = lysS PE = 3 SV = 1 | 1.506998666 | 0.00522716 | Up |
|  | A0A377CFY3 | *hchA* | Protein/nucleic acid deglycase HchA OS = *Escherichia coli* OX = 562 GN = hchA PE = 2 SV = 1 | 1.501059251 | 0.004368544 | Up |
|  | A0A6N8PGB0 | FQ021_16075 | GntR family transcriptional regulator OS = *Escherichia coli* OX = 562 GN = FQ021_16075 PE = 4 SV = 1 | 1.484964995 | 0.009405263 | Up |
|  | A0A7I8ZNN1 | *ydcH* | YdcH protein OS = *Escherichia coli* OX = 562 GN = ydcH PE = 4 SV = 1 | 1.48248864 | 0.036745594 | Up |
|  | A0A3U5UQ40 | *uspD* | Universal stress protein OS = *Escherichia coli* OX = 562 GN = uspD PE = 3 SV = 1 | 1.482411965 | 0.002311117 | Up |
|  | A0A2X1NE56 | *citT* | Citrate carrier OS = *Escherichia coli* OX = 562 GN = citT PE = 3 SV = 1 | 1.480778128 | 0.021140066 | Up |
|  | A0A5F1W0G1 | *zraP* | Zinc resistance-associated protein OS = *Escherichia coli* OX = 562 GN = zraP PE = 3 SV = 1 | 1.477390713 | 0.029834698 | Up |
|  | A0A828L8H3 | *cydB* | Cytochrome d ubiquinol oxidase subunit II OS = *Escherichia coli* OX = 562 GN = cydB PE = 4 SV = 1 | 1.471549582 | 0.018780779 | Up |
|  | A0A2U2VLW9 | *glpQ* | Glycerophosphodiester phosphodiesterase OS = *Escherichia coli* OX = 562 GN = glpQ PE = 4 SV = 1 | 1.469561152 | 0.021001785 | Up |
|  | A0A828UTS3 | *nrfA* | Formate-dependent cytochrome c nitrite reductase, c552 subunit OS = *Escherichia coli* 3.2608 OX = 869679 GN = nrfA PE = 4 SV = 1 | 1.467980767 | 0.00062036 | Up |
|  | D3GU02 | *dcuR* | Transcriptional regulatory protein OS = *Escherichia coli* O44 : H18 (strain 042 / EAEC) OX = 216592 GN = dcuR PE = 4 SV = 1 | 1.467556022 | 0.044730977 | Up |
|  | A0A826XAA9 | *putP* | Sodium/proline symporter PutP OS = *Escherichia coli* OX = 562 GN = putP PE = 4 SV = 1 | 1.466839647 | 0.00831131 | Up |
|  | E3PNJ1 | ETEC_1839 | t(6)A37 threonylcarbamoyladenosine biosynthesis protein TsaB OS = *Escherichia coli* O78 : H11 (strain H10407 / ETEC) OX = 316401 GN = ETEC_1839 PE = 3 SV = 1 | 1.46395542 | 0.044907287 | Up |
|  | A0A3L0VY65 | *mglB* | D-galactose-binding periplasmic protein OS = *Escherichia coli* OX = 562 GN = mglB PE = 3 SV = 1 | 1.463562434 | 0.004566468 | Up |
|  | A0A6N8K9S4 | *ribD* | 5-amino-6-(5-phosphoribosylamino)uracil reductase (Fragment) OS = *Escherichia coli* OX = 562 GN = ribD PE = 4 SV = 1 | 1.460288771 | 0.006555864 | Up |
|  | A0A844UUA4 | *galF* | UTP--glucose-1-phosphate uridylyltransferase GalF (Fragment) OS = *Escherichia coli* OX = 562 GN = galF PE = 4 SV = 1 | 1.458772715 | 0.007071513 | Up |
|  | D8AE21 | HMPREF9530_04824 | Uncharacterized protein OS = *Escherichia coli* (strain MS 21-1) OX = 749527 GN = HMPREF9530_04824 PE = 4 SV = 1 | 1.45798272 | 0.021924117 | Up |
|  | A0A828AVB4 | A8W81_002270 | Oxidative stress defense protein OS = *Escherichia coli* OX = 562 GN = A8W81_002270 PE = 4 SV = 1 | 1.454175807 | 0.014092028 | Up |
|  | A0A376MVG2 | *phoQ* | Sensor histidine protein kinase/phosphatase PhoQ OS = *Escherichia coli* OX = 562 GN = phoQ PE = 4 SV = 1 | 1.449356206 | 0.008226603 | Up |
|  | Q1R585 | *yhjA* | Probable cytochrome C peroxidase OS = *Escherichia coli* (strain UTI89 / UPEC) OX = 364106 GN = yhjA PE = 4 SV = 1 | 1.449071767 | 0.015773002 | Up |
|  | A0A5C9AIS4 | FWK02_15270 | DUF406 domain-containing protein (Fragment) OS = *Escherichia coli* OX = 562 GN = FWK02_15270 PE = 3 SV = 1 | 1.444601684 | 0.000618134 | Up |
|  | E6BDM7 | *dps* | DNA protection during starvation protein OS = *Escherichia coli* MS 85-1 OX = 679202 GN = dps PE = 3 SV = 1 | 1.443403914 | 0.019483154 | Up |
|  | A0A3R0VNX2 | *cbpA* | Curved DNA-binding protein OS = *Escherichia coli* OX = 562 GN = cbpA PE = 3 SV = 1 | 1.439816538 | 0.005598475 | Up |
|  | A0A3L0W8Z5 | *pykF* | Pyruvate kinase OS = *Escherichia coli* OX = 562 GN = pykF PE = 3 SV = 1 | 1.439516938 | 0.00754118 | Up |
|  | A0A854W4Y1 | COD50_08130 | Replication protein OS = *Escherichia coli* OX = 562 GN = COD50_08130 PE = 4 SV = 1 | 1.438982381 | 0.011806825 | Up |
|  | A0A826XCX3 | *lysS* | Lysine--tRNA ligase OS = *Escherichia coli* OX = 562 GN = lysS PE = 4 SV = 1 | 1.438789582 | 0.018224232 | Up |
|  | A0A376I0P4 | *pykA* | Pyruvate kinase OS = *Escherichia coli* OX = 562 GN = pykA PE = 3 SV = 1 | 1.432758458 | 0.026011651 | Up |
|  | A0A828S740 | ECSTEC7V_3840 | Enhancing lycopene biosynthesis protein 2 OS = *Escherichia coli* STEC_7v OX = 754082 GN = ECSTEC7V_3840 PE = 4 SV = 1 | 1.432318734 | 0.001347519 | Up |
|  | A0A7I8Z9P3 | ETECE36_02358 | Cytochrome c-type biogenesis protein OS = *Escherichia coli* OX = 562 GN = ETECE36_02358 PE = 3 SV = 1 | 1.431602059 | 0.001192782 | Up |
|  | A0A7H9QR77 | HVX31_00565 | Uncharacterized protein OS = *Escherichia coli* OX = 562 GN = HVX31_00565 PE = 4 SV = 1 | 1.430414443 | 0.001456218 | Up |
|  | A0A827E4E7 | A2F99_001537 | Anaerobic C4-dicarboxylate transporter OS = *Escherichia coli* OX = 562 GN = A2F99_001537 PE = 4 SV = 1 | 1.426949488 | 0.013220733 | Up |
|  | A0A789R943 | *kdsD* | Arabinose 5-phosphate isomerase OS = *Escherichia coli* OX = 562 GN = kdsD PE = 3 SV = 1 | 1.426110288 | 0.009650988 | Up |
|  | A0A826NBH0 | CXJ73_004090 | Putative transporter OS = *Escherichia coli* OX = 562 GN = CXJ73_004090 PE = 4 SV = 1 | 1.425998831 | 0.002958987 | Up |
|  | A0A826TR55 | *ivy* | C-lysozyme inhibitor OS = *Escherichia coli* OX = 562 GN = ivy PE = 4 SV = 1 | 1.425993428 | 0.009535524 | Up |
|  | A0A646J549 | *mug* | G/U mismatch-specific DNA glycosylase OS = *Escherichia coli* OX = 562 GN = mug PE = 3 SV = 1 | 1.422207043 | 0.003095587 | Up |
|  | A0A376I2K4 | *aspS* | Aspartate--tRNA ligase OS = *Escherichia coli* OX = 562 GN = aspS PE = 3 SV = 1 | 1.421964032 | 0.005802313 | Up |
|  | A0A0L1C3K3 | *fumB* | Fumarate hydratase class I OS = *Escherichia coli* OX = 562 GN = fumB PE = 3 SV = 1 | 1.414071258 | 0.004382608 | Up |
|  | A0A6D0H4X4 | *clpB* | Chaperone protein ClpB OS = *Escherichia coli* OX = 562 GN = clpB PE = 3 SV = 1 | 1.409625253 | 0.029692212 | Up |
|  | A0A2X6R2J2 | *hyb0* | NiFe hydrogenase OS = *Escherichia coli* OX = 562 GN = hyb0 PE = 3 SV = 1 | 1.407050743 | 0.009152029 | Up |
|  | A0A3Y3V6Q4 | GKF89_16485 | Uncharacterized protein OS = *Escherichia coli* OX = 562 GN = GKF89_16485 PE = 4 SV = 1 | 1.403279058 | 0.00781851 | Up |
|  | A0A7B5PWP4 | HNC52_18380 | C-type cytochrome OS = *Escherichia coli* OX = 562 GN = HNC52_18380 PE = 4 SV = 1 | 1.400803713 | 8.57569E-06 | Up |
|  | A0A3L0W6Q2 | *rapA* | RNA polymerase-associated protein RapA OS = *Escherichia coli* OX = 562 GN = rapA PE = 3 SV = 1 | 1.400732878 | 0.013121577 | Up |
|  | A0A827NRZ2 | *hdhA* | 7-alpha-hydroxysteroid dehydrogenase OS = *Escherichia coli* OX = 562 GN = hdhA PE = 4 SV = 1 | 1.399639067 | 0.000281597 | Up |
|  | A0A376TN50 | *rpoB_4* | DNA-directed RNA polymerase OS = *Escherichia coli* OX = 562 GN = rpoB_4 PE = 4 SV = 1 | 1.398796841 | 0.014214433 | Up |
|  | A0A2T1LIF6 | C6985_13480 | Glycerol-3-phosphate transporter OS = *Escherichia coli* OX = 562 GN = C6985_13480 PE = 3 SV = 1 | 1.39745126 | 0.022452565 | Up |
|  | A0A376D6E8 | *preT_2* | Putative oxidoreductase OS = *Escherichia coli* OX = 562 GN = preT_2 PE = 4 SV = 1 | 1.395890177 | 0.000521329 | Up |
|  | A0A827TFN0 | F9407_14020 | AAA family ATPase OS = *Escherichia coli* OX = 562 GN = F9407_14020 PE = 4 SV = 1 | 1.395662694 | 0.00335464 | Up |
|  | A0A0H2Z3V4 | *gor* | Glutathione reductase Gor OS = *Escherichia coli* O1 : K1 / APEC OX = 405955 GN = gor PE = 3 SV = 1 | 1.393681979 | 0.000641991 | Up |
|  | A0A827QN65 | GQW07_19440 | Inhibitor of g-type lysozyme OS = *Escherichia coli* OX = 562 GN = GQW07_19440 PE = 4 SV = 1 | 1.39284879 | 0.006128669 | Up |
|  | A0A1X3LME0 | EAXG_05135 | Protease 7 (Protease VII) (Omptin) (Outermembrane protein 3B) (Protease A) OS = *Escherichia coli* TA054 OX = 656433 GN = EAXG_05135 PE = 3 SV = 1 | 1.392493078 | 0.026414532 | Up |
|  | A0A2X7FW49 | SAMEA3753300_00011 | DNA injection protein OS = *Escherichia coli* OX = 562 GN = SAMEA3753300_00011 PE = 4 SV = 1 | 1.386360171 | 0.004313581 | Up |
|  | A0A376I976 | *yajQ* | UPF0234 protein YajQ OS = *Escherichia coli* OX = 562 GN = yajQ PE = 3 SV = 1 | 1.384981391 | 0.017472787 | Up |
|  | A0A7H9QZ14 | *yahK* | NADPH-dependent aldehyde reductase YahK OS = *Escherichia coli* OX = 562 GN = yahK PE = 3 SV = 1 | 1.383120292 | 0.002612264 | Up |
|  | A0A140NGD0 | ECBD_3794 | Alpha,alpha-phosphotrehalase OS = *Escherichia coli* (strain B / BL21-DE3) OX = 469008 GN = ECBD_3794 PE = 3 SV = 1 | 1.379826103 | 0.002477889 | Up |
|  | A0A2T1LIJ2 | C6985_13475 | Glycerophosphodiester phosphodiesterase OS = *Escherichia coli* OX = 562 GN = C6985_13475 PE = 4 SV = 1 | 1.377697177 | 0.001732818 | Up |
|  | A0A829CUZ6 | ECMP0215528_1535 | Outer membrane insertion C-terminal signal domain protein OS = *Escherichia coli* MP021552.8 OX = 1116133 GN = ECMP0215528_1535 PE = 4 SV = 1 | 1.377419482 | 0.038239749 | Up |
|  | A0A826RS52 | FPS82_03185 | DUF2756 family protein OS = *Escherichia coli* OX = 562 GN = FPS82_03185 PE = 4 SV = 1 | 1.374827391 | 0.006395609 | Up |
|  | A0A5B9B029 | FTV90_14225 | SecY/secA suppressor protein OS = *Escherichia coli* OX = 562 GN = FTV90_14225 PE = 4 SV = 1 | 1.371430072 | 3.38271E-05 | Up |
|  | A0A822U513 | *yfgG* | Protein OS = *Escherichia coli* OX = 562 GN = yfgG PE = 4 SV = 1 | 1.371041098 | 0.001898509 | Up |
|  | A0A377L8B3 | *livF_1* | Leucine/isoleucine/valine transporter ATP-binding subunit OS = *Escherichia coli* OX = 562 GN = livF_1 PE = 4 SV = 1 | 1.370868164 | 0.042682554 | Up |
|  | A0A828R0G1 | *mnmC* | UPF0209 protein yfcK OS = *Escherichia coli* 3431 OX = 670892 GN = mnmC PE = 4 SV = 1 | 1.369686854 | 0.022180022 | Up |
|  | A0A827LBS1 | *focA* | Formate transporter FocA OS = *Escherichia coli* OX = 562 GN = focA PE = 4 SV = 1 | 1.366968804 | 0.000555041 | Up |
|  | A0A829K1L3 | H000_01269 | Uncharacterized protein OS = *Escherichia coli* UMEA 3899-1 OX = 1281275 GN = H000_01269 PE = 4 SV = 1 | 1.366528502 | 0.005913788 | Up |
|  | A0A829A9C6 | A1Y7_04499 | Transcriptional activator rfaH OS = *Escherichia coli* KTE119 OX = 1182710 GN = A1Y7_04499 PE = 4 SV = 1 | 1.366167081 | 0.007135183 | Up |
|  | A0A826VXD8 | *dpiB* | Sensor histidine kinase DpiB OS = *Escherichia coli* OX = 562 GN = dpiB PE = 4 SV = 1 | 1.365116798 | 0.001953475 | Up |
|  | A0A6N8Q227 | *yoaH* | UPF0181 protein YoaH OS = *Escherichia coli* OX = 562 GN = yoaH PE = 3 SV = 1 | 1.364817539 | 0.00478291 | Up |
|  | A0A2X3A7G0 | *maeB* | NADP-dependent malic enzyme OS = *Escherichia coli* OX = 562 GN = maeB PE = 3 SV = 1 | 1.364196397 | 0.032895716 | Up |
|  | A0A0B1LVL6 | PU06_29190 | Toxin YhaV (Fragment) OS = *Escherichia coli* OX = 562 GN = PU06_29190 PE = 4 SV = 1 | 1.363411727 | 0.003879117 | Up |
|  | A0A2A2BPI3 | BFL24_10370 | Sulfatase OS = *Escherichia coli* OX = 562 GN = BFL24_10370 PE = 3 SV = 1 | 1.362677051 | 0.00269747 | Up |
|  | P14407 | *fumB* | Fumarate hydratase class I, anaerobic OS = *Escherichia coli* (strain K12) OX = 83333 GN = fumB PE = 1 SV = 2 | 1.361829636 | 0.006081997 | Up |
|  | A0A2X6RA66 | *bssR* | Biofilm formation regulator BssR OS = *Escherichia coli* OX = 562 GN = bssR PE = 4 SV = 1 | 1.361372204 | 0.004381399 | Up |
|  | A0A2A3WWM5 | BB545_05105 | NADH oxidoreductase OS = *Escherichia coli* OX = 562 GN = BB545_05105 PE = 4 SV = 1 | 1.361185001 | 0.002670781 | Up |
|  | A0A6D0GV09 | *glmS* | Glutamine--fructose-6-phosphate aminotransferase [isomerizing] OS = *Escherichia coli* OX = 562 GN = glmS PE = 3 SV = 1 | 1.360838634 | 0.001111639 | Up |
|  | P76549 | *yffR* | Uncharacterized protein YffR OS = *Escherichia coli* (strain K12) OX = 83333 GN = yffR PE = 3 SV = 1 | 1.360544442 | 0.001880453 | Up |
|  | A0A7A9SUH6 | *dmsB* | Dimethylsulfoxide reductase subunit B OS = *Escherichia coli* OX = 562 GN = dmsB PE = 4 SV = 1 | 1.357555599 | 0.000140864 | Up |
|  | A0A829FR97 | WCS_00361 | Phage protein OS = *Escherichia coli* KTE14 OX = 1169333 GN = WCS_00361 PE = 4 SV = 1 | 1.357425246 | 0.008508699 | Up |
|  | A0A7H9M0Z7 | *ebgR* | Transcriptional regulator EbgR OS = *Escherichia coli* OX = 562 GN = ebgR PE = 4 SV = 1 | 1.356906491 | 0.020067667 | Up |
|  | A0A6M1HUR9 | G5632_26260 | YciK family oxidoreductase (Fragment) OS = *Escherichia coli* OX = 562 GN = G5632_26260 PE = 4 SV = 1 | 1.355067049 | 0.044250632 | Up |
|  | B6I4S1 | *zapB* | Cell division protein ZapB OS = *Escherichia coli* (strain SE11) OX = 409438 GN = zapB PE = 3 SV = 1 | 1.35492226 | 0.008848747 | Up |
|  | A0A6D0EIL2 | *nrdD* | Anaerobic ribonucleoside-triphosphate reductase (Fragment) OS = *Escherichia coli* OX = 562 GN = nrdD PE = 4 SV = 1 | 1.353770668 | 0.035755374 | Up |
|  | A0A7A2X3S7 | *ivy* | C-lysozyme inhibitor OS = *Escherichia coli* OX = 562 GN = ivy PE = 4 SV = 1 | 1.352742988 | 0.002658081 | Up |
|  | A0A3L0WAU3 | *dnaK* | Chaperone protein DnaK OS = *Escherichia coli* OX = 562 GN = dnaK PE = 2 SV = 1 | 1.352715739 | 0.026768724 | Up |
|  | A0A5F1DU45 | *lpxP* | Lipid A biosynthesis palmitoleoyltransferase OS = *Escherichia coli* OX = 562 GN = lpxP PE = 3 SV = 1 | 1.351460326 | 0.01720093 | Up |
|  | A0A853WGK5 | *copA* | Cu+ exporting ATPase OS = *Escherichia coli* OX = 562 GN = copA PE = 4 SV = 1 | 1.350579585 | 0.008385901 | Up |
|  | A0A377DBF7 | *bfr* | Bacterioferritin OS = *Escherichia coli* OX = 562 GN = bfr PE = 3 SV = 1 | 1.349329194 | 1.27243E-05 | Up |
|  | A0A7B5NX36 | *dgoD* | D-galactonate dehydratase OS = *Escherichia coli* OX = 562 GN = dgoD PE = 3 SV = 1 | 1.348406935 | 0.036926784 | Up |
|  | A0A8B5HP37 | *gpr* | L-glyceraldehyde 3-phosphate reductase OS = *Escherichia coli* OX = 562 GN = gpr PE = 4 SV = 1 | 1.347466008 | 0.019382415 | Up |
|  | A0A080IHA6 | AC28_4007 | Uncharacterized protein OS = *Escherichia coli* 1-250-04_S3_C2 OX = 1444163 GN = AC28_4007 PE = 4 SV = 1 | 1.347444158 | 0.004275027 | Up |
|  | A0A826MHE4 | *ldtB* | L,D-transpeptidase OS = *Escherichia coli* OX = 562 GN = ldtB PE = 4 SV = 1 | 1.347118036 | 0.046716868 | Up |
|  | A0A6D0J0L5 | GP946_18280 | EAL domain-containing protein OS = *Escherichia coli* OX = 562 GN = GP946_18280 PE = 4 SV = 1 | 1.346811458 | 0.004122852 | Up |
|  | A0A1M0PEB6 | BK292_14295 | Maltose O-acetyltransferase OS = *Escherichia coli* OX = 562 GN = BK292_14295 PE = 3 SV = 1 | 1.346540843 | 0.001709721 | Up |
|  | A0A827RU54 | *bioD* | ATP-dependent dethiobiotin synthetase BioD OS = *Escherichia coli* OX = 562 GN = bioD PE = 4 SV = 1 | 1.343796288 | 0.00196748 | Up |
|  | A0A2H4TXE8 | CV83915_03928 | Glycerophosphodiester phosphodiesterase OS = *Escherichia coli* OX = 562 GN = CV83915_03928 PE = 4 SV = 1 | 1.34336535 | 0.001031104 | Up |
|  | A0A826X2M8 | *cspD* | Cold shock-like protein CspD OS = *Escherichia coli* OX = 562 GN = cspD PE = 4 SV = 1 | 1.341864979 | 0.019538773 | Up |
|  | A0A2A3VTG9 | *nrfD* | Cytochrome c nitrite reductase subunit NrfD OS = *Escherichia coli* OX = 562 GN = nrfD PE = 3 SV = 1 | 1.341244113 | 0.005759328 | Up |
|  | D8AEF0 | HMPREF9530_04954 | Sugar-binding domain protein OS = *Escherichia coli* (strain MS 21-1) OX = 749527 GN = HMPREF9530_04954 PE = 4 SV = 1 | 1.340652979 | 0.003964316 | Up |
|  | A0A2X1KKJ6 | *ydgH_1* | Protein YdgH OS = *Escherichia coli* OX = 562 GN = ydgH_1 PE = 4 SV = 1 | 1.340205681 | 0.007931042 | Up |
|  | A0A376RLB5 | *artI* | Arginine ABC transporter, substrate-binding protein OS = *Escherichia coli* OX = 562 GN = artI PE = 3 SV = 1 | 1.338043587 | 0.014515815 | Up |
|  | Q8XAW4 | Z2210 | Putative sulfatase OS = *Escherichia coli* O157 : H7 OX = 83334 GN = Z2210 PE = 3 SV = 2 | 1.336242557 | 0.00215629 | Up |
|  | E6BHM5 | *sbcD* | Nuclease SbcCD subunit D OS = *Escherichia coli* MS 85-1 OX = 679202 GN = sbcD PE = 3 SV = 1 | 1.335379252 | 0.000396355 | Up |
|  | A0A0H2V996 | *dsdC* | D-serine deaminase activator OS = *Escherichia coli* O6 : H1 (strain CFT073 / ATCC 700928 / UPEC) OX = 199310 GN = dsdC PE = 3 SV = 1 | 1.332187552 | 0.004325305 | Up |
|  | A0A789MAE1 | *katG* | Catalase-peroxidase OS = *Escherichia coli* OX = 562 GN = katG PE = 3 SV = 1 | 1.330933585 | 0.01547768 | Up |
|  | A0A826TZQ7 | FQF29_15680 | C-lysozyme inhibitor OS = *Escherichia coli* OX = 562 GN = FQF29_15680 PE = 4 SV = 1 | 1.329226981 | 0.003247507 | Up |
|  | P37194 | *slp* | Outer membrane protein Slp OS = *Escherichia coli* (strain K12) OX = 83333 GN = slp PE = 1 SV = 1 | 1.328154003 | 0.009828639 | Up |
|  | A0A5B9AZ62 | FTV90_19080 | Abasic site processing protein OS = *Escherichia coli* OX = 562 GN = FTV90_19080 PE = 3 SV = 1 | 1.326982649 | 0.043314391 | Up |
|  | A0A376HZX6 | *cobT* | Nicotinate-nucleotide--dimethylbenzimidazole phosphoribosyltransferase OS = *Escherichia coli* OX = 562 GN = cobT PE = 3 SV = 1 | 1.326884237 | 0.03122068 | Up |
|  | A0A376I7C2 | *ompN_2* | Porin OmpN OS = *Escherichia coli* OX = 562 GN = ompN_2 PE = 3 SV = 1 | 1.325973401 | 0.009275463 | Up |
|  | A0A6M0PSQ9 | *aceB* | Malate synthase OS = *Escherichia coli* OX = 562 GN = aceB PE = 3 SV = 1 | 1.325309241 | 0.032448396 | Up |
|  | A0A826QPY9 | *menE* | O-succinylbenzoate--CoA ligase OS = *Escherichia coli* OX = 562 GN = menE PE = 4 SV = 1 | 1.322655708 | 0.012535151 | Up |
|  | P76440 | *preT* | NAD-dependent dihydropyrimidine dehydrogenase subunit PreT OS = *Escherichia coli* (strain K12) OX = 83333 GN = preT PE = 1 SV = 1 | 1.320258656 | 0.004219619 | Up |
|  | A0A2X8FSG0 | SAMEA3751407_04734 | Putative kinase OS = *Escherichia coli* OX = 562 GN = SAMEA3751407_04734 PE = 3 SV = 1 | 1.318278695 | 0.005253422 | Up |
|  | A0A777RRA0 | GFY48_05910 | Aldo/keto reductase OS = *Escherichia coli* OX = 562 GN = GFY48_05910 PE = 4 SV = 1 | 1.317853998 | 0.000183909 | Up |
|  | A0A2S5UB44 | *proY* | Proline-specific permease ProY OS = *Escherichia coli* OX = 562 GN = proY PE = 4 SV = 1 | 1.317075419 | 0.003265943 | Up |
|  | A0A6D0M0X1 | GRW80_28915 | UDP-3-O-(3-hydroxymyristoyl)glucosamine N-acyltransferase (Fragment) OS = *Escherichia coli* OX = 562 GN = GRW80_28915 PE = 4 SV = 1 | 1.315752937 | 0.000782468 | Up |
|  | A0A377AWG1 | *dacC* | Serine-type D-Ala-D-Ala carboxypeptidase OS = *Escherichia coli* OX = 562 GN = dacC PE = 3 SV = 1 | 1.313412999 | 0.004317046 | Up |
|  | A0A6D0IS19 | *nagK* | N-acetyl-D-glucosamine kinase OS = *Escherichia coli* OX = 562 GN = nagK PE = 3 SV = 1 | 1.31335151 | 0.020022163 | Up |
|  | A0A8B1C9Z1 | *csiE* | Stationary phase inducible protein CsiE OS = *Escherichia coli* O128ac : H12 OX = 2822132 GN = csiE PE = 4 SV = 1 | 1.313265442 | 0.00578196 | Up |
|  | A0A6N9MN81 | *lysS* | Lysine--tRNA ligase OS = *Escherichia coli* OX = 562 GN = lysS PE = 3 SV = 1 | 1.311691399 | 0.010700691 | Up |
|  | A0A3L0W137 | *groL* | 60 kDa chaperonin OS = *Escherichia coli* OX = 562 GN = groL PE = 3 SV = 1 | 1.309857135 | 0.038965666 | Up |
|  | A0A7D7HRL8 | *ulaD* | 3-keto-L-gulonate-6-phosphate decarboxylase UlaD OS = *Escherichia coli* OX = 562 GN = ulaD PE = 2 SV = 1 | 1.309197867 | 0.005907934 | Up |
|  | A0A5D8RSR4 | E0I42_27095 | Lipoprotein (Fragment) OS = *Escherichia coli* OX = 562 GN = E0I42_27095 PE = 4 SV = 1 | 1.308239373 | 0.009798106 | Up |
|  | A0A2S5UBH6 | C4M78_14045 | Uncharacterized protein OS = *Escherichia coli* OX = 562 GN = C4M78_14045 PE = 4 SV = 1 | 1.307856803 | 0.005934642 | Up |
|  | A0A826X499 | CSE52_001296 | Fumarate hydratase OS = *Escherichia coli* OX = 562 GN = CSE52_001296 PE = 4 SV = 1 | 1.307488251 | 0.003354136 | Up |
|  | A0A827L354 | *lysA* | Diaminopimelate decarboxylase OS = *Escherichia coli* OX = 562 GN = lysA PE = 4 SV = 1 | 1.307160858 | 0.003526827 | Up |
|  | A0A6M0PYN9 | *glpQ* | Glycerophosphodiester phosphodiesterase OS = *Escherichia coli* OX = 562 GN = glpQ PE = 4 SV = 1 | 1.306538186 | 0.004670715 | Up |
|  | A0A7T2N4B4 | *fliA* | RNA polymerase sigma factor FliA OS = *Escherichia coli* OX = 562 GN = fliA PE = 4 SV = 1 | 1.304726332 | 0.001866425 | Up |
|  | A0A765T6L6 | *ompC* | Porin OmpC OS = *Escherichia coli* OX = 562 GN = ompC PE = 3 SV = 1 | 1.304179 | 0.000258561 | Up |
|  | A0A7D7PKU0 | *cpdB* | 2',3'-cyclic-nucleotide 2'-phosphodiesterase/3'-nucleotidase OS = *Escherichia coli* OX = 562 GN = cpdB PE = 3 SV = 1 | 1.302869468 | 0.003201164 | Up |
|  | A0A6M0PS67 | *glnS* | Glutamine--tRNA ligase OS = *Escherichia coli* OX = 562 GN = glnS PE = 3 SV = 1 | 1.300972491 | 0.013431517 | Up |
|  | A0A7H9LX11 | *fucO* | Lactaldehyde reductase OS = *Escherichia coli* OX = 562 GN = fucO PE = 4 SV = 1 | 1.300291548 | 0.033391023 | Up |
|  | A0A6N8PZM9 | GRW77_14420 | YciK family oxidoreductase OS = *Escherichia coli* OX = 562 GN = GRW77_14420 PE = 4 SV = 1 | 1.300102337 | 0.023764959 | Up |
|  | A0A6L4XNV3 | *dacC* | Serine-type D-Ala-D-Ala carboxypeptidase OS = *Escherichia coli* OX = 562 GN = dacC PE = 3 SV = 1 | 1.29909392 | 0.024180706 | Up |
|  | L7UYD3 |  | OmpC porin (Fragment) OS = *Escherichia coli* OX = 562 PE = 3 SV = 1 | 1.29857611 | 0.007633785 | Up |
|  | A0A8B4IWC9 | *mdh* | Malate dehydrogenase OS = *Escherichia coli* OX = 562 GN = mdh PE = 4 SV = 1 | 1.298190392 | 0.003266468 | Up |
|  | A0A6F8QMT4 | *sul2* | Dihydropteroate synthase OS = *Escherichia coli* OX = 562 GN = sul2 PE = 3 SV = 1 | 1.294463288 | 0.036290499 | Up |
|  | A0A376LG23 | *groL* | 60 kDa chaperonin OS = *Escherichia coli* OX = 562 GN = groL PE = 3 SV = 1 | 1.293947227 | 0.003152133 | Up |
|  | A0A828UQB5 | *lysA* | Diaminopimelate decarboxylase OS = *Escherichia coli* 3.2608 OX = 869679 GN = lysA PE = 4 SV = 1 | 1.292840928 | 0.029726594 | Up |
|  | A0A3L0W067 | D9F05_14910 | Uncharacterized protein OS = *Escherichia coli* OX = 562 GN = D9F05_14910 PE = 4 SV = 1 | 1.292519738 | 0.004786346 | Up |
|  | A0A829GBE0 | WG3_02894 | Arginine ABC transporter permease ArtQ OS = *Escherichia coli* KTE36 OX = 1169353 GN = WG3_02894 PE = 4 SV = 1 | 1.292123785 | 0.003317618 | Up |
|  | A0A5D8SZL8 | E0I42_08635 | NAD(P)H oxidoreductase OS = *Escherichia coli* OX = 562 GN = E0I42_08635 PE = 4 SV = 1 | 1.291601129 | 0.000577982 | Up |
|  | E6BF25 | HMPREF9350_00923 | 4Fe-4S binding domain protein OS = *Escherichia coli* MS 85-1 OX = 679202 GN = HMPREF9350_00923 PE = 4 SV = 1 | 1.291018317 | 0.017876939 | Up |
|  | A0A376KZC4 | *ddlA_1* | D-alanine--D-alanine ligase OS = *Escherichia coli* OX = 562 GN = ddlA_1 PE = 4 SV = 1 | 1.290115749 | 0.034318501 | Up |
|  | A0A4D2QXI7 | *yegP* | YegP family protein OS = *Escherichia coli* OX = 562 GN = yegP PE = 3 SV = 1 | 1.290111493 | 0.034880781 | Up |
|  | A0A2X1N950 | *mliC* | Putative lipoprotein OS = *Escherichia coli* OX = 562 GN = mliC PE = 4 SV = 1 | 1.289665182 | 0.00099602 | Up |
|  | A0A377HG86 | *qseG_2* | Putative lipoprotein OS = *Escherichia coli* OX = 562 GN = qseG_2 PE = 4 SV = 1 | 1.289137511 | 0.000450485 | Up |
|  | A0A2X1JNR5 | *rihC* | Non-specific ribonucleoside hydrolase RihC OS = *Escherichia coli* OX = 562 GN = rihC PE = 3 SV = 1 | 1.288633946 | 0.012155669 | Up |
|  | A0A4Y8G431 | BON92_16580 | Dihydrothymine dehydrogenase OS = *Escherichia coli* OX = 562 GN = BON92_16580 PE = 3 SV = 1 | 1.286660018 | 9.35936E-05 | Up |
|  | A0A376PP12 | *yrfD* | DNA utilization protein HofM OS = *Escherichia coli* OX = 562 GN = yrfD PE = 4 SV = 1 | 1.285854315 | 0.013331339 | Up |
|  | E2QDH5 | unknow | Uncharacterized protein OS = *Escherichia coli* LF82 OX = 591946 PE = 4 SV = 1 | 1.284167533 | 0.024217505 | Up |
|  | A0A6D0GXH7 | *pepA* | Probable cytosol aminopeptidase OS = *Escherichia coli* OX = 562 GN = pepA PE = 3 SV = 1 | 1.283769211 | 0.027994989 | Up |
|  | A0A843M827 | D9F92_10695 | Uncharacterized protein OS = *Escherichia coli* OX = 562 GN = D9F92_10695 PE = 4 SV = 1 | 1.282970715 | 0.036932313 | Up |
|  | A0A7D7DMB0 | *glpQ* | Glycerophosphodiester phosphodiesterase OS = *Escherichia coli* OX = 562 GN = glpQ PE = 4 SV = 1 | 1.282146248 | 0.047557652 | Up |
|  | A0A2T1LK09 | C6985_09105 | Mannitol dehydrogenase family protein OS = *Escherichia coli* OX = 562 GN = C6985_09105 PE = 4 SV = 1 | 1.281996129 | 0.020079295 | Up |
|  | A0A403LPP3 | DLX40_17495 | DeoR/GlpR transcriptional regulator OS = *Escherichia coli* OX = 562 GN = DLX40_17495 PE = 4 SV = 1 | 1.281970919 | 0.002195664 | Up |
|  | A0A765X6R8 | GGB84_001845 | L,D-transpeptidase family protein OS = *Escherichia coli* OX = 562 GN = GGB84_001845 PE = 3 SV = 1 | 1.28140303 | 0.017107846 | Up |
|  | A0A7A2WTW6 | *tar* | Methyl-accepting chemotaxis protein II OS = *Escherichia coli* OX = 562 GN = tar PE = 4 SV = 1 | 1.28001379 | 0.046493008 | Up |
|  | A0A6N4KJZ2 | BON98_19775 | Uncharacterized protein OS = *Escherichia coli* OX = 562 GN = BON98_19775 PE = 4 SV = 1 | 1.278714209 | 0.005682857 | Up |
|  | A0A3L5AFZ3 | AAS29_000798 | DUF1471 domain-containing protein OS = *Escherichia coli* OX = 562 GN = AAS29_000798 PE = 4 SV = 1 | 1.27797826 | 0.033263048 | Up |
|  | A0A2U9KS47 | *narX_2* | Histidine kinase OS = *Escherichia coli* OX = 562 GN = narX_2 PE = 4 SV = 1 | 1.276517652 | 0.004973769 | Up |
|  | A0A5F1T656 | *grpE* | Protein GrpE OS = *Escherichia coli* OX = 562 GN = grpE PE = 3 SV = 1 | 1.275050737 | 0.000476704 | Up |
|  | A0A826V574 | *wzzB* | LPS O-antigen chain length determinant protein WzzB OS = *Escherichia coli* OX = 562 GN = wzzB PE = 4 SV = 1 | 1.274961018 | 0.017973986 | Up |
|  | A0A3L0VW82 | D9F05_02350 | Thioredoxin peroxidase OS = *Escherichia coli* OX = 562 GN = D9F05_02350 PE = 4 SV = 1 | 1.274877207 | 0.018554578 | Up |
|  | P0A952 | *speG* | Spermidine N(1)-acetyltransferase OS = *Escherichia coli* O157 : H7 OX = 83334 GN = speG PE = 3 SV = 2 | 1.274772742 | 0.005029277 | Up |
|  | A0A826Y1R6 | FFF58_15265 | DNA-binding transcriptional regulator H-NS OS = *Escherichia coli* OX = 562 GN = FFF58_15265 PE = 4 SV = 1 | 1.274751862 | 0.015106267 | Up |
|  | A0A6L9DJA7 | FZC17_15120 | Glycerol-3-phosphate dehydrogenase OS = *Escherichia coli* OX = 562 GN = FZC17_15120 PE = 3 SV = 1 | 1.274494892 | 0.015616022 | Up |
|  | D3QUE6 | *hdeB* | Acid stress chaperone HdeB OS = *Escherichia coli* O55 : H7 (strain CB9615 / EPEC) OX = 701177 GN = hdeB PE = 3 SV = 1 | 1.274374752 | 0.01590927 | Up |
|  | A0A8A8NQQ1 | *idnD* | L-idonate 5-dehydrogenase OS = *Escherichia coli* OX = 562 GN = idnD PE = 4 SV = 1 | 1.274247313 | 7.4525E-05 | Up |
|  | A0A376VYA9 | *yaeP* | UPF0253 protein YaeP OS = *Escherichia coli* OX = 562 GN = yaeP PE = 3 SV = 1 | 1.273344838 | 0.003755179 | Up |
|  | A0A3W2VQN2 | *ldtE* | L,D-transpeptidase LdtE OS = *Escherichia coli* OX = 562 GN = ldtE PE = 3 SV = 1 | 1.271895273 | 3.91162E-05 | Up |
|  | A0A4U9U1E4 | *cpdB* | 2',3'-cyclic-nucleotide 2'-phosphodiesterase/3'-nucleotidase OS = *Escherichia coli* OX = 562 GN = cpdB PE = 3 SV = 1 | 1.270229285 | 0.020050327 | Up |
|  | A0A243V6D8 | AZZ83_000699 | Hydrogenase 1 maturation protease (Fragment) OS = *Escherichia coli* OX = 562 GN = AZZ83_000699 PE = 3 SV = 1 | 1.268952292 | 0.021629601 | Up |
|  | A0A6N8PRW7 | *cmk* | Cytidylate kinase OS = *Escherichia coli* OX = 562 GN = cmk PE = 3 SV = 1 | 1.268753649 | 0.030598296 | Up |
|  | A0A827ELZ6 | B6R17_001942 | Class II glutamine amidotransferase OS = *Escherichia coli* OX = 562 GN = B6R17_001942 PE = 4 SV = 1 | 1.268591581 | 0.000163859 | Up |
|  | A0A6D1D2E5 | G5595_24695 | Transcriptional repressor PurR (Fragment) OS = *Escherichia coli* OX = 562 GN = G5595_24695 PE = 4 SV = 1 | 1.267253528 | 0.036439233 | Up |
|  | A0A3L9GVF5 | *tatA* | Sec-independent protein translocase protein TatA OS = *Escherichia coli* OX = 562 GN = tatA PE = 3 SV = 1 | 1.266856201 | 0.001746245 | Up |
|  | A0A6L7A3K9 | *nanA* | N-acetylneuraminate lyase (Fragment) OS = *Escherichia coli* OX = 562 GN = nanA PE = 3 SV = 1 | 1.266508225 | 0.026787576 | Up |
|  | A0A828AWN3 | *narQ* | Nitrate/nitrite two-component system sensor histidine kinase NarQ OS = *Escherichia coli* OX = 562 GN = narQ PE = 4 SV = 1 | 1.26644774 | 0.001720903 | Up |
|  | A0A2I8SX17 | C2U48_31755 | DUF3313 domain-containing protein OS = *Escherichia coli* OX = 562 GN = C2U48_31755 PE = 4 SV = 1 | 1.26621495 | 0.002747999 | Up |
|  | A0A5P0J533 | EIZ93_02765 | Transcriptional regulatory protein OS = *Escherichia coli* OX = 562 GN = EIZ93_02765 PE = 4 SV = 1 | 1.265019064 | 0.000289895 | Up |
|  | A0A7U2WD15 | *idnO* | Gluconate 5-dehydrogenase OS = *Escherichia coli* O84 : H7 OX = 2697517 GN = idnO PE = 3 SV = 1 | 1.26183163 | 6.62196E-05 | Up |
|  | A0A6M7H4E4 | *nanK* | N-acetylmannosamine kinase OS = *Escherichia coli* O157 : H7 OX = 83334 GN = nanK PE = 3 SV = 1 | 1.261527044 | 0.010831889 | Up |
|  | A0A2X7G1M0 | *mbeA_4* | Putative mobilization protein 3 OS = *Escherichia coli* OX = 562 GN = mbeA_4 PE = 4 SV = 1 | 1.261403335 | 0.042156883 | Up |
|  | A0A7D7I6K9 | *pmbA* | Metalloprotease PmbA OS = *Escherichia coli* OX = 562 GN = pmbA PE = 3 SV = 1 | 1.259768593 | 0.036735219 | Up |
|  | A0A4T4XPV4 | DXT71_16195 | L-arabinose-binding periplasmic protein OS = *Escherichia coli* OX = 562 GN = DXT71_16195 PE = 3 SV = 1 | 1.2596137 | 0.007526701 | Up |
|  | A0A2X3K8E1 | *rpoC_5* | DNA-directed RNA polymerase OS = *Escherichia coli* OX = 562 GN = rpoC_5 PE = 4 SV = 1 | 1.258036612 | 0.038178975 | Up |
|  | A0A192EWN6 | *cpdB* | 2',3'-cyclic-nucleotide 2'-phosphodiesterase/3'-nucleotidase OS = *Escherichia coli* OX = 562 GN = cpdB PE = 3 SV = 1 | 1.257517359 | 0.015683823 | Up |
|  | D2SX86 | *fimC* | Type 1 fimbrial chaperone protein (Fragment) OS = *Escherichia coli* OX = 562 GN = fimC PE = 3 SV = 1 | 1.256046987 | 0.014427782 | Up |
|  | S0F2G7 | *adk* | Adenylate kinase (Fragment) OS = *Escherichia coli* OX = 562 GN = adk PE = 3 SV = 1 | 1.255496413 | 0.00107755 | Up |
|  | A0A6C9QTS1 | *eutM* | Ethanolamine utilization microcompartment protein EutM OS = *Escherichia coli* OX = 562 GN = eutM PE = 4 SV = 1 | 1.254678631 | 0.005375674 | Up |
|  | P28904 | *treC* | Trehalose-6-phosphate hydrolase OS = *Escherichia coli* (strain K12) OX = 83333 GN = treC PE = 1 SV = 3 | 1.254563628 | 0.001308639 | Up |
|  | A0A1X3KA87 | EATG_03963 | Mannitol operon repressor (Mannitol repressor protein) OS = *Escherichia coli* H605 OX = 656410 GN = EATG_03963 PE = 4 SV = 1 | 1.253696165 | 0.004552058 | Up |
|  | A0A827NKN5 | *glpC* | Anaerobic glycerol-3-phosphate dehydrogenase subunit C OS = *Escherichia coli* OX = 562 GN = glpC PE = 4 SV = 1 | 1.253005172 | 0.027119019 | Up |
|  | A0A827IXR6 | GIB53_25780 | IS1 family transposase OS = *Escherichia coli* OX = 562 GN = GIB53_25780 PE = 4 SV = 1 | 1.252696734 | 0.04881189 | Up |
|  | A0A376ZTY7 | *ispE* | 4-diphosphocytidyl-2-C-methyl-D-erythritol kinase OS = *Escherichia coli* OX = 562 GN = ispE PE = 3 SV = 1 | 1.252573957 | 0.006596745 | Up |
|  | A0A2J1D5L3 | *dnaK* | Molecular chaperone DnaK (Fragment) OS = *Escherichia coli* OX = 562 GN = dnaK PE = 4 SV = 1 | 1.252202133 | 0.029742715 | Up |
|  | A0A6M1J488 | G5696_15990 | Site-specific DNA-methyltransferase (adenine-specific) OS = *Escherichia coli* OX = 562 GN = G5696_15990 PE = 3 SV = 1 | 1.251826702 | 0.046544616 | Up |
|  | A0A6D0DAA5 | GQM13_02820 | Tail fiber assembly protein OS = *Escherichia coli* OX = 562 GN = GQM13_02820 PE = 4 SV = 1 | 1.250347136 | 0.006684943 | Up |
|  | D8ADF8 | *moaB* | Molybdenum cofactor biosynthesis protein B OS = *Escherichia coli* (strain MS 21-1) OX = 749527 GN = moaB PE = 3 SV = 1 | 1.248215703 | 0.000520957 | Up |
|  | A0A0K4GR52 | *hybD* | HyaD/HybD family hydrogenase maturation endopeptidase OS = *Escherichia coli* OX = 562 GN = hybD PE = 3 SV = 1 | 1.24751192 | 0.017816325 | Up |
|  | A0A827QMC8 | *metQ* | D-methionine-binding transport system MetQ OS = *Escherichia coli* OX = 562 GN = metQ PE = 4 SV = 1 | 1.246811908 | 0.003000952 | Up |
|  | A0A7H9QJD6 | *tusA* | Sulfur carrier protein TusA OS = *Escherichia coli* OX = 562 GN = tusA PE = 3 SV = 1 | 1.246650412 | 3.73173E-05 | Up |
|  | A0A826TAQ1 | *hdeB* | Acid-activated periplasmic chaperone HdeB OS = *Escherichia coli* OX = 562 GN = hdeB PE = 4 SV = 1 | 1.246493274 | 0.048228518 | Up |
|  | A0A5B9AWW1 | *yajQ* | UPF0234 protein YajQ OS = *Escherichia coli* OX = 562 GN = yajQ PE = 3 SV = 1 | 1.245195876 | 0.010650808 | Up |
|  | A0A2X1NZD1 | *yhbL_1* | Isoprenoid biosynthesis protein with amidotransferase-like domain OS = *Escherichia coli* OX = 562 GN = yhbL_1 PE = 4 SV = 1 | 1.244981097 | 0.00213436 | Up |
|  | A0A0K3UQX2 | ERS085366_02842 | Fels-1 Propage domain-containing protein OS = *Escherichia coli* OX = 562 GN = ERS085366_02842 PE = 4 SV = 1 | 1.244214484 | 0.047058577 | Up |
|  | A0A827HLW1 | *surE* | 5'/3'-nucleotidase SurE OS = *Escherichia coli* OX = 562 GN = surE PE = 4 SV = 1 | 1.244142827 | 0.000391866 | Up |
|  | F4T6A9 | ECIG_04883 | Sulfofructose kinase OS = *Escherichia coli* M605 OX = 656417 GN = ECIG_04883 PE = 3 SV = 1 | 1.243417671 | 0.022215636 | Up |
|  | A0A827K1F8 | *sslE* | Lipoprotein metalloprotease SslE (Fragment) OS = *Escherichia coli* OX = 562 GN = sslE PE = 4 SV = 1 | 1.243238771 | 0.011481726 | Up |
|  | A0A6N8Q332 | GRW77_20505 | 2',3'-cyclic-nucleotide 2'-phosphodiesterase/3'-nucleotidase OS = *Escherichia coli* OX = 562 GN = GRW77_20505 PE = 3 SV = 1 | 1.242079903 | 0.006628854 | Up |
|  | E6BR57 | *mtfA* | Protein MtfA OS = *Escherichia coli* MS 85-1 OX = 679202 GN = mtfA PE = 3 SV = 1 | 1.241631517 | 0.041057667 | Up |
|  | A0A6N8K5V0 | GQF59_28550 | DNA replication protein DnaC (Fragment) OS = *Escherichia coli* OX = 562 GN = GQF59_28550 PE = 4 SV = 1 | 1.240612065 | 0.00228295 | Up |
|  | A0A3L5HCC8 | *ompT* | Omptin family outer membrane protease OmpT OS = *Escherichia coli* OX = 562 GN = ompT PE = 3 SV = 1 | 1.240600393 | 0.038703969 | Up |
|  | A0A2T1LHY1 | *gltX* | Glutamate--tRNA ligase OS = *Escherichia coli* OX = 562 GN = gltX PE = 3 SV = 1 | 1.24048096 | 0.045461787 | Up |
|  | A0A7H9LRE0 | *comR* | TetR family copper-responsive transcriptional repressor ComR OS = *Escherichia coli* OX = 562 GN = comR PE = 4 SV = 1 | 1.240028669 | 0.003995944 | Up |
|  | A0A827TRU7 | F9571_25225 | Elongation factor Tu OS = *Escherichia coli* OX = 562 GN = F9571_25225 PE = 4 SV = 1 | 1.239892439 | 0.01109487 | Up |
|  | A0A0K4G9W2 | *greB* | Transcription elongation factor GreB OS = *Escherichia coli* OX = 562 GN = greB PE = 3 SV = 1 | 1.239771551 | 0.004898892 | Up |
|  | A0A826LFD7 | C5542_001192 | DeoR/GlpR transcriptional regulator OS = *Escherichia coli* OX = 562 GN = C5542_001192 PE = 4 SV = 1 | 1.239731113 | 0.00100182 | Up |
|  | A0A828UNV2 | *ptsN* | PTS IIA-like nitrogen-regulatory protein PtsN OS = *Escherichia coli* 3.2608 OX = 869679 GN = ptsN PE = 4 SV = 1 | 1.239538632 | 0.006342003 | Up |
|  | A0A417ZR54 | *asmA* | Outer membrane assembly protein AsmA OS = *Escherichia coli* OX = 562 GN = asmA PE = 4 SV = 1 | 1.239410656 | 0.005810221 | Up |
|  | A0A828KM58 | HCF72_003305 | Putative DNA-binding transcriptional regulator OS = *Escherichia coli* OX = 562 GN = HCF72_003305 PE = 4 SV = 1 | 1.238918675 | 0.007637479 | Up |
|  | A0A1X3KRB9 | *yceH* | UPF0502 protein YceH OS = *Escherichia coli* H605 OX = 656410 GN = yceH PE = 3 SV = 1 | 1.238583766 | 0.005608081 | Up |
|  | A0A376PXV3 | *ybjD* | ATP-dependent endonuclease OS = *Escherichia coli* OX = 562 GN = ybjD PE = 4 SV = 1 | 1.238440718 | 0.001792265 | Up |
|  | A0A6N6WNH5 | GP711_28040 | Porin OmpC (Fragment) OS = *Escherichia coli* OX = 562 GN = GP711_28040 PE = 4 SV = 1 | 1.238336132 | 0.024322734 | Up |
|  | A0A1X3KRU5 | *nudJ* | Phosphatase NudJ OS = *Escherichia coli* H605 OX = 656410 GN = nudJ PE = 3 SV = 1 | 1.236966188 | 0.001249922 | Up |
|  | A0A6L4XKW5 | *bamB* | Outer membrane protein assembly factor BamB OS = *Escherichia coli* OX = 562 GN = bamB PE = 3 SV = 1 | 1.235774549 | 0.021470285 | Up |
|  | A0A829DWE3 | *yciS* | Inner membrane protein yciS OS = *Escherichia coli* 2875150 OX = 1116036 GN = yciS PE = 4 SV = 1 | 1.235737251 | 8.99007E-05 | Up |
|  | A0A1V2GDA3 | BXT93_15460 | Uncharacterized protein OS = *Escherichia coli* OX = 562 GN = BXT93_15460 PE = 3 SV = 1 | 1.235486547 | 0.007465978 | Up |
|  | P08660 | *lysC* | Lysine-sensitive aspartokinase 3 OS = *Escherichia coli* (strain K12) OX = 83333 GN = lysC PE = 1 SV = 2 | 1.235325642 | 0.01902326 | Up |
|  | A0A6C8RUQ2 | *fucI* | L-fucose isomerase OS = *Escherichia coli* OX = 562 GN = fucI PE = 3 SV = 1 | 1.234761898 | 0.004083157 | Up |
|  | A0A0K4BWX3 | *macA_3* | UPF0194 membrane protein YbhG OS = *Escherichia coli* OX = 562 GN = macA_3 PE = 3 SV = 1 | 1.234127896 | 0.004596464 | Up |
|  | A0A6M0PUM0 | *ggt* | Glutathione hydrolase proenzyme OS = *Escherichia coli* OX = 562 GN = ggt PE = 3 SV = 1 | 1.231376249 | 0.023174144 | Up |
|  | A0A7T6C0A4 | *fucI* | L-fucose isomerase OS = *Escherichia coli* OX = 562 GN = fucI PE = 3 SV = 1 | 1.231306979 | 0.004853393 | Up |
|  | U9XLC8 | HMPREF1589_05305 | Formate dehydrogenase, gamma subunit OS = *Escherichia coli* 113290 OX = 1268976 GN = HMPREF1589_05305 PE = 3 SV = 1 | 1.230538596 | 0.045500042 | Up |
|  | A0A789MJ73 | *elbB* | Glyoxalase OS = *Escherichia coli* OX = 562 GN = elbB PE = 3 SV = 1 | 1.230532041 | 0.045983599 | Up |
|  | P0ABE2 | *bolA* | DNA-binding transcriptional regulator BolA OS = *Escherichia coli* (strain K12) OX = 83333 GN = bolA PE = 1 SV = 1 | 1.229366998 | 0.005845995 | Up |
|  | A0A2W6Q4G2 | DNQ45_07465 | Type I site-specific deoxyribonuclease OS = *Escherichia coli* OX = 562 GN = DNQ45_07465 PE = 3 SV = 1 | 1.229277004 | 0.010024024 | Up |
|  | A0A826LKU7 | C5542_001496 | HlyD family secretion protein OS = *Escherichia coli* OX = 562 GN = C5542_001496 PE = 4 SV = 1 | 1.228486893 | 0.036548355 | Up |
|  | A0A271QSQ8 | *katG* | Catalase-peroxidase OS = *Escherichia coli* OX = 562 GN = katG PE = 3 SV = 1 | 1.228168761 | 0.034433177 | Up |
|  | A0A829AE96 | A1Y7_02739 | Response regulator in two-component system withYehU OS = *Escherichia coli* KTE119 OX = 1182710 GN = A1Y7_02739 PE = 4 SV = 1 | 1.22785271 | 0.003449171 | Up |
|  | A0A6C9YER7 | E5S62_15810 | Putative sulfurtransferase YedF OS = *Escherichia coli* OX = 562 GN = E5S62_15810 PE = 3 SV = 1 | 1.226604883 | 0.039685493 | Up |
|  | A0A6D0IB78 | *mdtK* | Multidrug resistance protein MdtK (Fragment) OS = *Escherichia coli* OX = 562 GN = mdtK PE = 3 SV = 1 | 1.225318006 | 0.021847794 | Up |
|  | L3CC44 | A13W_00503 | Isomerase/hydrolase OS = *Escherichia coli* KTE193 OX = 1181739 GN = A13W_00503 PE = 4 SV = 1 | 1.223928203 | 0.006424743 | Up |
|  | A0A0A0H1H8 | *aes* | Acetyl esterase OS = *Escherichia coli* OX = 562 GN = aes PE = 3 SV = 1 | 1.223623893 | 0.005774656 | Up |
|  | A0A6D0IPV2 | *ribE* | Riboflavin synthase OS = *Escherichia coli* OX = 562 GN = ribE PE = 4 SV = 1 | 1.223374109 | 0.009293196 | Up |
|  | A0A827E234 | A2F99_003556 | YihD family protein OS = *Escherichia coli* OX = 562 GN = A2F99_003556 PE = 4 SV = 1 | 1.222552602 | 0.028535026 | Up |
|  | A0A2U2VVI3 | E2123_08350 | ABC transporter substrate-binding protein OS = *Escherichia coli* OX = 562 GN = E2123_08350 PE = 4 SV = 1 | 1.222054173 | 0.027617092 | Up |
|  | A0A2X7H6G4 | *fimD_5* | Outer membrane usher protein FimD OS = *Escherichia coli* OX = 562 GN = fimD_5 PE = 3 SV = 1 | 1.221945968 | 0.014021668 | Up |
|  | A0A8A8YI47 | *nagE* | PTS N-acetyl glucosamine transporter subunit IIABC OS = *Escherichia coli* OX = 562 GN = nagE PE = 4 SV = 1 | 1.220552203 | 0.00108211 | Up |
|  | A0A6C8TFT0 | *eutC* | Ethanolamine ammonia-lyase small subunit OS = *Escherichia coli* OX = 562 GN = eutC PE = 3 SV = 1 | 1.219895613 | 0.000914182 | Up |
|  | A0A376HWG1 | *aegA* | Oxidoreductase OS = *Escherichia coli* OX = 562 GN = aegA PE = 4 SV = 1 | 1.219679202 | 0.04149139 | Up |
|  | A0A376KS23 | *ftn* | Ferritin OS = *Escherichia coli* OX = 562 GN = ftn PE = 3 SV = 1 | 1.219674639 | 0.014003694 | Up |
|  | A0A2T1LPX8 | *ftsZ* | Cell division protein FtsZ OS = *Escherichia coli* OX = 562 GN = ftsZ PE = 3 SV = 1 | 1.218824025 | 0.049356453 | Up |
|  | E1IW65 | *allR* | HTH-type transcriptional repressor AllR OS = *Escherichia coli* MS 145-7 OX = 679204 GN = allR PE = 4 SV = 1 | 1.218432425 | 0.027578706 | Up |
|  | A0A8A5HUG8 | *uidA* | Beta-glucuronidase OS = *Escherichia coli* O126 : H45 OX = 2810407 GN = uidA PE = 4 SV = 1 | 1.218385086 | 0.012955854 | Up |
|  | A0A2T3TC58 | *ydcF* | YdcF family protein OS = *Escherichia coli* OX = 562 GN = ydcF PE = 4 SV = 1 | 1.218112143 | 0.001870945 | Up |
|  | A0A830VI41 | GF698_09710 | Amino-acid N-acetyltransferase OS = *Escherichia coli* OX = 562 GN = GF698_09710 PE = 4 SV = 1 | 1.217804428 | 0.002214206 | Up |
|  | A0A5F1DTT1 | *upp* | Uracil phosphoribosyltransferase OS = *Escherichia coli* OX = 562 GN = upp PE = 3 SV = 1 | 1.217138607 | 0.015228181 | Up |
|  | A0A366YRP2 | DS966_11260 | DUF937 domain-containing protein OS = *Escherichia coli* OX = 562 GN = DS966_11260 PE = 4 SV = 1 | 1.217136089 | 0.027564142 | Up |
|  | A0A2T1LLD6 | *purU* | Formyltetrahydrofolate deformylase OS = *Escherichia coli* OX = 562 GN = purU PE = 3 SV = 1 | 1.216953401 | 0.009714282 | Up |
|  | A0A826W9B9 | FC554_15080 | HPr family phosphocarrier protein OS = *Escherichia coli* OX = 562 GN = FC554_15080 PE = 4 SV = 1 | 1.216819268 | 0.001694812 | Up |
|  | A0A2X1NJ68 | *yeiQ* | Oxidoreductase yeiQ OS = *Escherichia coli* OX = 562 GN = yeiQ PE = 4 SV = 1 | 1.216761485 | 0.028463623 | Up |
|  | Q8X7U5 | *ybiP* | Putative enzyme OS = *Escherichia coli* O157 : H7 OX = 83334 GN = ybiP PE = 4 SV = 2 | 1.216405877 | 0.001573921 | Up |
|  | A0A831F693 | *allB* | Allantoinase AllB OS = *Escherichia coli* OX = 562 GN = allB PE = 4 SV = 1 | 1.216134951 | 0.01963718 | Up |
|  | B7UFB6 | *yegU* | Predicted hydrolase OS = *Escherichia coli* O127 : H6 (strain E2348/69 / EPEC) OX = 574521 GN = yegU PE = 4 SV = 1 | 1.215699121 | 0.00204171 | Up |
|  | A0A377KE75 | NCTC9075_06183 | Putative lipoprotein OS = *Escherichia coli* OX = 562 GN = NCTC9075_06183 PE = 4 SV = 1 | 1.215667271 | 0.002058524 | Up |
|  | A0A2X3KYC6 | *pflB2_2* | Formate acetyltransferase 1 OS = *Escherichia coli* OX = 562 GN = pflB2_2 PE = 4 SV = 1 | 1.215418262 | 0.018416388 | Up |
|  | A0A659GVV1 | *tnaA* | Tryptophanase OS = *Escherichia coli* OX = 562 GN = tnaA PE = 3 SV = 1 | 1.214610425 | 0.04173032 | Up |
|  | L4JCY5 | A311_01474 | Arginine transport ATP-binding protein ArtP OS = *Escherichia coli* KTE146 OX = 1182725 GN = A311_01474 PE = 3 SV = 1 | 1.213590056 | 0.003590042 | Up |
|  | F4SIU8 | ECHG_01354 | Toxin-antitoxin system, toxin component, HipA family OS = *Escherichia coli* H736 OX = 656414 GN = ECHG_01354 PE = 3 SV = 1 | 1.212039114 | 0.044634446 | Up |
|  | A0A0K4FIU4 | *murF* | UDP-N-acetylmuramoyl-tripeptide--D-alanyl-D-alanine ligase OS = *Escherichia coli* OX = 562 GN = murF PE = 3 SV = 1 | 1.211502483 | 0.047984358 | Up |
|  | A0A241QPT0 | *kdbD* | Histidine kinase OS = *Escherichia coli* OX = 562 GN = kdbD PE = 4 SV = 1 | 1.211270343 | 0.012855767 | Up |
|  | F4T7V4 | *frdD* | Fumarate reductase subunit D OS = *Escherichia coli* M605 OX = 656417 GN = frdD PE = 3 SV = 1 | 1.211251642 | 0.011963112 | Up |
|  | A0A376I3W0 | *bglX_1* | Periplasmic beta-glucosidase OS = *Escherichia coli* OX = 562 GN = bglX_1 PE = 3 SV = 1 | 1.211097686 | 0.021493739 | Up |
|  | B1XGY3 | *argG* | Argininosuccinate synthase OS = *Escherichia coli* (strain K12 / DH10B) OX = 316385 GN = argG PE = 3 SV = 1 | 1.210863208 | 0.007304289 | Up |
|  | A0A844EYQ6 | GKE84_23720 | Mobilization protein OS = *Escherichia coli* OX = 562 GN = GKE84_23720 PE = 4 SV = 1 | 1.208957227 | 0.014999487 | Up |
|  | A0A2Y0SKJ1 | *prlF* | Antitoxin PrlF OS = *Escherichia coli* OX = 562 GN = prlF PE = 4 SV = 1 | 1.206840891 | 0.00814453 | Up |
|  | A0A6N8R5F7 | GRW05_27270 | Arsenate reductase (Fragment) OS = *Escherichia coli* OX = 562 GN = GRW05_27270 PE = 4 SV = 1 | 1.206778428 | 0.027539277 | Up |
|  | A0A827KKT1 | AAS29_001167 | Polysaccharide deacetylase family protein OS = *Escherichia coli* OX = 562 GN = AAS29_001167 PE = 4 SV = 1 | 1.206109004 | 0.016632398 | Up |
|  | A0A7U2IP63 | *fucU* | L-fucose mutarotase OS = *Escherichia coli* OX = 562 GN = fucU PE = 3 SV = 1 | 1.20576367 | 0.010445268 | Up |
|  | A0A777CQL7 | *pdxK* | Pyridoxine/pyridoxal/pyridoxamine kinase OS = *Escherichia coli* OX = 562 GN = pdxK PE = 3 SV = 1 | 1.205554527 | 0.007972672 | Up |
|  | A7ZV11 | *groES* | Co-chaperonin GroES OS = *Escherichia coli* O139 : H28 (strain E24377A / ETEC) OX = 331111 GN = groES PE = 3 SV = 1 | 1.205411387 | 0.022661696 | Up |
|  | A0A6D0EJA7 | GQM09_23615 | Glutamine--fructose-6-phosphate aminotransferase [isomerizing] (Fragment) OS = *Escherichia coli* OX = 562 GN = GQM09_23615 PE = 4 SV = 1 | 1.204837544 | 0.007762505 | Up |
|  | A0A792T099 | *glpA* | Glycerol-3-phosphate dehydrogenase OS = *Escherichia coli* OX = 562 GN = glpA PE = 3 SV = 1 | 1.204467756 | 0.009482899 | Up |
|  | A0A418GJM4 | D3C88_15285 | Anaerobic ribonucleoside-triphosphate reductase (Fragment) OS = *Escherichia coli* OX = 562 GN = D3C88_15285 PE = 4 SV = 1 | 1.204428928 | 0.008914115 | Up |
|  | A0A7I6H1X7 | *psuG* | Pseudouridine-5'-phosphate glycosidase OS = *Escherichia coli* O9 : H4 (strain HS) OX = 331112 GN = psuG PE = 3 SV = 1 | 1.203987331 | 0.044790831 | Up |
|  | A0A827KNR7 | *eutL* | Ethanolamine utilization microcompartment protein EutL OS = *Escherichia coli* OX = 562 GN = eutL PE = 4 SV = 1 | 1.203612927 | 0.000173533 | Up |
|  | A0A6G4BN73 | *purT* | Formate-dependent phosphoribosylglycinamide formyltransferase OS = *Escherichia coli* OX = 562 GN = purT PE = 3 SV = 1 | 1.203607142 | 0.008895069 | Up |
|  | A0A793U2L0 | *mrr* | Methylated adenine and cytosine restriction protein OS = *Escherichia coli* OX = 562 GN = mrr PE = 4 SV = 1 | 1.203414222 | 0.009386633 | Up |
|  | A0A6D0F9L4 | *uxuR* | Uxu operon transcriptional regulator OS = *Escherichia coli* OX = 562 GN = uxuR PE = 4 SV = 1 | 1.203224431 | 0.000311689 | Up |
|  | A0A838AV65 | H0O53_20540 | ABC transporter substrate-binding protein OS = *Escherichia coli* OX = 562 GN = H0O53_20540 PE = 4 SV = 1 | 1.203154008 | 0.016973038 | Up |
|  | A0A8B3HZ19 | EIA13_03215 | Cyclopropane fatty acyl phospholipid synthase OS = *Escherichia coli* OX = 562 GN = EIA13_03215 PE = 4 SV = 1 | 1.202902115 | 0.020089126 | Up |
|  | A0A485JCI0 | *glpQ* | Glycerophosphodiester phosphodiesterase OS = *Escherichia coli* OX = 562 GN = glpQ PE = 4 SV = 1 | 1.201088942 | 0.003100554 | Up |
|  | A0A7H9LQR7 | HV146_07990 | DUF535 domain-containing protein OS = *Escherichia coli* OX = 562 GN = HV146_07990 PE = 4 SV = 1 | 1.20056136 | 0.024146356 | Up |
|  | A0A376RIF4 | *gst* | Glutathione S-transferase OS = *Escherichia coli* OX = 562 GN = gst PE = 4 SV = 1 | 1.200107272 | 0.002950533 | Up |
|  | A0A5F1T0A9 | *maeA* | NAD-dependent malic enzyme OS = *Escherichia coli* OX = 562 GN = maeA PE = 3 SV = 1 | 0.833244778 | 0.00950875 | Down |
|  | A0A377K5F9 | *flhE* | Flagellar protein FlhE OS = *Escherichia coli* OX = 562 GN = flhE PE = 4 SV = 1 | 0.832495663 | 0.016158276 | Down |
|  | A0A826X5I1 | *uvrA* | Excinuclease ABC subunit UvrA OS = *Escherichia coli* OX = 562 GN = uvrA PE = 4 SV = 1 | 0.831566732 | 0.001030504 | Down |
|  | C6UY58 | *cheZ* | Protein phosphatase CheZ OS = *Escherichia coli* O157 : H7 (strain TW14359 / EHEC) OX = 544404 GN = cheZ PE = 3 SV = 1 | 0.831467732 | 0.012851281 | Down |
|  | A0A0L1C331 | *aphA* | Class B acid phosphatase OS = *Escherichia coli* OX = 562 GN = aphA PE = 3 SV = 1 | 0.830886478 | 0.001568434 | Down |
|  | D6IFZ9 | ECDG_04381 | HTH-type transcriptional regulator yieP OS = *Escherichia coli* B185 OX = 550676 GN = ECDG_04381 PE = 4 SV = 1 | 0.830881749 | 0.021948145 | Down |
|  | B1XGK1 | *nadE* | NH(3)-dependent NAD(+) synthetase OS = *Escherichia coli* (strain K12 / DH10B) OX = 316385 GN = nadE PE = 3 SV = 1 | 0.830708327 | 0.008421745 | Down |
|  | S1ECA0 | A1UI_03248 | DUF927 domain-containing protein OS = *Escherichia coli* KTE73 OX = 1182680 GN = A1UI_03248 PE = 4 SV = 1 | 0.830633407 | 0.003584886 | Down |
|  | A0A854WA48 | *glgC* | Glucose-1-phosphate adenylyltransferase OS = *Escherichia coli* OX = 562 GN = glgC PE = 4 SV = 1 | 0.830391155 | 0.008809631 | Down |
|  | A0A773MCP9 | *yegD* | Molecular chaperone OS = *Escherichia coli* OX = 562 GN = yegD PE = 3 SV = 1 | 0.830388636 | 0.04351979 | Down |
|  | A0A826WQ01 | *uspE* | Universal stress protein UspE OS = *Escherichia coli* OX = 562 GN = uspE PE = 4 SV = 1 | 0.830151374 | 0.00070737 | Down |
|  | S4VDV0 | *thrB* | Homoserine kinase OS = *Escherichia coli* OX = 562 GN = thrB PE = 3 SV = 1 | 0.829941358 | 0.01333352 | Down |
|  | A0A376U3J7 | *yjiY* | Putative carbon starvation protein OS = *Escherichia coli* OX = 562 GN = yjiY PE = 3 SV = 1 | 0.829817593 | 0.04599746 | Down |
|  | B7UGT9 | E2348C_2776 | RecT family protein OS = *Escherichia coli* O127 : H6 (strain E2348/69 / EPEC) OX = 574521 GN = E2348C_2776 PE = 4 SV = 1 | 0.829741484 | 0.002462614 | Down |
|  | A0A376U8C7 | *cmoA* | Protein YecO OS = *Escherichia coli* OX = 562 GN = cmoA PE = 4 SV = 1 | 0.829476286 | 0.021609627 | Down |
|  | A0A7Z8HVR4 | *nifJ* | Pyruvate : ferredoxin (Flavodoxin) oxidoreductase OS = *Escherichia coli* OX = 562 GN = nifJ PE = 3 SV = 1 | 0.829393164 | 0.029878201 | Down |
|  | P0ADU3 | *ygiN* | Probable quinol monooxygenase YgiN OS = *Escherichia coli* O157 : H7 OX = 83334 GN = ygiN PE = 3 SV = 1 | 0.828909012 | 0.004139666 | Down |
|  | A0A6D0G0H1 | GP711_16415 | Flagellar hook protein FlgE OS = *Escherichia coli* OX = 562 GN = GP711_16415 PE = 3 SV = 1 | 0.82883034 | 0.048706559 | Down |
|  | A0A2X1J7U1 | *ppiC* | Peptidyl-prolyl cis-trans isomerase C OS = *Escherichia coli* OX = 562 GN = ppiC PE = 4 SV = 1 | 0.828637034 | 0.022480065 | Down |
|  | A0A6D0LUI0 | GRW80_21050 | Tyrosine-type recombinase/integrase OS = *Escherichia coli* OX = 562 GN = GRW80_21050 PE = 4 SV = 1 | 0.828463393 | 0.043823877 | Down |
|  | A0A7L5L2P8 | *rpsP* | 30S ribosomal protein S16 OS = *Escherichia coli* OX = 562 GN = rpsP PE = 3 SV = 1 | 0.828244521 | 0.004775401 | Down |
|  | V0SFH5 | *clpP* | ATP-dependent Clp protease proteolytic subunit OS = *Escherichia coli* 907672 OX = 1268982 GN = clpP PE = 3 SV = 1 | 0.828059136 | 0.000109397 | Down |
|  | A0A826XAI7 | *aphA* | Acid phosphatase AphA OS = *Escherichia coli* OX = 562 GN = aphA PE = 4 SV = 1 | 0.8279924 | 0.034927769 | Down |
|  | A0A376Y3C3 | *ygfB* | UPF0149 protein YgfB OS = *Escherichia coli* OX = 562 GN = ygfB PE = 3 SV = 1 | 0.827943017 | 0.010375784 | Down |
|  | A0A2T1LLY9 | C6985_05005 | Phosphoporin PhoE OS = *Escherichia coli* OX = 562 GN = C6985_05005 PE = 3 SV = 1 | 0.826530371 | 0.038138228 | Down |
|  | A0A1X3LVF6 | EAZG_00618 | Galactose-1-phosphate uridylyltransferase OS = *Escherichia coli* TA249 OX = 656441 GN = EAZG_00618 PE = 3 SV = 1 | 0.826433183 | 0.002896609 | Down |
|  | A0A210BJJ3 | A8C65_07375 | Formate C-acetyltransferase OS = *Escherichia coli* OX = 562 GN = A8C65_07375 PE = 4 SV = 1 | 0.826289203 | 0.04969988 | Down |
|  | A0A828HF64 | *gatB* | PTS galactitol transporter subunit IIB OS = *Escherichia coli* OX = 562 GN = gatB PE = 4 SV = 1 | 0.825842492 | 0.000249764 | Down |
|  | B1LDE8 | *speA* | Biosynthetic arginine decarboxylase OS = *Escherichia coli* (strain SMS-3-5 / SECEC) OX = 439855 GN = speA PE = 3 SV = 1 | 0.825594552 | 0.000147716 | Down |
|  | A0A246NR53 | *cysJ* | Sulfite reductase [NADPH] flavoprotein alpha-component OS = *Escherichia coli* OX = 562 GN = cysJ PE = 3 SV = 1 | 0.824983898 | 0.0139376 | Down |
|  | A0A4D9Z8J3 | ELX70_02700 | LysR family transcriptional regulator OS = *Escherichia coli* OX = 562 GN = ELX70_02700 PE = 3 SV = 1 | 0.824538905 | 0.006575827 | Down |
|  | A0A6L4XHG6 | GP710_13335 | Efflux pump membrane transporter OS = *Escherichia coli* OX = 562 GN = GP710_13335 PE = 3 SV = 1 | 0.824327334 | 0.017096847 | Down |
|  | A0A447Y522 | *glnA_1* | Glutamine synthetase OS = *Escherichia coli* OX = 562 GN = glnA_1 PE = 3 SV = 1 | 0.824036596 | 0.022856607 | Down |
|  | A0A5N3D643 | *dld* | Quinone-dependent D-lactate dehydrogenase OS = *Escherichia coli* OX = 562 GN = dld PE = 3 SV = 1 | 0.823794647 | 0.008321069 | Down |
|  | A0A6M0PRB2 | *rssB* | Regulator of RpoS OS = *Escherichia coli* OX = 562 GN = rssB PE = 3 SV = 1 | 0.823648548 | 0.016267821 | Down |
|  | P77475 | *yqaB* | Fructose-1-phosphate phosphatase YqaB OS = *Escherichia coli* (strain K12) OX = 83333 GN = yqaB PE = 1 SV = 1 | 0.823179382 | 0.015289795 | Down |
|  | A0A2T1LE54 | *rpsL* | 30S ribosomal protein S12 OS = *Escherichia coli* OX = 562 GN = rpsL PE = 3 SV = 1 | 0.822681903 | 0.00244075 | Down |
|  | A0A6N8PRT9 | GRW77_04245 | DNA translocase FtsK OS = *Escherichia coli* OX = 562 GN = GRW77_04245 PE = 3 SV = 1 | 0.822537689 | 0.009934741 | Down |
|  | F4SNW0 | *zapE* | Cell division protein ZapE OS = *Escherichia coli* H736 OX = 656414 GN = zapE PE = 3 SV = 1 | 0.822415204 | 0.002791676 | Down |
|  | A0A5R8TDT4 | EWT59_07740 | 30S ribosomal protein S12 methylthiotransferase accessory protein YcaO OS = *Escherichia coli* O25b : H4 OX = 941280 GN = EWT59_07740 PE = 4 SV = 1 | 0.821997744 | 0.000366623 | Down |
|  | A0A6L4XIH0 | GP710_11515 | EAL domain-containing protein OS = *Escherichia coli* OX = 562 GN = GP710_11515 PE = 4 SV = 1 | 0.821856164 | 0.005911274 | Down |
|  | A0A838AWA8 | *lpxB* | Lipid-A-disaccharide synthase OS = *Escherichia coli* OX = 562 GN = lpxB PE = 4 SV = 1 | 0.821843033 | 0.00488499 | Down |
|  | A0A376PPP7 | *tdcG* | L-serine dehydratase OS = *Escherichia coli* OX = 562 GN = tdcG PE = 3 SV = 1 | 0.82103875 | 0.00941755 | Down |
|  | A0A826NJ86 | *infB* | Translation initiation factor IF-2 OS = *Escherichia coli* O157 OX = 1045010 GN = infB PE = 4 SV = 1 | 0.819729251 | 0.000367611 | Down |
|  | A0A3W4GGJ5 | *mreC* | Cell shape protein MreC OS = *Escherichia coli* OX = 562 GN = mreC PE = 3 SV = 1 | 0.819638999 | 0.022148223 | Down |
|  | A0A828LQQ9 | *modF* | Molybdate ABC transporter ATP-binding protein ModF OS = *Escherichia coli* OX = 562 GN = modF PE = 4 SV = 1 | 0.818215963 | 0.002794917 | Down |
|  | A0A826KAA6 | *lolA* | Outer membrane lipoprotein chaperone LolA OS = *Escherichia coli* OX = 562 GN = lolA PE = 4 SV = 1 | 0.817497246 | 0.001019923 | Down |
|  | A0A6N8Q549 | GRW77_32280 | Tagatose-bisphosphate aldolase subunit GatZ (Fragment) OS = *Escherichia coli* OX = 562 GN = GRW77_32280 PE = 4 SV = 1 | 0.817111979 | 0.005349367 | Down |
|  | A0A4C4K7Q3 | *fliC* | Flagellin OS = *Escherichia coli* OX = 562 GN = fliC PE = 3 SV = 1 | 0.816932036 | 0.009948958 | Down |
|  | A0A2Y8JQV3 | *ycjG* | Dipeptide epimerase OS = *Escherichia coli* OX = 562 GN = ycjG PE = 3 SV = 1 | 0.816763875 | 0.003885551 | Down |
|  | A0A826RU97 | FPS82_07065 | Aldo/keto reductase family oxidoreductase OS = *Escherichia coli* OX = 562 GN = FPS82_07065 PE = 4 SV = 1 | 0.816398448 | 0.001376658 | Down |
|  | A0A1M2EKK4 | BK375_23085 | DrsE domain-containing protein OS = *Escherichia coli* OX = 562 GN = BK375_23085 PE = 4 SV = 1 | 0.816012273 | 0.007019491 | Down |
|  | A0A827BBU1 | *gatZ* | Tagatose-bisphosphate aldolase subunit GatZ OS = *Escherichia coli* OX = 562 GN = gatZ PE = 4 SV = 1 | 0.815479354 | 0.000684879 | Down |
|  | A0A770C125 | *malK* | Maltose/maltodextrin import ATP-binding protein MalK OS = *Escherichia coli* OX = 562 GN = malK PE = 4 SV = 1 | 0.815187806 | 0.040839951 | Down |
|  | B1X6D5 | *tsaC* | Threonylcarbamoyl-AMP synthase OS = *Escherichia coli* (strain K12 / DH10B) OX = 316385 GN = tsaC PE = 3 SV = 1 | 0.815111295 | 0.003236429 | Down |
|  | A0A6A0Q765 | *malK* | Maltose/maltodextrin ABC transporter ATP-binding protein MalK OS = *Escherichia coli* OX = 562 GN = malK PE = 4 SV = 1 | 0.815003897 | 0.045399839 | Down |
|  | A0A0A0H5V9 | *fliP* | Flagellar biosynthetic protein FliP OS = *Escherichia coli* OX = 562 GN = fliP PE = 3 SV = 1 | 0.814573798 | 0.001034689 | Down |
|  | A0A029HTA9 | *rcsB* | Capsular synthesis regulator component B OS = *Escherichia coli* 2-005-03_S4_C3 OX = 1444258 GN = rcsB PE = 4 SV = 1 | 0.814541662 | 0.001202531 | Down |
|  | A0A5B9AB60 | *priB* | Primosomal replication protein N OS = *Escherichia coli* OX = 562 GN = priB PE = 3 SV = 1 | 0.813903286 | 0.01171404 | Down |
|  | A0A7H9LPC0 | HV146_05120 | LysR family transcriptional regulator OS = *Escherichia coli* OX = 562 GN = HV146_05120 PE = 3 SV = 1 | 0.812869113 | 0.007889384 | Down |
|  | A0A6D0JE36 | GP946_28495 | ATP-binding cassette domain-containing protein (Fragment) OS = *Escherichia coli* OX = 562 GN = GP946_28495 PE = 4 SV = 1 | 0.812785347 | 0.039008698 | Down |
|  | A0A844EXI2 | GKE84_15090 | AAA family ATPase (Fragment) OS = *Escherichia coli* OX = 562 GN = GKE84_15090 PE = 4 SV = 1 | 0.812526875 | 0.007027247 | Down |
|  | A0A7B9FK36 | *ebgR* | Transcriptional regulator EbgR OS = *Escherichia coli* OX = 562 GN = ebgR PE = 4 SV = 1 | 0.81250669 | 0.00192441 | Down |
|  | F4TAV4 | *lon* | Lon protease OS = *Escherichia coli* M718 OX = 656419 GN = lon PE = 2 SV = 1 | 0.812407028 | 0.011355499 | Down |
|  | A0A6L4XLX1 | GP710_05650 | Lipopolysaccharide heptosyltransferase family protein OS = *Escherichia coli* OX = 562 GN = GP710_05650 PE = 4 SV = 1 | 0.812380071 | 0.012082109 | Down |
|  | A0A376I0H2 | *pta* | Phosphate acetyltransferase OS = *Escherichia coli* OX = 562 GN = pta PE = 3 SV = 1 | 0.811486162 | 0.004851251 | Down |
|  | C3SRM0 | ECs4127 | Biotin carboxyl carrier protein of acetyl-CoA carboxylase OS = *Escherichia coli* OX = 562 GN = ECs4127 PE = 4 SV = 1 | 0.810970172 | 0.006112399 | Down |
|  | A0A3Q0N3B6 | CR539_22890 | Chromosome segregation protein SMC OS = *Escherichia coli* OX = 562 GN = CR539_22890 PE = 4 SV = 1 | 0.810958551 | 0.044941527 | Down |
|  | A0A6L4XNK1 | GP710_04895 | Sugar-phosphatase OS = *Escherichia coli* OX = 562 GN = GP710_04895 PE = 4 SV = 1 | 0.810545736 | 0.019517845 | Down |
|  | A0A3T9CJN2 | *nusA* | Transcription termination/antitermination protein NusA OS = *Escherichia coli* OX = 562 GN = nusA PE = 3 SV = 1 | 0.809665605 | 7.80117E-05 | Down |
|  | A0A831B9J4 | HJS37_002968 | TIGR01620 family protein OS = *Escherichia coli* OX = 562 GN = HJS37_002968 PE = 4 SV = 1 | 0.809331952 | 0.000875982 | Down |
|  | A0A2X7I5K6 | *yigZ* | Elongation factor OS = *Escherichia coli* OX = 562 GN = yigZ PE = 3 SV = 1 | 0.809270691 | 0.006354291 | Down |
|  | V0YFL6 | *rpmC* | 50S ribosomal protein L29 OS = *Escherichia coli* 908525 OX = 1268995 GN = rpmC PE = 3 SV = 1 | 0.809059401 | 0.047356241 | Down |
|  | I2STI4 | *phsB* | Thiosulfate reductase electron transport protein phsb OS = *Escherichia coli* 1.2264 OX = 869675 GN = phsB PE = 4 SV = 1 | 0.80901082 | 0.003163349 | Down |
|  | A0A377D334 | *trpC* | Multifunctional fusion protein OS = *Escherichia coli* OX = 562 GN = trpC PE = 3 SV = 1 | 0.808888357 | 0.003637226 | Down |
|  | A0A827RWR5 | *aceE* | Pyruvate dehydrogenase (Acetyl-transferring), homodimeric type OS = *Escherichia coli* OX = 562 GN = aceE PE = 4 SV = 1 | 0.808374899 | 0.043617754 | Down |
|  | A0A6C9XEF8 | GP906_06025 | YjjI family glycine radical enzyme OS = *Escherichia coli* OX = 562 GN = GP906_06025 PE = 4 SV = 1 | 0.807752507 | 0.032859064 | Down |
|  | A0A1X9THD8 | CA593_09065 | Alpha-1,4 glucan phosphorylase OS = *Escherichia coli* OX = 562 GN = CA593_09065 PE = 3 SV = 1 | 0.80737852 | 0.003747104 | Down |
|  | A0A0K4MBS7 | *trmJ* | tRNA (cytidine/uridine-2'-O-)-methyltransferase TrmJ OS = *Escherichia coli* OX = 562 GN = trmJ PE = 3 SV = 1 | 0.807259548 | 0.00858634 | Down |
|  | B6HZL7 | *nusB* | Transcription antitermination protein NusB OS = *Escherichia coli* (strain SE11) OX = 409438 GN = nusB PE = 3 SV = 1 | 0.807170431 | 0.008174045 | Down |
|  | D3QWK1 | *gmhB* | D,D-heptose 1,7-bisphosphate phosphatase OS = *Escherichia coli* O55 : H7 (strain CB9615 / EPEC) OX = 701177 GN = gmhB PE = 3 SV = 1 | 0.806720316 | 0.023366451 | Down |
|  | A0A2H4TNY6 | *nagB* | Glucosamine-6-phosphate deaminase OS = *Escherichia coli* OX = 562 GN = nagB PE = 3 SV = 1 | 0.806543765 | 0.014886477 | Down |
|  | A0A368IPW2 | *yidA* | Sugar phosphatase YidA OS = *Escherichia coli* OX = 562 GN = yidA PE = 4 SV = 1 | 0.80652238 | 0.004576251 | Down |
|  | A0A7U0H0D9 | *topA* | DNA topoisomerase 1 OS = *Escherichia coli* OX = 562 GN = topA PE = 3 SV = 1 | 0.805914104 | 0.0034276 | Down |
|  | A0A2I6IHS3 | BVL39_24760 | Phospho-2-dehydro-3-deoxyheptonate aldolase OS = *Escherichia coli* OX = 562 GN = BVL39_24760 PE = 3 SV = 1 | 0.805004944 | 5.36773E-05 | Down |
|  | A0A5D8SK84 | *alsE* | D-allulose-6-phosphate 3-epimerase OS = *Escherichia coli* OX = 562 GN = alsE PE = 3 SV = 1 | 0.80456987 | 0.023349347 | Down |
|  | A0A6L4XR91 | *aroG* | Phospho-2-dehydro-3-deoxyheptonate aldolase OS = *Escherichia coli* OX = 562 GN = aroG PE = 3 SV = 1 | 0.804545427 | 0.002900539 | Down |
|  | A0A3L0VYM4 | D9F05_06435 | AMP nucleosidase OS = *Escherichia coli* OX = 562 GN = D9F05_06435 PE = 4 SV = 1 | 0.804122854 | 0.008920009 | Down |
|  | A0A790HG40 | BO068_003177 | YncE family protein OS = *Escherichia coli* OX = 562 GN = BO068_003177 PE = 4 SV = 1 | 0.802669547 | 0.022413255 | Down |
|  | A0A3L0VT52 | *lon* | Lon protease OS = *Escherichia coli* OX = 562 GN = lon PE = 2 SV = 1 | 0.802291131 | 0.016863586 | Down |
|  | A0A0K5M4M1 | *pmbA* | Metalloprotease PmbA OS = *Escherichia coli* OX = 562 GN = pmbA PE = 3 SV = 1 | 0.802272918 | 0.023651456 | Down |
|  | P39384 | *yjiM* | Putative dehydratase subunit YjiM OS = *Escherichia coli* (strain K12) OX = 83333 GN = yjiM PE = 3 SV = 2 | 0.802252835 | 0.019423666 | Down |
|  | A0A377HF78 | *eae* | Putative adhesin OS = *Escherichia coli* OX = 562 GN = eae PE = 3 SV = 1 | 0.801566769 | 0.020427439 | Down |
|  | A0A193LMB8 | GJ11_00930 | Chaperone protein Skp OS = *Escherichia coli* OX = 562 GN = GJ11_00930 PE = 3 SV = 1 | 0.800096737 | 0.003066617 | Down |
|  | D3GY01 | EC042_4857 | Putative glycoprotein/receptor OS = *Escherichia coli* O44 : H18 (strain 042 / EAEC) OX = 216592 GN = EC042_4857 PE = 4 SV = 1 | 0.799703064 | 0.000246013 | Down |
|  | A0A2T1LHJ7 | *iscA* | Iron-binding protein IscA OS = *Escherichia coli* OX = 562 GN = iscA PE = 3 SV = 1 | 0.799621774 | 0.002321857 | Down |
|  | A0A0K4GWR9 | *speA* | Biosynthetic arginine decarboxylase OS = *Escherichia coli* OX = 562 GN = speA PE = 3 SV = 1 | 0.799014146 | 0.003617678 | Down |
|  | A0A6N8Q4X1 | GRW77_29845 | Tagatose-bisphosphate aldolase (Fragment) OS = *Escherichia coli* OX = 562 GN = GRW77_29845 PE = 4 SV = 1 | 0.798609831 | 0.001911374 | Down |
|  | B1IY62 | *mqo* | Probable malate : quinone oxidoreductase OS = *Escherichia coli* (strain ATCC 8739 / DSM 1576 / NBRC 3972 / NCIMB 8545 / WDCM 00012 / Crooks) OX = 481805 GN = mqo PE = 3 SV = 1 | 0.798477728 | 0.037537908 | Down |
|  | A0A271QU37 | *yncE* | Putative receptor OS = *Escherichia coli* OX = 562 GN = yncE PE = 4 SV = 1 | 0.797827837 | 0.004410201 | Down |
|  | A0A2H9EVR4 | *aceE* | Pyruvate dehydrogenase E1 component OS = *Escherichia coli* OX = 562 GN = aceE PE = 4 SV = 1 | 0.797134443 | 0.001498922 | Down |
|  | A0A3Q0MWV8 | *flgE* | Flagellar hook protein FlgE OS = *Escherichia coli* OX = 562 GN = flgE PE = 3 SV = 1 | 0.79684677 | 0.005303501 | Down |
|  | A0A827VIM6 | *yeiB* | DUF418 domain-containing protein OS = *Escherichia coli* OX = 562 GN = yeiB PE = 4 SV = 1 | 0.796235619 | 0.009057132 | Down |
|  | P04949 | *fliC* | Flagellin OS = *Escherichia coli* (strain K12) OX = 83333 GN = fliC PE = 1 SV = 2 | 0.796127056 | 0.045691276 | Down |
|  | A0A829JPY3 | H000_05313 | Transposase OS = *Escherichia coli* UMEA 3899-1 OX = 1281275 GN = H000_05313 PE = 4 SV = 1 | 0.795834782 | 0.029100007 | Down |
|  | A0A6M1HYJ4 | *acrA* | Multidrug efflux RND transporter periplasmic adaptor subunit AcrA (Fragment) OS = *Escherichia coli* OX = 562 GN = acrA PE = 3 SV = 1 | 0.79540181 | 0.025060138 | Down |
|  | P76046 | *ycjX* | Uncharacterized protein YcjX OS = *Escherichia coli* (strain K12) OX = 83333 GN = ycjX PE = 1 SV = 1 | 0.794082427 | 0.001530991 | Down |
|  | A0A826URB7 | *exbB* | Tol-pal system-associated acyl-CoA thioesterase OS = *Escherichia coli* OX = 562 GN = exbB PE = 4 SV = 1 | 0.793974091 | 0.001403102 | Down |
|  | B3SGP3 | *flkA* | Flagellin OS = *Escherichia coli* OX = 562 GN = flkA PE = 3 SV = 1 | 0.793873856 | 0.02412548 | Down |
|  | A0A376I4P1 | *gltP_2* | Proton/glutamate-aspartate symporter OS = *Escherichia coli* OX = 562 GN = gltP_2 PE = 3 SV = 1 | 0.793137932 | 0.000840814 | Down |
|  | A0A2J1DEI0 | *malP* | Alpha-1,4 glucan phosphorylase OS = *Escherichia coli* OX = 562 GN = malP PE = 3 SV = 1 | 0.792332598 | 0.029168768 | Down |
|  | P0ACM9 | *yihL* | Uncharacterized HTH-type transcriptional regulator YihL OS = *Escherichia coli* (strain K12) OX = 83333 GN = yihL PE = 4 SV = 1 | 0.792289274 | 0.026670984 | Down |
|  | A0A7L7E9P5 | *pepN* | Aminopeptidase N OS = *Escherichia coli* OX = 562 GN = pepN PE = 3 SV = 1 | 0.790897061 | 0.013856894 | Down |
|  | P0AEH3 | *elaA* | Protein ElaA OS = *Escherichia coli* (strain K12) OX = 83333 GN = elaA PE = 1 SV = 1 | 0.790889391 | 0.000760455 | Down |
|  | A0A1M0D457 | *ycbX* | 6-N-hydroxylaminopurine resistance protein YcbX OS = *Escherichia coli* OX = 562 GN = ycbX PE = 4 SV = 1 | 0.790078677 | 0.003812731 | Down |
|  | A0A377CPF6 | *rhlB* | ATP-dependent RNA helicase RhlB OS = *Escherichia coli* OX = 562 GN = rhlB PE = 3 SV = 1 | 0.789431879 | 0.000282288 | Down |
|  | F4SMG2 | ECHG_00753 | Protein YccU OS = *Escherichia coli* H736 OX = 656414 GN = ECHG_00753 PE = 4 SV = 1 | 0.788952419 | 0.002889762 | Down |
|  | A0A193LVC4 | *iscS* | Cysteine desulfurase IscS OS = *Escherichia coli* OX = 562 GN = iscS PE = 3 SV = 1 | 0.788645438 | 0.002044116 | Down |
|  | Q8XAR1 | *wecE* | dTDP-4-amino-4,6-dideoxygalactose transaminase OS = *Escherichia coli* O157 : H7 OX = 83334 GN = wecE PE = 3 SV = 2 | 0.788153857 | 0.018023207 | Down |
|  | A0A828A1X0 | *evgA* | Acid-sensing system DNA-binding response regulator EvgA OS = *Escherichia coli* OX = 562 GN = evgA PE = 4 SV = 1 | 0.787997838 | 0.001561284 | Down |
|  | A0A4P8BZI1 | CCU01_012670 | Methionine-R-sulfoxide reductase OS = *Escherichia coli* O145 : NM OX = 991919 GN = CCU01_012670 PE = 4 SV = 1 | 0.787792603 | 0.001426403 | Down |
|  | A0A0E1T1H7 | *caiB* | L-carnitine CoA-transferase OS = *Escherichia coli* 53638 OX = 344610 GN = caiB PE = 3 SV = 1 | 0.787328949 | 0.009373029 | Down |
|  | P29744 | *flgL* | Flagellar hook-associated protein 3 OS = *Escherichia coli* (strain K12) OX = 83333 GN = flgL PE = 3 SV = 2 | 0.787267149 | 0.03266794 | Down |
|  | A0A829DGU5 | EC2875150_2933 | Haloacid dehalogenase-like hydrolase family protein OS = *Escherichia coli* 2875150 OX = 1116036 GN = EC2875150_2933 PE = 4 SV = 1 | 0.786509056 | 0.011420028 | Down |
|  | E6BD92 | HMPREF9350_00052 | Phospho-2-dehydro-3-deoxyheptonate aldolase OS = *Escherichia coli* MS 85-1 OX = 679202 GN = HMPREF9350_00052 PE = 3 SV = 1 | 0.786459001 | 0.004838667 | Down |
|  | A0A6N8PSS5 | *nadC* | Quinolinate phosphoribosyltransferase [decarboxylating] OS = *Escherichia coli* OX = 562 GN = nadC PE = 3 SV = 1 | 0.786273326 | 0.018362138 | Down |
|  | I2X034 | EC40967_1679 | Oxidoreductase, NAD-binding domain protein OS = *Escherichia coli* 4.0967 OX = 869687 GN = EC40967_1679 PE = 4 SV = 1 | 0.785244232 | 0.000387017 | Down |
|  | P24203 | *yjiA* | P-loop guanosine triphosphatase YjiA OS = *Escherichia coli* (strain K12) OX = 83333 GN = yjiA PE = 1 SV = 3 | 0.785154838 | 0.018199175 | Down |
|  | A0A828B9X1 | *nudK* | GDP-mannose pyrophosphatase NudK OS = *Escherichia coli* OX = 562 GN = nudK PE = 4 SV = 1 | 0.785119065 | 0.000220267 | Down |
|  | P64476 | *ydiH* | Uncharacterized protein YdiH OS = *Escherichia coli* (strain K12) OX = 83333 GN = ydiH PE = 4 SV = 2 | 0.784984184 | 0.002745137 | Down |
|  | A0A6M0PZZ0 | *rhlB* | ATP-dependent RNA helicase RhlB OS = *Escherichia coli* OX = 562 GN = rhlB PE = 3 SV = 1 | 0.784355663 | 0.04148676 | Down |
|  | A0A4P0YBN2 | *gatD* | Galactitol-1-phosphate 5-dehydrogenase OS = *Escherichia coli* OX = 562 GN = gatD PE = 3 SV = 1 | 0.78374365 | 0.003808533 | Down |
|  | B7UQ77 | *emtA* | Endo-type membrane-bound lytic murein transglycosylase A OS = *Escherichia coli* O127 : H6 (strain E2348/69 / EPEC) OX = 574521 GN = emtA PE = 3 SV = 1 | 0.78366153 | 0.004854554 | Down |
|  | Q8XE60 | *lon* | Lon protease OS = *Escherichia coli* O157 : H7 OX = 83334 GN = lon PE = 2 SV = 2 | 0.78356383 | 0.000738615 | Down |
|  | A0A0J8XKA8 | *metC* | Cystathionine beta-lyase OS = *Escherichia coli* OX = 562 GN = metC PE = 3 SV = 1 | 0.782815877 | 0.000464088 | Down |
|  | A0A8B5MA57 | *gatZ* | Tagatose-bisphosphate aldolase subunit GatZ OS = *Escherichia coli* OX = 562 GN = gatZ PE = 4 SV = 1 | 0.782584667 | 0.017815832 | Down |
|  | A0A8A5IM57 | *tdcE* | 2-ketobutyrate formate-lyase/pyruvate formate-lyase OS = *Escherichia coli* O176 : H45 OX = 2810408 GN = tdcE PE = 4 SV = 1 | 0.78217172 | 0.046190901 | Down |
|  | P32053 | *intA* | Prophage integrase IntA OS = *Escherichia coli* (strain K12) OX = 83333 GN = intA PE = 1 SV = 2 | 0.781700449 | 0.001714774 | Down |
|  | A0A826I3D1 | C5909_001448 | Molecular chaperone HscC OS = *Escherichia coli* O108 : H9 OX = 2100504 GN = C5909_001448 PE = 4 SV = 1 | 0.779111478 | 0.00959549 | Down |
|  | A0A210BXS2 | *yncD* | Iron outer membrane transporter OS = *Escherichia coli* OX = 562 GN = yncD PE = 3 SV = 1 | 0.778983088 | 0.02578377 | Down |
|  | A0A0H2V995 | *cysK* | Cysteine synthase OS = *Escherichia coli* O6 : H1 (strain CFT073 / ATCC 700928 / UPEC) OX = 199310 GN = cysK PE = 3 SV = 1 | 0.778903921 | 0.000126191 | Down |
|  | A0A1X3LY07 | EAZG_01691 | Putative oxidoreductase Fe-S subunit OS = *Escherichia coli* TA249 OX = 656441 GN = EAZG_01691 PE = 4 SV = 1 | 0.778850625 | 0.023873243 | Down |
|  | D8A1D9 | HMPREF9530_00303 | Toxin-antitoxin system, toxin component, Fic domain protein OS = *Escherichia coli* (strain MS 21-1) OX = 749527 GN = HMPREF9530_00303 PE = 4 SV = 1 | 0.777856259 | 0.008658363 | Down |
|  | A0A4Y8FSS6 | BON92_21880 | ATP-dependent Clp protease ATP-binding subunit ClpA OS = *Escherichia coli* OX = 562 GN = BON92_21880 PE = 3 SV = 1 | 0.77686683 | 4.34677E-05 | Down |
|  | A0A831FLZ4 | *lon* | Endopeptidase La OS = *Escherichia coli* OX = 562 GN = lon PE = 4 SV = 1 | 0.776438172 | 0.024302398 | Down |
|  | D6I9G0 | *gatZ* | D-tagatose-1,6-bisphosphate aldolase subunit GatZ OS = *Escherichia coli* B185 OX = 550676 GN = gatZ PE = 3 SV = 1 | 0.776288812 | 0.001873847 | Down |
|  | A0A831DIL6 | HMU48_28895 | RNA chaperone Hfq OS = *Escherichia coli* OX = 562 GN = HMU48_28895 PE = 4 SV = 1 | 0.775751872 | 0.028561869 | Down |
|  | A0A6P1KG06 | *proP* | Glycine betaine/L-proline transporter ProP OS = *Escherichia coli* OX = 562 GN = proP PE = 3 SV = 1 | 0.775462114 | 0.009474662 | Down |
|  | A0A827Q848 | *exoX* | Exodeoxyribonuclease X OS = *Escherichia coli* OX = 562 GN = exoX PE = 4 SV = 1 | 0.775201345 | 0.011258376 | Down |
|  | A0A377KA41 | *ybeD* | Protein YbeD OS = *Escherichia coli* OX = 562 GN = ybeD PE = 3 SV = 1 | 0.774388134 | 0.006424584 | Down |
|  | A0A6M1HI81 | *ruvX* | Putative pre-16S rRNA nuclease OS = *Escherichia coli* OX = 562 GN = ruvX PE = 3 SV = 1 | 0.773353284 | 0.000485983 | Down |
|  | A0A2T1LIJ7 | C6985_13710 | SLC13 family permease OS = *Escherichia coli* OX = 562 GN = C6985_13710 PE = 4 SV = 1 | 0.773343526 | 0.011379515 | Down |
|  | B7MGL2 | *gpmA* | 2,3-bisphosphoglycerate-dependent phosphoglycerate mutase OS = *Escherichia coli* O45 : K1 (strain S88 / ExPEC) OX = 585035 GN = gpmA PE = 3 SV = 1 | 0.773047455 | 0.004556861 | Down |
|  | A0A376HGP0 | *tppB* | Dipeptide and tripeptide permease A OS = *Escherichia coli* OX = 562 GN = tppB PE = 3 SV = 1 | 0.772900144 | 0.005958994 | Down |
|  | Q9R5A8 | unknow | Ferric enterobactin protein receptor (Fragment) OS = *Escherichia coli* OX = 562 PE = 1 SV = 1 | 0.772712731 | 0.012978696 | Down |
|  | A0A827CGX5 | *nemA* | N-ethylmaleimide reductase OS = *Escherichia coli* OX = 562 GN = nemA PE = 4 SV = 1 | 0.772677944 | 0.013563593 | Down |
|  | A0A377A2N6 | *prc_5* | Tail-specific protease OS = *Escherichia coli* OX = 562 GN = prc_5 PE = 4 SV = 1 | 0.772491308 | 0.006420918 | Down |
|  | P0AD50 | *yfiA* | Ribosome-associated factor Y OS = *Escherichia coli* O6 : H1 (strain CFT073 / ATCC 700928 / UPEC) OX = 199310 GN = yfiA PE = 3 SV = 2 | 0.772305658 | 0.017175199 | Down |
|  | A0A376U2I8 | *hemL* | Glutamate-1-semialdehyde 2,1-aminomutase OS = *Escherichia coli* OX = 562 GN = hemL PE = 3 SV = 1 | 0.771665083 | 0.035444507 | Down |
|  | A0A8B3HX17 | EIA13_06520 | Maltoporin OS = *Escherichia coli* OX = 562 GN = EIA13_06520 PE = 4 SV = 1 | 0.771286349 | 0.04934568 | Down |
|  | P0AFW6 | *rnk* | Regulator of nucleoside diphosphate kinase OS = *Escherichia coli* O157 : H7 OX = 83334 GN = rnk PE = 3 SV = 1 | 0.77118931 | 0.014743514 | Down |
|  | A0A377D3X7 | *cysK* | Cysteine synthase OS = *Escherichia coli* OX = 562 GN = cysK PE = 4 SV = 1 | 0.770909578 | 0.000599799 | Down |
|  | A0A376RJ77 | *trg* | Methyl-accepting chemotaxis protein III (Ribose an galactose chemoreceptor protein) OS = *Escherichia coli* OX = 562 GN = trg PE = 4 SV = 1 | 0.770471624 | 0.02499829 | Down |
|  | F4VK71 | *asd* | Aspartate-semialdehyde dehydrogenase OS = *Escherichia coli* H591 OX = 656408 GN = asd PE = 3 SV = 1 | 0.769273609 | 0.000364207 | Down |
|  | A0A826TRD1 | *efp* | Elongation factor P OS = *Escherichia coli* OX = 562 GN = efp PE = 4 SV = 1 | 0.768451243 | 0.007953198 | Down |
|  | A0A234XVQ4 | D4V09_11885 | Class B acid phosphatase OS = *Escherichia coli* OX = 562 GN = D4V09_11885 PE = 3 SV = 1 | 0.767838284 | 0.014747383 | Down |
|  | A0A485DVY2 | *dnaJ_2* | Chaperone protein DnaJ OS = *Escherichia coli* OX = 562 GN = dnaJ_2 PE = 3 SV = 1 | 0.767020528 | 0.031670863 | Down |
|  | B7UFB0 | *gatB* | Galactitol-specific enzyme IIB component of PTS OS = *Escherichia coli* O127 : H6 (strain E2348/69 / EPEC) OX = 574521 GN = gatB PE = 4 SV = 1 | 0.767014921 | 0.031545359 | Down |
|  | A0A7U8X814 | ECFG_02822 | Aminopeptidase N OS = *Escherichia coli* FVEC1302 OX = 656379 GN = ECFG_02822 PE = 3 SV = 1 | 0.766257975 | 0.002828313 | Down |
|  | A0A5F1DKT1 | CQB02_23345 | Chemotaxis protein CheY OS = *Escherichia coli* OX = 562 GN = CQB02_23345 PE = 4 SV = 1 | 0.765851846 | 0.002526049 | Down |
|  | A0A024L1I7 | *cheB* | Protein-glutamate methylesterase/protein-glutamine glutaminase OS = *Escherichia coli* OX = 562 GN = cheB PE = 3 SV = 1 | 0.765580849 | 0.003620371 | Down |
|  | A0A2S8JKZ0 | *ipaB* | Putative cell invasion type III effector protein OS = *Escherichia coli* OX = 562 GN = ipaB PE = 4 SV = 1 | 0.765475623 | 0.031961265 | Down |
|  | A0A7U9B3A0 | ECMG_00535 | Chemotaxis protein MotB OS = *Escherichia coli* TA143 OX = 656437 GN = ECMG_00535 PE = 3 SV = 1 | 0.765345089 | 0.004916043 | Down |
|  | A0A376Q109 | *aceF* | Acetyltransferase component of pyruvate dehydrogenase complex OS = *Escherichia coli* OX = 562 GN = aceF PE = 3 SV = 1 | 0.765311015 | 0.001714758 | Down |
|  | A0A5E8NUZ0 | ACN68_19100 | Peptidyl-prolyl cis-trans isomerase OS = *Escherichia coli* OX = 562 GN = ACN68_19100 PE = 3 SV = 1 | 0.763759038 | 0.00156533 | Down |
|  | A0A5D8SP90 | E0I42_21390 | Peptidyl-prolyl cis-trans isomerase OS = *Escherichia coli* OX = 562 GN = E0I42_21390 PE = 3 SV = 1 | 0.762833044 | 0.001441317 | Down |
|  | D3GZF3 | *gatZ* | D-tagatose-1,6-bisphosphate aldolase subunit GatZ OS = *Escherichia coli* O44 : H18 (strain 042 / EAEC) OX = 216592 GN = gatZ PE = 3 SV = 1 | 0.761668459 | 0.044902938 | Down |
|  | A0A3L0VV53 | *aceE* | Pyruvate dehydrogenase E1 component OS = *Escherichia coli* OX = 562 GN = aceE PE = 4 SV = 1 | 0.761229507 | 0.002060439 | Down |
|  | A0A2T1LKD4 | C6985_09835 | Aldehyde ferredoxin oxidoreductase OS = *Escherichia coli* OX = 562 GN = C6985_09835 PE = 3 SV = 1 | 0.761027364 | 0.013181974 | Down |
|  | A0A377CXM6 | *folK_1* | 2-amino-4-hydroxy-6-hydroxymethyldihydropteridine pyrophosphokinase OS = *Escherichia coli* OX = 562 GN = folK_1 PE = 3 SV = 1 | 0.760421082 | 0.002184264 | Down |
|  | A0A1X9TR86 | *rihA* | Pyrimidine-specific ribonucleoside hydrolase RihA OS = *Escherichia coli* OX = 562 GN = rihA PE = 3 SV = 1 | 0.760325977 | 0.002527196 | Down |
|  | A0A829FJ04 | WCS_01189 | Nitrite extrusion protein 1 OS = *Escherichia coli* KTE14 OX = 1169333 GN = WCS_01189 PE = 4 SV = 1 | 0.76023755 | 0.011278693 | Down |
|  | A0A6S4W2J6 | WP2S18E08_47150 | DNA helicase OS = *Escherichia coli* OX = 562 GN = WP2S18E08_47150 PE = 3 SV = 1 | 0.76007601 | 0.014768723 | Down |
|  | A0A0K4GBA4 | *glpE* | Thiosulfate sulfurtransferase GlpE OS = *Escherichia coli* OX = 562 GN = glpE PE = 3 SV = 1 | 0.760006324 | 0.007570499 | Down |
|  | A0A4Y8FUT9 | *cheY* | Chemotaxis protein CheY OS = *Escherichia coli* OX = 562 GN = cheY PE = 4 SV = 1 | 0.759919778 | 0.01604383 | Down |
|  | A0A826YZR0 | FEL34_22920 | HNH endonuclease OS = *Escherichia coli* OX = 562 GN = FEL34_22920 PE = 4 SV = 1 | 0.759773704 | 0.029858437 | Down |
|  | A0A6M0PZM1 | *csrA* | Translational regulator CsrA OS = *Escherichia coli* OX = 562 GN = csrA PE = 3 SV = 1 | 0.759682911 | 0.003702056 | Down |
|  | A0A1M2HSR1 | *ygaP* | Inner membrane protein YgaP OS = *Escherichia coli* OX = 562 GN = ygaP PE = 4 SV = 1 | 0.759634198 | 0.003796624 | Down |
|  | A0A854RJR3 | *clpP* | ATP-dependent Clp endopeptidase, proteolytic subunit ClpP OS = *Escherichia coli* OX = 562 GN = clpP PE = 4 SV = 1 | 0.758964827 | 6.96458E-05 | Down |
|  | A0A7H9QNW5 | *pepN* | Aminopeptidase N OS = *Escherichia coli* OX = 562 GN = pepN PE = 3 SV = 1 | 0.758931356 | 0.00086022 | Down |
|  | A0A827DZ73 | A2F99_001257 | LysR family transcriptional regulator OS = *Escherichia coli* OX = 562 GN = A2F99_001257 PE = 4 SV = 1 | 0.757939377 | 0.00179043 | Down |
|  | A0A376SXV3 | *rraA* | Regulator of ribonuclease activity A OS = *Escherichia coli* OX = 562 GN = rraA PE = 3 SV = 1 | 0.757448493 | 0.042227449 | Down |
|  | P0A7M2 | *rpmB* | 50S ribosomal protein L28 OS = *Escherichia coli* (strain K12) OX = 83333 GN = rpmB PE = 1 SV = 2 | 0.75738965 | 0.006682046 | Down |
|  | A0A7U3BCZ4 | *flgF* | Flagellar basal body protein OS = *Escherichia coli* OX = 562 GN = flgF PE = 3 SV = 1 | 0.756090563 | 0.033980834 | Down |
|  | A0A6M0PZE3 | *accC* | Biotin carboxylase OS = *Escherichia coli* OX = 562 GN = accC PE = 4 SV = 1 | 0.755914735 | 0.005924885 | Down |
|  | A0A6D0ISL3 | *kdsA* | 2-dehydro-3-deoxyphosphooctonate aldolase OS = *Escherichia coli* OX = 562 GN = kdsA PE = 3 SV = 1 | 0.755855491 | 0.003829207 | Down |
|  | A0A2T1LPG8 | C6985_01380 | Cytosol nonspecific dipeptidase OS = *Escherichia coli* OX = 562 GN = C6985_01380 PE = 4 SV = 1 | 0.755067093 | 0.023241151 | Down |
|  | A0A6L4XKB9 | *iscA* | Iron-binding protein IscA OS = *Escherichia coli* OX = 562 GN = iscA PE = 3 SV = 1 | 0.753648004 | 0.014749762 | Down |
|  | A0A828FPP9 | AAS29_000306 | Non-heme ferritin-like protein OS = *Escherichia coli* OX = 562 GN = AAS29_000306 PE = 4 SV = 1 | 0.75343957 | 0.00123463 | Down |
|  | A0A827G2Z0 | *alsR* | Als operon DNA-binding transcriptional repressor AlsR OS = *Escherichia coli* OX = 562 GN = alsR PE = 4 SV = 1 | 0.751700841 | 0.018903357 | Down |
|  | A0A5F1DXH6 | CQB02_08155 | Alkene reductase OS = *Escherichia coli* OX = 562 GN = CQB02_08155 PE = 4 SV = 1 | 0.751587579 | 0.015012398 | Down |
|  | A0A5N8HAY0 | *fumD* | Fumarase D OS = *Escherichia coli* OX = 562 GN = fumD PE = 3 SV = 1 | 0.751471101 | 0.013874377 | Down |
|  | Q8XDE8 | *pncB* | Nicotinate phosphoribosyltransferase OS = *Escherichia coli* O157 : H7 OX = 83334 GN = pncB PE = 3 SV = 3 | 0.750911778 | 0.005146455 | Down |
|  | E9THW5 | *fliZ* | Flagellar regulatory protein FliZ OS = *Escherichia coli* MS 117-3 OX = 749539 GN = fliZ PE = 3 SV = 1 | 0.750676454 | 0.003915087 | Down |
|  | A0A2H9EUJ5 | *ndk* | Nucleoside diphosphate kinase OS = *Escherichia coli* OX = 562 GN = ndk PE = 3 SV = 1 | 0.749963141 | 0.022969927 | Down |
|  | A0A6L4XLZ2 | *wecC* | UDP-N-acetyl-D-mannosamine dehydrogenase OS = *Escherichia coli* OX = 562 GN = wecC PE = 3 SV = 1 | 0.749914234 | 0.028996582 | Down |
|  | A0A827JUP0 | *gcvP* | Aminomethyl-transferring glycine dehydrogenase OS = *Escherichia coli* OX = 562 GN = gcvP PE = 4 SV = 1 | 0.748857522 | 0.001801241 | Down |
|  | A0A7U2A5P4 | *dcuA* | Anaerobic C4-dicarboxylate transporter OS = *Escherichia coli* OX = 562 GN = dcuA PE = 3 SV = 1 | 0.748383461 | 0.00255214 | Down |
|  | P0AB72 | *fbaA* | Fructose-bisphosphate aldolase class 2 OS = *Escherichia coli* O157 : H7 OX = 83334 GN = fbaA PE = 3 SV = 2 | 0.748251147 | 0.002287846 | Down |
|  | A0A2T1LCH0 | C6985_23965 | Cold-shock protein OS = *Escherichia coli* OX = 562 GN = C6985_23965 PE = 4 SV = 1 | 0.748084596 | 0.003074318 | Down |
|  | A0A1V3W1U0 | BMT91_06785 | Transposase OS = *Escherichia coli* OX = 562 GN = BMT91_06785 PE = 3 SV = 1 | 0.747470142 | 0.014702289 | Down |
|  | A0A6M0PTL1 | *trpB* | Tryptophan synthase beta chain OS = *Escherichia coli* OX = 562 GN = trpB PE = 3 SV = 1 | 0.747331611 | 0.042336439 | Down |
|  | A0A0K3QKD8 | *tpiA* | Triosephosphate isomerase OS = *Escherichia coli* OX = 562 GN = tpiA PE = 3 SV = 1 | 0.746197742 | 0.019977813 | Down |
|  | A0A6M1I0C1 | G5632_32545 | Aldo/keto reductase (Fragment) OS = *Escherichia coli* OX = 562 GN = G5632_32545 PE = 4 SV = 1 | 0.744946087 | 0.007488474 | Down |
|  | A0A2X1N3J8 | *aspA* | Aspartate ammonia-lyase OS = *Escherichia coli* OX = 562 GN = aspA PE = 3 SV = 1 | 0.744628365 | 0.008625048 | Down |
|  | A0A376VU06 | *tufA_2* | Elongation factor Tu OS = *Escherichia coli* OX = 562 GN = tufA_2 PE = 4 SV = 1 | 0.744411464 | 0.027233177 | Down |
|  | A0A774NC69 | GFY48_24340 | S-formylglutathione hydrolase OS = *Escherichia coli* OX = 562 GN = GFY48_24340 PE = 3 SV = 1 | 0.743830092 | 0.006401885 | Down |
|  | A0A5F1DL70 | *hslU* | ATP-dependent protease ATPase subunit HslU OS = *Escherichia coli* OX = 562 GN = hslU PE = 2 SV = 1 | 0.742659301 | 0.001901698 | Down |
|  | A0A376TX29 | *tdcE_5* | Keto-acid formate acetyltransferase OS = *Escherichia coli* OX = 562 GN = tdcE_5 PE = 4 SV = 1 | 0.742204183 | 0.002195835 | Down |
|  | A0A660HCD7 | A9X72_10295 | Ferritin OS = *Escherichia coli* OX = 562 GN = A9X72_10295 PE = 3 SV = 1 | 0.742160316 | 0.000385561 | Down |
|  | A0A271QLV6 | *yaiI* | UPF0178 protein YaiI OS = *Escherichia coli* OX = 562 GN = yaiI PE = 3 SV = 1 | 0.741416959 | 0.020668017 | Down |
|  | A0A845P5Y3 | GUB92_17650 | Cytochrome o ubiquinol oxidase subunit III OS = *Escherichia coli* OX = 562 GN = GUB92_17650 PE = 4 SV = 1 | 0.740581001 | 0.026432892 | Down |
|  | U9XL08 | HMPREF1589_04929 | Uncharacterized protein OS = *Escherichia coli* 113290 OX = 1268976 GN = HMPREF1589_04929 PE = 4 SV = 1 | 0.73926341 | 0.038241894 | Down |
|  | Q6JZC5 | *trpB* | Tryptophan synthase beta chain (Fragment) OS = *Escherichia coli* OX = 562 GN = trpB PE = 3 SV = 1 | 0.739061885 | 2.82349E-05 | Down |
|  | A0A2X1PFM4 | *ygiB* | UPF0441 protein YgiB OS = *Escherichia coli* OX = 562 GN = ygiB PE = 3 SV = 1 | 0.738588977 | 0.00154067 | Down |
|  | A0A837MF64 | SM09_00856 | Pyruvate dehydrogenase E1 component OS = *Escherichia coli* OX = 562 GN = SM09_00856 PE = 4 SV = 1 | 0.738466046 | 0.002868095 | Down |
|  | A0A828S3C4 | *rpoN* | RNA polymerase sigma-54 factor OS = *Escherichia coli* STEC_7v OX = 754082 GN = rpoN PE = 4 SV = 1 | 0.738439868 | 0.008481172 | Down |
|  | Q57132 | *mob1* | Mob1 protein (Gtg start codon) OS = *Escherichia coli* OX = 562 GN = mob1 PE = 4 SV = 1 | 0.736248721 | 0.002436661 | Down |
|  | E6BD97 | *galE* | UDP-glucose 4-epimerase OS = *Escherichia coli* MS 85-1 OX = 679202 GN = galE PE = 3 SV = 1 | 0.735259147 | 0.010173709 | Down |
|  | A0A3Q0MYR4 | CR539_12755 | Oxidoreductase OS = *Escherichia coli* OX = 562 GN = CR539_12755 PE = 4 SV = 1 | 0.734694011 | 0.037341378 | Down |
|  | A0A0H2V4K9 | *pepD* | Aminoacyl-histidine dipeptidase OS = *Escherichia coli* O6 : H1 (strain CFT073 / ATCC 700928 / UPEC) OX = 199310 GN = pepD PE = 4 SV = 1 | 0.733864739 | 0.007942675 | Down |
|  | A0A4P0YY48 | *pepD_1* | Aminoacyl-histidine dipeptidase OS = *Escherichia coli* OX = 562 GN = pepD_1 PE = 4 SV = 1 | 0.733697632 | 0.000213133 | Down |
|  | W1X0Z6 | Q609_ECAC01641G0005 | PTS system maltose-and glucose-specific EIICB component OS = *Escherichia coli* DORA_A_5_14_21 OX = 1403943 GN = Q609_ECAC01641G0005 PE = 4 SV = 1 | 0.733054028 | 0.008280623 | Down |
|  | A0A1M2GPQ1 | *galK* | Galactokinase OS = *Escherichia coli* OX = 562 GN = galK PE = 3 SV = 1 | 0.732637907 | 0.005656349 | Down |
|  | A0A376TRN1 | *deaD_1* | ATP-dependent RNA helicase (DEAD-box protein) OS = *Escherichia coli* OX = 562 GN = deaD_1 PE = 4 SV = 1 | 0.73235881 | 8.63156E-05 | Down |
|  | U9Z5W5 | HMPREF1589_00327 | Uncharacterized protein OS = *Escherichia coli* 113290 OX = 1268976 GN = HMPREF1589_00327 PE = 4 SV = 1 | 0.732248184 | 0.011778841 | Down |
|  | V0YJG2 | HMPREF1611_03527 | Isoaspartyl dipeptidase OS = *Escherichia coli* 908573 OX = 1268998 GN = HMPREF1611_03527 PE = 3 SV = 1 | 0.730430537 | 0.0260228 | Down |
|  | A0A6D0LQA8 | *yajO* | 1-deoxyxylulose-5-phosphate synthase YajO OS = *Escherichia coli* OX = 562 GN = yajO PE = 4 SV = 1 | 0.729060711 | 0.002543678 | Down |
|  | A0A2X7IQM9 | *gpmA* | 2,3-bisphosphoglycerate-dependent phosphoglycerate mutase OS = *Escherichia coli* OX = 562 GN = gpmA PE = 3 SV = 1 | 0.727468874 | 0.007194159 | Down |
|  | A0A810TKM9 | *chbF* | 6-phospho-beta-glucosidase OS = *Escherichia coli* OX = 562 GN = chbF PE = 4 SV = 1 | 0.725796195 | 0.043612983 | Down |
|  | A0A827UE97 | *cdd* | Cytidine deaminase OS = *Escherichia coli* OX = 562 GN = cdd PE = 4 SV = 1 | 0.72517959 | 0.018142866 | Down |
|  | A0A826YR84 | *srlB* | PTS glucitol/sorbitol transporter subunit IIA OS = *Escherichia coli* OX = 562 GN = srlB PE = 4 SV = 1 | 0.724704077 | 0.006700394 | Down |
|  | A0A891ZVH5 | unknow | Uncharacterized protein OS = *Escherichia coli* OX = 562 PE = 4 SV = 1 | 0.723358774 | 0.047534085 | Down |
|  | A0A4Q0BG29 | ELY48_00195 | Thymidylate kinase OS = *Escherichia coli* OX = 562 GN = ELY48_00195 PE = 4 SV = 1 | 0.72332651 | 0.008384212 | Down |
|  | A0A4V3QD96 | *crl* | Sigma factor-binding protein Crl OS = *Escherichia coli* OX = 562 GN = crl PE = 3 SV = 1 | 0.723012602 | 0.030847886 | Down |
|  | A0A417ZXX0 | D3O91_08965 | PTS glucitol/sorbitol transporter subunit IIA OS = *Escherichia coli* OX = 562 GN = D3O91_08965 PE = 4 SV = 1 | 0.722974806 | 0.012416246 | Down |
|  | W1ETG5 | unknow | Uncharacterized protein OS = *Escherichia coli* ISC7 OX = 1432555 PE = 4 SV = 1 | 0.721784753 | 0.00527945 | Down |
|  | F4SVE4 | ECIG_00164 | Cold shock protein OS = *Escherichia coli* M605 OX = 656417 GN = ECIG_00164 PE = 4 SV = 1 | 0.720957676 | 0.004974327 | Down |
|  | A0A6N7KEC5 | *dmlA* | D-malate dehydrogenase [decarboxylating] OS = *Escherichia coli* OX = 562 GN = dmlA PE = 3 SV = 1 | 0.720935395 | 0.004332138 | Down |
|  | A0A826WTR0 | *gatZ* | Tagatose-bisphosphate aldolase subunit GatZ OS = *Escherichia coli* OX = 562 GN = gatZ PE = 4 SV = 1 | 0.72019578 | 0.023749777 | Down |
|  | A0A5C9A7M4 | *dinD* | DNA damage-inducible protein D (Fragment) OS = *Escherichia coli* OX = 562 GN = dinD PE = 4 SV = 1 | 0.720044102 | 0.000585502 | Down |
|  | A0A3K2YEL7 | D9J61_17275 | 2-oxoglutarate reductase OS = *Escherichia coli* OX = 562 GN = D9J61_17275 PE = 3 SV = 1 | 0.718418408 | 3.56578E-05 | Down |
|  | A0A6D0FFI4 | GQM06_36645 | DUF3459 domain-containing protein (Fragment) OS = *Escherichia coli* OX = 562 GN = GQM06_36645 PE = 3 SV = 1 | 0.718346396 | 0.032133533 | Down |
|  | A0A2S8JN98 | *pepD* | Aminoacyl-histidine dipeptidase OS = *Escherichia coli* OX = 562 GN = pepD PE = 4 SV = 1 | 0.718273033 | 0.004148624 | Down |
|  | A0A6D0F4E6 | GQM06_24570 | TIM barrel protein (Fragment) OS = *Escherichia coli* OX = 562 GN = GQM06_24570 PE = 4 SV = 1 | 0.71782187 | 0.025674376 | Down |
|  | A0A826XBG0 | *gatB* | PTS galactitol transporter subunit IIB OS = *Escherichia coli* OX = 562 GN = gatB PE = 4 SV = 1 | 0.71604053 | 0.00613096 | Down |
|  | A0A831DZJ5 | HMV95_06490 | Peptidylprolyl isomerase OS = *Escherichia coli* OX = 562 GN = HMV95_06490 PE = 4 SV = 1 | 0.715963983 | 0.009983535 | Down |
|  | A0A826X6Z1 | *mepS* | Bifunctional murein DD-endopeptidase/murein LD-carboxypeptidase OS = *Escherichia coli* OX = 562 GN = mepS PE = 4 SV = 1 | 0.713853867 | 0.002874219 | Down |
|  | A9X4P5 | *gyrB* | DNA topoisomerase (ATP-hydrolyzing) (Fragment) OS = *Escherichia coli* OX = 562 GN = gyrB PE = 3 SV = 1 | 0.713303855 | 0.001836376 | Down |
|  | A0A4C3QKN0 | *lsrK* | Autoinducer-2 kinase OS = *Escherichia coli* OX = 562 GN = lsrK PE = 3 SV = 1 | 0.713231862 | 0.039745874 | Down |
|  | V0SH21 | HMPREF1595_03595 | Putative selenate reductase, YgfK subunit OS = *Escherichia coli* 907672 OX = 1268982 GN = HMPREF1595_03595 PE = 4 SV = 1 | 0.711262565 | 0.00285442 | Down |
|  | A0A5D8S3Y2 | *fliD* | Filament cap protein (Fragment) OS = *Escherichia coli* OX = 562 GN = fliD PE = 3 SV = 1 | 0.710959066 | 0.013435731 | Down |
|  | A0A7H9LRW3 | *cobO* | Corrinoid adenosyltransferase OS = *Escherichia coli* OX = 562 GN = cobO PE = 3 SV = 1 | 0.710269262 | 0.011687864 | Down |
|  | A0A1V2GF16 | BXT93_12685 | Iron-sulfur cluster assembly scaffold protein IscU (Fragment) OS = *Escherichia coli* OX = 562 GN = BXT93_12685 PE = 3 SV = 1 | 0.70942544 | 0.009144638 | Down |
|  | A0A799VWC7 | *dcuC* | Anaerobic C4-dicarboxylate transporter DcuC OS = *Escherichia coli* OX = 562 GN = dcuC PE = 3 SV = 1 | 0.707977382 | 0.006734997 | Down |
|  | A0A7H9LUQ5 | *pdxY* | Pyridoxal kinase PdxY OS = *Escherichia coli* OX = 562 GN = pdxY PE = 3 SV = 1 | 0.707796138 | 0.023285472 | Down |
|  | A0A3A6SBY8 | BMC79_003960 | OmpA family protein OS = *Escherichia coli* OX = 562 GN = BMC79_003960 PE = 4 SV = 1 | 0.706831551 | 0.003118624 | Down |
|  | A0A777CE63 | *rhlE* | ATP-dependent RNA helicase RhlE OS = *Escherichia coli* OX = 562 GN = rhlE PE = 3 SV = 1 | 0.7059119 | 0.00476425 | Down |
|  | A0A5F1E0X3 | *cdd* | Cytidine deaminase OS = *Escherichia coli* OX = 562 GN = cdd PE = 3 SV = 1 | 0.702613817 | 0.002557402 | Down |
|  | A0A826X8H6 | CSE52_003051 | YchJ family protein OS = *Escherichia coli* OX = 562 GN = CSE52_003051 PE = 4 SV = 1 | 0.701505672 | 0.008037265 | Down |
|  | A0A768EGW4 | HMT45_14985 | PTS galactitol transporter subunit IIA OS = *Escherichia coli* OX = 562 GN = HMT45_14985 PE = 4 SV = 1 | 0.700186378 | 0.001752571 | Down |
|  | A0A377B2G3 | cof_1 | HMP-PP phosphatase OS = *Escherichia coli* OX = 562 GN = cof_1 PE = 3 SV = 1 | 0.699015245 | 8.05135E-05 | Down |
|  | A0A7I9ASU8 | ETECE925_04375 | Dynamin-type G domain-containing protein OS = *Escherichia coli* OX = 562 GN = ETECE925_04375 PE = 4 SV = 1 | 0.698456094 | 0.005571669 | Down |
|  | A0A827ABQ5 | CN875_002057 | NADP(H)-dependent aldo-keto reductase OS = *Escherichia coli* OX = 562 GN = CN875_002057 PE = 4 SV = 1 | 0.697635052 | 0.004861184 | Down |
|  | A0A1U9SZS3 | *ribH* | 6,7-dimethyl-8-ribityllumazine synthase OS = *Escherichia coli* OX = 562 GN = ribH PE = 3 SV = 1 | 0.697365084 | 0.000884008 | Down |
|  | A0A2T1LH30 | *lexA* | LexA repressor OS = *Escherichia coli* OX = 562 GN = lexA PE = 3 SV = 1 | 0.696941649 | 0.001230048 | Down |
|  | J7QSF1 | *fliO* | Flagellar protein OS = *Escherichia coli* OX = 562 GN = fliO PE = 3 SV = 1 | 0.695441571 | 0.000994002 | Down |
|  | A0A376MTF2 | *nagB_1* | Glucosamine-6-phosphate isomerase OS = *Escherichia coli* OX = 562 GN = nagB_1 PE = 4 SV = 1 | 0.6944376 | 0.00090634 | Down |
|  | A0A8B5MAI7 | *yeaE* | Methylglyoxal reductase YeaE OS = *Escherichia coli* OX = 562 GN = yeaE PE = 4 SV = 1 | 0.69393733 | 0.0007669 | Down |
|  | A0A789MBN3 | *gatB* | PTS galactitol transporter subunit IIB OS = *Escherichia coli* OX = 562 GN = gatB PE = 4 SV = 1 | 0.691379321 | 0.048746189 | Down |
|  | A0A0H3EKR0 | NRG857_10630 | Galactitol-specific PTS system component IIA OS = *Escherichia coli* O83 : H1 (strain NRG 857C / AIEC) OX = 685038 GN = NRG857_10630 PE = 4 SV = 1 | 0.687742308 | 0.001945015 | Down |
|  | A0A828URM2 | *gcvH* | Glycine cleavage system H protein OS = *Escherichia coli* 3.2608 OX = 869679 GN = gcvH PE = 4 SV = 1 | 0.685875571 | 0.014977762 | Down |
|  | A0A271R642 | BIZ41_06685 | Universal stress protein UspE OS = *Escherichia coli* OX = 562 GN = BIZ41_06685 PE = 4 SV = 1 | 0.685280301 | 0.000708095 | Down |
|  | W1X365 | Q609_ECAC01377G0008 | D-tagatose-1,6-bisphosphate aldolase subunit kbaZ (Fragment) OS = *Escherichia coli* DORA_A_5_14_21 OX = 1403943 GN = Q609_ECAC01377G0008 PE = 4 SV = 1 | 0.684543363 | 0.001738225 | Down |
|  | P42616 | *yqjC* | Protein YqjC OS = *Escherichia coli* (strain K12) OX = 83333 GN = yqjC PE = 1 SV = 3 | 0.684113545 | 0.003645796 | Down |
|  | A0A7U9LV71 | *ydhW* | Uncharacterized protein OS = *Escherichia coli* O145 : H28 OX = 1078034 GN = ydhW PE = 4 SV = 1 | 0.683830544 | 0.001983583 | Down |
|  | A0A376HYQ1 | *trpB* | Tryptophan synthase beta chain OS = *Escherichia coli* OX = 562 GN = trpB PE = 3 SV = 1 | 0.68375765 | 0.001087947 | Down |
|  | A0A383G4P5 | *ppc* | Phosphoenolpyruvate carboxylase OS = *Escherichia coli* OX = 562 GN = ppc PE = 3 SV = 1 | 0.682131669 | 0.000140504 | Down |
|  | U9XK73 | HMPREF1589_05182 | Hemolysin expression-modulating protein OS = *Escherichia coli* 113290 OX = 1268976 GN = HMPREF1589_05182 PE = 3 SV = 1 | 0.681554915 | 0.00971806 | Down |
|  | A0A346GGF1 | *rihA* | Pyrimidine-specific ribonucleoside hydrolase RihA OS = *Escherichia coli* OX = 562 GN = rihA PE = 3 SV = 1 | 0.6813163 | 0.007811036 | Down |
|  | A0A5D8MJ02 | E0I42_15750 | PTS glucitol/sorbitol transporter subunit IIA OS = *Escherichia coli* OX = 562 GN = E0I42_15750 PE = 4 SV = 1 | 0.681221731 | 0.005217842 | Down |
|  | A0A288XG28 | DL654_25255 | Arabinose 5-phosphate isomerase OS = *Escherichia coli* OX = 562 GN = DL654_25255 PE = 3 SV = 1 | 0.680995976 | 0.018040784 | Down |
|  | A7ZUJ2 | *tuf2* | Elongation factor Tu 2 OS = *Escherichia coli* O139 : H28 (strain E24377A / ETEC) OX = 331111 GN = tuf2 PE = 3 SV = 1 | 0.680002235 | 0.032900714 | Down |
|  | A0A6M0PZR8 | *fis* | DNA-binding protein Fis OS = *Escherichia coli* OX = 562 GN = fis PE = 3 SV = 1 | 0.677511907 | 0.003097181 | Down |
|  | A0A6N8QE93 | *pnp* | Polyribonucleotide nucleotidyltransferase OS = *Escherichia coli* OX = 562 GN = pnp PE = 3 SV = 1 | 0.675113421 | 6.10123E-05 | Down |
|  | A0A377CBD1 | *sodA* | Superoxide dismutase OS = *Escherichia coli* OX = 562 GN = sodA PE = 3 SV = 1 | 0.673207868 | 0.019038738 | Down |
|  | A0A6D0UCH6 | *gatZ* | D-tagatose-1,6-bisphosphate aldolase subunit GatZ OS = *Escherichia coli* OX = 562 GN = gatZ PE = 3 SV = 1 | 0.672296711 | 0.003824062 | Down |
|  | A0A6N8P9J3 | *gatZ* | D-tagatose-1,6-bisphosphate aldolase subunit GatZ OS = *Escherichia coli* OX = 562 GN = gatZ PE = 3 SV = 1 | 0.672267593 | 0.005078333 | Down |
|  | A0A6M7GT65 | E3158_01100 | Glutathione S-transferase OS = *Escherichia coli* O157 : H7 OX = 83334 GN = E3158_01100 PE = 4 SV = 1 | 0.67217165 | 0.018777525 | Down |
|  | P69222 | *infA* | Translation initiation factor IF-1 OS = *Escherichia coli* (strain K12) OX = 83333 GN = infA PE = 1 SV = 2 | 0.670260107 | 0.014207218 | Down |
|  | A0A2A3WKJ5 | BB545_24830 | Flagellar hook-associated protein 2 OS = *Escherichia coli* OX = 562 GN = BB545_24830 PE = 3 SV = 1 | 0.667469957 | 0.022941963 | Down |
|  | A0A2K4P2A8 | EC1094V2_4975 | NAD(P)H dehydrogenase (quinone) OS = *Escherichia coli* OX = 562 GN = EC1094V2_4975 PE = 3 SV = 1 | 0.66693354 | 0.012549566 | Down |
|  | U9YV70 | HMPREF1589_00495 | Putative dihydrodipicolinate synthase OS = *Escherichia coli* 113290 OX = 1268976 GN = HMPREF1589_00495 PE = 3 SV = 1 | 0.666619952 | 0.049193752 | Down |
|  | A0A3G8RE03 | *caiF* | CaiF/GrlA family transcriptional regulator OS = *Escherichia coli* OX = 562 GN = caiF PE = 4 SV = 1 | 0.666587793 | 0.020138654 | Down |
|  | B6I4S5 | *hslV* | ATP-dependent protease subunit HslV OS = *Escherichia coli* (strain SE11) OX = 409438 GN = hslV PE = 3 SV = 1 | 0.665364836 | 0.015760399 | Down |
|  | A0A6D0GUE7 | *crl* | Sigma factor-binding protein Crl OS = *Escherichia coli* OX = 562 GN = crl PE = 3 SV = 1 | 0.662911766 | 0.01019494 | Down |
|  | A0A828S4S8 | *lacF* | Cellobiose-specific phosphotransferase enzyme IIA component OS = *Escherichia coli* STEC_7v OX = 754082 GN = lacF PE = 4 SV = 1 | 0.662038691 | 0.004180821 | Down |
|  | A0A417ZQI7 | *pspA* | Phage shock protein PspA OS = *Escherichia coli* OX = 562 GN = pspA PE = 3 SV = 1 | 0.661650226 | 0.000195083 | Down |
|  | A0A5D1FPW1 | *motA* | Flagellar motor stator protein MotA OS = *Escherichia coli* OX = 562 GN = motA PE = 3 SV = 1 | 0.660772478 | 0.008005981 | Down |
|  | A0A6D0EJ65 | GQM09_24560 | Pyridoxal-phosphate dependent enzyme (Fragment) OS = *Escherichia coli* OX = 562 GN = GQM09_24560 PE = 4 SV = 1 | 0.659249099 | 0.026277305 | Down |
|  | A0A0H3EIE9 | *znuA* | High-affinity zinc uptake system protein ZnuA OS = *Escherichia coli* O83 : H1 (strain NRG 857C / AIEC) OX = 685038 GN = znuA PE = 3 SV = 1 | 0.657237963 | 2.17585E-05 | Down |
|  | A0A1E5WU20 | DNQ45_24495 | PTS galactitol transporter subunit IIB OS = *Escherichia coli* OX = 562 GN = DNQ45_24495 PE = 4 SV = 1 | 0.652074708 | 0.0007556 | Down |
|  | Q6JZ44 | *trpA* | Tryptophan synthase (Fragment) OS = *Escherichia coli* OX = 562 GN = trpA PE = 3 SV = 1 | 0.650647227 | 0.014076068 | Down |
|  | A0A4Y8GFI5 | BON92_01645 | Alpha-ketoglutarate transporter OS = *Escherichia coli* OX = 562 GN = BON92_01645 PE = 3 SV = 1 | 0.648781151 | 0.004438477 | Down |
|  | A0A7H9LQH0 | HV146_06765 | PhoH family protein OS = *Escherichia coli* OX = 562 GN = HV146_06765 PE = 3 SV = 1 | 0.648743695 | 0.002059174 | Down |
|  | A0A7A2V838 | *ppnP* | Pyrimidine/purine nucleoside phosphorylase OS = *Escherichia coli* OX = 562 GN = ppnP PE = 3 SV = 1 | 0.648137575 | 0.000228712 | Down |
|  | A0A853WCM3 | BJI68_19085 | Uncharacterized protein OS = *Escherichia coli* OX = 562 GN = BJI68_19085 PE = 4 SV = 1 | 0.643897126 | 0.009229995 | Down |
|  | A0A830SFJ1 | HHH44_000902 | Ferredoxin-like protein OS = *Escherichia coli* OX = 562 GN = HHH44_000902 PE = 4 SV = 1 | 0.640483253 | 0.002763257 | Down |
|  | D7Y521 | *pnp* | Polyribonucleotide nucleotidyltransferase OS = *Escherichia coli* (strain MS 115-1) OX = 749537 GN = pnp PE = 3 SV = 1 | 0.639806027 | 0.000193401 | Down |
|  | A0A844EWT9 | *uspE* | Universal stress protein UspE OS = *Escherichia coli* OX = 562 GN = uspE PE = 4 SV = 1 | 0.634605818 | 0.018249571 | Down |
|  | A0A0J2BU24 | *zntR* | HTH-type transcriptional regulator ZntR OS = *Escherichia coli* OX = 562 GN = zntR PE = 4 SV = 1 | 0.633085245 | 0.00712752 | Down |
|  | B1LQY8 | EcSMS35_2935 | AMP nucleosidase OS = *Escherichia coli* (strain SMS-3-5 / SECEC) OX = 439855 GN = EcSMS35_2935 PE = 4 SV = 1 | 0.632349796 | 0.001398732 | Down |
|  | A0A777TQ67 | GF199_03260 | Aldo/keto reductase OS = *Escherichia coli* OX = 562 GN = GF199_03260 PE = 4 SV = 1 | 0.630923718 | 0.00807968 | Down |
|  | A0A826L929 | *proV* | Glycine betaine/L-proline ABC transporter ATP-binding protein ProV OS = *Escherichia coli* OX = 562 GN = proV PE = 4 SV = 1 | 0.629086759 | 0.00441068 | Down |
|  | A0A7D7I018 | *nemA* | N-ethylmaleimide reductase OS = *Escherichia coli* OX = 562 GN = nemA PE = 4 SV = 1 | 0.62806371 | 0.048110637 | Down |
|  | E6BK96 | HMPREF9350_02703 | Uncharacterized protein OS = *Escherichia coli* MS 85-1 OX = 679202 GN = HMPREF9350_02703 PE = 4 SV = 1 | 0.626613672 | 0.006418987 | Down |
|  | A0A765T0J1 | *pnp* | Polyribonucleotide nucleotidyltransferase OS = *Escherichia coli* OX = 562 GN = pnp PE = 3 SV = 1 | 0.624150917 | 0.000357036 | Down |
|  | A0A7L7XED2 | *deaD* | ATP-dependent RNA helicase DeaD OS = *Escherichia coli* O18ac : H14 OX = 2773705 GN = deaD PE = 3 SV = 1 | 0.623246072 | 0.000528209 | Down |
|  | A0A826XI71 | *srlB* | PTS glucitol/sorbitol transporter subunit IIA OS = *Escherichia coli* OX = 562 GN = srlB PE = 4 SV = 1 | 0.622313146 | 0.040264138 | Down |
|  | A0A6M0PQ81 | G4V03_00695 | 4Fe-4S dicluster domain-containing protein OS = *Escherichia coli* OX = 562 GN = G4V03_00695 PE = 4 SV = 1 | 0.621047061 | 0.002750444 | Down |
|  | A0A4Y8DX03 | BON63_04935 | SLC13 family permease OS = *Escherichia coli* OX = 562 GN = BON63_04935 PE = 4 SV = 1 | 0.620382757 | 0.02381331 | Down |
|  | A0A0E1SXQ0 | *gcvT* | Aminomethyltransferase OS = *Escherichia coli* 53638 OX = 344610 GN = gcvT PE = 3 SV = 1 | 0.620261967 | 0.003548908 | Down |
|  | A0A080IZS0 | AC28_1820 | Aldehyde ferredoxin oxidoreductase, domains 2 & 3 family protein OS = *Escherichia coli* 1-250-04_S3_C2 OX = 1444163 GN = AC28_1820 PE = 3 SV = 1 | 0.618253839 | 0.003971383 | Down |
|  | A0A376RE19 | *proX_1* | Glycine betaine/L-proline ABC transporter, substrate-binding protein OS = *Escherichia coli* OX = 562 GN = proX_1 PE = 4 SV = 1 | 0.617693911 | 0.028384759 | Down |
|  | A0A5D8MVY1 | *deaD* | ATP-dependent RNA helicase DeaD OS = *Escherichia coli* OX = 562 GN = deaD PE = 3 SV = 1 | 0.616163862 | 0.000570675 | Down |
|  | A0A377CD10 | *gatZ* | D-tagatose-1,6-bisphosphate aldolase subunit GatZ OS = *Escherichia coli* OX = 562 GN = gatZ PE = 3 SV = 1 | 0.614999555 | 0.000214197 | Down |
|  | U9Y2M9 | HMPREF1589_03441 | Fimbrial protein OS = *Escherichia coli* 113290 OX = 1268976 GN = HMPREF1589_03441 PE = 4 SV = 1 | 0.614480949 | 0.002817463 | Down |
|  | A0A6M0PXN7 | *malE* | Maltodextrin-binding protein OS = *Escherichia coli* OX = 562 GN = malE PE = 3 SV = 1 | 0.61307059 | 0.047896091 | Down |
|  | A0A7H9LVC8 | *gatZ* | D-tagatose-1,6-bisphosphate aldolase subunit GatZ OS = *Escherichia coli* OX = 562 GN = gatZ PE = 3 SV = 1 | 0.612582525 | 0.000882505 | Down |
|  | A0A6G2G671 | *scpB* | Methylmalonyl-CoA decarboxylase OS = *Escherichia coli* OX = 562 GN = scpB PE = 3 SV = 1 | 0.610064167 | 0.004760609 | Down |
|  | A0A4V4SBS9 | *tpiA* | Triosephosphate isomerase OS = *Escherichia coli* OX = 562 GN = tpiA PE = 3 SV = 1 | 0.609567286 | 0.032212814 | Down |
|  | A0A8A9FI50 | *gatZ* | Tagatose-bisphosphate aldolase subunit GatZ OS = *Escherichia coli* OX = 562 GN = gatZ PE = 4 SV = 1 | 0.609117413 | 0.000681612 | Down |
|  | A0A376I3P5 | *ushA* | Protein UshA [includes : UDP-sugar hydrolase 5'-nucleotidase] OS = *Escherichia coli* OX = 562 GN = ushA PE = 3 SV = 1 | 0.608886674 | 0.017055944 | Down |
|  | A0A7U1SA07 | JNN41_00770 | Head_binding domain-containing protein OS = *Escherichia coli* OX = 562 GN = JNN41_00770 PE = 4 SV = 1 | 0.60649998 | 0.005975106 | Down |
|  | W1VWC7 | Q609_ECAC02901G0001 | Uncharacterized protein (Fragment) OS = *Escherichia coli* DORA_A_5_14_21 OX = 1403943 GN = Q609_ECAC02901G0001 PE = 4 SV = 1 | 0.604572961 | 0.001703234 | Down |
|  | B7MEE9 | *gatA* | Galactitol-specific enzyme IIA component of PTS OS = *Escherichia coli* O45 : K1 (strain S88 / ExPEC) OX = 585035 GN = gatA PE = 4 SV = 1 | 0.602969176 | 0.00065998 | Down |
|  | A0A377FHP1 | *yhbT* | Ubiquinone biosynthesis accessory factor UbiT OS = *Escherichia coli* OX = 562 GN = yhbT PE = 3 SV = 1 | 0.600108729 | 0.03853302 | Down |
|  | P06149 | *dld* | Quinone-dependent D-lactate dehydrogenase OS = *Escherichia coli* (strain K12) OX = 83333 GN = dld PE = 1 SV = 3 | 0.599056484 | 0.000533947 | Down |
|  | E6BPP9 | *proP* | Proline porter II OS = *Escherichia coli* MS 85-1 OX = 679202 GN = proP PE = 3 SV = 1 | 0.599005011 | 0.003365726 | Down |
|  | A0A6C9TKU4 | *hycE* | Hydrogenase large subunit OS = *Escherichia coli* OX = 562 GN = hycE PE = 4 SV = 1 | 0.598622226 | 0.016083171 | Down |
|  | P52129 | *rnlA* | mRNA endoribonuclease toxin LS OS = *Escherichia coli* (strain K12) OX = 83333 GN = rnlA PE = 1 SV = 2 | 0.597465878 | 0.001202227 | Down |
|  | A0A0P0SWP2 | *gatZ* | D-tagatose-1,6-bisphosphate aldolase subunit GatZ OS = *Escherichia coli* OX = 562 GN = gatZ PE = 3 SV = 1 | 0.595354278 | 0.001455453 | Down |
|  | A0A1X3JHH2 | *uxaB* | Altronate oxidoreductase OS = *Escherichia coli* H386 OX = 656397 GN = uxaB PE = 3 SV = 1 | 0.595319182 | 0.022574269 | Down |
|  | A0A376TD77 | *gatZ_3* | Putative tagatose 6-phosphate kinase OS = *Escherichia coli* OX = 562 GN = gatZ_3 PE = 4 SV = 1 | 0.595311865 | 3.50209E-05 | Down |
|  | A0A843M746 | *tdcB* | Bifunctional threonine ammonia-lyase/L-serine ammonia-lyase TdcB OS = *Escherichia coli* OX = 562 GN = tdcB PE = 4 SV = 1 | 0.592502912 | 0.035291341 | Down |
|  | V0TDJ1 | HMPREF1595_01611 | Glycine betaine/L-proline transport system permease protein ProW OS = *Escherichia coli* 907672 OX = 1268982 GN = HMPREF1595_01611 PE = 3 SV = 1 | 0.590291351 | 1.13472E-05 | Down |
|  | A0A843NGY2 | *fghA* | S-formylglutathione hydrolase OS = *Escherichia coli* OX = 562 GN = fghA PE = 4 SV = 1 | 0.589342689 | 0.000612265 | Down |
|  | A0A368IN84 | A6592_18150 | Peptidyl-prolyl cis-trans isomerase OS = *Escherichia coli* OX = 562 GN = A6592_18150 PE = 3 SV = 1 | 0.58650441 | 0.001775239 | Down |
|  | A0A826YKV9 | FA849_18875 | Formate dehydrogenase subunit alpha OS = *Escherichia coli* OX = 562 GN = FA849_18875 PE = 4 SV = 1 | 0.585705851 | 0.023703639 | Down |
|  | A0A6L6RZ23 | *gatZ* | D-tagatose-1,6-bisphosphate aldolase subunit GatZ OS = *Escherichia coli* OX = 562 GN = gatZ PE = 3 SV = 1 | 0.577246091 | 0.000479455 | Down |
|  | A0A6D0HB74 | *chbB* | PTS N,N'-diacetylchitobiose transporter subunit IIB OS = *Escherichia coli* OX = 562 GN = chbB PE = 4 SV = 1 | 0.570935375 | 0.00440244 | Down |
|  | A0A5P0JCN2 | *gatZ* | D-tagatose-1,6-bisphosphate aldolase subunit GatZ OS = *Escherichia coli* OX = 562 GN = gatZ PE = 3 SV = 1 | 0.569107265 | 0.000375893 | Down |
|  | A0A376VYA0 | *tdcF* | TdcF protein OS = *Escherichia coli* OX = 562 GN = tdcF PE = 3 SV = 1 | 0.567592085 | 0.00267654 | Down |
|  | C0ST84 | *gatA* | Galactitol-specific enzyme IIA component of PTS OS = *Escherichia coli* O55 : H7 OX = 244320 GN = gatA PE = 4 SV = 1 | 0.565977851 | 0.037830108 | Down |
|  | A0A6C9ILX1 | GKF86_29565 | Betaine/proline/choline family ABC transporter ATP-binding protein (Fragment) OS = *Escherichia coli* OX = 562 GN = GKF86_29565 PE = 4 SV = 1 | 0.563807535 | 0.04898278 | Down |
|  | A0A641JB40 | *gutM* | Transcriptional regulator GutM OS = *Escherichia coli* OX = 562 GN = gutM PE = 4 SV = 1 | 0.561887414 | 0.014221974 | Down |
|  | A0A8B3MB75 | CIG67_04390 | Uncharacterized protein OS = *Escherichia coli* OX = 562 GN = CIG67_04390 PE = 4 SV = 1 | 0.560922983 | 0.001633563 | Down |
|  | A0A7A3AF22 | HJ940_004371 | YfeK family protein OS = *Escherichia coli* OX = 562 GN = HJ940_004371 PE = 4 SV = 1 | 0.559594042 | 0.034497319 | Down |
|  | A0A376I7H5 | *mgsA* | Methylglyoxal synthase OS = *Escherichia coli* OX = 562 GN = mgsA PE = 3 SV = 1 | 0.553843556 | 0.000309571 | Down |
|  | P0AB15 | *yccJ* | Uncharacterized protein YccJ OS = *Escherichia coli* O6 : H1 (strain CFT073 / ATCC 700928 / UPEC) OX = 199310 GN = yccJ PE = 4 SV = 1 | 0.553279975 | 0.000464928 | Down |
|  | A0A6M1HJW3 | G5632_09585 | Aldehyde ferredoxin oxidoreductase OS = *Escherichia coli* OX = 562 GN = G5632_09585 PE = 3 SV = 1 | 0.542699996 | 0.001387422 | Down |
|  | A0A6D0H698 | *rplX* | 50S ribosomal protein L24 OS = *Escherichia coli* OX = 562 GN = rplX PE = 3 SV = 1 | 0.540468485 | 0.017736553 | Down |
|  | A0A8A5ITE9 | *yieF* | Class I chromate reductase YieF OS = *Escherichia coli* H20 OX = 2810409 GN = yieF PE = 4 SV = 1 | 0.538400509 | 1.89822E-05 | Down |
|  | P0AD05 | *yecA* | Uncharacterized protein YecA OS = *Escherichia coli* (strain K12) OX = 83333 GN = yecA PE = 4 SV = 1 | 0.536212732 | 0.000293759 | Down |
|  | A0A826TWS8 | FQF29_10515 | Molybdopterin-dependent oxidoreductase FAD-binding subunit OS = *Escherichia coli* OX = 562 GN = FQF29_10515 PE = 4 SV = 1 | 0.535608158 | 0.011011125 | Down |
|  | A0A829IMX9 | G686_02181 | D-tagatose-1,6-bisphosphate aldolase subunit gatZ OS = *Escherichia coli* HVH 6 (3-8296502) OX = 1280961 GN = G686_02181 PE = 4 SV = 1 | 0.531118747 | 0.003220336 | Down |
|  | A0A4Y8FPW6 | BON92_27575 | Cold-shock protein OS = *Escherichia coli* OX = 562 GN = BON92_27575 PE = 4 SV = 1 | 0.530871679 | 0.001758459 | Down |
|  | A0A7L5VD13 | *galP* | Galactose/proton symporter OS = *Escherichia coli* OX = 562 GN = galP PE = 3 SV = 1 | 0.529706607 | 0.019610836 | Down |
|  | P06960 | *argF* | Ornithine carbamoyltransferase subunit F OS = *Escherichia coli* (strain K12) OX = 83333 GN = argF PE = 1 SV = 4 | 0.521148722 | 0.001372844 | Down |
|  | A0A6D0H6A5 | *gatZ* | D-tagatose-1,6-bisphosphate aldolase subunit GatZ OS = *Escherichia coli* OX = 562 GN = gatZ PE = 3 SV = 1 | 0.519526012 | 0.013031751 | Down |
|  | A0A6L4XP60 | *glnH* | Glutamine ABC transporter substrate-binding protein GlnH OS = *Escherichia coli* OX = 562 GN = glnH PE = 3 SV = 1 | 0.518180832 | 0.00016322 | Down |
|  | A0A6L4XIV1 | *uxaB* | Altronate oxidoreductase OS = *Escherichia coli* OX = 562 GN = uxaB PE = 3 SV = 1 | 0.512449464 | 0.044198844 | Down |
|  | S1F7L1 | A1UI_01612 | Oxygen sensor protein DosP OS = *Escherichia coli* KTE73 OX = 1182680 GN = A1UI_01612 PE = 4 SV = 1 | 0.507655796 | 0.013247294 | Down |
|  | A0A5P0JHD6 | *inaA* | Lipopolysaccharide kinase InaA (Fragment) OS = *Escherichia coli* OX = 562 GN = inaA PE = 4 SV = 1 | 0.505877726 | 0.002317598 | Down |
|  | A0A0A0FJG0 | EL76_1121 | Formate hydrogenlyase subunit 6 OS = *Escherichia coli* G3/10 OX = 1455601 GN = EL76_1121 PE = 4 SV = 1 | 0.497314741 | 0.040011842 | Down |
|  | B6I140 | *ybeD* | UPF0250 protein YbeD OS = *Escherichia coli* (strain SE11) OX = 409438 GN = ybeD PE = 3 SV = 1 | 0.494831582 | 0.000801738 | Down |
|  | A0A827EK03 | *tdcD* | Propionate kinase OS = *Escherichia coli* OX = 562 GN = tdcD PE = 4 SV = 1 | 0.493248966 | 0.010669873 | Down |
|  | A0A3L2Q016 | D9J03_02630 | Tetratricopeptide repeat-containing protein OS = *Escherichia coli* OX = 562 GN = D9J03_02630 PE = 3 SV = 1 | 0.487501718 | 0.003408676 | Down |
|  | A0A6M0PUX4 | G4V03_05755 | Replication endonuclease OS = *Escherichia coli* OX = 562 GN = G4V03_05755 PE = 3 SV = 1 | 0.483111791 | 0.009941949 | Down |
|  | P26608 | *fliS* | Flagellar secretion chaperone FliS OS = *Escherichia coli* (strain K12) OX = 83333 GN = fliS PE = 3 SV = 1 | 0.478548536 | 0.002116251 | Down |
|  | A0A641J6U1 | *gatZ* | D-tagatose-1,6-bisphosphate aldolase subunit GatZ OS = *Escherichia coli* OX = 562 GN = gatZ PE = 3 SV = 1 | 0.473816493 | 2.66902E-05 | Down |
|  | A0A2X5F0L7 | *rpsV* | 30S ribosomal protein S22 OS = *Escherichia coli* OX = 562 GN = rpsV PE = 3 SV = 1 | 0.47272587 | 0.008035959 | Down |
|  | A0A855FRH0 | CT143_09980 | Formate dehydrogenase OS = *Escherichia coli* OX = 562 GN = CT143_09980 PE = 4 SV = 1 | 0.467748248 | 0.012077841 | Down |
|  | A0A1Y2XL69 | AW059_18480 | Uncharacterized protein OS = *Escherichia coli* OX = 562 GN = AW059_18480 PE = 4 SV = 1 | 0.458573426 | 0.001611927 | Down |
|  | A0A479JTS0 | *gatA* | Galactitol-specific enzyme IIA component of PTS OS = *Escherichia coli* OX = 562 GN = gatA PE = 4 SV = 1 | 0.45820574 | 3.4234E-06 | Down |
|  | A0A8A5HPJ3 | JSU13_08840 | Uncharacterized protein OS = *Escherichia coli* O89m : H9 OX = 2810406 GN = JSU13_08840 PE = 4 SV = 1 | 0.433514813 | 0.008189811 | Down |
|  | A0A7B4LXL2 | HMV41_29505 | NAD(P)H-dependent oxidoreductase OS = *Escherichia coli* OX = 562 GN = HMV41_29505 PE = 4 SV = 1 | 0.424562933 | 0.000329577 | Down |
|  | A0A5B9AHZ7 | *gatZ* | D-tagatose-1,6-bisphosphate aldolase subunit GatZ OS = *Escherichia coli* OX = 562 GN = gatZ PE = 3 SV = 1 | 0.421971338 | 0.000477144 | Down |
|  | A0A2X1K1Q5 | *gatZ* | D-tagatose-1,6-bisphosphate aldolase subunit GatZ OS = *Escherichia coli* OX = 562 GN = gatZ PE = 3 SV = 1 | 0.418376 | 0.003987273 | Down |
|  | A0A775TXC2 | GRC90_26205 | Type 1 fimbrial protein (Fragment) OS = *Escherichia coli* OX = 562 GN = GRC90_26205 PE = 4 SV = 1 | 0.403941676 | 0.001240819 | Down |
|  | A0A1X3I1D3 | EAMG_01047 | Glycine betaine-binding periplasmic protein OS = *Escherichia coli* M056 OX = 656415 GN = EAMG_01047 PE = 4 SV = 1 | 0.40353363 | 0.012109656 | Down |
|  | A0A377FAX0 | *flgD_1* | Basal-body rod modification protein FlgD OS = *Escherichia coli* OX = 562 GN = flgD_1 PE = 3 SV = 1 | 0.391643955 | 0.001493479 | Down |
|  | A0A5F1T6E1 | DAH34_09615 | Uncharacterized protein OS = *Escherichia coli* OX = 562 GN = DAH34_09615 PE = 4 SV = 1 | 0.379839439 | 0.002094179 | Down |
|  | P0AB34 | *bssS* | Biofilm regulator BssS OS = *Escherichia coli* O6 : H1 (strain CFT073 / ATCC 700928 / UPEC) OX = 199310 GN = bssS PE = 3 SV = 1 | 0.377984749 | 4.15798E-05 | Down |
|  | A0A376M3X3 | *hycA* | Formate hydrogenlyase regulatory protein OS = *Escherichia coli* OX = 562 GN = hycA PE = 4 SV = 1 | 0.371764177 | 0.035833766 | Down |
|  | A0A827K3H5 | *proX* | Glycine betaine/L-proline ABC transporter substrate-binding protein ProX OS = *Escherichia coli* OX = 562 GN = proX PE = 4 SV = 1 | 0.363668742 | 0.047735585 | Down |
|  | A0A2X3JZ76 | *smg* | Protein Smg OS = *Escherichia coli* OX = 562 GN = smg PE = 3 SV = 1 | 0.353883271 | 0.002122652 | Down |
|  | A0A376HX34 | *yhgF* | Putative transcription accessory protein OS = *Escherichia coli* OX = 562 GN = yhgF PE = 4 SV = 1 | 0.321733139 | 0.001486645 | Down |
|  | A0A6L4XKW1 | GP710_07770 | Cold-shock protein OS = *Escherichia coli* OX = 562 GN = GP710_07770 PE = 4 SV = 1 | 0.256108114 | 0.005514668 | Down |
|  | A0A837Y776 | AML23_24525 | Cold-shock protein OS = *Escherichia coli* OX = 562 GN = AML23_24525 PE = 4 SV = 1 | 0.250383142 | 0.001021351 | Down |
|  | A0A1U9SKI6 | A8C65_23405 | Cold shock protein YdfK OS = *Escherichia coli* OX = 562 GN = A8C65_23405 PE = 4 SV = 1 | 0.22621039 | 0.002323117 | Down |
|  | P76073 | *ynaE* | Uncharacterized protein YnaE OS = *Escherichia coli* (strain K12) OX = 83333 GN = ynaE PE = 2 SV = 2 | 0.171298151 | 0.002408123 | Down |
|  | P0A9Y0 | *cspA* | Cold shock protein CspA OS = *Escherichia coli* O6 : H1 (strain CFT073 / ATCC 700928 / UPEC) OX = 199310 GN = cspA PE = 3 SV = 2 | 0.157734672 | 0.000227476 | Down |
|  | A0A6M0PSK1 | *cspA* | RNA chaperone/antiterminator CspA OS = *Escherichia coli* OX = 562 GN = cspA PE = 4 SV = 1 | 0.113921493 | 0.000256227 | Down |
| S2-2 | A0A8B4PN44 | *tnaA* | Tryptophanase OS = *Escherichia coli* OX = 562 GN = tnaA PE = 4 SV = 1 | 5.343234575 | 0.003829015 | Up |
|  | A0A771BBG3 | *tnaA* | Tryptophanase OS = *Escherichia coli* OX = 562 GN = tnaA PE = 4 SV = 1 | 3.733164165 | 0.049834461 | Up |
|  | A0A2X7H313 | *tnaA* | Tryptophanase OS = *Escherichia coli* OX = 562 GN = tnaA PE = 3 SV = 1 | 3.732628127 | 0.002899254 | Up |
|  | A0A7A3AYV6 | *hemX* | Uroporphyrinogen-III C-methyltransferase OS = *Escherichia coli* OX = 562 GN = hemX PE = 4 SV = 1 | 2.796054057 | 0.00604086 | Up |
|  | A0A418U310 | *tnaA* | Tryptophanase OS = *Escherichia coli* OX = 562 GN = tnaA PE = 3 SV = 1 | 2.739383665 | 0.000632746 | Up |
|  | A0A6C9IM12 | GKF86_27255 | SAM-dependent methyltransferase OS = *Escherichia coli* OX = 562 GN = GKF86_27255 PE = 4 SV = 1 | 2.707906574 | 0.000337911 | Up |
|  | A0A2A3WTJ2 | *hdeA* | Acid stress chaperone HdeA OS = *Escherichia coli* OX = 562 GN = hdeA PE = 3 SV = 1 | 2.703989172 | 0.031329139 | Up |
|  | A0A6M0PU76 | *rplI* | 50S ribosomal protein L9 OS = *Escherichia coli* OX = 562 GN = rplI PE = 3 SV = 1 | 2.671555335 | 0.002445514 | Up |
|  | U9XXA4 | *xseB* | Exodeoxyribonuclease 7 small subunit OS = *Escherichia coli* 113290 OX = 1268976 GN = xseB PE = 3 SV = 1 | 2.665297914 | 0.000878467 | Up |
|  | A0A829JIU5 | G938_01661 | Sulfatase YdeN OS = *Escherichia coli* UMEA 3200-1 OX = 1281213 GN = G938_01661 PE = 4 SV = 1 | 2.663570261 | 0.000405451 | Up |
|  | A0A7U9ASB9 | *tnaA* | Tryptophanase OS = *Escherichia coli* TA206 OX = 656440 GN = tnaA PE = 3 SV = 1 | 2.626087347 | 0.000647668 | Up |
|  | A0A376L5P7 | *ompW_1* | Outer membrane protein W OS = *Escherichia coli* OX = 562 GN = ompW_1 PE = 4 SV = 1 | 2.601003952 | 0.004136192 | Up |
|  | A0A376JKT8 | *wrbA_3* | Putative flavoprotein OS = *Escherichia coli* OX = 562 GN = wrbA_3 PE = 4 SV = 1 | 2.59908094 | 0.000998285 | Up |
|  | A0A7U9FZE8 | *tnaA* | Tryptophanase OS = *Escherichia coli* TA143 OX = 656437 GN = tnaA PE = 3 SV = 1 | 2.534609684 | 0.004073786 | Up |
|  | A0A7L5V678 | *glpT* | Glycerol-3-phosphate transporter OS = *Escherichia coli* OX = 562 GN = glpT PE = 3 SV = 1 | 2.497118729 | 0.001045121 | Up |
|  | Q8XCG1 | *flhB* | Flagellar biosynthetic protein FlhB OS = *Escherichia coli* O157 : H7 OX = 83334 GN = flhB PE = 3 SV = 2 | 2.399288016 | 0.012919243 | Up |
|  | A0A7T8PQW7 | *waaO* | Lipopolysaccharide 3-alpha-galactosyltransferase OS = *Escherichia coli* OX = 562 GN = waaO PE = 4 SV = 1 | 2.345944273 | 0.002510568 | Up |
|  | A0A7B3MKM9 | HLZ50_09340 | DUF892 family protein OS = *Escherichia coli* OX = 562 GN = HLZ50_09340 PE = 4 SV = 1 | 2.295755351 | 0.009628486 | Up |
|  | A0A826R875 | *envC* | Murein hydrolase activator EnvC OS = *Escherichia coli* OX = 562 GN = envC PE = 4 SV = 1 | 2.278541679 | 0.001213861 | Up |
|  | S1IP87 | A1WU_02908 | Uncharacterized protein OS = *Escherichia coli* KTE108 OX = 1182704 GN = A1WU_02908 PE = 4 SV = 1 | 2.218484947 | 0.040784709 | Up |
|  | A0A853RYC6 | BHF52_02880 | Tryptophanase OS = *Escherichia coli* OX = 562 GN = BHF52_02880 PE = 4 SV = 1 | 2.17740457 | 0.011135854 | Up |
|  | A0A2S8JZY6 | *asnA* | Aspartate--ammonia ligase OS = *Escherichia coli* OX = 562 GN = asnA PE = 3 SV = 1 | 2.099311905 | 0.000786 | Up |
|  | A0A376S463 | *tnaA* | Tryptophanase OS = *Escherichia coli* OX = 562 GN = tnaA PE = 4 SV = 1 | 2.069561351 | 0.006910497 | Up |
|  | A0A6D0IT61 | *nanC* | N-acetylneuraminic acid outer membrane channel NanC OS = *Escherichia coli* OX = 562 GN = nanC PE = 4 SV = 1 | 2.048446041 | 0.034523886 | Up |
|  | A0A777SAD9 | *tnaA* | Tryptophanase OS = *Escherichia coli* OX = 562 GN = tnaA PE = 3 SV = 1 | 2.042148921 | 0.001281427 | Up |
|  | A0A3Y1V1S8 | *galF* | Alpha-D-glucosyl-1-phosphate uridylyltransferase OS = *Escherichia coli* OX = 562 GN = galF PE = 3 SV = 1 | 2.037238149 | 0.001546765 | Up |
|  | A0A377CV50 | *mazE* | Antitoxin MazE OS = *Escherichia coli* OX = 562 GN = mazE PE = 4 SV = 1 | 2.036132846 | 0.033770366 | Up |
|  | A0A6N8PVJ3 | *ackA* | Acetate kinase OS = *Escherichia coli* OX = 562 GN = ackA PE = 3 SV = 1 | 2.025055364 | 0.001586078 | Up |
|  | A0A8A5J2H5 | JSU10_11650 | LysR family transcriptional regulator OS = *Escherichia coli* H20 OX = 2810409 GN = JSU10_11650 PE = 4 SV = 1 | 1.995937329 | 0.033129507 | Up |
|  | A0A7U9FZE7 | ECMG_00118 | Glutamate decarboxylase beta (GAD-beta) OS = *Escherichia coli* TA143 OX = 656437 GN = ECMG_00118 PE = 4 SV = 1 | 1.986167869 | 0.015666714 | Up |
|  | A0A7H9LNK0 | *cadA* | Lysine decarboxylase CadA OS = *Escherichia coli* OX = 562 GN = cadA PE = 3 SV = 1 | 1.976877161 | 0.003381021 | Up |
|  | A0A6M0PVJ7 | *pyrG* | CTP synthase OS = *Escherichia coli* OX = 562 GN = pyrG PE = 3 SV = 1 | 1.976292113 | 0.000415996 | Up |
|  | A0A6L9DL34 | FZC17_12545 | Pirin family protein OS = *Escherichia coli* OX = 562 GN = FZC17_12545 PE = 3 SV = 1 | 1.974845763 | 0.011321792 | Up |
|  | A0A271QYN7 | BIZ41_14720 | Glutamate synthase large subunit OS = *Escherichia coli* OX = 562 GN = BIZ41_14720 PE = 3 SV = 1 | 1.962275822 | 0.001783965 | Up |
|  | A0A7U2BDF2 | *ompW* | Outer membrane protein OmpW OS = *Escherichia coli* OX = 562 GN = ompW PE = 4 SV = 1 | 1.959307898 | 0.000680773 | Up |
|  | A0A377BSF6 | *ompW_1* | Outer membrane protein W OS = *Escherichia coli* OX = 562 GN = ompW_1 PE = 4 SV = 1 | 1.9468898 | 0.005299291 | Up |
|  | W9AMC1 | *tnaA* | Tryptophanase OS = *Escherichia coli* O25b : H4-ST131 OX = 941322 GN = tnaA PE = 3 SV = 1 | 1.94111087 | 0.016034755 | Up |
|  | A0A376J8Y2 | *tnaB* | Aromatic amino acid permease OS = *Escherichia coli* OX = 562 GN = tnaB PE = 3 SV = 1 | 1.92964522 | 0.004827432 | Up |
|  | A0A1X3LUN7 | EAZG_00482 | Citrate lyase alpha chain OS = *Escherichia coli* TA249 OX = 656441 GN = EAZG_00482 PE = 4 SV = 1 | 1.907131413 | 0.000282399 | Up |
|  | A0A826JV02 | *fadD* | Long-chain-fatty-acid--CoA ligase FadD OS = *Escherichia coli* OX = 562 GN = fadD PE = 4 SV = 1 | 1.867149654 | 0.00489364 | Up |
|  | A0A826TP87 | CX694_002946 | HlyD family secretion protein OS = *Escherichia coli* OX = 562 GN = CX694_002946 PE = 4 SV = 1 | 1.852246711 | 0.028053116 | Up |
|  | A8A6H0 | *tnaA* | Tryptophanase OS = *Escherichia coli* O9 : H4 (strain HS) OX = 331112 GN = tnaA PE = 3 SV = 1 | 1.842611799 | 0.002957386 | Up |
|  | A0A6N9MUA6 | *glpQ* | Glycerophosphodiester phosphodiesterase OS = *Escherichia coli* OX = 562 GN = glpQ PE = 4 SV = 1 | 1.807868123 | 0.009273841 | Up |
|  | A0A7L5V1W3 | *mscK* | Mechanosensitive channel MscK OS = *Escherichia coli* OX = 562 GN = mscK PE = 3 SV = 1 | 1.803462373 | 0.00616843 | Up |
|  | A0A855W463 | DB282_27280 | Protease (Fragment) OS = *Escherichia coli* OX = 562 GN = DB282_27280 PE = 4 SV = 1 | 1.803461639 | 0.012774094 | Up |
|  | A0A828FSI3 | *cybC* | Cytochrome b562 OS = *Escherichia coli* OX = 562 GN = cybC PE = 4 SV = 1 | 1.774341665 | 0.011365413 | Up |
|  | A0A6L4XMK4 | GP710_03455 | Fumarate hydratase class I OS = *Escherichia coli* OX = 562 GN = GP710_03455 PE = 3 SV = 1 | 1.763530063 | 0.001737478 | Up |
|  | A0A828JSI0 | EH88_005446 | Acetyltransferase OS = *Escherichia coli* OX = 562 GN = EH88_005446 PE = 4 SV = 1 | 1.748115779 | 0.000170541 | Up |
|  | A0A8B5PFJ8 | *tnaA* | Tryptophanase OS = *Escherichia coli* OX = 562 GN = tnaA PE = 4 SV = 1 | 1.72284616 | 0.007039594 | Up |
|  | A0A5D8QI45 | E0I42_29365 | N-acetylneuraminate epimerase (Fragment) OS = *Escherichia coli* OX = 562 GN = E0I42_29365 PE = 4 SV = 1 | 1.716521664 | 0.011709611 | Up |
|  | A0A0A1A5H6 | *rihC* | Non-specific ribonucleoside hydrolase RihC OS = *Escherichia coli* OX = 562 GN = rihC PE = 3 SV = 1 | 1.71615706 | 0.000513101 | Up |
|  | A0A6D0DK91 | *cydA* | Cytochrome ubiquinol oxidase subunit I OS = *Escherichia coli* OX = 562 GN = cydA PE = 3 SV = 1 | 1.707575946 | 0.03930825 | Up |
|  | A0A3W5XXF0 | *cydA* | Cytochrome bd-I ubiquinol oxidase subunit CydA OS = *Escherichia coli* OX = 562 GN = cydA PE = 3 SV = 1 | 1.704328694 | 0.011221904 | Up |
|  | A0A5B1FAQ8 | *glpT* | GlpT protein OS = *Escherichia coli* OX = 562 GN = glpT PE = 3 SV = 1 | 1.692684045 | 0.008279463 | Up |
|  | A0A2P6IUD5 | *rfaF* | ADP-heptose--LPS heptosyltransferase RfaF OS = *Escherichia coli* OX = 562 GN = rfaF PE = 4 SV = 1 | 1.685832962 | 0.007062557 | Up |
|  | A0A2A3VUL7 | *tnaA* | Tryptophanase OS = *Escherichia coli* OX = 562 GN = tnaA PE = 3 SV = 1 | 1.672545252 | 0.015160923 | Up |
|  | W1WA20 | Q609_ECAC02517G0001 | Uncharacterized protein (Fragment) OS = *Escherichia coli* DORA_A_5_14_21 OX = 1403943 GN = Q609_ECAC02517G0001 PE = 4 SV = 1 | 1.662465138 | 0.006972883 | Up |
|  | Q46774 | *glpQ* | Glycerophosphoryl diester phosphodiesterase (Fragment) OS = *Escherichia coli* OX = 562 GN = glpQ PE = 4 SV = 1 | 1.655430801 | 0.049580887 | Up |
|  | A0A2I6IG80 | *ompW* | Outer membrane protein OmpW OS = *Escherichia coli* OX = 562 GN = ompW PE = 4 SV = 1 | 1.654173018 | 0.031135485 | Up |
|  | A0A836N789 | AB05_4678 | Oxidoreductase, NAD-binding Rossmann fold family protein OS = *Escherichia coli* 2-460-02_S1_C1 OX = 1444044 GN = AB05_4678 PE = 4 SV = 1 | 1.646733906 | 0.013365414 | Up |
|  | A0A7I8Z834 | *glpQ* | GlpQ protein OS = *Escherichia coli* OX = 562 GN = glpQ PE = 4 SV = 1 | 1.64304101 | 0.00164032 | Up |
|  | A0A7U9QDI6 | *torS* | Histidine kinase OS = *Escherichia coli* O145 : H28 OX = 1078034 GN = torS PE = 4 SV = 1 | 1.642008895 | 0.008124071 | Up |
|  | A0A5D8QXC9 | *ompT* | Omptin family outer membrane protease OmpT (Fragment) OS = *Escherichia coli* OX = 562 GN = ompT PE = 4 SV = 1 | 1.64121702 | 0.00302477 | Up |
|  | A0A6D0C6T7 | *cadA* | Lysine decarboxylase CadA OS = *Escherichia coli* OX = 562 GN = cadA PE = 3 SV = 1 | 1.636333655 | 0.008317323 | Up |
|  | A0A376HT67 | *gor* | Glutathione reductase OS = *Escherichia coli* OX = 562 GN = gor PE = 3 SV = 1 | 1.63438183 | 0.005427948 | Up |
|  | A0A5B9GFD8 | unknow | Glycosyltransferase OS = *Escherichia coli* OX = 562 PE = 4 SV = 1 | 1.633965899 | 0.00294875 | Up |
|  | A0A8B5F2G2 | BON70_20110 | Protease OS = *Escherichia coli* OX = 562 GN = BON70_20110 PE = 4 SV = 1 | 1.633150169 | 0.028672379 | Up |
|  | A0A6D0FG95 | *flgM* | Anti-sigma-28 factor OS = *Escherichia coli* OX = 562 GN = flgM PE = 3 SV = 1 | 1.626445538 | 0.003038649 | Up |
|  | A0A660HE83 | A9X72_14540 | Uncharacterized protein OS = *Escherichia coli* OX = 562 GN = A9X72_14540 PE = 4 SV = 1 | 1.618810606 | 0.003450402 | Up |
|  | A0A6D0Y0N5 | G3M51_04305 | DUF4765 family protein OS = *Escherichia coli* OX = 562 GN = G3M51_04305 PE = 4 SV = 1 | 1.617875531 | 0.015995414 | Up |
|  | A0A827L415 | BRV34_001673 | NAD(P)/FAD-dependent oxidoreductase OS = *Escherichia coli* OX = 562 GN = BRV34_001673 PE = 4 SV = 1 | 1.603564277 | 0.000730793 | Up |
|  | A0A376I8B9 | *cydA* | Cytochrome d ubiquinol oxidase subunit 1 OS = *Escherichia coli* OX = 562 GN = cydA PE = 3 SV = 1 | 1.594408753 | 0.006509071 | Up |
|  | A0A2W6PAC2 | DNQ45_17115 | YhcH/YjgK/YiaL family protein OS = *Escherichia coli* OX = 562 GN = DNQ45_17115 PE = 4 SV = 1 | 1.593560791 | 0.035106223 | Up |
|  | A0A6N6YDL1 | *glpQ* | Glycerophosphodiester phosphodiesterase OS = *Escherichia coli* OX = 562 GN = glpQ PE = 4 SV = 1 | 1.586496419 | 0.008650972 | Up |
|  | F4SG82 | ECHG_02138 | Glycerophosphoryl diester phosphodiesterase GlpQ OS = *Escherichia coli* H736 OX = 656414 GN = ECHG_02138 PE = 4 SV = 1 | 1.583376305 | 0.006036756 | Up |
|  | A0A376HV96 | *nanT_2* | KpLE2 phage-like element transporter OS = *Escherichia coli* OX = 562 GN = nanT_2 PE = 4 SV = 1 | 1.582529561 | 0.04568804 | Up |
|  | A0A828GWE9 | AAG81_004059 | Omptin family outer membrane protease OS = *Escherichia coli* OX = 562 GN = AAG81_004059 PE = 4 SV = 1 | 1.582058819 | 0.044326406 | Up |
|  | A0A1X3I3W6 | *lysS* | Lysine--tRNA ligase OS = *Escherichia coli* M056 OX = 656415 GN = lysS PE = 3 SV = 1 | 1.569479648 | 0.005891081 | Up |
|  | A0A828L8H3 | *cydB* | Cytochrome d ubiquinol oxidase subunit II OS = *Escherichia coli* OX = 562 GN = cydB PE = 4 SV = 1 | 1.566582162 | 0.004905634 | Up |
|  | W1ETT4 | unknow | Shikimate kinase I OS = *Escherichia coli* ISC7 OX = 1432555 PE = 4 SV = 1 | 1.563455026 | 0.001384919 | Up |
|  | A0A6D0USM6 | *glpQ* | Glycerophosphodiester phosphodiesterase (Fragment) OS = *Escherichia coli* OX = 562 GN = glpQ PE = 4 SV = 1 | 1.561803722 | 6.38657E-05 | Up |
|  | Q5IIG4 | *aspC* | Aspartate amino transferase (Fragment) OS = *Escherichia coli* OX = 562 GN = aspC PE = 4 SV = 1 | 1.561172004 | 0.027636808 | Up |
|  | A0A418H5N2 | *ybtA* | Yersiniabactin transcriptional regulator YbtA (Fragment) OS = *Escherichia coli* OX = 562 GN = ybtA PE = 4 SV = 1 | 1.549264365 | 0.039845024 | Up |
|  | A0A831EYL4 | *yqhD* | Alcohol dehydrogenase OS = *Escherichia coli* OX = 562 GN = yqhD PE = 4 SV = 1 | 1.549050356 | 0.012472566 | Up |
|  | A0A5P0J560 | *uspF* | Universal stress protein UspF OS = *Escherichia coli* OX = 562 GN = uspF PE = 3 SV = 1 | 1.536432749 | 0.01072035 | Up |
|  | A0A6L4XL24 | *secA* | Protein translocase subunit SecA OS = *Escherichia coli* OX = 562 GN = secA PE = 2 SV = 1 | 1.534137067 | 0.011586731 | Up |
|  | A0A0K3PZ85 | *rmuC* | DNA recombination protein RmuC OS = *Escherichia coli* OX = 562 GN = rmuC PE = 3 SV = 1 | 1.53012661 | 0.000310334 | Up |
|  | A0A843ZG16 | E4K54_28035 | GTP-binding protein (Fragment) OS = *Escherichia coli* OX = 562 GN = E4K54_28035 PE = 4 SV = 1 | 1.528377922 | 0.049539709 | Up |
|  | K4XKM3 | *tnaA* | Tryptophanase/L-cysteine desulfhydrase, PLP-dependent OS = *Escherichia coli* O111 : H11 str. CVM9455 OX = 1165939 GN = tnaA PE = 4 SV = 1 | 1.525698199 | 0.007123976 | Up |
|  | A0A826XCX3 | *lysS* | Lysine--tRNA ligase OS = *Escherichia coli* OX = 562 GN = lysS PE = 4 SV = 1 | 1.524066368 | 0.011801903 | Up |
|  | A0A6C9LHP4 | *yedE* | Selenium metabolism membrane protein YedE/FdhT OS = *Escherichia coli* OX = 562 GN = yedE PE = 3 SV = 1 | 1.523822517 | 0.019808697 | Up |
|  | A0A844UUA4 | *galF* | UTP--glucose-1-phosphate uridylyltransferase GalF (Fragment) OS = *Escherichia coli* OX = 562 GN = galF PE = 4 SV = 1 | 1.52064641 | 0.008482077 | Up |
|  | A0A376S994 | *rmlB_1* | dTDP-D-glucose 4,6-dehydratase rmlB OS = *Escherichia coli* OX = 562 GN = rmlB_1 PE = 4 SV = 1 | 1.514825096 | 0.001211843 | Up |
|  | A0A376HZ91 | *mglB* | D-galactose-binding periplasmic protein OS = *Escherichia coli* OX = 562 GN = mglB PE = 3 SV = 1 | 1.514353288 | 0.006310704 | Up |
|  | D7Y8S5 | *fucO* | Lactaldehyde reductase OS = *Escherichia coli* (strain MS 115-1) OX = 749537 GN = fucO PE = 4 SV = 1 | 1.51418374 | 0.002024058 | Up |
|  | A0A1X3LME0 | EAXG_05135 | Protease 7 (Protease VII) (Omptin) (Outermembrane protein 3B) (Protease A) OS = *Escherichia coli* TA054 OX = 656433 GN = EAXG_05135 PE = 3 SV = 1 | 1.514177009 | 0.011814307 | Up |
|  | A0A6G4BZV9 | *tnaA* | Tryptophanase OS = *Escherichia coli* OX = 562 GN = tnaA PE = 3 SV = 1 | 1.513501942 | 0.001482245 | Up |
|  | A0A826SG38 | *glpD* | Glycerol-3-phosphate dehydrogenase OS = *Escherichia coli* OX = 562 GN = glpD PE = 4 SV = 1 | 1.512899283 | 0.035953899 | Up |
|  | A0A2T1LDM7 | *dsdA* | D-serine dehydratase OS = *Escherichia coli* OX = 562 GN = dsdA PE = 3 SV = 1 | 1.510859141 | 0.018742207 | Up |
|  | A0A1Q6BB69 | *hemL* | Glutamate-1-semialdehyde 2,1-aminomutase OS = *Escherichia coli* OX = 562 GN = hemL PE = 3 SV = 1 | 1.509173114 | 0.045811086 | Up |
|  | A0A827TFN0 | F9407_14020 | AAA family ATPase OS = *Escherichia coli* OX = 562 GN = F9407_14020 PE = 4 SV = 1 | 1.504083069 | 0.01598919 | Up |
|  | A0A826TR55 | *ivy* | C-lysozyme inhibitor OS = *Escherichia coli* OX = 562 GN = ivy PE = 4 SV = 1 | 1.500456409 | 0.002726161 | Up |
|  | D8AE21 | HMPREF9530_04824 | Uncharacterized protein OS = *Escherichia coli* (strain MS 21-1) OX = 749527 GN = HMPREF9530_04824 PE = 4 SV = 1 | 1.498959647 | 0.002631604 | Up |
|  | A0A7Z1F0N1 | APX88_03850 | Fumarate hydratase class I OS = *Escherichia coli* OX = 562 GN = APX88_03850 PE = 3 SV = 1 | 1.498206238 | 0.023010775 | Up |
|  | A0A843MJ33 | *nfuA* | Iron-sulfur cluster biogenesis protein NfuA OS = *Escherichia coli* OX = 562 GN = nfuA PE = 4 SV = 1 | 1.492993557 | 0.003380496 | Up |
|  | A0A6C9EEH0 | GKG27_26075 | Dipeptidase (Fragment) OS = *Escherichia coli* OX = 562 GN = GKG27_26075 PE = 3 SV = 1 | 1.491588997 | 0.02584438 | Up |
|  | A0A6N8PGB0 | FQ021_16075 | GntR family transcriptional regulator OS = *Escherichia coli* OX = 562 GN = FQ021_16075 PE = 4 SV = 1 | 1.490911656 | 0.002154427 | Up |
|  | A0A1X3LV10 | EAZG_00415 | Protease 7 (Protease VII) (Omptin) (Outermembrane protein 3B) (Protease A) OS = *Escherichia coli* TA249 OX = 656441 GN = EAZG_00415 PE = 3 SV = 1 | 1.488394168 | 0.033207585 | Up |
|  | A0A3L0VV29 | D9F05_05105 | Glycerophosphodiester phosphodiesterase OS = *Escherichia coli* OX = 562 GN = D9F05_05105 PE = 4 SV = 1 | 1.485809142 | 0.000513005 | Up |
|  | A0A6M0Q0B7 | *purC* | Phosphoribosylaminoimidazole-succinocarboxamide synthase OS = *Escherichia coli* OX = 562 GN = purC PE = 3 SV = 1 | 1.479065844 | 0.024679829 | Up |
|  | Q8XAW4 | Z2210 | Putative sulfatase OS = *Escherichia coli* O157 : H7 OX = 83334 GN = Z2210 PE = 3 SV = 2 | 1.478541914 | 0.000688705 | Up |
|  | A0A0E0XTU6 | *uxaC* | Uronate isomerase OS = *Escherichia coli* O104 : H4 (strain 2011C-3493) OX = 1133852 GN = uxaC PE = 3 SV = 1 | 1.475719208 | 0.044402657 | Up |
|  | A0A4T5JV04 | *mglB* | D-galactose-binding periplasmic protein OS = *Escherichia coli* OX = 562 GN = mglB PE = 3 SV = 1 | 1.472687976 | 0.002165104 | Up |
|  | A0A7H9QR77 | HVX31_00565 | Uncharacterized protein OS = *Escherichia coli* OX = 562 GN = HVX31_00565 PE = 4 SV = 1 | 1.471561151 | 0.019140922 | Up |
|  | A0A377E1X8 | *rcsF* | RcsF--phosphorelay glucose and zinc sensor OS = *Escherichia coli* OX = 562 GN = rcsF PE = 4 SV = 1 | 1.470974807 | 0.003224591 | Up |
|  | A0A3L2NTY7 | *uxaC* | Uronate isomerase OS = *Escherichia coli* OX = 562 GN = uxaC PE = 3 SV = 1 | 1.469211684 | 0.027549317 | Up |
|  | A0A376MVG2 | *phoQ* | Sensor histidine protein kinase/phosphatase PhoQ OS = *Escherichia coli* OX = 562 GN = phoQ PE = 4 SV = 1 | 1.469076762 | 0.002857484 | Up |
|  | A0A3Z8Z4S7 | BON95_20515 | Sensor protein OS = *Escherichia coli* OX = 562 GN = BON95_20515 PE = 4 SV = 1 | 1.466780253 | 0.02366767 | Up |
|  | A0A8A8NRS0 | *gntP* | Gluconate permease GntP OS = *Escherichia coli* OX = 562 GN = gntP PE = 4 SV = 1 | 1.464444931 | 0.001370823 | Up |
|  | A0A827QN65 | GQW07_19440 | Inhibitor of g-type lysozyme OS = *Escherichia coli* OX = 562 GN = GQW07_19440 PE = 4 SV = 1 | 1.464280876 | 0.002859237 | Up |
|  | A0A826ZD52 | *hdhA* | 7-alpha-hydroxysteroid dehydrogenase OS = *Escherichia coli* OX = 562 GN = hdhA PE = 4 SV = 1 | 1.46329038 | 6.93754E-06 | Up |
|  | J7QZG7 | *yjhT* | Uncharacterized protein (Fragment) OS = *Escherichia coli* OX = 562 GN = yjhT PE = 3 SV = 1 | 1.462755845 | 0.018711202 | Up |
|  | A0A2T3THJ9 | C7B02_06005 | Monooxygenase OS = *Escherichia coli* OX = 562 GN = C7B02_06005 PE = 4 SV = 1 | 1.459235964 | 0.00558034 | Up |
|  | A0A403LMR9 | *leuS* | Leucine--tRNA ligase OS = *Escherichia coli* OX = 562 GN = leuS PE = 3 SV = 1 | 1.457834882 | 0.00931084 | Up |
|  | A0A829K1L3 | H000_01269 | Uncharacterized protein OS = *Escherichia coli* UMEA 3899-1 OX = 1281275 GN = H000_01269 PE = 4 SV = 1 | 1.457711821 | 2.2581E-05 | Up |
|  | A0A646J549 | *mug* | G/U mismatch-specific DNA glycosylase OS = *Escherichia coli* OX = 562 GN = mug PE = 3 SV = 1 | 1.45709933 | 0.001658291 | Up |
|  | A0A777HIK4 | *glpT* | Glycerol-3-phosphate transporter OS = *Escherichia coli* OX = 562 GN = glpT PE = 3 SV = 1 | 1.450546298 | 0.014087378 | Up |
|  | A0A7H9QSS3 | HVX31_03840 | Oxidative stress defense protein OS = *Escherichia coli* OX = 562 GN = HVX31_03840 PE = 4 SV = 1 | 1.445208386 | 0.019761568 | Up |
|  | A0A828R0G1 | *mnmC* | UPF0209 protein yfcK OS = *Escherichia coli* 3431 OX = 670892 GN = mnmC PE = 4 SV = 1 | 1.443919678 | 0.009582024 | Up |
|  | A0A377BSB0 | *tnaA_1* | Tryptophanase OS = *Escherichia coli* OX = 562 GN = tnaA_1 PE = 4 SV = 1 | 1.443100239 | 0.00112502 | Up |
|  | A0A140NGD0 | ECBD_3794 | Alpha,alpha-phosphotrehalase OS = *Escherichia coli* (strain B / BL21-DE3) OX = 469008 GN = ECBD_3794 PE = 3 SV = 1 | 1.441784115 | 0.007247027 | Up |
|  | A0A376NYE6 | *nrfA* | Cytochrome c-552 OS = *Escherichia coli* OX = 562 GN = nrfA PE = 3 SV = 1 | 1.441697893 | 0.003812218 | Up |
|  | A0A828AVB4 | A8W81_002270 | Oxidative stress defense protein OS = *Escherichia coli* OX = 562 GN = A8W81_002270 PE = 4 SV = 1 | 1.439169114 | 0.017389484 | Up |
|  | A0A6N8Q227 | *yoaH* | UPF0181 protein YoaH OS = *Escherichia coli* OX = 562 GN = yoaH PE = 3 SV = 1 | 1.43863837 | 0.002429924 | Up |
|  | A0A3L0VY65 | *mglB* | D-galactose-binding periplasmic protein OS = *Escherichia coli* OX = 562 GN = mglB PE = 3 SV = 1 | 1.433284477 | 0.003987208 | Up |
|  | A0A2X7FCL6 | SAMEA3753300_01799 | Nucleotide di-P-sugar epimerase or dehydratase OS = *Escherichia coli* OX = 562 GN = SAMEA3753300_01799 PE = 4 SV = 1 | 1.431453817 | 0.03052699 | Up |
|  | A0A6N8Q653 | GRW77_31895 | YhcH/YjgK/YiaL family protein (Fragment) OS = *Escherichia coli* OX = 562 GN = GRW77_31895 PE = 4 SV = 1 | 1.429221923 | 0.04022472 | Up |
|  | A0A6N6X641 | *glpC* | Anaerobic glycerol-3-phosphate dehydrogenase subunit C (Fragment) OS = *Escherichia coli* OX = 562 GN = glpC PE = 4 SV = 1 | 1.429214531 | 0.018661055 | Up |
|  | A0A826NBH0 | CXJ73_004090 | Putative transporter OS = *Escherichia coli* OX = 562 GN = CXJ73_004090 PE = 4 SV = 1 | 1.428306771 | 0.005244823 | Up |
|  | A0A377DBF7 | *bfr* | Bacterioferritin OS = *Escherichia coli* OX = 562 GN = bfr PE = 3 SV = 1 | 1.426638433 | 9.05733E-05 | Up |
|  | A0A827LBS1 | *focA* | Formate transporter FocA OS = *Escherichia coli* OX = 562 GN = focA PE = 4 SV = 1 | 1.424163417 | 0.001329887 | Up |
|  | A0A376JDI4 | *cheA* | Chemotaxis protein CheA OS = *Escherichia coli* OX = 562 GN = cheA PE = 4 SV = 1 | 1.421130711 | 0.006396878 | Up |
|  | A0A377AU17 | *cbpA_2* | Chaperone modulatory protein CbpM OS = *Escherichia coli* OX = 562 GN = cbpA_2 PE = 3 SV = 1 | 1.418462233 | 0.009982358 | Up |
|  | A0A2A2BPI3 | BFL24_10370 | Sulfatase OS = *Escherichia coli* OX = 562 GN = BFL24_10370 PE = 3 SV = 1 | 1.415756989 | 0.001940924 | Up |
|  | A0A829CUZ6 | ECMP0215528_1535 | Outer membrane insertion C-terminal signal domain protein OS = *Escherichia coli* MP021552.8 OX = 1116133 GN = ECMP0215528_1535 PE = 4 SV = 1 | 1.414321913 | 0.00068572 | Up |
|  | A0A5C9AIS4 | FWK02_15270 | DUF406 domain-containing protein (Fragment) OS = *Escherichia coli* OX = 562 GN = FWK02_15270 PE = 3 SV = 1 | 1.412969836 | 0.003289636 | Up |
|  | A0A827IXR6 | GIB53_25780 | IS1 family transposase OS = *Escherichia coli* OX = 562 GN = GIB53_25780 PE = 4 SV = 1 | 1.409319233 | 0.009496841 | Up |
|  | A0A2U2VLW9 | *glpQ* | Glycerophosphodiester phosphodiesterase OS = *Escherichia coli* OX = 562 GN = glpQ PE = 4 SV = 1 | 1.408643373 | 0.025254528 | Up |
|  | Q1R585 | *yhjA* | Probable cytochrome C peroxidase OS = *Escherichia coli* (strain UTI89 / UPEC) OX = 364106 GN = yhjA PE = 4 SV = 1 | 1.406883485 | 0.032087764 | Up |
|  | A0A2X7FW49 | SAMEA3753300_00011 | DNA injection protein OS = *Escherichia coli* OX = 562 GN = SAMEA3753300_00011 PE = 4 SV = 1 | 1.40156952 | 0.004016541 | Up |
|  | I2UGI9 | EC40522_5562 | tRNA ligases class II (D, K and N) OS = *Escherichia coli* 4.0522 OX = 869681 GN = EC40522_5562 PE = 4 SV = 1 | 1.399896499 | 0.007541956 | Up |
|  | A0A369DD84 | *fdnG* | Formate dehydrogenase OS = *Escherichia coli* OX = 562 GN = fdnG PE = 3 SV = 1 | 1.397992604 | 0.019358812 | Up |
|  | A0A7I8Z9P3 | ETECE36_02358 | Cytochrome c-type biogenesis protein OS = *Escherichia coli* OX = 562 GN = ETECE36_02358 PE = 3 SV = 1 | 1.397581956 | 0.001547987 | Up |
|  | A0A6N8PP51 | GRW77_00150 | 2-amino-thiazoline-4-carboxylic acid hydrolase OS = *Escherichia coli* OX = 562 GN = GRW77_00150 PE = 4 SV = 1 | 1.397503753 | 0.026266362 | Up |
|  | A0A377CFY3 | *hchA* | Protein/nucleic acid deglycase HchA OS = *Escherichia coli* OX = 562 GN = hchA PE = 2 SV = 1 | 1.396929069 | 0.016831616 | Up |
|  | A0A376I976 | *yajQ* | UPF0234 protein YajQ OS = *Escherichia coli* OX = 562 GN = yajQ PE = 3 SV = 1 | 1.39672489 | 0.024634502 | Up |
|  | A0A828S740 | ECSTEC7V_3840 | Enhancing lycopene biosynthesis protein 2 OS = *Escherichia coli* STEC_7v OX = 754082 GN = ECSTEC7V_3840 PE = 4 SV = 1 | 1.390252781 | 0.003206067 | Up |
|  | A0A771MQY9 | *kbl* | 2-amino-3-ketobutyrate coenzyme A ligase OS = *Escherichia coli* OX = 562 GN = kbl PE = 3 SV = 1 | 1.389457082 | 0.01848828 | Up |
|  | A0A6M7H4E4 | *nanK* | N-acetylmannosamine kinase OS = *Escherichia coli* O157 : H7 OX = 83334 GN = nanK PE = 3 SV = 1 | 1.389251294 | 0.002897376 | Up |
|  | A0A6N8NDP3 | *grcA* | Autonomous glycyl radical cofactor OS = *Escherichia coli* OX = 562 GN = grcA PE = 3 SV = 1 | 1.388843402 | 0.011368602 | Up |
|  | P37194 | *slp* | Outer membrane protein Slp OS = *Escherichia coli* (strain K12) OX = 83333 GN = slp PE = 1 SV = 1 | 1.38496701 | 0.016221589 | Up |
|  | A0A0D3QSV6 | *ompC* | Outer membrane protein C (Fragment) OS = *Escherichia coli* OX = 562 GN = ompC PE = 3 SV = 1 | 1.384159284 | 0.016589323 | Up |
|  | A0A7T2N4B4 | *fliA* | RNA polymerase sigma factor FliA OS = *Escherichia coli* OX = 562 GN = fliA PE = 4 SV = 1 | 1.383246927 | 0.000329172 | Up |
|  | A0A827L354 | *lysA* | Diaminopimelate decarboxylase OS = *Escherichia coli* OX = 562 GN = lysA PE = 4 SV = 1 | 1.383061171 | 0.01127567 | Up |
|  | A0A377L8B3 | *livF_1* | Leucine/isoleucine/valine transporter ATP-binding subunit OS = *Escherichia coli* OX = 562 GN = livF_1 PE = 4 SV = 1 | 1.382911582 | 0.02043426 | Up |
|  | A0A0V9G8Z5 | *nanM* | N-acetylneuraminate epimerase OS = *Escherichia coli* OX = 562 GN = nanM PE = 3 SV = 1 | 1.3812316 | 0.031795232 | Up |
|  | A0A827LJ87 | *yqhD* | Alcohol dehydrogenase OS = *Escherichia coli* OX = 562 GN = yqhD PE = 4 SV = 1 | 1.380884161 | 0.019692254 | Up |
|  | A0A828UQB5 | *lysA* | Diaminopimelate decarboxylase OS = *Escherichia coli* 3.2608 OX = 869679 GN = lysA PE = 4 SV = 1 | 1.380118326 | 0.02109129 | Up |
|  | A0A6N8K9S4 | *ribD* | 5-amino-6-(5-phosphoribosylamino)uracil reductase (Fragment) OS = *Escherichia coli* OX = 562 GN = ribD PE = 4 SV = 1 | 1.379603391 | 0.023960146 | Up |
|  | A0A789R943 | *kdsD* | Arabinose 5-phosphate isomerase OS = *Escherichia coli* OX = 562 GN = kdsD PE = 3 SV = 1 | 1.37923256 | 0.013083932 | Up |
|  | A0A827NRZ2 | *hdhA* | 7-alpha-hydroxysteroid dehydrogenase OS = *Escherichia coli* OX = 562 GN = hdhA PE = 4 SV = 1 | 1.378891897 | 0.00128079 | Up |
|  | A0A2X6RA66 | *bssR* | Biofilm formation regulator BssR OS = *Escherichia coli* OX = 562 GN = bssR PE = 4 SV = 1 | 1.376612139 | 0.000917144 | Up |
|  | A0A3U5UQ40 | *uspD* | Universal stress protein OS = *Escherichia coli* OX = 562 GN = uspD PE = 3 SV = 1 | 1.375355916 | 0.003074884 | Up |
|  | P0A844 | *tatE* | Probable Sec-independent protein translocase protein TatE OS = *Escherichia coli* O6 : H1 (strain CFT073 / ATCC 700928 / UPEC) OX = 199310 GN = tatE PE = 3 SV = 1 | 1.371874016 | 0.002913372 | Up |
|  | A0A376RLB5 | *artI* | Arginine ABC transporter, substrate-binding protein OS = *Escherichia coli* OX = 562 GN = artI PE = 3 SV = 1 | 1.370325867 | 0.011779429 | Up |
|  | P76549 | *yffR* | Uncharacterized protein YffR OS = *Escherichia coli* (strain K12) OX = 83333 GN = yffR PE = 3 SV = 1 | 1.369937787 | 0.003568951 | Up |
|  | A0A376I2K4 | *aspS* | Aspartate--tRNA ligase OS = *Escherichia coli* OX = 562 GN = aspS PE = 3 SV = 1 | 1.369678131 | 0.031874877 | Up |
|  | A0A8B5HP37 | *gpr* | L-glyceraldehyde 3-phosphate reductase OS = *Escherichia coli* OX = 562 GN = gpr PE = 4 SV = 1 | 1.367552466 | 0.026851256 | Up |
|  | S0F2G7 | *adk* | Adenylate kinase (Fragment) OS = *Escherichia coli* OX = 562 GN = adk PE = 3 SV = 1 | 1.362933614 | 0.021233151 | Up |
|  | A0A3Y3V6Q4 | GKF89_16485 | Uncharacterized protein OS = *Escherichia coli* OX = 562 GN = GKF89_16485 PE = 4 SV = 1 | 1.361005741 | 0.009183349 | Up |
|  | A0A0K3UQX2 | ERS085366_02842 | Fels-1 Propage domain-containing protein OS = *Escherichia coli* OX = 562 GN = ERS085366_02842 PE = 4 SV = 1 | 1.360567319 | 0.024321442 | Up |
|  | A0A2T1LIJ2 | C6985_13475 | Glycerophosphodiester phosphodiesterase OS = *Escherichia coli* OX = 562 GN = C6985_13475 PE = 4 SV = 1 | 1.357750594 | 0.001772862 | Up |
|  | A0A3K0JQE8 | D9J61_18285 | FAD-binding protein OS = *Escherichia coli* OX = 562 GN = D9J61_18285 PE = 4 SV = 1 | 1.357121948 | 5.95434E-05 | Up |
|  | A0A2T1LK09 | C6985_09105 | Mannitol dehydrogenase family protein OS = *Escherichia coli* OX = 562 GN = C6985_09105 PE = 4 SV = 1 | 1.351717114 | 0.020652357 | Up |
|  | A0A7A2X3S7 | *ivy* | C-lysozyme inhibitor OS = *Escherichia coli* OX = 562 GN = ivy PE = 4 SV = 1 | 1.351108683 | 0.008964714 | Up |
|  | A0A826XA79 | *plsB* | Glycerol-3-phosphate 1-O-acyltransferase PlsB OS = *Escherichia coli* OX = 562 GN = plsB PE = 4 SV = 1 | 1.351062137 | 0.007242867 | Up |
|  | A0A0L1C3K3 | *fumB* | Fumarate hydratase class I OS = *Escherichia coli* OX = 562 GN = fumB PE = 3 SV = 1 | 1.351041259 | 0.007347661 | Up |
|  | A0A827E0G2 | CX692_001784 | Tryptophanase OS = *Escherichia coli* OX = 562 GN = CX692_001784 PE = 4 SV = 1 | 1.35094852 | 0.017575524 | Up |
|  | E6BHM5 | *sbcD* | Nuclease SbcCD subunit D OS = *Escherichia coli* MS 85-1 OX = 679202 GN = sbcD PE = 3 SV = 1 | 1.343813615 | 0.000132882 | Up |
|  | A0A3R0VNX2 | *cbpA* | Curved DNA-binding protein OS = *Escherichia coli* OX = 562 GN = cbpA PE = 3 SV = 1 | 1.341983836 | 0.025720554 | Up |
|  | E6BDM7 | *dps* | DNA protection during starvation protein OS = *Escherichia coli* MS 85-1 OX = 679202 GN = dps PE = 3 SV = 1 | 1.340531275 | 0.019871763 | Up |
|  | A0A6N9MN81 | *lysS* | Lysine--tRNA ligase OS = *Escherichia coli* OX = 562 GN = lysS PE = 3 SV = 1 | 1.340019196 | 0.012457526 | Up |
|  | A0A854W4Y1 | COD50_08130 | Replication protein OS = *Escherichia coli* OX = 562 GN = COD50_08130 PE = 4 SV = 1 | 1.33741207 | 0.019841805 | Up |
|  | A0A843M827 | D9F92_10695 | Uncharacterized protein OS = *Escherichia coli* OX = 562 GN = D9F92_10695 PE = 4 SV = 1 | 1.337406247 | 0.006350628 | Up |
|  | A0A3L0W8Z5 | *pykF* | Pyruvate kinase OS = *Escherichia coli* OX = 562 GN = pykF PE = 3 SV = 1 | 1.336141443 | 0.013603952 | Up |
|  | A0A6M0PS89 | G4V03_04295 | Succinate dehydrogenase iron-sulfur subunit OS = *Escherichia coli* OX = 562 GN = G4V03_04295 PE = 3 SV = 1 | 1.335758704 | 0.001067744 | Up |
|  | A0A6M1J473 | G5696_15940 | Uncharacterized protein OS = *Escherichia coli* OX = 562 GN = G5696_15940 PE = 4 SV = 1 | 1.335733671 | 0.001469932 | Up |
|  | A0A417ZXJ2 | *uxaC* | Uronate isomerase OS = *Escherichia coli* OX = 562 GN = uxaC PE = 3 SV = 1 | 1.335567276 | 0.001808803 | Up |
|  | A0A6M0Q0X4 | G4V03_16410 | Fumarylacetoacetate hydrolase family protein OS = *Escherichia coli* OX = 562 GN = G4V03_16410 PE = 4 SV = 1 | 1.334728038 | 0.041262798 | Up |
|  | A0A4Q4HRJ7 | EWK56_27595 | Elongation factor Tu (Fragment) OS = *Escherichia coli* OX = 562 GN = EWK56_27595 PE = 4 SV = 1 | 1.332941645 | 0.029328852 | Up |
|  | A0A826XAA9 | *putP* | Sodium/proline symporter PutP OS = *Escherichia coli* OX = 562 GN = putP PE = 4 SV = 1 | 1.332500642 | 0.045109508 | Up |
|  | A0A376I7C2 | *ompN_2* | Porin OmpN OS = *Escherichia coli* OX = 562 GN = ompN_2 PE = 3 SV = 1 | 1.330815001 | 0.017075715 | Up |
|  | A0A6L4XER2 | GP710_20030 | HAMP domain-containing protein OS = *Escherichia coli* OX = 562 GN = GP710_20030 PE = 4 SV = 1 | 1.330518421 | 0.003421087 | Up |
|  | A0A7H9QZ14 | *yahK* | NADPH-dependent aldehyde reductase YahK OS = *Escherichia coli* OX = 562 GN = yahK PE = 3 SV = 1 | 1.330210097 | 0.015996048 | Up |
|  | A0A853WIQ8 | BJI68_03965 | D-mannonate oxidoreductase OS = *Escherichia coli* OX = 562 GN = BJI68_03965 PE = 4 SV = 1 | 1.329330315 | 0.025057486 | Up |
|  | A0A6M0PSQ9 | *aceB* | Malate synthase OS = *Escherichia coli* OX = 562 GN = aceB PE = 3 SV = 1 | 1.329180754 | 0.036669328 | Up |
|  | Q1RE43 | *cspD* | Cold shock-like protein CspD OS = *Escherichia coli* (strain UTI89 / UPEC) OX = 364106 GN = cspD PE = 4 SV = 1 | 1.32574093 | 0.000108718 | Up |
|  | A0A3L9H549 | *rpmG* | 50S ribosomal protein L33 (Fragment) OS = *Escherichia coli* OX = 562 GN = rpmG PE = 3 SV = 1 | 1.324786811 | 0.015640561 | Up |
|  | A0A6L4XNV3 | *dacC* | Serine-type D-Ala-D-Ala carboxypeptidase OS = *Escherichia coli* OX = 562 GN = dacC PE = 3 SV = 1 | 1.324188983 | 1.51669E-05 | Up |
|  | A0A6N8PZM9 | GRW77_14420 | YciK family oxidoreductase OS = *Escherichia coli* OX = 562 GN = GRW77_14420 PE = 4 SV = 1 | 1.320407281 | 0.015140101 | Up |
|  | A0A376UFH3 | *ftsB* | Cell division protein FtsB OS = *Escherichia coli* OX = 562 GN = ftsB PE = 3 SV = 1 | 1.32036384 | 0.013052158 | Up |
|  | A0A6M0PUC9 | *mglB* | D-galactose-binding periplasmic protein OS = *Escherichia coli* OX = 562 GN = mglB PE = 3 SV = 1 | 1.32031562 | 0.028796264 | Up |
|  | A0A080IHA6 | AC28_4007 | Uncharacterized protein OS = *Escherichia coli* 1-250-04_S3_C2 OX = 1444163 GN = AC28_4007 PE = 4 SV = 1 | 1.318968611 | 0.006485926 | Up |
|  | A0A6D0EIL2 | *nrdD* | Anaerobic ribonucleoside-triphosphate reductase (Fragment) OS = *Escherichia coli* OX = 562 GN = nrdD PE = 4 SV = 1 | 1.316807333 | 0.036352553 | Up |
|  | A0A826RS52 | FPS82_03185 | DUF2756 family protein OS = *Escherichia coli* OX = 562 GN = FPS82_03185 PE = 4 SV = 1 | 1.316287473 | 0.024738496 | Up |
|  | A0A6L4XM71 | *tdh* | L-threonine 3-dehydrogenase OS = *Escherichia coli* OX = 562 GN = tdh PE = 3 SV = 1 | 1.31521479 | 0.001873968 | Up |
|  | A0A826MHE4 | *ldtB* | L,D-transpeptidase OS = *Escherichia coli* OX = 562 GN = ldtB PE = 4 SV = 1 | 1.315159233 | 0.016371856 | Up |
|  | E6BGX8 | *glyA* | Serine hydroxymethyltransferase OS = *Escherichia coli* MS 85-1 OX = 679202 GN = glyA PE = 3 SV = 1 | 1.314469907 | 0.002229095 | Up |
|  | E9XR66 | ERFG_03563 | SASA domain-containing protein OS = *Escherichia coli* TW10509 OX = 656449 GN = ERFG_03563 PE = 4 SV = 1 | 1.312798258 | 0.035525516 | Up |
|  | A0A2X3K8E1 | *rpoC_5* | DNA-directed RNA polymerase OS = *Escherichia coli* OX = 562 GN = rpoC_5 PE = 4 SV = 1 | 1.312367884 | 0.001368999 | Up |
|  | A0A5D8RSR4 | E0I42_27095 | Lipoprotein (Fragment) OS = *Escherichia coli* OX = 562 GN = E0I42_27095 PE = 4 SV = 1 | 1.310015842 | 0.004838305 | Up |
|  | A0A826VXD8 | *dpiB* | Sensor histidine kinase DpiB OS = *Escherichia coli* OX = 562 GN = dpiB PE = 4 SV = 1 | 1.309455226 | 0.009210153 | Up |
|  | A0A826X2M8 | *cspD* | Cold shock-like protein CspD OS = *Escherichia coli* OX = 562 GN = cspD PE = 4 SV = 1 | 1.309165026 | 0.002486364 | Up |
|  | A0A822U513 | *yfgG* | Protein OS = *Escherichia coli* OX = 562 GN = yfgG PE = 4 SV = 1 | 1.308230689 | 0.017434423 | Up |
|  | A0A827E4E7 | A2F99_001537 | Anaerobic C4-dicarboxylate transporter OS = *Escherichia coli* OX = 562 GN = A2F99_001537 PE = 4 SV = 1 | 1.307361689 | 0.032882519 | Up |
|  | A0A377DEX1 | *dnaK_4* | Chaperone protein DnaK (Heat shock protein 70) (Heat shock 70 kDaprotein) (HSP70) OS = *Escherichia coli* OX = 562 GN = dnaK_4 PE = 4 SV = 1 | 1.306722155 | 0.005093719 | Up |
|  | A0A5B9B029 | FTV90_14225 | SecY/secA suppressor protein OS = *Escherichia coli* OX = 562 GN = FTV90_14225 PE = 4 SV = 1 | 1.306271801 | 0.000727153 | Up |
|  | A0A777RRA0 | GFY48_05910 | Aldo/keto reductase OS = *Escherichia coli* OX = 562 GN = GFY48_05910 PE = 4 SV = 1 | 1.304251226 | 0.003393407 | Up |
|  | A0A6D0J0L5 | GP946_18280 | EAL domain-containing protein OS = *Escherichia coli* OX = 562 GN = GP946_18280 PE = 4 SV = 1 | 1.303859415 | 0.005971092 | Up |
|  | A0A376KS23 | *ftn* | Ferritin OS = *Escherichia coli* OX = 562 GN = ftn PE = 3 SV = 1 | 1.302661276 | 0.001652037 | Up |
|  | A0A7D7I6K9 | *pmbA* | Metalloprotease PmbA OS = *Escherichia coli* OX = 562 GN = pmbA PE = 3 SV = 1 | 1.300733583 | 0.003729886 | Up |
|  | A0A827TRU7 | F9571_25225 | Elongation factor Tu OS = *Escherichia coli* OX = 562 GN = F9571_25225 PE = 4 SV = 1 | 1.298788576 | 0.00309944 | Up |
|  | P28904 | *treC* | Trehalose-6-phosphate hydrolase OS = *Escherichia coli* (strain K12) OX = 83333 GN = treC PE = 1 SV = 3 | 1.298649124 | 0.00062339 | Up |
|  | Q56UC7 | unknow | Host specificity protein (Fragment) OS = *Escherichia coli* OX = 562 PE = 4 SV = 1 | 1.297169942 | 0.043410015 | Up |
|  | A0A6D0IS19 | *nagK* | N-acetyl-D-glucosamine kinase OS = *Escherichia coli* OX = 562 GN = nagK PE = 3 SV = 1 | 1.294149373 | 0.007297879 | Up |
|  | A0A2X1JNR5 | *rihC* | Non-specific ribonucleoside hydrolase RihC OS = *Escherichia coli* OX = 562 GN = rihC PE = 3 SV = 1 | 1.293279022 | 0.006905994 | Up |
|  | A0A7H9LUK6 | *glpC* | Anaerobic glycerol-3-phosphate dehydrogenase subunit C OS = *Escherichia coli* OX = 562 GN = glpC PE = 4 SV = 1 | 1.291860504 | 0.044848338 | Up |
|  | A0A7I8HMX8 | blaCTX-M-157 | Extended-spectrum beta-lactamase OS = *Escherichia coli* OX = 562 GN = blaCTX-M-157 PE = 4 SV = 1 | 1.291831579 | 0.001872913 | Up |
|  | A0A447X8U7 | *argI_2* | Ornithine carbamoyltransferase OS = *Escherichia coli* OX = 562 GN = argI_2 PE = 3 SV = 1 | 1.291716132 | 0.037875116 | Up |
|  | A0A6M0PYN9 | *glpQ* | Glycerophosphodiester phosphodiesterase OS = *Escherichia coli* OX = 562 GN = glpQ PE = 4 SV = 1 | 1.291244492 | 0.011992084 | Up |
|  | A0A5B9AIC4 | FTV93_04135 | DUF1315 family protein OS = *Escherichia coli* OX = 562 GN = FTV93_04135 PE = 4 SV = 1 | 1.291117354 | 0.024276704 | Up |
|  | A0A789MAE1 | *katG* | Catalase-peroxidase OS = *Escherichia coli* OX = 562 GN = katG PE = 3 SV = 1 | 1.291089803 | 0.037242681 | Up |
|  | A0A5F1DU45 | *lpxP* | Lipid A biosynthesis palmitoleoyltransferase OS = *Escherichia coli* OX = 562 GN = lpxP PE = 3 SV = 1 | 1.290659351 | 0.044074223 | Up |
|  | A0A2X1KKJ6 | *ydgH_1* | Protein YdgH OS = *Escherichia coli* OX = 562 GN = ydgH_1 PE = 4 SV = 1 | 1.290562212 | 0.002075882 | Up |
|  | A0A0K3R520 | *tar* | Methyl-accepting chemotaxis protein II OS = *Escherichia coli* OX = 562 GN = tar PE = 4 SV = 1 | 1.287916995 | 0.005961974 | Up |
|  | U9Y7D9 | *nanE* | Putative N-acetylmannosamine-6-phosphate 2-epimerase OS = *Escherichia coli* 113290 OX = 1268976 GN = nanE PE = 3 SV = 1 | 1.287051158 | 0.035393128 | Up |
|  | D8AEF0 | HMPREF9530_04954 | Sugar-binding domain protein OS = *Escherichia coli* (strain MS 21-1) OX = 749527 GN = HMPREF9530_04954 PE = 4 SV = 1 | 1.287040251 | 0.000941167 | Up |
|  | A0A828UTS3 | *nrfA* | Formate-dependent cytochrome c nitrite reductase, c552 subunit OS = *Escherichia coli* 3.2608 OX = 869679 GN = nrfA PE = 4 SV = 1 | 1.286958606 | 0.008230161 | Up |
|  | A0A827NG99 | BMC34_001646 | Dimethyl sulfoxide reductase subunit A OS = *Escherichia coli* OX = 562 GN = BMC34_001646 PE = 4 SV = 1 | 1.286951743 | 0.044806953 | Up |
|  | A0A3L0WAU3 | *dnaK* | Chaperone protein DnaK OS = *Escherichia coli* OX = 562 GN = dnaK PE = 2 SV = 1 | 1.286801856 | 0.031859559 | Up |
|  | A0A854ADD1 | BMT50_17580 | Autonomous glycyl radical cofactor GrcA OS = *Escherichia coli* OX = 562 GN = BMT50_17580 PE = 4 SV = 1 | 1.286601359 | 0.045042977 | Up |
|  | A0A0B1LVL6 | PU06_29190 | Toxin YhaV (Fragment) OS = *Escherichia coli* OX = 562 GN = PU06_29190 PE = 4 SV = 1 | 1.286213876 | 0.003895176 | Up |
|  | A0A6D1D2E5 | G5595_24695 | Transcriptional repressor PurR (Fragment) OS = *Escherichia coli* OX = 562 GN = G5595_24695 PE = 4 SV = 1 | 1.285614477 | 0.017286442 | Up |
|  | A0A403LPP3 | DLX40_17495 | DeoR/GlpR transcriptional regulator OS = *Escherichia coli* OX = 562 GN = DLX40_17495 PE = 4 SV = 1 | 1.285168462 | 0.000742276 | Up |
|  | A0A8A8NQQ1 | *idnD* | L-idonate 5-dehydrogenase OS = *Escherichia coli* OX = 562 GN = idnD PE = 4 SV = 1 | 1.28408698 | 0.000553167 | Up |
|  | A0A140N3E6 | *glnE* | Bifunctional glutamine synthetase adenylyltransferase/adenylyl-removing enzyme OS = *Escherichia coli* (strain B / BL21-DE3) OX = 469008 GN = glnE PE = 3 SV = 1 | 1.283547957 | 0.006906399 | Up |
|  | A0A3L9GVF5 | *tatA* | Sec-independent protein translocase protein TatA OS = *Escherichia coli* OX = 562 GN = tatA PE = 3 SV = 1 | 1.283516121 | 0.001466869 | Up |
|  | A0A376VYA9 | *yaeP* | UPF0253 protein YaeP OS = *Escherichia coli* OX = 562 GN = yaeP PE = 3 SV = 1 | 1.282265948 | 0.003438272 | Up |
|  | A0A828Q7V1 | *rlhA* | 23S rRNA 5-hydroxycytidine C2501 synthase OS = *Escherichia coli* OX = 562 GN = rlhA PE = 4 SV = 1 | 1.28135865 | 0.028282465 | Up |
|  | A0A376HZX6 | *cobT* | Nicotinate-nucleotide--dimethylbenzimidazole phosphoribosyltransferase OS = *Escherichia coli* OX = 562 GN = cobT PE = 3 SV = 1 | 1.280586701 | 0.010303037 | Up |
|  | A0A826TZQ7 | FQF29_15680 | C-lysozyme inhibitor OS = *Escherichia coli* OX = 562 GN = FQF29_15680 PE = 4 SV = 1 | 1.280465186 | 0.024638733 | Up |
|  | A0A829GBE0 | WG3_02894 | Arginine ABC transporter permease ArtQ OS = *Escherichia coli* KTE36 OX = 1169353 GN = WG3_02894 PE = 4 SV = 1 | 1.280448412 | 0.002090791 | Up |
|  | A0A0A0F8R1 | *ycgR* | Flagellar brake protein YcgR OS = *Escherichia coli* G3/10 OX = 1455601 GN = ycgR PE = 3 SV = 1 | 1.279954382 | 0.00048036 | Up |
|  | U9ZL98 | HMPREF1620_04332 | Hexuronate transporter OS = *Escherichia coli* 909945-2 OX = 1269007 GN = HMPREF1620_04332 PE = 4 SV = 1 | 1.27986781 | 0.015444722 | Up |
|  | P76440 | *preT* | NAD-dependent dihydropyrimidine dehydrogenase subunit PreT OS = *Escherichia coli* (strain K12) OX = 83333 GN = preT PE = 1 SV = 1 | 1.279571582 | 0.01270719 | Up |
|  | A0A2J1D5L3 | *dnaK* | Molecular chaperone DnaK (Fragment) OS = *Escherichia coli* OX = 562 GN = dnaK PE = 4 SV = 1 | 1.278557319 | 0.047416919 | Up |
|  | A0A836Z8X9 | AB05_5555 | Antigen 43 (Fragment) OS = *Escherichia coli* 2-460-02_S1_C1 OX = 1444044 GN = AB05_5555 PE = 4 SV = 1 | 1.277408581 | 0.014136767 | Up |
|  | A0A6M0PS67 | *glnS* | Glutamine--tRNA ligase OS = *Escherichia coli* OX = 562 GN = glnS PE = 3 SV = 1 | 1.276707688 | 0.029971941 | Up |
|  | P0A952 | *speG* | Spermidine N(1)-acetyltransferase OS = *Escherichia coli* O157 : H7 OX = 83334 GN = speG PE = 3 SV = 2 | 1.276402746 | 0.004646043 | Up |
|  | A0A828NK69 | B6R12_005050 | Sulfatase-like hydrolase/transferase OS = *Escherichia coli* OX = 562 GN = B6R12_005050 PE = 4 SV = 1 | 1.275378555 | 0.043075791 | Up |
|  | A0A7H9LXR6 | *glcB* | Malate synthase G OS = *Escherichia coli* OX = 562 GN = glcB PE = 3 SV = 1 | 1.275078087 | 0.038989276 | Up |
|  | A0A3L5AFZ3 | AAS29_000798 | DUF1471 domain-containing protein OS = *Escherichia coli* OX = 562 GN = AAS29_000798 PE = 4 SV = 1 | 1.274460041 | 0.011140234 | Up |
|  | A0A2S5UB44 | *proY* | Proline-specific permease ProY OS = *Escherichia coli* OX = 562 GN = proY PE = 4 SV = 1 | 1.273899313 | 0.000777127 | Up |
|  | A0A2X1Q327 | *yjjK_4* | Putative ABC transporter ATP-binding protein YjjK OS = *Escherichia coli* OX = 562 GN = yjjK_4 PE = 4 SV = 1 | 1.272652643 | 0.032711117 | Up |
|  | F4T7V4 | *frdD* | Fumarate reductase subunit D OS = *Escherichia coli* M605 OX = 656417 GN = frdD PE = 3 SV = 1 | 1.272521104 | 0.000449794 | Up |
|  | A0A765T6L6 | *ompC* | Porin OmpC OS = *Escherichia coli* OX = 562 GN = ompC PE = 3 SV = 1 | 1.271547818 | 0.011623049 | Up |
|  | A0A377HG86 | *qseG_2* | Putative lipoprotein OS = *Escherichia coli* OX = 562 GN = qseG_2 PE = 4 SV = 1 | 1.270718818 | 0.002566424 | Up |
|  | A0A6N8PQU5 | *cysM* | Cysteine synthase OS = *Escherichia coli* OX = 562 GN = cysM PE = 3 SV = 1 | 1.269934289 | 0.012648067 | Up |
|  | A0A376HVR8 | NCTC9094_00744 | Putative adenylate cyclase OS = *Escherichia coli* OX = 562 GN = NCTC9094_00744 PE = 4 SV = 1 | 1.269310071 | 8.64736E-05 | Up |
|  | A0A6M0PU94 | *ytfE* | Iron-sulfur cluster repair protein YtfE OS = *Escherichia coli* OX = 562 GN = ytfE PE = 3 SV = 1 | 1.267496863 | 0.027189684 | Up |
|  | A0A7D7DU33 | *hchA* | Protein deglycase HchA OS = *Escherichia coli* OX = 562 GN = hchA PE = 4 SV = 1 | 1.26428784 | 0.003694589 | Up |
|  | A0A5B9AYS4 | FTV90_17080 | Molybdopterin-dependent oxidoreductase OS = *Escherichia coli* OX = 562 GN = FTV90_17080 PE = 4 SV = 1 | 1.263898731 | 0.022538287 | Up |
|  | A0A7D7PKU0 | *cpdB* | 2',3'-cyclic-nucleotide 2'-phosphodiesterase/3'-nucleotidase OS = *Escherichia coli* OX = 562 GN = cpdB PE = 3 SV = 1 | 1.263181984 | 0.004324635 | Up |
|  | A0A485JCI0 | *glpQ* | Glycerophosphodiester phosphodiesterase OS = *Escherichia coli* OX = 562 GN = glpQ PE = 4 SV = 1 | 1.262656874 | 0.003207089 | Up |
|  | A0A6C8RUQ2 | *fucI* | L-fucose isomerase OS = *Escherichia coli* OX = 562 GN = fucI PE = 3 SV = 1 | 1.262109066 | 0.001359968 | Up |
|  | A0A827ELZ6 | B6R17_001942 | Class II glutamine amidotransferase OS = *Escherichia coli* OX = 562 GN = B6R17_001942 PE = 4 SV = 1 | 1.261980209 | 0.002153187 | Up |
|  | A0A6D0GV09 | *glmS* | Glutamine--fructose-6-phosphate aminotransferase [isomerizing] OS = *Escherichia coli* OX = 562 GN = glmS PE = 3 SV = 1 | 1.259021511 | 0.00190149 | Up |
|  | A0A193LNB2 | GJ11_02835 | Cys-tRNA(Pro)/Cys-tRNA(Cys) deacylase OS = *Escherichia coli* OX = 562 GN = GJ11_02835 PE = 3 SV = 1 | 1.258653919 | 0.018273507 | Up |
|  | A0A2U9KS47 | *narX_2* | Histidine kinase OS = *Escherichia coli* OX = 562 GN = narX_2 PE = 4 SV = 1 | 1.257378253 | 0.00671261 | Up |
|  | A0A6C9QTS1 | *eutM* | Ethanolamine utilization microcompartment protein EutM OS = *Escherichia coli* OX = 562 GN = eutM PE = 4 SV = 1 | 1.256081045 | 0.003682539 | Up |
|  | E1IW65 | *allR* | HTH-type transcriptional repressor AllR OS = *Escherichia coli* MS 145-7 OX = 679204 GN = allR PE = 4 SV = 1 | 1.25581669 | 0.014493071 | Up |
|  | A0A5C9ACD7 | FWK02_31630 | ATP-dependent metalloprotease (Fragment) OS = *Escherichia coli* OX = 562 GN = FWK02_31630 PE = 4 SV = 1 | 1.255641981 | 0.033026918 | Up |
|  | A0A0H2Z3V4 | *gor* | Glutathione reductase Gor OS = *Escherichia coli* O1 : K1 / APEC OX = 405955 GN = gor PE = 3 SV = 1 | 1.253924353 | 0.032016913 | Up |
|  | A0A7U2WD15 | *idnO* | Gluconate 5-dehydrogenase OS = *Escherichia coli* O84 : H7 OX = 2697517 GN = idnO PE = 3 SV = 1 | 1.253828874 | 0.001950847 | Up |
|  | P06996 | *ompC* | Outer membrane porin C OS = *Escherichia coli* (strain K12) OX = 83333 GN = ompC PE = 1 SV = 1 | 1.253717157 | 0.033363885 | Up |
|  | A0A2T1LLF6 | C6985_07380 | Outer membrane protein OmpW OS = *Escherichia coli* OX = 562 GN = C6985_07380 PE = 4 SV = 1 | 1.252411465 | 0.046724034 | Up |
|  | A0A7B5PWP4 | HNC52_18380 | C-type cytochrome OS = *Escherichia coli* OX = 562 GN = HNC52_18380 PE = 4 SV = 1 | 1.25195813 | 0.009945978 | Up |
|  | A0A2H4TXE8 | CV83915_03928 | Glycerophosphodiester phosphodiesterase OS = *Escherichia coli* OX = 562 GN = CV83915_03928 PE = 4 SV = 1 | 1.251821198 | 0.014773654 | Up |
|  | A0A6D0M0X1 | GRW80_28915 | UDP-3-O-(3-hydroxymyristoyl)glucosamine N-acyltransferase (Fragment) OS = *Escherichia coli* OX = 562 GN = GRW80_28915 PE = 4 SV = 1 | 1.250948155 | 0.000588405 | Up |
|  | L7UYD3 | unknow | OmpC porin (Fragment) OS = *Escherichia coli* OX = 562 PE = 3 SV = 1 | 1.250500758 | 0.010343471 | Up |
|  | A0A7T6C0A4 | *fucI* | L-fucose isomerase OS = *Escherichia coli* OX = 562 GN = fucI PE = 3 SV = 1 | 1.249947819 | 0.002365013 | Up |
|  | A0A827QMC8 | *metQ* | D-methionine-binding transport system MetQ OS = *Escherichia coli* OX = 562 GN = metQ PE = 4 SV = 1 | 1.24843325 | 0.002409171 | Up |
|  | M9H7W0 | *dsdA* | D-serine dehydratase OS = *Escherichia coli* MP021561.2 OX = 1116136 GN = dsdA PE = 3 SV = 1 | 1.248263171 | 0.01504265 | Up |
|  | A0A246NVF4 | *flgN* | Flagella biosynthesis chaperone FlgN OS = *Escherichia coli* OX = 562 GN = flgN PE = 3 SV = 1 | 1.248110964 | 0.015196387 | Up |
|  | A0A377KAM5 | *pflB2_2* | Formate acetyltransferase 1 OS = *Escherichia coli* OX = 562 GN = pflB2_2 PE = 4 SV = 1 | 1.247943799 | 0.008260905 | Up |
|  | A0A826X499 | CSE52_001296 | Fumarate hydratase OS = *Escherichia coli* OX = 562 GN = CSE52_001296 PE = 4 SV = 1 | 1.247787763 | 0.002741168 | Up |
|  | A0A1M0PEB6 | BK292_14295 | Maltose O-acetyltransferase OS = *Escherichia coli* OX = 562 GN = BK292_14295 PE = 3 SV = 1 | 1.247238614 | 0.011024347 | Up |
|  | A0A6D0GXH7 | *pepA* | Probable cytosol aminopeptidase OS = *Escherichia coli* OX = 562 GN = pepA PE = 3 SV = 1 | 1.246747472 | 0.023848005 | Up |
|  | P0ACD8 | *hyaB* | Hydrogenase-1 large chain OS = *Escherichia coli* (strain K12) OX = 83333 GN = hyaB PE = 1 SV = 1 | 1.242767182 | 0.036582433 | Up |
|  | A0A6D0ELA1 | GQM09_28295 | Peptidase Do (Fragment) OS = *Escherichia coli* OX = 562 GN = GQM09_28295 PE = 4 SV = 1 | 1.242375674 | 0.001515448 | Up |
|  | A0A4C4J6A1 | *amiC* | N-acetylmuramoyl-L-alanine amidase OS = *Escherichia coli* OX = 562 GN = amiC PE = 4 SV = 1 | 1.241258113 | 0.001352246 | Up |
|  | E8Z5D7 | *pgm* | Pgm (Fragment) OS = *Escherichia coli* OX = 562 GN = pgm PE = 4 SV = 1 | 1.240593312 | 0.019361201 | Up |
|  | A0A2T1LPX8 | *ftsZ* | Cell division protein FtsZ OS = *Escherichia coli* OX = 562 GN = ftsZ PE = 3 SV = 1 | 1.240116726 | 0.039792155 | Up |
|  | E6BR57 | *mtfA* | Protein MtfA OS = *Escherichia coli* MS 85-1 OX = 679202 GN = mtfA PE = 3 SV = 1 | 1.240039038 | 0.038050017 | Up |
|  | A0A377AWG1 | *dacC* | Serine-type D-Ala-D-Ala carboxypeptidase OS = *Escherichia coli* OX = 562 GN = dacC PE = 3 SV = 1 | 1.240024304 | 0.014019725 | Up |
|  | A0A829A9C6 | A1Y7_04499 | Transcriptional activator rfaH OS = *Escherichia coli* KTE119 OX = 1182710 GN = A1Y7_04499 PE = 4 SV = 1 | 1.240005686 | 0.036339317 | Up |
|  | A0A2S5UBH6 | C4M78_14045 | Uncharacterized protein OS = *Escherichia coli* OX = 562 GN = C4M78_14045 PE = 4 SV = 1 | 1.239257197 | 0.003401376 | Up |
|  | A0A829AE96 | A1Y7_02739 | Response regulator in two-component system withYehU OS = *Escherichia coli* KTE119 OX = 1182710 GN = A1Y7_02739 PE = 4 SV = 1 | 1.239246939 | 0.005776282 | Up |
|  | A0A0H2VDQ8 | *uxuB* | D-mannonate oxidoreductase OS = *Escherichia coli* O6 : H1 (strain CFT073 / ATCC 700928 / UPEC) OX = 199310 GN = uxuB PE = 4 SV = 1 | 1.238861769 | 0.013921996 | Up |
|  | A0A376D6E8 | *preT_2* | Putative oxidoreductase OS = *Escherichia coli* OX = 562 GN = preT_2 PE = 4 SV = 1 | 1.238628168 | 0.006706747 | Up |
|  | A0A830VI41 | GF698_09710 | Amino-acid N-acetyltransferase OS = *Escherichia coli* OX = 562 GN = GF698_09710 PE = 4 SV = 1 | 1.238071838 | 0.002599802 | Up |
|  | A0A376LG23 | *groL* | 60 kDa chaperonin OS = *Escherichia coli* OX = 562 GN = groL PE = 3 SV = 1 | 1.237759203 | 0.001067558 | Up |
|  | A0A6N8PRW7 | *cmk* | Cytidylate kinase OS = *Escherichia coli* OX = 562 GN = cmk PE = 3 SV = 1 | 1.237016023 | 0.046579587 | Up |
|  | A0A2X1Q016 | *nagE_1* | N-acetylglucosamine-specific PTS system EIICBA components OS = *Escherichia coli* OX = 562 GN = nagE_1 PE = 4 SV = 1 | 1.236590611 | 0.031461937 | Up |
|  | A0A6D0EJF9 | GQM09_25075 | MetQ/NlpA family lipoprotein (Fragment) OS = *Escherichia coli* OX = 562 GN = GQM09_25075 PE = 3 SV = 1 | 1.23630199 | 0.027725595 | Up |
|  | A0A271QSQ8 | *katG* | Catalase-peroxidase OS = *Escherichia coli* OX = 562 GN = katG PE = 3 SV = 1 | 1.234897461 | 0.018651198 | Up |
|  | A0A376PXV3 | *ybjD* | ATP-dependent endonuclease OS = *Escherichia coli* OX = 562 GN = ybjD PE = 4 SV = 1 | 1.234259647 | 0.006957925 | Up |
|  | A0A827J5L2 | *metK* | Methionine adenosyltransferase OS = *Escherichia coli* OX = 562 GN = metK PE = 4 SV = 1 | 1.232499768 | 0.00793744 | Up |
|  | A0A3L5HCC8 | *ompT* | Omptin family outer membrane protease OmpT OS = *Escherichia coli* OX = 562 GN = ompT PE = 3 SV = 1 | 1.232111004 | 0.038060877 | Up |
|  | A0A4Q0DRR3 | E2123_04090 | Uncharacterized protein OS = *Escherichia coli* OX = 562 GN = E2123_04090 PE = 4 SV = 1 | 1.231287632 | 0.009106025 | Up |
|  | A0A768K1M3 | *yejL* | UPF0352 protein YejL OS = *Escherichia coli* OX = 562 GN = yejL PE = 3 SV = 1 | 1.229728789 | 0.003293101 | Up |
|  | A0A454A4V4 | ECP_1768 | Uncharacterized protein YebO OS = *Escherichia coli* O6 : K15 : H31 (strain 536 / UPEC) OX = 362663 GN = ECP_1768 PE = 4 SV = 1 | 1.229274546 | 0.000534563 | Up |
|  | A0A376MN46 | *katG_3* | Peroxidase/catalase HPI OS = *Escherichia coli* OX = 562 GN = katG_3 PE = 4 SV = 1 | 1.229028865 | 0.00618307 | Up |
|  | A0A7H9QJD6 | *tusA* | Sulfur carrier protein TusA OS = *Escherichia coli* OX = 562 GN = tusA PE = 3 SV = 1 | 1.228380337 | 0.003113612 | Up |
|  | A0A7H9LQR7 | HV146_07990 | DUF535 domain-containing protein OS = *Escherichia coli* OX = 562 GN = HV146_07990 PE = 4 SV = 1 | 1.228342754 | 0.037138602 | Up |
|  | A0A6D0EZ53 | *garR* | 2-hydroxy-3-oxopropionate reductase OS = *Escherichia coli* OX = 562 GN = garR PE = 3 SV = 1 | 1.228293026 | 0.019637108 | Up |
|  | A0A791VCX8 | HJU54_004164 | Fructuronate reductase OS = *Escherichia coli* OX = 562 GN = HJU54_004164 PE = 4 SV = 1 | 1.228258446 | 0.048876263 | Up |
|  | A0A376S727 | dmsA_10 | Putative oxidoreductase major subunit OS = *Escherichia coli* OX = 562 GN = dmsA_10 PE = 4 SV = 1 | 1.227644638 | 0.003590798 | Up |
|  | P11072 | *lit* | Cell death peptidase OS = *Escherichia coli* (strain K12) OX = 83333 GN = lit PE = 3 SV = 2 | 1.226794003 | 0.00215637 | Up |
|  | A0A377KE75 | NCTC9075_06183 | Putative lipoprotein OS = *Escherichia coli* OX = 562 GN = NCTC9075_06183 PE = 4 SV = 1 | 1.225380988 | 0.000504002 | Up |
|  | P14407 | *fumB* | Fumarate hydratase class I, anaerobic OS = *Escherichia coli* (strain K12) OX = 83333 GN = fumB PE = 1 SV = 2 | 1.225057557 | 0.04706666 | Up |
|  | A0A5F1T656 | *grpE* | Protein GrpE OS = *Escherichia coli* OX = 562 GN = grpE PE = 3 SV = 1 | 1.224256467 | 0.000952048 | Up |
|  | A0A4U9U1E4 | *cpdB* | 2',3'-cyclic-nucleotide 2'-phosphodiesterase/3'-nucleotidase OS = *Escherichia coli* OX = 562 GN = cpdB PE = 3 SV = 1 | 1.223402824 | 0.01139111 | Up |
|  | A0A2W6Q4G2 | DNQ45_07465 | Type I site-specific deoxyribonuclease OS = *Escherichia coli* OX = 562 GN = DNQ45_07465 PE = 3 SV = 1 | 1.222930738 | 0.005346039 | Up |
|  | A0A5B9AWW1 | *yajQ* | UPF0234 protein YajQ OS = *Escherichia coli* OX = 562 GN = yajQ PE = 3 SV = 1 | 1.222122859 | 0.020562382 | Up |
|  | A0A2A3WWM5 | BB545_05105 | NADH oxidoreductase OS = *Escherichia coli* OX = 562 GN = BB545_05105 PE = 4 SV = 1 | 1.221855796 | 0.015722136 | Up |
|  | A0A376ZHZ8 | *mdaB* | Modulator of drug activity B OS = *Escherichia coli* OX = 562 GN = mdaB PE = 4 SV = 1 | 1.221619776 | 0.001767347 | Up |
|  | A0A366YRP2 | DS966_11260 | DUF937 domain-containing protein OS = *Escherichia coli* OX = 562 GN = DS966_11260 PE = 4 SV = 1 | 1.221082675 | 0.020194774 | Up |
|  | A0A3W2VQN2 | *ldtE* | L,D-transpeptidase LdtE OS = *Escherichia coli* OX = 562 GN = ldtE PE = 3 SV = 1 | 1.220734435 | 0.012993975 | Up |
|  | A0A2T1LLD6 | *purU* | Formyltetrahydrofolate deformylase OS = *Escherichia coli* OX = 562 GN = purU PE = 3 SV = 1 | 1.220492092 | 0.019440542 | Up |
|  | A0A418GJM4 | D3C88_15285 | Anaerobic ribonucleoside-triphosphate reductase (Fragment) OS = *Escherichia coli* OX = 562 GN = D3C88_15285 PE = 4 SV = 1 | 1.219921712 | 0.001355365 | Up |
|  | A0A6D0DG16 | *hsdR* | Type I site-specific deoxyribonuclease OS = *Escherichia coli* OX = 562 GN = hsdR PE = 3 SV = 1 | 1.218584168 | 0.023862139 | Up |
|  | A0A376ME67 | *melA* | Alpha-galactosidase OS = *Escherichia coli* OX = 562 GN = melA PE = 3 SV = 1 | 1.218579573 | 0.017689455 | Up |
|  | A0A6L7A3K9 | *nanA* | N-acetylneuraminate lyase (Fragment) OS = *Escherichia coli* OX = 562 GN = nanA PE = 3 SV = 1 | 1.218012937 | 0.020942735 | Up |
|  | A0A831F693 | *allB* | Allantoinase AllB OS = *Escherichia coli* OX = 562 GN = allB PE = 4 SV = 1 | 1.21796455 | 0.005805418 | Up |
|  | A0A1X3KRU5 | *nudJ* | Phosphatase NudJ OS = *Escherichia coli* H605 OX = 656410 GN = nudJ PE = 3 SV = 1 | 1.216748303 | 0.000614544 | Up |
|  | A0A4T4XPV4 | DXT71_16195 | L-arabinose-binding periplasmic protein OS = *Escherichia coli* OX = 562 GN = DXT71_16195 PE = 3 SV = 1 | 1.216226032 | 0.014808886 | Up |
|  | A0A827RU54 | *bioD* | ATP-dependent dethiobiotin synthetase BioD OS = *Escherichia coli* OX = 562 GN = bioD PE = 4 SV = 1 | 1.216182904 | 0.007373587 | Up |
|  | A0A7I8ZNN1 | *ydcH* | YdcH protein OS = *Escherichia coli* OX = 562 GN = ydcH PE = 4 SV = 1 | 1.215770155 | 0.048310445 | Up |
|  | A0A3W4K7I4 | EAX79_29040 | GTP-binding protein (Fragment) OS = *Escherichia coli* OX = 562 GN = EAX79_29040 PE = 4 SV = 1 | 1.215269084 | 0.029677278 | Up |
|  | A0A6N8Q332 | GRW77_20505 | 2',3'-cyclic-nucleotide 2'-phosphodiesterase/3'-nucleotidase OS = *Escherichia coli* OX = 562 GN = GRW77_20505 PE = 3 SV = 1 | 1.215174408 | 0.004231872 | Up |
|  | A0A2X7I0D2 | *pabB* | Aminodeoxychorismate synthase OS = *Escherichia coli* OX = 562 GN = pabB PE = 3 SV = 1 | 1.214694526 | 0.006639291 | Up |
|  | A0A377B7Z2 | *fruA_2* | PTS system fructose-specific transporter subunit IIBC OS = *Escherichia coli* OX = 562 GN = fruA_2 PE = 4 SV = 1 | 1.214621939 | 0.009644385 | Up |
|  | A0A826LFD7 | C5542_001192 | DeoR/GlpR transcriptional regulator OS = *Escherichia coli* OX = 562 GN = C5542_001192 PE = 4 SV = 1 | 1.213973672 | 9.8714E-05 | Up |
|  | D8A5G6 | *atoS* | Histidine kinase OS = *Escherichia coli* (strain MS 21-1) OX = 749527 GN = atoS PE = 4 SV = 1 | 1.213957901 | 0.036407955 | Up |
|  | A0A5D8SLG5 | E0I42_23495 | Acetyl-CoA C-acetyltransferase OS = *Escherichia coli* OX = 562 GN = E0I42_23495 PE = 3 SV = 1 | 1.213867551 | 0.036595452 | Up |
|  | A0A792TAR7 | *rsgA* | Small ribosomal subunit biogenesis GTPase RsgA OS = *Escherichia coli* OX = 562 GN = rsgA PE = 3 SV = 1 | 1.2138081 | 0.022840229 | Up |
|  | A0A0K4G9W2 | *greB* | Transcription elongation factor GreB OS = *Escherichia coli* OX = 562 GN = greB PE = 3 SV = 1 | 1.212905627 | 0.004960106 | Up |
|  | A0A376ZTY7 | *ispE* | 4-diphosphocytidyl-2-C-methyl-D-erythritol kinase OS = *Escherichia coli* OX = 562 GN = ispE PE = 3 SV = 1 | 1.21280229 | 0.008488412 | Up |
|  | Q8XAA8 | Z3776 | Ancillary SecYEG translocon subunit OS = *Escherichia coli* O157 : H7 OX = 83334 GN = Z3776 PE = 3 SV = 2 | 1.212511991 | 0.019569627 | Up |
|  | A0A826L3Z6 | *cysZ* | Sulfate transporter CysZ OS = *Escherichia coli* O6 OX = 217992 GN = cysZ PE = 4 SV = 1 | 1.21117506 | 0.047851486 | Up |
|  | C4ZW75 | *dctA* | C4-dicarboxylate transport protein OS = *Escherichia coli* (strain K12 / MC4100 / BW2952) OX = 595496 GN = dctA PE = 3 SV = 1 | 1.210799181 | 0.01926694 | Up |
|  | A0A5E8FDU5 | *parE* | DNA topoisomerase 4 subunit B OS = *Escherichia coli* D6-113.11 OX = 1400022 GN = parE PE = 3 SV = 1 | 1.210499587 | 0.000665873 | Up |
|  | A0A6D0EJA7 | GQM09_23615 | Glutamine--fructose-6-phosphate aminotransferase [isomerizing] (Fragment) OS = *Escherichia coli* OX = 562 GN = GQM09_23615 PE = 4 SV = 1 | 1.210317903 | 0.040650521 | Up |
|  | A0A6D0DAQ1 | FQ021_12490 | DUF1877 family protein OS = *Escherichia coli* OX = 562 GN = FQ021_12490 PE = 4 SV = 1 | 1.209105835 | 0.001342456 | Up |
|  | A0A376LED5 | *trxA_2* | Thioredoxin OS = *Escherichia coli* OX = 562 GN = trxA_2 PE = 3 SV = 1 | 1.2089899 | 0.010767977 | Up |
|  | A0A827HLW1 | *surE* | 5'/3'-nucleotidase SurE OS = *Escherichia coli* OX = 562 GN = surE PE = 4 SV = 1 | 1.208287481 | 0.001676709 | Up |
|  | A0A241QPT0 | *kdbD* | Histidine kinase OS = *Escherichia coli* OX = 562 GN = kdbD PE = 4 SV = 1 | 1.207730632 | 0.021364017 | Up |
|  | A0A1V2GDA3 | BXT93_15460 | Uncharacterized protein OS = *Escherichia coli* OX = 562 GN = BXT93_15460 PE = 3 SV = 1 | 1.206874095 | 0.013747817 | Up |
|  | A0A0K4PX90 | *glpK* | Glycerol kinase OS = *Escherichia coli* OX = 562 GN = glpK PE = 3 SV = 1 | 1.206504066 | 9.08424E-05 | Up |
|  | A0A3L0W137 | *groL* | 60 kDa chaperonin OS = *Escherichia coli* OX = 562 GN = groL PE = 3 SV = 1 | 1.20646098 | 0.027760015 | Up |
|  | A0A5F1DTT1 | *upp* | Uracil phosphoribosyltransferase OS = *Escherichia coli* OX = 562 GN = upp PE = 3 SV = 1 | 1.206443099 | 0.018486454 | Up |
|  | A0A6D0IPV2 | *ribE* | Riboflavin synthase OS = *Escherichia coli* OX = 562 GN = ribE PE = 4 SV = 1 | 1.205684762 | 0.022717455 | Up |
|  | B7N7L8 | *yggX* | Probable Fe(2+)-trafficking protein OS = *Escherichia coli* O17 : K52 : H18 (strain UMN026 / ExPEC) OX = 585056 GN = yggX PE = 3 SV = 1 | 1.205264551 | 0.000205896 | Up |
|  | A0A2T1LJ96 | C6985_11415 | Flagellar M-ring protein OS = *Escherichia coli* OX = 562 GN = C6985_11415 PE = 3 SV = 1 | 1.205230632 | 0.007651608 | Up |
|  | A0A5P0J6D0 | EIZ93_05025 | EAL domain-containing protein OS = *Escherichia coli* OX = 562 GN = EIZ93_05025 PE = 4 SV = 1 | 1.205205844 | 0.009967346 | Up |
|  | A0A827KNR7 | *eutL* | Ethanolamine utilization microcompartment protein EutL OS = *Escherichia coli* OX = 562 GN = eutL PE = 4 SV = 1 | 1.204832909 | 0.005257325 | Up |
|  | A0A7U2IP63 | *fucU* | L-fucose mutarotase OS = *Escherichia coli* OX = 562 GN = fucU PE = 3 SV = 1 | 1.204144263 | 0.006663365 | Up |
|  | A0A826LKU7 | C5542_001496 | HlyD family secretion protein OS = *Escherichia coli* OX = 562 GN = C5542_001496 PE = 4 SV = 1 | 1.203177045 | 0.014273639 | Up |
|  | A0A7I9AJI9 | *appY_1* | AppY_1 protein OS = *Escherichia coli* OX = 562 GN = appY_1 PE = 4 SV = 1 | 1.20316094 | 0.013342008 | Up |
|  | A0A826V574 | *wzzB* | LPS O-antigen chain length determinant protein WzzB OS = *Escherichia coli* OX = 562 GN = wzzB PE = 4 SV = 1 | 1.202971061 | 0.043663233 | Up |
|  | A0A6L4XF87 | *ompC* | Porin OmpC OS = *Escherichia coli* OX = 562 GN = ompC PE = 3 SV = 1 | 1.202584417 | 0.022378165 | Up |
|  | A0A484Y9W9 | *pgi_2* | Glucose-6-phosphate isomerase OS = *Escherichia coli* OX = 562 GN = pgi_2 PE = 4 SV = 1 | 1.20219485 | 0.04772389 | Up |
|  | A0A853WGK5 | *copA* | Cu+ exporting ATPase OS = *Escherichia coli* OX = 562 GN = copA PE = 4 SV = 1 | 1.201902976 | 0.0178182 | Up |
|  | C3SII8 | *sthA* | Soluble pyridine nucleotide transhydrogenase OS = *Escherichia coli* OX = 562 GN = sthA PE = 3 SV = 1 | 1.200443066 | 0.007609822 | Up |
|  | A0A6N8QIT2 | *ileS* | Isoleucine--tRNA ligase (Fragment) OS = *Escherichia coli* OX = 562 GN = ileS PE = 3 SV = 1 | 0.833072243 | 0.012679312 | Down |
|  | A0A6L4XIH0 | GP710_11515 | EAL domain-containing protein OS = *Escherichia coli* OX = 562 GN = GP710_11515 PE = 4 SV = 1 | 0.832948325 | 0.013579962 | Down |
|  | A0A827MI46 | BOY03_000822 | Holo-ACP synthase OS = *Escherichia coli* OX = 562 GN = BOY03_000822 PE = 4 SV = 1 | 0.83288902 | 0.000788933 | Down |
|  | F4SUP9 | ECIG_01167 | Protein II protein (Protein X protein) OS = *Escherichia coli* M605 OX = 656417 GN = ECIG_01167 PE = 4 SV = 1 | 0.832334888 | 0.007073705 | Down |
|  | A0A0B0Z3I7 | *rmlA* | Glucose-1-phosphate thymidylyltransferase OS = *Escherichia coli* OX = 562 GN = rmlA PE = 3 SV = 1 | 0.832015008 | 0.024321853 | Down |
|  | A0A222QNW9 | A610_2752 | Cytochrome c-type protein OS = *Escherichia coli* NCCP15648 OX = 1200752 GN = A610_2752 PE = 3 SV = 1 | 0.831957919 | 0.040194634 | Down |
|  | A0A376I262 | *pgm* | Phosphoglucomutase OS = *Escherichia coli* OX = 562 GN = pgm PE = 3 SV = 1 | 0.831735355 | 0.046561676 | Down |
|  | A0A0L1C331 | *aphA* | Class B acid phosphatase OS = *Escherichia coli* OX = 562 GN = aphA PE = 3 SV = 1 | 0.83157961 | 0.016811636 | Down |
|  | B1XGK1 | *nadE* | NH(3)-dependent NAD(+) synthetase OS = *Escherichia coli* (strain K12 / DH10B) OX = 316385 GN = nadE PE = 3 SV = 1 | 0.831290612 | 0.012289941 | Down |
|  | P0ADU3 | *ygiN* | Probable quinol monooxygenase YgiN OS = *Escherichia coli* O157 : H7 OX = 83334 GN = ygiN PE = 3 SV = 1 | 0.830894483 | 0.001028699 | Down |
|  | A0A7U9AWI8 | *trpA* | Tryptophan synthase alpha chain OS = *Escherichia coli* TA206 OX = 656440 GN = trpA PE = 3 SV = 1 | 0.830560037 | 0.006085218 | Down |
|  | A0A6L9C2L7 | *lpxC* | UDP-3-O-acyl-N-acetylglucosamine deacetylase OS = *Escherichia coli* OX = 562 GN = lpxC PE = 3 SV = 1 | 0.83044885 | 0.002773231 | Down |
|  | A0A376I4P1 | *gltP_2* | Proton/glutamate-aspartate symporter OS = *Escherichia coli* OX = 562 GN = gltP_2 PE = 3 SV = 1 | 0.830202893 | 0.005873072 | Down |
|  | A0A376HXM1 | *speC* | Ornithine decarboxylase OS = *Escherichia coli* OX = 562 GN = speC PE = 3 SV = 1 | 0.829731258 | 0.031827597 | Down |
|  | A0A5N3D643 | *dld* | Quinone-dependent D-lactate dehydrogenase OS = *Escherichia coli* OX = 562 GN = dld PE = 3 SV = 1 | 0.829408117 | 0.026254926 | Down |
|  | D6IFZ9 | ECDG_04381 | HTH-type transcriptional regulator yieP OS = *Escherichia coli* B185 OX = 550676 GN = ECDG_04381 PE = 4 SV = 1 | 0.829076204 | 0.005770803 | Down |
|  | A0A2T1LLY9 | C6985_05005 | Phosphoporin PhoE OS = *Escherichia coli* OX = 562 GN = C6985_05005 PE = 3 SV = 1 | 0.829000029 | 0.018970894 | Down |
|  | A0A377K5F9 | *flhE* | Flagellar protein FlhE OS = *Escherichia coli* OX = 562 GN = flhE PE = 4 SV = 1 | 0.82874775 | 0.006664763 | Down |
|  | A0A7U9A075 | ECFG_04560 | YajO OS = *Escherichia coli* FVEC1302 OX = 656379 GN = ECFG_04560 PE = 4 SV = 1 | 0.828662209 | 0.001265768 | Down |
|  | A0A6N4KHV8 | *sbmC* | DNA gyrase inhibitor OS = *Escherichia coli* OX = 562 GN = sbmC PE = 3 SV = 1 | 0.82812687 | 0.020353871 | Down |
|  | A0A774NC69 | GFY48_24340 | S-formylglutathione hydrolase OS = *Escherichia coli* OX = 562 GN = GFY48_24340 PE = 3 SV = 1 | 0.828061239 | 0.035835639 | Down |
|  | Q47308 | *aph* | Aminoglycoside 3'-phosphotransferase OS = *Escherichia coli* OX = 562 GN = aph PE = 3 SV = 1 | 0.827065043 | 0.014093621 | Down |
|  | A0A6D0ELG5 | GQM09_27360 | Branched chain amino acid aminotransferase (Fragment) OS = *Escherichia coli* OX = 562 GN = GQM09_27360 PE = 3 SV = 1 | 0.826515056 | 0.02143724 | Down |
|  | Q6JZJ4 | *icd* | Isocitrate dehydrogenase [NADP] (Fragment) OS = *Escherichia coli* OX = 562 GN = icd PE = 4 SV = 1 | 0.826373785 | 0.0002165 | Down |
|  | A0A854RJR3 | *clpP* | ATP-dependent Clp endopeptidase, proteolytic subunit ClpP OS = *Escherichia coli* OX = 562 GN = clpP PE = 4 SV = 1 | 0.826346467 | 0.014978089 | Down |
|  | B7UHJ6 | *pyrG* | CTP synthase OS = *Escherichia coli* O127 : H6 (strain E2348/69 / EPEC) OX = 574521 GN = pyrG PE = 3 SV = 1 | 0.826342394 | 0.001230265 | Down |
|  | A0A368IPW2 | *yidA* | Sugar phosphatase YidA OS = *Escherichia coli* OX = 562 GN = yidA PE = 4 SV = 1 | 0.82629239 | 0.006603239 | Down |
|  | A0A3Q0MWV8 | *flgE* | Flagellar hook protein FlgE OS = *Escherichia coli* OX = 562 GN = flgE PE = 3 SV = 1 | 0.826111407 | 0.007247281 | Down |
|  | A0A7A2WX01 | *yebG* | DNA damage-inducible protein YebG OS = *Escherichia coli* OX = 562 GN = yebG PE = 4 SV = 1 | 0.825378982 | 0.040086759 | Down |
|  | A0A3L0VYM4 | D9F05_06435 | AMP nucleosidase OS = *Escherichia coli* OX = 562 GN = D9F05_06435 PE = 4 SV = 1 | 0.824552773 | 0.010613571 | Down |
|  | A0A3P4Z1A7 | *dhaL* | PEP-dependent dihydroxyacetone kinase, ADP-binding subunit DhaL OS = *Escherichia coli* OX = 562 GN = dhaL PE = 4 SV = 1 | 0.824517971 | 0.000830606 | Down |
|  | P0AEH3 | *elaA* | Protein ElaA OS = *Escherichia coli* (strain K12) OX = 83333 GN = elaA PE = 1 SV = 1 | 0.824156057 | 0.008965943 | Down |
|  | A0A829DLS5 | EC2875150_4547 | Binding--dependent transport system inner membrane component family protein OS = *Escherichia coli* 2875150 OX = 1116036 GN = EC2875150_4547 PE = 4 SV = 1 | 0.823628449 | 0.04487711 | Down |
|  | A0A6M0PRB2 | *rssB* | Regulator of RpoS OS = *Escherichia coli* OX = 562 GN = rssB PE = 3 SV = 1 | 0.823552505 | 0.009249623 | Down |
|  | A0A4D9Z8J3 | ELX70_02700 | LysR family transcriptional regulator OS = *Escherichia coli* OX = 562 GN = ELX70_02700 PE = 3 SV = 1 | 0.823444934 | 0.007402459 | Down |
|  | A0A854WA48 | *glgC* | Glucose-1-phosphate adenylyltransferase OS = *Escherichia coli* OX = 562 GN = glgC PE = 4 SV = 1 | 0.823422133 | 0.005693405 | Down |
|  | A0A2K3TRG7 | *fdxH* | Formate dehydrogenase iron-sulfur subunit OS = *Escherichia coli* OX = 562 GN = fdxH PE = 4 SV = 1 | 0.823378977 | 0.002607935 | Down |
|  | A0A246NR53 | *cysJ* | Sulfite reductase [NADPH] flavoprotein alpha-component OS = *Escherichia coli* OX = 562 GN = cysJ PE = 3 SV = 1 | 0.823286374 | 0.00982949 | Down |
|  | A0A826X5I1 | *uvrA* | Excinuclease ABC subunit UvrA OS = *Escherichia coli* OX = 562 GN = uvrA PE = 4 SV = 1 | 0.822211573 | 0.001126731 | Down |
|  | A0A1X3J422 | ECXG_01895 | Putative MOSC domain protein OS = *Escherichia coli* TA447 OX = 656447 GN = ECXG_01895 PE = 4 SV = 1 | 0.821632947 | 0.039207338 | Down |
|  | A0A838AWA8 | *lpxB* | Lipid-A-disaccharide synthase OS = *Escherichia coli* OX = 562 GN = lpxB PE = 4 SV = 1 | 0.82146976 | 0.008910748 | Down |
|  | P0AFW6 | *rnk* | Regulator of nucleoside diphosphate kinase OS = *Escherichia coli* O157 : H7 OX = 83334 GN = rnk PE = 3 SV = 1 | 0.820396172 | 0.02131678 | Down |
|  | B7MGL2 | *gpmA* | 2,3-bisphosphoglycerate-dependent phosphoglycerate mutase OS = *Escherichia coli* O45 : K1 (strain S88 / ExPEC) OX = 585035 GN = gpmA PE = 3 SV = 1 | 0.820130312 | 0.010737051 | Down |
|  | A0A6L4XPN6 | *galK* | Galactokinase OS = *Escherichia coli* OX = 562 GN = galK PE = 3 SV = 1 | 0.819582482 | 0.033920003 | Down |
|  | A0A7D7DVA6 | *icd* | Isocitrate dehydrogenase [NADP] OS = *Escherichia coli* OX = 562 GN = icd PE = 4 SV = 1 | 0.819463902 | 0.000279427 | Down |
|  | D6JF44 | *rbfA* | Ribosome-binding factor A OS = *Escherichia coli* B354 OX = 550677 GN = rbfA PE = 3 SV = 1 | 0.818925659 | 0.005627983 | Down |
|  | E6BD92 | HMPREF9350_00052 | Phospho-2-dehydro-3-deoxyheptonate aldolase OS = *Escherichia coli* MS 85-1 OX = 679202 GN = HMPREF9350_00052 PE = 3 SV = 1 | 0.818643015 | 0.013039822 | Down |
|  | A0A790HG40 | BO068_003177 | YncE family protein OS = *Escherichia coli* OX = 562 GN = BO068_003177 PE = 4 SV = 1 | 0.818618899 | 0.038430294 | Down |
|  | P46887 | *yecH* | Uncharacterized protein YecH OS = *Escherichia coli* (strain K12) OX = 83333 GN = yecH PE = 4 SV = 2 | 0.818600642 | 0.047296868 | Down |
|  | A0A7A9K434 | HMS79_22590 | ProQ domain-containing protein OS = *Escherichia coli* OX = 562 GN = HMS79_22590 PE = 4 SV = 1 | 0.817793125 | 0.0123811 | Down |
|  | A0A376Y3C3 | *ygfB* | UPF0149 protein YgfB OS = *Escherichia coli* OX = 562 GN = ygfB PE = 3 SV = 1 | 0.816449769 | 0.001041213 | Down |
|  | A0A0L1C6L2 | *dppD* | Peptide ABC transporter ATP-binding protein OS = *Escherichia coli* OX = 562 GN = dppD PE = 4 SV = 1 | 0.816236299 | 0.008132883 | Down |
|  | A0A6N8Q4X1 | GRW77_29845 | Tagatose-bisphosphate aldolase (Fragment) OS = *Escherichia coli* OX = 562 GN = GRW77_29845 PE = 4 SV = 1 | 0.815904405 | 0.00400837 | Down |
|  | A0A826KAA6 | *lolA* | Outer membrane lipoprotein chaperone LolA OS = *Escherichia coli* OX = 562 GN = lolA PE = 4 SV = 1 | 0.815320041 | 0.002510505 | Down |
|  | A0A6N8PSS5 | *nadC* | Quinolinate phosphoribosyltransferase [decarboxylating] OS = *Escherichia coli* OX = 562 GN = nadC PE = 3 SV = 1 | 0.815001455 | 0.032804895 | Down |
|  | A0A844EXI2 | GKE84_15090 | AAA family ATPase (Fragment) OS = *Escherichia coli* OX = 562 GN = GKE84_15090 PE = 4 SV = 1 | 0.814252698 | 0.006826172 | Down |
|  | A0A827DZ73 | A2F99_001257 | LysR family transcriptional regulator OS = *Escherichia coli* OX = 562 GN = A2F99_001257 PE = 4 SV = 1 | 0.814163262 | 0.000374803 | Down |
|  | A0A1X9THD8 | CA593_09065 | Alpha-1,4 glucan phosphorylase OS = *Escherichia coli* OX = 562 GN = CA593_09065 PE = 3 SV = 1 | 0.813965317 | 0.003916769 | Down |
|  | A0A826NJ86 | *infB* | Translation initiation factor IF-2 OS = *Escherichia coli* O157 OX = 1045010 GN = infB PE = 4 SV = 1 | 0.81388137 | 0.000508767 | Down |
|  | A0A376MTF2 | *nagB_1* | Glucosamine-6-phosphate isomerase OS = *Escherichia coli* OX = 562 GN = nagB_1 PE = 4 SV = 1 | 0.8134024 | 0.0163636 | Down |
|  | A0A1X3LVF6 | EAZG_00618 | Galactose-1-phosphate uridylyltransferase OS = *Escherichia coli* TA249 OX = 656441 GN = EAZG_00618 PE = 3 SV = 1 | 0.812872738 | 0.002446779 | Down |
|  | C3SRM0 | ECs4127 | Biotin carboxyl carrier protein of acetyl-CoA carboxylase OS = *Escherichia coli* OX = 562 GN = ECs4127 PE = 4 SV = 1 | 0.81199084 | 0.012385506 | Down |
|  | P24203 | *yjiA* | P-loop guanosine triphosphatase YjiA OS = *Escherichia coli* (strain K12) OX = 83333 GN = yjiA PE = 1 SV = 3 | 0.811976307 | 0.031892013 | Down |
|  | A0A376U3J7 | *yjiY* | Putative carbon starvation protein OS = *Escherichia coli* OX = 562 GN = yjiY PE = 3 SV = 1 | 0.811890775 | 0.030055172 | Down |
|  | A0A6L6I9I8 | F9B07_26140 | Cupin domain-containing protein OS = *Escherichia coli* OX = 562 GN = F9B07_26140 PE = 4 SV = 1 | 0.811741657 | 0.048601503 | Down |
|  | A0A2X1N9Q1 | *yhbN* | Lipopolysaccharide transport periplasmic protein LptA OS = *Escherichia coli* OX = 562 GN = yhbN PE = 4 SV = 1 | 0.810739218 | 0.014766239 | Down |
|  | F4SMG2 | ECHG_00753 | Protein YccU OS = *Escherichia coli* H736 OX = 656414 GN = ECHG_00753 PE = 4 SV = 1 | 0.809982618 | 0.005113181 | Down |
|  | P76015 | *dhaK* | PEP-dependent dihydroxyacetone kinase, dihydroxyacetone-binding subunit DhaK OS = *Escherichia coli* (strain K12) OX = 83333 GN = dhaK PE = 1 SV = 2 | 0.80995605 | 0.007603449 | Down |
|  | A0A2T1LHD8 | *bepA* | Beta-barrel assembly-enhancing protease OS = *Escherichia coli* OX = 562 GN = bepA PE = 3 SV = 1 | 0.808895695 | 0.02834506 | Down |
|  | V0SPM8 | HMPREF1595_02050 | Uncharacterized protein OS = *Escherichia coli* 907672 OX = 1268982 GN = HMPREF1595_02050 PE = 4 SV = 1 | 0.80786268 | 0.008461476 | Down |
|  | A0A2I6IHS3 | BVL39_24760 | Phospho-2-dehydro-3-deoxyheptonate aldolase OS = *Escherichia coli* OX = 562 GN = BVL39_24760 PE = 3 SV = 1 | 0.807288029 | 0.00123434 | Down |
|  | A0A417ZXX0 | D3O91_08965 | PTS glucitol/sorbitol transporter subunit IIA OS = *Escherichia coli* OX = 562 GN = D3O91_08965 PE = 4 SV = 1 | 0.805585744 | 0.041601685 | Down |
|  | A0A0A0FCP4 | EL76_2820 | Dihydroxyacetone kinase subunit K OS = *Escherichia coli* G3/10 OX = 1455601 GN = EL76_2820 PE = 4 SV = 1 | 0.805487592 | 0.003366127 | Down |
|  | A0A6D0LUI0 | GRW80_21050 | Tyrosine-type recombinase/integrase OS = *Escherichia coli* OX = 562 GN = GRW80_21050 PE = 4 SV = 1 | 0.805439131 | 0.035261115 | Down |
|  | A0A5F1DXH6 | CQB02_08155 | Alkene reductase OS = *Escherichia coli* OX = 562 GN = CQB02_08155 PE = 4 SV = 1 | 0.803554733 | 0.017755619 | Down |
|  | P0AD50 | *yfiA* | Ribosome-associated factor Y OS = *Escherichia coli* O6 : H1 (strain CFT073 / ATCC 700928 / UPEC) OX = 199310 GN = yfiA PE = 3 SV = 2 | 0.803431395 | 0.040967032 | Down |
|  | A0A7D5H5I7 | *napA* | Periplasmic nitrate reductase OS = *Escherichia coli* OX = 562 GN = napA PE = 3 SV = 1 | 0.803306415 | 0.014427192 | Down |
|  | A0A0K4MBS7 | *trmJ* | tRNA (cytidine/uridine-2'-O-)-methyltransferase TrmJ OS = *Escherichia coli* OX = 562 GN = trmJ PE = 3 SV = 1 | 0.803110907 | 0.010002019 | Down |
|  | A0A377D334 | *trpC* | Multifunctional fusion protein OS = *Escherichia coli* OX = 562 GN = trpC PE = 3 SV = 1 | 0.802686661 | 0.002459768 | Down |
|  | A0A5E8NUZ0 | ACN68_19100 | Peptidyl-prolyl cis-trans isomerase OS = *Escherichia coli* OX = 562 GN = ACN68_19100 PE = 3 SV = 1 | 0.802204682 | 0.010319483 | Down |
|  | A0A4C4JJD6 | *qorA* | Quinone oxidoreductase OS = *Escherichia coli* OX = 562 GN = qorA PE = 3 SV = 1 | 0.802129032 | 0.032753318 | Down |
|  | A0A770C125 | *malK* | Maltose/maltodextrin import ATP-binding protein MalK OS = *Escherichia coli* OX = 562 GN = malK PE = 4 SV = 1 | 0.801997734 | 0.007552954 | Down |
|  | A0A6L4XR91 | *aroG* | Phospho-2-dehydro-3-deoxyheptonate aldolase OS = *Escherichia coli* OX = 562 GN = aroG PE = 3 SV = 1 | 0.801715754 | 0.000215688 | Down |
|  | D3GY01 | EC042_4857 | Putative glycoprotein/receptor OS = *Escherichia coli* O44 : H18 (strain 042 / EAEC) OX = 216592 GN = EC042_4857 PE = 4 SV = 1 | 0.801601351 | 7.82972E-05 | Down |
|  | I2STI4 | *phsB* | Thiosulfate reductase electron transport protein phsb OS = *Escherichia coli* 1.2264 OX = 869675 GN = phsB PE = 4 SV = 1 | 0.801170843 | 0.004614375 | Down |
|  | A0A024L1I7 | *cheB* | Protein-glutamate methylesterase/protein-glutamine glutaminase OS = *Escherichia coli* OX = 562 GN = cheB PE = 3 SV = 1 | 0.800933696 | 0.007002249 | Down |
|  | A0A3T9CJN2 | *nusA* | Transcription termination/antitermination protein NusA OS = *Escherichia coli* OX = 562 GN = nusA PE = 3 SV = 1 | 0.800563112 | 0.000672662 | Down |
|  | A0A7U0H0D9 | *topA* | DNA topoisomerase 1 OS = *Escherichia coli* OX = 562 GN = topA PE = 3 SV = 1 | 0.799913316 | 0.003812575 | Down |
|  | V0YFL6 | *rpmC* | 50S ribosomal protein L29 OS = *Escherichia coli* 908525 OX = 1268995 GN = rpmC PE = 3 SV = 1 | 0.799795898 | 0.013608716 | Down |
|  | A0A4Y8FUT9 | *cheY* | Chemotaxis protein CheY OS = *Escherichia coli* OX = 562 GN = cheY PE = 4 SV = 1 | 0.799659073 | 0.028155036 | Down |
|  | A0A7B9FK36 | *ebgR* | Transcriptional regulator EbgR OS = *Escherichia coli* OX = 562 GN = ebgR PE = 4 SV = 1 | 0.799218598 | 0.003486453 | Down |
|  | A0A5D1FPW1 | *motA* | Flagellar motor stator protein MotA OS = *Escherichia coli* OX = 562 GN = motA PE = 3 SV = 1 | 0.798971765 | 0.025181116 | Down |
|  | P0ACM9 | *yihL* | Uncharacterized HTH-type transcriptional regulator YihL OS = *Escherichia coli* (strain K12) OX = 83333 GN = yihL PE = 4 SV = 1 | 0.79897062 | 0.024356884 | Down |
|  | A0A1X9TR86 | *rihA* | Pyrimidine-specific ribonucleoside hydrolase RihA OS = *Escherichia coli* OX = 562 GN = rihA PE = 3 SV = 1 | 0.798693752 | 0.007635579 | Down |
|  | A0A5D8S3Y2 | *fliD* | Filament cap protein (Fragment) OS = *Escherichia coli* OX = 562 GN = fliD PE = 3 SV = 1 | 0.797645747 | 0.035603648 | Down |
|  | P32053 | *intA* | Prophage integrase IntA OS = *Escherichia coli* (strain K12) OX = 83333 GN = intA PE = 1 SV = 2 | 0.797240109 | 0.001390378 | Down |
|  | A0A0H2V995 | *cysK* | Cysteine synthase OS = *Escherichia coli* O6 : H1 (strain CFT073 / ATCC 700928 / UPEC) OX = 199310 GN = cysK PE = 3 SV = 1 | 0.797147546 | 0.000928305 | Down |
|  | A0A271QU37 | *yncE* | Putative receptor OS = *Escherichia coli* OX = 562 GN = yncE PE = 4 SV = 1 | 0.794397549 | 0.005255402 | Down |
|  | P25516 | *acnA* | Aconitate hydratase A OS = *Escherichia coli* (strain K12) OX = 83333 GN = acnA PE = 1 SV = 3 | 0.793744011 | 0.003164396 | Down |
|  | A0A2T1LFH3 | *parC* | DNA topoisomerase 4 subunit A OS = *Escherichia coli* OX = 562 GN = parC PE = 3 SV = 1 | 0.792886463 | 0.043940712 | Down |
|  | A0A6D0JE36 | GP946_28495 | ATP-binding cassette domain-containing protein (Fragment) OS = *Escherichia coli* OX = 562 GN = GP946_28495 PE = 4 SV = 1 | 0.7924127 | 0.021700161 | Down |
|  | I2X034 | EC40967_1679 | Oxidoreductase, NAD-binding domain protein OS = *Escherichia coli* 4.0967 OX = 869687 GN = EC40967_1679 PE = 4 SV = 1 | 0.792339361 | 0.001012206 | Down |
|  | A0A830VIZ5 | GF698_14020 | Maltodextrin phosphorylase OS = *Escherichia coli* OX = 562 GN = GF698_14020 PE = 4 SV = 1 | 0.792245617 | 0.023669494 | Down |
|  | F4TAV4 | *lon* | Lon protease OS = *Escherichia coli* M718 OX = 656419 GN = lon PE = 2 SV = 1 | 0.792021648 | 0.00414107 | Down |
|  | A0A0J8XKA8 | *metC* | Cystathionine beta-lyase OS = *Escherichia coli* OX = 562 GN = metC PE = 3 SV = 1 | 0.790521215 | 0.00057535 | Down |
|  | A0A831EYQ8 | HIR12_000351 | SDR family oxidoreductase OS = *Escherichia coli* OX = 562 GN = HIR12_000351 PE = 4 SV = 1 | 0.790177018 | 0.025317574 | Down |
|  | A0A376Q109 | *aceF* | Acetyltransferase component of pyruvate dehydrogenase complex OS = *Escherichia coli* OX = 562 GN = aceF PE = 3 SV = 1 | 0.789763502 | 0.000155924 | Down |
|  | A0A2Y0KUW1 | *mgtA* | Magnesium-transporting ATPase, P-type 1 OS = *Escherichia coli* OX = 562 GN = mgtA PE = 3 SV = 1 | 0.789371732 | 0.021870508 | Down |
|  | A0A377CPF6 | *rhlB* | ATP-dependent RNA helicase RhlB OS = *Escherichia coli* OX = 562 GN = rhlB PE = 3 SV = 1 | 0.789162611 | 0.000494169 | Down |
|  | A0A5D8SP90 | E0I42_21390 | Peptidyl-prolyl cis-trans isomerase OS = *Escherichia coli* OX = 562 GN = E0I42_21390 PE = 3 SV = 1 | 0.788911348 | 7.49852E-05 | Down |
|  | A0A6N6XC98 | *serS* | Seryl-tRNA synthetase (Fragment) OS = *Escherichia coli* OX = 562 GN = serS PE = 4 SV = 1 | 0.788839735 | 0.018301976 | Down |
|  | A0A377A2N6 | *prc_5* | Tail-specific protease OS = *Escherichia coli* OX = 562 GN = prc_5 PE = 4 SV = 1 | 0.787479783 | 0.00512535 | Down |
|  | A0A1V2GF16 | BXT93_12685 | Iron-sulfur cluster assembly scaffold protein IscU (Fragment) OS = *Escherichia coli* OX = 562 GN = BXT93_12685 PE = 3 SV = 1 | 0.78679281 | 0.005360602 | Down |
|  | A0A377KA41 | *ybeD* | Protein YbeD OS = *Escherichia coli* OX = 562 GN = ybeD PE = 3 SV = 1 | 0.786366391 | 0.003273745 | Down |
|  | A0A1M0D457 | *ycbX* | 6-N-hydroxylaminopurine resistance protein YcbX OS = *Escherichia coli* OX = 562 GN = ycbX PE = 4 SV = 1 | 0.786348959 | 0.004438 | Down |
|  | B7UFB0 | *gatB* | Galactitol-specific enzyme IIB component of PTS OS = *Escherichia coli* O127 : H6 (strain E2348/69 / EPEC) OX = 574521 GN = gatB PE = 4 SV = 1 | 0.785947937 | 0.041559838 | Down |
|  | A0A0K4UB01 | *msbA* | Lipid A ABC transporter ATP-binding protein/permease MsbA OS = *Escherichia coli* OX = 562 GN = msbA PE = 4 SV = 1 | 0.785922015 | 0.047992717 | Down |
|  | A0A6D0LQA8 | *yajO* | 1-deoxyxylulose-5-phosphate synthase YajO OS = *Escherichia coli* OX = 562 GN = yajO PE = 4 SV = 1 | 0.784935241 | 0.018224152 | Down |
|  | A0A3L0VZ91 | *pnp* | Polyribonucleotide nucleotidyltransferase OS = *Escherichia coli* OX = 562 GN = pnp PE = 3 SV = 1 | 0.784896011 | 0.046173734 | Down |
|  | A0A271QRD0 | *ileS* | Isoleucine--tRNA ligase OS = *Escherichia coli* OX = 562 GN = ileS PE = 3 SV = 1 | 0.783632529 | 0.014618703 | Down |
|  | A0A3L0VV53 | *aceE* | Pyruvate dehydrogenase E1 component OS = *Escherichia coli* OX = 562 GN = aceE PE = 4 SV = 1 | 0.7834573 | 0.013165863 | Down |
|  | A0A3L0VT52 | *lon* | Lon protease OS = *Escherichia coli* OX = 562 GN = lon PE = 2 SV = 1 | 0.783267958 | 0.013410618 | Down |
|  | A0A0H2V4K9 | *pepD* | Aminoacyl-histidine dipeptidase OS = *Escherichia coli* O6 : H1 (strain CFT073 / ATCC 700928 / UPEC) OX = 199310 GN = pepD PE = 4 SV = 1 | 0.781909708 | 0.021017325 | Down |
|  | A0A0K4GBA4 | *glpE* | Thiosulfate sulfurtransferase GlpE OS = *Escherichia coli* OX = 562 GN = glpE PE = 3 SV = 1 | 0.781833553 | 0.025376125 | Down |
|  | A0A6P1KG06 | *proP* | Glycine betaine/L-proline transporter ProP OS = *Escherichia coli* OX = 562 GN = proP PE = 3 SV = 1 | 0.781561026 | 0.008530838 | Down |
|  | A0A7L7E9P5 | *pepN* | Aminopeptidase N OS = *Escherichia coli* OX = 562 GN = pepN PE = 3 SV = 1 | 0.781467237 | 0.007493549 | Down |
|  | L3CFU3 | A13W_00233 | Glutaredoxin-2 OS = *Escherichia coli* KTE193 OX = 1181739 GN = A13W_00233 PE = 4 SV = 1 | 0.78036991 | 0.007680003 | Down |
|  | A0A826RU97 | FPS82_07065 | Aldo/keto reductase family oxidoreductase OS = *Escherichia coli* OX = 562 GN = FPS82_07065 PE = 4 SV = 1 | 0.78010489 | 0.000312609 | Down |
|  | A0A2H9EUJ5 | *ndk* | Nucleoside diphosphate kinase OS = *Escherichia coli* OX = 562 GN = ndk PE = 3 SV = 1 | 0.779683532 | 0.044519055 | Down |
|  | A0A2H9EVR4 | *aceE* | Pyruvate dehydrogenase E1 component OS = *Escherichia coli* OX = 562 GN = aceE PE = 4 SV = 1 | 0.779512076 | 0.001113327 | Down |
|  | F4VK71 | *asd* | Aspartate-semialdehyde dehydrogenase OS = *Escherichia coli* H591 OX = 656408 GN = asd PE = 3 SV = 1 | 0.779233347 | 0.000532385 | Down |
|  | A0A828FPP9 | AAS29_000306 | Non-heme ferritin-like protein OS = *Escherichia coli* OX = 562 GN = AAS29_000306 PE = 4 SV = 1 | 0.779079406 | 0.004392325 | Down |
|  | A0A6A0Q765 | *malK* | Maltose/maltodextrin ABC transporter ATP-binding protein MalK OS = *Escherichia coli* OX = 562 GN = malK PE = 4 SV = 1 | 0.7789912 | 0.011586282 | Down |
|  | S1ECA0 | A1UI_03248 | DUF927 domain-containing protein OS = *Escherichia coli* KTE73 OX = 1182680 GN = A1UI_03248 PE = 4 SV = 1 | 0.778510283 | 0.006657457 | Down |
|  | P42616 | *yqjC* | Protein YqjC OS = *Escherichia coli* (strain K12) OX = 83333 GN = yqjC PE = 1 SV = 3 | 0.778485829 | 0.01967837 | Down |
|  | A0A2S8JN98 | *pepD* | Aminoacyl-histidine dipeptidase OS = *Escherichia coli* OX = 562 GN = pepD PE = 4 SV = 1 | 0.778225446 | 0.035340958 | Down |
|  | A0A377BMY6 | *yecR* | Protein OS = *Escherichia coli* OX = 562 GN = yecR PE = 4 SV = 1 | 0.777793174 | 0.003069054 | Down |
|  | Q8X5W6 | *malM* | Periplasmic protein of mal regulon OS = *Escherichia coli* O157 : H7 OX = 83334 GN = malM PE = 4 SV = 2 | 0.77741701 | 0.001943874 | Down |
|  | P69229 | *baeR* | Transcriptional regulatory protein BaeR OS = *Escherichia coli* O6 : H1 (strain CFT073 / ATCC 700928 / UPEC) OX = 199310 GN = baeR PE = 1 SV = 1 | 0.777398878 | 0.00047985 | Down |
|  | A0A828B9X1 | *nudK* | GDP-mannose pyrophosphatase NudK OS = *Escherichia coli* OX = 562 GN = nudK PE = 4 SV = 1 | 0.777052016 | 0.000540491 | Down |
|  | A0A0K5M4M1 | *pmbA* | Metalloprotease PmbA OS = *Escherichia coli* OX = 562 GN = pmbA PE = 3 SV = 1 | 0.776069194 | 0.029802002 | Down |
|  | A0A6D0G0H1 | GP711_16415 | Flagellar hook protein FlgE OS = *Escherichia coli* OX = 562 GN = GP711_16415 PE = 3 SV = 1 | 0.775953466 | 0.019601987 | Down |
|  | W1X0Z6 | Q609_ECAC01641G0005 | PTS system maltose-and glucose-specific EIICB component OS = *Escherichia coli* DORA_A_5_14_21 OX = 1403943 GN = Q609_ECAC01641G0005 PE = 4 SV = 1 | 0.775871904 | 0.013365713 | Down |
|  | A0A831DZJ5 | HMV95_06490 | Peptidylprolyl isomerase OS = *Escherichia coli* OX = 562 GN = HMV95_06490 PE = 4 SV = 1 | 0.775691675 | 0.023175792 | Down |
|  | A0A029HTA9 | *rcsB* | Capsular synthesis regulator component B OS = *Escherichia coli* 2-005-03_S4_C3 OX = 1444258 GN = rcsB PE = 4 SV = 1 | 0.775192858 | 0.001842976 | Down |
|  | A0A1M2EKK4 | BK375_23085 | DrsE domain-containing protein OS = *Escherichia coli* OX = 562 GN = BK375_23085 PE = 4 SV = 1 | 0.775164984 | 0.00670834 | Down |
|  | D8A1D9 | HMPREF9530_00303 | Toxin-antitoxin system, toxin component, Fic domain protein OS = *Escherichia coli* (strain MS 21-1) OX = 749527 GN = HMPREF9530_00303 PE = 4 SV = 1 | 0.774427453 | 0.004769555 | Down |
|  | A0A660HCD7 | A9X72_10295 | Ferritin OS = *Escherichia coli* OX = 562 GN = A9X72_10295 PE = 3 SV = 1 | 0.774336342 | 0.004683572 | Down |
|  | A0A7H9QNW5 | *pepN* | Aminopeptidase N OS = *Escherichia coli* OX = 562 GN = pepN PE = 3 SV = 1 | 0.774250375 | 0.003778671 | Down |
|  | A0A7Z8HVR4 | *nifJ* | Pyruvate : ferredoxin (Flavodoxin) oxidoreductase OS = *Escherichia coli* OX = 562 GN = nifJ PE = 3 SV = 1 | 0.773557107 | 0.009754084 | Down |
|  | A0A6C9XEF8 | GP906_06025 | YjjI family glycine radical enzyme OS = *Escherichia coli* OX = 562 GN = GP906_06025 PE = 4 SV = 1 | 0.772866356 | 0.01514467 | Down |
|  | P75726 | *citF* | Citrate lyase alpha chain OS = *Escherichia coli* (strain K12) OX = 83333 GN = citF PE = 2 SV = 1 | 0.772124315 | 0.039397152 | Down |
|  | A0A6L4XG12 | *fbaA* | Fructose-bisphosphate aldolase OS = *Escherichia coli* OX = 562 GN = fbaA PE = 3 SV = 1 | 0.770715682 | 0.022657362 | Down |
|  | A0A3K2YEL7 | D9J61_17275 | 2-oxoglutarate reductase OS = *Escherichia coli* OX = 562 GN = D9J61_17275 PE = 3 SV = 1 | 0.770115306 | 0.003061257 | Down |
|  | B6HZL7 | *nusB* | Transcription antitermination protein NusB OS = *Escherichia coli* (strain SE11) OX = 409438 GN = nusB PE = 3 SV = 1 | 0.769790647 | 0.000812919 | Down |
|  | A0A3Q0MYR4 | CR539_12755 | Oxidoreductase OS = *Escherichia coli* OX = 562 GN = CR539_12755 PE = 4 SV = 1 | 0.76944572 | 0.043582462 | Down |
|  | A0A6M1HI81 | *ruvX* | Putative pre-16S rRNA nuclease OS = *Escherichia coli* OX = 562 GN = ruvX PE = 3 SV = 1 | 0.768863693 | 0.000759696 | Down |
|  | A0A6N8Q549 | GRW77_32280 | Tagatose-bisphosphate aldolase subunit GatZ (Fragment) OS = *Escherichia coli* OX = 562 GN = GRW77_32280 PE = 4 SV = 1 | 0.768749542 | 0.006760432 | Down |
|  | A0A2X1PFM4 | *ygiB* | UPF0441 protein YgiB OS = *Escherichia coli* OX = 562 GN = ygiB PE = 3 SV = 1 | 0.768150815 | 0.008176022 | Down |
|  | A0A1X3LY07 | EAZG_01691 | Putative oxidoreductase Fe-S subunit OS = *Escherichia coli* TA249 OX = 656441 GN = EAZG_01691 PE = 4 SV = 1 | 0.768037888 | 0.029473705 | Down |
|  | A0A827BBU1 | *gatZ* | Tagatose-bisphosphate aldolase subunit GatZ OS = *Escherichia coli* OX = 562 GN = gatZ PE = 4 SV = 1 | 0.767875861 | 0.000897895 | Down |
|  | A0A2Y8JQV3 | *ycjG* | Dipeptide epimerase OS = *Escherichia coli* OX = 562 GN = ycjG PE = 3 SV = 1 | 0.767621537 | 0.003129731 | Down |
|  | A0A827VIM6 | *yeiB* | DUF418 domain-containing protein OS = *Escherichia coli* OX = 562 GN = yeiB PE = 4 SV = 1 | 0.767578345 | 0.016853987 | Down |
|  | A9X4P5 | *gyrB* | DNA topoisomerase (ATP-hydrolyzing) (Fragment) OS = *Escherichia coli* OX = 562 GN = gyrB PE = 3 SV = 1 | 0.767180114 | 0.013324213 | Down |
|  | A0A827CGX5 | *nemA* | N-ethylmaleimide reductase OS = *Escherichia coli* OX = 562 GN = nemA PE = 4 SV = 1 | 0.76713655 | 0.010789294 | Down |
|  | A0A828HF64 | *gatB* | PTS galactitol transporter subunit IIB OS = *Escherichia coli* OX = 562 GN = gatB PE = 4 SV = 1 | 0.767060216 | 0.000336116 | Down |
|  | E6BD97 | *galE* | UDP-glucose 4-epimerase OS = *Escherichia coli* MS 85-1 OX = 679202 GN = galE PE = 3 SV = 1 | 0.766718905 | 0.016278867 | Down |
|  | A0A0E1T1H7 | *caiB* | L-carnitine CoA-transferase OS = *Escherichia coli* 53638 OX = 344610 GN = caiB PE = 3 SV = 1 | 0.765965258 | 0.004351732 | Down |
|  | A0A376SXV3 | *rraA* | Regulator of ribonuclease activity A OS = *Escherichia coli* OX = 562 GN = rraA PE = 3 SV = 1 | 0.764585537 | 0.042847873 | Down |
|  | A0A826TRD1 | *efp* | Elongation factor P OS = *Escherichia coli* OX = 562 GN = efp PE = 4 SV = 1 | 0.763548999 | 0.005994448 | Down |
|  | P64476 | *ydiH* | Uncharacterized protein YdiH OS = *Escherichia coli* (strain K12) OX = 83333 GN = ydiH PE = 4 SV = 2 | 0.76349661 | 0.001389767 | Down |
|  | A0A377FA98 | NCTC9706_00114 | IS186, transposase OS = *Escherichia coli* OX = 562 GN = NCTC9706_00114 PE = 4 SV = 1 | 0.762750161 | 0.000574501 | Down |
|  | A0A6D0F4E6 | GQM06_24570 | TIM barrel protein (Fragment) OS = *Escherichia coli* OX = 562 GN = GQM06_24570 PE = 4 SV = 1 | 0.761484704 | 0.042808759 | Down |
|  | A0A827HIH8 | F9V22_07280 | D-amino acid dehydrogenase OS = *Escherichia coli* OX = 562 GN = F9V22_07280 PE = 4 SV = 1 | 0.761200321 | 0.009841408 | Down |
|  | A0A826X6Z1 | *mepS* | Bifunctional murein DD-endopeptidase/murein LD-carboxypeptidase OS = *Escherichia coli* OX = 562 GN = mepS PE = 4 SV = 1 | 0.76039391 | 0.011199236 | Down |
|  | Q8XDE8 | *pncB* | Nicotinate phosphoribosyltransferase OS = *Escherichia coli* O157 : H7 OX = 83334 GN = pncB PE = 3 SV = 3 | 0.760090256 | 0.003926354 | Down |
|  | A0A2T1LHJ7 | *iscA* | Iron-binding protein IscA OS = *Escherichia coli* OX = 562 GN = iscA PE = 3 SV = 1 | 0.759436569 | 9.0682E-05 | Down |
|  | A0A837MF64 | SM09_00856 | Pyruvate dehydrogenase E1 component OS = *Escherichia coli* OX = 562 GN = SM09_00856 PE = 4 SV = 1 | 0.75935358 | 0.000172596 | Down |
|  | A0A7H9LPC7 | *tsaA* | tRNA (N6-threonylcarbamoyladenosine(37)-N6)-methyltransferase TrmO OS = *Escherichia coli* OX = 562 GN = tsaA PE = 4 SV = 1 | 0.75906983 | 0.033877623 | Down |
|  | P15977 | *malQ* | 4-alpha-glucanotransferase OS = *Escherichia coli* (strain K12) OX = 83333 GN = malQ PE = 1 SV = 2 | 0.759064061 | 0.045587465 | Down |
|  | A0A2J1DEI0 | *malP* | Alpha-1,4 glucan phosphorylase OS = *Escherichia coli* OX = 562 GN = malP PE = 3 SV = 1 | 0.757691331 | 0.007617531 | Down |
|  | A0A6M0PV01 | *atpD* | ATP synthase subunit beta OS = *Escherichia coli* OX = 562 GN = atpD PE = 3 SV = 1 | 0.757602067 | 0.023182842 | Down |
|  | A0A4Y8FSS6 | BON92_21880 | ATP-dependent Clp protease ATP-binding subunit ClpA OS = *Escherichia coli* OX = 562 GN = BON92_21880 PE = 3 SV = 1 | 0.757184617 | 0.002730531 | Down |
|  | D3QQ79 | *rne* | Ribonuclease E OS = *Escherichia coli* O55 : H7 (strain CB9615 / EPEC) OX = 701177 GN = rne PE = 3 SV = 1 | 0.756233164 | 0.032398459 | Down |
|  | A0A6D0ISL3 | *kdsA* | 2-dehydro-3-deoxyphosphooctonate aldolase OS = *Escherichia coli* OX = 562 GN = kdsA PE = 3 SV = 1 | 0.756033236 | 0.003233144 | Down |
|  | A0A7I7HZN9 | *citD* | Citrate lyase acyl carrier protein OS = *Escherichia coli* OX = 562 GN = citD PE = 4 SV = 1 | 0.755440914 | 0.044821388 | Down |
|  | A0A828S3C4 | *rpoN* | RNA polymerase sigma-54 factor OS = *Escherichia coli* STEC_7v OX = 754082 GN = rpoN PE = 4 SV = 1 | 0.755166848 | 0.010729999 | Down |
|  | A0A0K3QKD8 | *tpiA* | Triosephosphate isomerase OS = *Escherichia coli* OX = 562 GN = tpiA PE = 3 SV = 1 | 0.754335504 | 0.02101734 | Down |
|  | A0A829KPZ7 | HMPREF1602_05499 | Superoxide dismutase OS = *Escherichia coli* 907889 OX = 1268989 GN = HMPREF1602_05499 PE = 4 SV = 1 | 0.754029863 | 0.029968371 | Down |
|  | A0A8A8PXR3 | *aspA* | Aspartate ammonia-lyase OS = *Escherichia coli* OX = 562 GN = aspA PE = 4 SV = 1 | 0.753840898 | 0.046959966 | Down |
|  | A0A2X1N3J8 | *aspA* | Aspartate ammonia-lyase OS = *Escherichia coli* OX = 562 GN = aspA PE = 3 SV = 1 | 0.753728468 | 0.013306536 | Down |
|  | A0A4P0YY48 | *pepD_1* | Aminoacyl-histidine dipeptidase OS = *Escherichia coli* OX = 562 GN = pepD_1 PE = 4 SV = 1 | 0.752979632 | 0.001608221 | Down |
|  | Q9R5A8 | unknow | Ferric enterobactin protein receptor (Fragment) OS = *Escherichia coli* OX = 562 PE = 1 SV = 1 | 0.752772772 | 0.023129982 | Down |
|  | A0A829DGU5 | EC2875150_2933 | Haloacid dehalogenase-like hydrolase family protein OS = *Escherichia coli* 2875150 OX = 1116036 GN = EC2875150_2933 PE = 4 SV = 1 | 0.751859476 | 0.004865562 | Down |
|  | A0A828A1X0 | *evgA* | Acid-sensing system DNA-binding response regulator EvgA OS = *Escherichia coli* OX = 562 GN = evgA PE = 4 SV = 1 | 0.751377153 | 0.000992297 | Down |
|  | A0A7U9LV71 | *ydhW* | Uncharacterized protein OS = *Escherichia coli* O145 : H28 OX = 1078034 GN = ydhW PE = 4 SV = 1 | 0.750459776 | 0.016339547 | Down |
|  | A0A831B9J4 | HJS37_002968 | TIGR01620 family protein OS = *Escherichia coli* OX = 562 GN = HJS37_002968 PE = 4 SV = 1 | 0.749986693 | 0.000315778 | Down |
|  | A0A5F1DM40 | CQB02_21170 | Maltose/maltodextrin transport system permease protein OS = *Escherichia coli* OX = 562 GN = CQB02_21170 PE = 3 SV = 1 | 0.747813349 | 0.004082809 | Down |
|  | A0A0H3EKR0 | NRG857_10630 | Galactitol-specific PTS system component IIA OS = *Escherichia coli* O83 : H1 (strain NRG 857C / AIEC) OX = 685038 GN = NRG857_10630 PE = 4 SV = 1 | 0.747590835 | 0.002397406 | Down |
|  | P0A7M2 | *rpmB* | 50S ribosomal protein L28 OS = *Escherichia coli* (strain K12) OX = 83333 GN = rpmB PE = 1 SV = 2 | 0.747474531 | 0.006581886 | Down |
|  | V0SH21 | HMPREF1595_03595 | Putative selenate reductase, YgfK subunit OS = *Escherichia coli* 907672 OX = 1268982 GN = HMPREF1595_03595 PE = 4 SV = 1 | 0.747398483 | 0.001558842 | Down |
|  | A0A4Y9XHJ6 | *gatZ* | D-tagatose-1,6-bisphosphate aldolase subunit GatZ OS = *Escherichia coli* OX = 562 GN = gatZ PE = 3 SV = 1 | 0.747291697 | 0.023129704 | Down |
|  | A0A2T1LCH0 | C6985_23965 | Cold-shock protein OS = *Escherichia coli* OX = 562 GN = C6985_23965 PE = 4 SV = 1 | 0.747162889 | 0.005104446 | Down |
|  | U9Z5W5 | HMPREF1589_00327 | Uncharacterized protein OS = *Escherichia coli* 113290 OX = 1268976 GN = HMPREF1589_00327 PE = 4 SV = 1 | 0.745830023 | 0.015571765 | Down |
|  | A0A5N8HAY0 | *fumD* | Fumarase D OS = *Escherichia coli* OX = 562 GN = fumD PE = 3 SV = 1 | 0.745562014 | 0.009533048 | Down |
|  | F4SVE4 | ECIG_00164 | Cold shock protein OS = *Escherichia coli* M605 OX = 656417 GN = ECIG_00164 PE = 4 SV = 1 | 0.744683821 | 0.013017073 | Down |
|  | A0A2T1LK83 | C6985_09530 | Superoxide dismutase OS = *Escherichia coli* OX = 562 GN = C6985_09530 PE = 3 SV = 1 | 0.74455815 | 0.012783209 | Down |
|  | A0A2T1LKD4 | C6985_09835 | Aldehyde ferredoxin oxidoreductase OS = *Escherichia coli* OX = 562 GN = C6985_09835 PE = 3 SV = 1 | 0.74404942 | 0.009372011 | Down |
|  | A0A376HGP0 | *tppB* | Dipeptide and tripeptide permease A OS = *Escherichia coli* OX = 562 GN = tppB PE = 3 SV = 1 | 0.743357712 | 0.001904756 | Down |
|  | A0A6S4W2J6 | WP2S18E08_47150 | DNA helicase OS = *Escherichia coli* OX = 562 GN = WP2S18E08_47150 PE = 3 SV = 1 | 0.743055115 | 0.026475801 | Down |
|  | A0A288XG28 | DL654_25255 | Arabinose 5-phosphate isomerase OS = *Escherichia coli* OX = 562 GN = DL654_25255 PE = 3 SV = 1 | 0.740712225 | 0.035274012 | Down |
|  | W1ETG5 | unknow | Uncharacterized protein OS = *Escherichia coli* ISC7 OX = 1432555 PE = 4 SV = 1 | 0.74002701 | 0.008119956 | Down |
|  | A0A826L929 | *proV* | Glycine betaine/L-proline ABC transporter ATP-binding protein ProV OS = *Escherichia coli* OX = 562 GN = proV PE = 4 SV = 1 | 0.739360062 | 0.02314386 | Down |
|  | P0AB72 | *fbaA* | Fructose-bisphosphate aldolase class 2 OS = *Escherichia coli* O157 : H7 OX = 83334 GN = fbaA PE = 3 SV = 2 | 0.739276186 | 0.002590822 | Down |
|  | A0A7B4HUY5 | HMV41_25160 | NCS2 family permease (Fragment) OS = *Escherichia coli* OX = 562 GN = HMV41_25160 PE = 4 SV = 1 | 0.737709891 | 0.017605978 | Down |
|  | A0A6D0GUE7 | *crl* | Sigma factor-binding protein Crl OS = *Escherichia coli* OX = 562 GN = crl PE = 3 SV = 1 | 0.737489743 | 0.000216988 | Down |
|  | A0A2T1LIJ7 | C6985_13710 | SLC13 family permease OS = *Escherichia coli* OX = 562 GN = C6985_13710 PE = 4 SV = 1 | 0.736371893 | 0.005668731 | Down |
|  | A0A6M0PZE3 | *accC* | Biotin carboxylase OS = *Escherichia coli* OX = 562 GN = accC PE = 4 SV = 1 | 0.734190689 | 0.004342528 | Down |
|  | E6BPP9 | *proP* | Proline porter II OS = *Escherichia coli* MS 85-1 OX = 679202 GN = proP PE = 3 SV = 1 | 0.734118182 | 0.010504765 | Down |
|  | A0A6M7GT65 | E3158_01100 | Glutathione S-transferase OS = *Escherichia coli* O157 : H7 OX = 83334 GN = E3158_01100 PE = 4 SV = 1 | 0.734014093 | 0.036269096 | Down |
|  | Q8XE60 | *lon* | Lon protease OS = *Escherichia coli* O157 : H7 OX = 83334 GN = lon PE = 2 SV = 2 | 0.733939357 | 0.000277809 | Down |
|  | A0A6M0PZR8 | *fis* | DNA-binding protein Fis OS = *Escherichia coli* OX = 562 GN = fis PE = 3 SV = 1 | 0.731982982 | 0.016680265 | Down |
|  | A0A0V7WK93 | *narH* | Nitrate reductase subunit beta OS = *Escherichia coli* OX = 562 GN = narH PE = 4 SV = 1 | 0.731035778 | 0.043083692 | Down |
|  | A0A377BVZ1 | *narG* | Nitrate reductase (quinone) OS = *Escherichia coli* OX = 562 GN = narG PE = 3 SV = 1 | 0.730681813 | 0.0382329 | Down |
|  | A0A6M0PZM1 | *csrA* | Translational regulator CsrA OS = *Escherichia coli* OX = 562 GN = csrA PE = 3 SV = 1 | 0.729775323 | 0.000742527 | Down |
|  | A0A6D0FFI4 | GQM06_36645 | DUF3459 domain-containing protein (Fragment) OS = *Escherichia coli* OX = 562 GN = GQM06_36645 PE = 3 SV = 1 | 0.729754851 | 0.036780639 | Down |
|  | A0A891ZVH5 | unknow | Uncharacterized protein OS = *Escherichia coli* OX = 562 PE = 4 SV = 1 | 0.729537652 | 0.046467965 | Down |
|  | A0A826Q325 | *citE* | Citrate (Pro-3S)-lyase subunit beta OS = *Escherichia coli* OX = 562 GN = citE PE = 4 SV = 1 | 0.729505515 | 0.01882812 | Down |
|  | A0A828URM2 | *gcvH* | Glycine cleavage system H protein OS = *Escherichia coli* 3.2608 OX = 869679 GN = gcvH PE = 4 SV = 1 | 0.729393054 | 0.045100857 | Down |
|  | A0A7I9ASU8 | ETECE925_04375 | Dynamin-type G domain-containing protein OS = *Escherichia coli* OX = 562 GN = ETECE925_04375 PE = 4 SV = 1 | 0.728966633 | 0.006914565 | Down |
|  | A0A1M2GPQ1 | *galK* | Galactokinase OS = *Escherichia coli* OX = 562 GN = galK PE = 3 SV = 1 | 0.728814986 | 0.004785816 | Down |
|  | A0A271QLV6 | *yaiI* | UPF0178 protein YaiI OS = *Escherichia coli* OX = 562 GN = yaiI PE = 3 SV = 1 | 0.726227429 | 0.014526226 | Down |
|  | A0A831FLZ4 | *lon* | Endopeptidase La OS = *Escherichia coli* OX = 562 GN = lon PE = 4 SV = 1 | 0.725216538 | 0.008736674 | Down |
|  | A0A5F1DL70 | *hslU* | ATP-dependent protease ATPase subunit HslU OS = *Escherichia coli* OX = 562 GN = hslU PE = 2 SV = 1 | 0.724294932 | 0.001537585 | Down |
|  | A0A827G2Z0 | *alsR* | Als operon DNA-binding transcriptional repressor AlsR OS = *Escherichia coli* OX = 562 GN = alsR PE = 4 SV = 1 | 0.723924015 | 0.008244473 | Down |
|  | J7QSF1 | *fliO* | Flagellar protein OS = *Escherichia coli* OX = 562 GN = fliO PE = 3 SV = 1 | 0.723913739 | 0.000804538 | Down |
|  | A0A844EWT9 | *uspE* | Universal stress protein UspE OS = *Escherichia coli* OX = 562 GN = uspE PE = 4 SV = 1 | 0.720614541 | 0.049779077 | Down |
|  | A0A831DIL6 | HMU48_28895 | RNA chaperone Hfq OS = *Escherichia coli* OX = 562 GN = HMU48_28895 PE = 4 SV = 1 | 0.720306059 | 0.008627379 | Down |
|  | A0A6N7KEC5 | *dmlA* | D-malate dehydrogenase [decarboxylating] OS = *Escherichia coli* OX = 562 GN = dmlA PE = 3 SV = 1 | 0.71950021 | 0.004103782 | Down |
|  | A0A826X8H6 | CSE52_003051 | YchJ family protein OS = *Escherichia coli* OX = 562 GN = CSE52_003051 PE = 4 SV = 1 | 0.719135706 | 0.018490346 | Down |
|  | V0TDJ1 | HMPREF1595_01611 | Glycine betaine/L-proline transport system permease protein ProW OS = *Escherichia coli* 907672 OX = 1268982 GN = HMPREF1595_01611 PE = 3 SV = 1 | 0.718095197 | 0.016036012 | Down |
|  | A0A6M1I0C1 | G5632_32545 | Aldo/keto reductase (Fragment) OS = *Escherichia coli* OX = 562 GN = G5632_32545 PE = 4 SV = 1 | 0.715337113 | 0.005673733 | Down |
|  | A0A5C9A7M4 | *dinD* | DNA damage-inducible protein D (Fragment) OS = *Escherichia coli* OX = 562 GN = dinD PE = 4 SV = 1 | 0.715050626 | 4.62899E-05 | Down |
|  | A0A1V3W1U0 | BMT91_06785 | Transposase OS = *Escherichia coli* OX = 562 GN = BMT91_06785 PE = 3 SV = 1 | 0.714065498 | 0.007962694 | Down |
|  | A0A810TKM9 | *chbF* | 6-phospho-beta-glucosidase OS = *Escherichia coli* OX = 562 GN = chbF PE = 4 SV = 1 | 0.712829536 | 0.037225552 | Down |
|  | A0A383G4P5 | *ppc* | Phosphoenolpyruvate carboxylase OS = *Escherichia coli* OX = 562 GN = ppc PE = 3 SV = 1 | 0.71245723 | 0.000568982 | Down |
|  | A0A6D0ILH3 | *citF* | Citrate lyase alpha chain OS = *Escherichia coli* OX = 562 GN = citF PE = 4 SV = 1 | 0.71210541 | 0.016714121 | Down |
|  | A0A7U2A5P4 | *dcuA* | Anaerobic C4-dicarboxylate transporter OS = *Escherichia coli* OX = 562 GN = dcuA PE = 3 SV = 1 | 0.710973103 | 0.002064916 | Down |
|  | A0A4V3QD96 | *crl* | Sigma factor-binding protein Crl OS = *Escherichia coli* OX = 562 GN = crl PE = 3 SV = 1 | 0.710592423 | 0.014299208 | Down |
|  | A0A6L4XKB9 | *iscA* | Iron-binding protein IscA OS = *Escherichia coli* OX = 562 GN = iscA PE = 3 SV = 1 | 0.710450756 | 0.002259417 | Down |
|  | P76046 | *ycjX* | Uncharacterized protein YcjX OS = *Escherichia coli* (strain K12) OX = 83333 GN = ycjX PE = 1 SV = 1 | 0.708171721 | 0.00020054 | Down |
|  | A0A376VU06 | *tufA_2* | Elongation factor Tu OS = *Escherichia coli* OX = 562 GN = tufA_2 PE = 4 SV = 1 | 0.707694506 | 0.019060536 | Down |
|  | Q6JZC5 | *trpB* | Tryptophan synthase beta chain (Fragment) OS = *Escherichia coli* OX = 562 GN = trpB PE = 3 SV = 1 | 0.704242926 | 2.284E-05 | Down |
|  | A0A377CXM6 | *folK_1* | 2-amino-4-hydroxy-6-hydroxymethyldihydropteridine pyrophosphokinase OS = *Escherichia coli* OX = 562 GN = folK_1 PE = 3 SV = 1 | 0.701798288 | 0.000239481 | Down |
|  | A0A827ABQ5 | CN875_002057 | NADP(H)-dependent aldo-keto reductase OS = *Escherichia coli* OX = 562 GN = CN875_002057 PE = 4 SV = 1 | 0.701665194 | 0.007738009 | Down |
|  | A0A271R642 | BIZ41_06685 | Universal stress protein UspE OS = *Escherichia coli* OX = 562 GN = BIZ41_06685 PE = 4 SV = 1 | 0.700551168 | 0.002133531 | Down |
|  | A0A7H9LUQ5 | *pdxY* | Pyridoxal kinase PdxY OS = *Escherichia coli* OX = 562 GN = pdxY PE = 3 SV = 1 | 0.699991348 | 0.003919555 | Down |
|  | A0A768EGW4 | HMT45_14985 | PTS galactitol transporter subunit IIA OS = *Escherichia coli* OX = 562 GN = HMT45_14985 PE = 4 SV = 1 | 0.699849576 | 0.001465313 | Down |
|  | A0A346GGF1 | *rihA* | Pyrimidine-specific ribonucleoside hydrolase RihA OS = *Escherichia coli* OX = 562 GN = rihA PE = 3 SV = 1 | 0.69898821 | 0.007645746 | Down |
|  | A0A2H3MBD6 | CO706_08145 | Galactitol-1-phosphate 5-dehydrogenase OS = *Escherichia coli* OX = 562 GN = CO706_08145 PE = 3 SV = 1 | 0.698492042 | 0.010216757 | Down |
|  | U9XK73 | HMPREF1589_05182 | Hemolysin expression-modulating protein OS = *Escherichia coli* 113290 OX = 1268976 GN = HMPREF1589_05182 PE = 3 SV = 1 | 0.697610804 | 0.008326301 | Down |
|  | P39384 | *yjiM* | Putative dehydratase subunit YjiM OS = *Escherichia coli* (strain K12) OX = 83333 GN = yjiM PE = 3 SV = 2 | 0.696512847 | 0.003104838 | Down |
|  | A0A0H3EIE9 | *znuA* | High-affinity zinc uptake system protein ZnuA OS = *Escherichia coli* O83 : H1 (strain NRG 857C / AIEC) OX = 685038 GN = znuA PE = 3 SV = 1 | 0.696275863 | 0.000477864 | Down |
|  | V0YJG2 | HMPREF1611_03527 | Isoaspartyl dipeptidase OS = *Escherichia coli* 908573 OX = 1268998 GN = HMPREF1611_03527 PE = 3 SV = 1 | 0.69564014 | 0.00207599 | Down |
|  | Q6JZ44 | *trpA* | Tryptophan synthase (Fragment) OS = *Escherichia coli* OX = 562 GN = trpA PE = 3 SV = 1 | 0.695360431 | 0.017630712 | Down |
|  | A0A7H9LRW3 | *cobO* | Corrinoid adenosyltransferase OS = *Escherichia coli* OX = 562 GN = cobO PE = 3 SV = 1 | 0.694472878 | 0.009669984 | Down |
|  | A0A777CE63 | *rhlE* | ATP-dependent RNA helicase RhlE OS = *Escherichia coli* OX = 562 GN = rhlE PE = 3 SV = 1 | 0.692074264 | 0.003946637 | Down |
|  | A0A826XBG0 | *gatB* | PTS galactitol transporter subunit IIB OS = *Escherichia coli* OX = 562 GN = gatB PE = 4 SV = 1 | 0.689759354 | 0.002124493 | Down |
|  | A0A8B5MAI7 | *yeaE* | Methylglyoxal reductase YeaE OS = *Escherichia coli* OX = 562 GN = yeaE PE = 4 SV = 1 | 0.688091771 | 0.000109481 | Down |
|  | A0A8B5MA57 | *gatZ* | Tagatose-bisphosphate aldolase subunit GatZ OS = *Escherichia coli* OX = 562 GN = gatZ PE = 4 SV = 1 | 0.684452691 | 0.000526235 | Down |
|  | A0A830SFJ1 | HHH44_000902 | Ferredoxin-like protein OS = *Escherichia coli* OX = 562 GN = HHH44_000902 PE = 4 SV = 1 | 0.683846975 | 0.007525307 | Down |
|  | P11350 | *narI* | Respiratory nitrate reductase 1 gamma chain OS = *Escherichia coli* (strain K12) OX = 83333 GN = narI PE = 1 SV = 1 | 0.682221316 | 0.037411457 | Down |
|  | A0A6N8QE93 | *pnp* | Polyribonucleotide nucleotidyltransferase OS = *Escherichia coli* OX = 562 GN = pnp PE = 3 SV = 1 | 0.679952887 | 9.41748E-05 | Down |
|  | D6I9G0 | *gatZ* | D-tagatose-1,6-bisphosphate aldolase subunit GatZ OS = *Escherichia coli* B185 OX = 550676 GN = gatZ PE = 3 SV = 1 | 0.678889152 | 0.002316554 | Down |
|  | A0A376VYA0 | *tdcF* | TdcF protein OS = *Escherichia coli* OX = 562 GN = tdcF PE = 3 SV = 1 | 0.678070282 | 0.023663249 | Down |
|  | A0A376HYQ1 | *trpB* | Tryptophan synthase beta chain OS = *Escherichia coli* OX = 562 GN = trpB PE = 3 SV = 1 | 0.677648552 | 0.001108763 | Down |
|  | A0A2T1LH30 | *lexA* | LexA repressor OS = *Escherichia coli* OX = 562 GN = lexA PE = 3 SV = 1 | 0.67683033 | 0.00037671 | Down |
|  | I4T542 | *cdd* | Cytidine deaminase OS = *Escherichia coli* 541-15 OX = 752785 GN = cdd PE = 3 SV = 1 | 0.672937001 | 0.003889238 | Down |
|  | A0A376RE19 | *proX_1* | Glycine betaine/L-proline ABC transporter, substrate-binding protein OS = *Escherichia coli* OX = 562 GN = proX_1 PE = 4 SV = 1 | 0.670907021 | 0.045793361 | Down |
|  | A0A1U9SZS3 | *ribH* | 6,7-dimethyl-8-ribityllumazine synthase OS = *Escherichia coli* OX = 562 GN = ribH PE = 3 SV = 1 | 0.670689946 | 0.00014017 | Down |
|  | A0A376TRN1 | *deaD_1* | ATP-dependent RNA helicase (DEAD-box protein) OS = *Escherichia coli* OX = 562 GN = deaD_1 PE = 4 SV = 1 | 0.670560387 | 0.006667717 | Down |
|  | A0A826WYJ8 | CSE52_000824 | 2-hydroxyacyl-CoA dehydratase subunit D OS = *Escherichia coli* OX = 562 GN = CSE52_000824 PE = 4 SV = 1 | 0.670383996 | 0.011192035 | Down |
|  | A0A5F1E0X3 | *cdd* | Cytidine deaminase OS = *Escherichia coli* OX = 562 GN = cdd PE = 3 SV = 1 | 0.668920853 | 0.001235653 | Down |
|  | A0A827UE97 | *cdd* | Cytidine deaminase OS = *Escherichia coli* OX = 562 GN = cdd PE = 4 SV = 1 | 0.667407912 | 0.003968531 | Down |
|  | A0A7A2V838 | *ppnP* | Pyrimidine/purine nucleoside phosphorylase OS = *Escherichia coli* OX = 562 GN = ppnP PE = 3 SV = 1 | 0.667248171 | 0.001946609 | Down |
|  | A0A3A6SBY8 | BMC79_003960 | OmpA family protein OS = *Escherichia coli* OX = 562 GN = BMC79_003960 PE = 4 SV = 1 | 0.663939145 | 0.001716123 | Down |
|  | A0A0J1XV59 | *infA* | Translation initiation factor IF-1 OS = *Escherichia coli* OX = 562 GN = infA PE = 3 SV = 1 | 0.663810563 | 0.021499851 | Down |
|  | A0A377B2G3 | *cof_1* | HMP-PP phosphatase OS = *Escherichia coli* OX = 562 GN = cof_1 PE = 3 SV = 1 | 0.662134032 | 5.51108E-05 | Down |
|  | A0A4Y8GFI5 | BON92_01645 | Alpha-ketoglutarate transporter OS = *Escherichia coli* OX = 562 GN = BON92_01645 PE = 3 SV = 1 | 0.660300671 | 1.84533E-05 | Down |
|  | A0A478KX94 | BvCmsH19A_01829 | DNA injection protein OS = *Escherichia coli* OX = 562 GN = BvCmsH19A_01829 PE = 4 SV = 1 | 0.660114179 | 0.000408722 | Down |
|  | A0A829FJ04 | WCS_01189 | Nitrite extrusion protein 1 OS = *Escherichia coli* KTE14 OX = 1169333 GN = WCS_01189 PE = 4 SV = 1 | 0.659515532 | 0.004119529 | Down |
|  | A0A799VWC7 | *dcuC* | Anaerobic C4-dicarboxylate transporter DcuC OS = *Escherichia coli* OX = 562 GN = dcuC PE = 3 SV = 1 | 0.65744663 | 0.004246281 | Down |
|  | A0A0E1SXQ0 | *gcvT* | Aminomethyltransferase OS = *Escherichia coli* 53638 OX = 344610 GN = gcvT PE = 3 SV = 1 | 0.656644232 | 0.006466962 | Down |
|  | A0A828S4S8 | *lacF* | Cellobiose-specific phosphotransferase enzyme IIA component OS = *Escherichia coli* STEC_7v OX = 754082 GN = lacF PE = 4 SV = 1 | 0.652181436 | 0.003846529 | Down |
|  | A0A080IBY9 | *lamB* | Maltoporin OS = *Escherichia coli* 1-250-04_S3_C2 OX = 1444163 GN = lamB PE = 2 SV = 1 | 0.649596453 | 0.024470743 | Down |
|  | A0A6M0PTL1 | *trpB* | Tryptophan synthase beta chain OS = *Escherichia coli* OX = 562 GN = trpB PE = 3 SV = 1 | 0.648578188 | 0.013283741 | Down |
|  | P69222 | *infA* | Translation initiation factor IF-1 OS = *Escherichia coli* (strain K12) OX = 83333 GN = infA PE = 1 SV = 2 | 0.645883808 | 0.007921992 | Down |
|  | A0A080IZS0 | AC28_1820 | Aldehyde ferredoxin oxidoreductase, domains 2 & 3 family protein OS = *Escherichia coli* 1-250-04_S3_C2 OX = 1444163 GN = AC28_1820 PE = 3 SV = 1 | 0.645506679 | 0.004949641 | Down |
|  | B1LQY8 | EcSMS35_2935 | AMP nucleosidase OS = *Escherichia coli* (strain SMS-3-5 / SECEC) OX = 439855 GN = EcSMS35_2935 PE = 4 SV = 1 | 0.644832921 | 0.001561242 | Down |
|  | A0A4P0YBN2 | *gatD* | Galactitol-1-phosphate 5-dehydrogenase OS = *Escherichia coli* OX = 562 GN = gatD PE = 3 SV = 1 | 0.643496694 | 0.001870882 | Down |
|  | A0A4Q0BG29 | ELY48_00195 | Thymidylate kinase OS = *Escherichia coli* OX = 562 GN = ELY48_00195 PE = 4 SV = 1 | 0.643167112 | 0.000240027 | Down |
|  | A0A6N8P9J3 | *gatZ* | D-tagatose-1,6-bisphosphate aldolase subunit GatZ OS = *Escherichia coli* OX = 562 GN = gatZ PE = 3 SV = 1 | 0.639101193 | 0.000548457 | Down |
|  | A0A2T1LHC8 | C6985_14235 | Phosphoenolpyruvate-protein phosphotransferase OS = *Escherichia coli* OX = 562 GN = C6985_14235 PE = 3 SV = 1 | 0.632566328 | 0.006395278 | Down |
|  | A0A6M0PQ81 | G4V03_00695 | 4Fe-4S dicluster domain-containing protein OS = *Escherichia coli* OX = 562 GN = G4V03_00695 PE = 4 SV = 1 | 0.631809747 | 0.001101905 | Down |
|  | A0A4V4SBS9 | *tpiA* | Triosephosphate isomerase OS = *Escherichia coli* OX = 562 GN = tpiA PE = 3 SV = 1 | 0.628357018 | 0.038299435 | Down |
|  | A0A826WTR0 | *gatZ* | Tagatose-bisphosphate aldolase subunit GatZ OS = *Escherichia coli* OX = 562 GN = gatZ PE = 4 SV = 1 | 0.627551656 | 0.008519856 | Down |
|  | W1VWC7 | Q609_ECAC02901G0001 | Uncharacterized protein (Fragment) OS = *Escherichia coli* DORA_A_5_14_21 OX = 1403943 GN = Q609_ECAC02901G0001 PE = 4 SV = 1 | 0.627429738 | 0.002945976 | Down |
|  | A0A6L4XIJ4 | GP710_11370 | Nitrate/nitrite transporter OS = *Escherichia coli* OX = 562 GN = GP710_11370 PE = 3 SV = 1 | 0.627169809 | 0.019172626 | Down |
|  | B6I4S5 | *hslV* | ATP-dependent protease subunit HslV OS = *Escherichia coli* (strain SE11) OX = 409438 GN = hslV PE = 3 SV = 1 | 0.626149591 | 0.010349325 | Down |
|  | A0A417ZQI7 | *pspA* | Phage shock protein PspA OS = *Escherichia coli* OX = 562 GN = pspA PE = 3 SV = 1 | 0.623012291 | 0.000284121 | Down |
|  | A0A7A3AF22 | HJ940_004371 | YfeK family protein OS = *Escherichia coli* OX = 562 GN = HJ940_004371 PE = 4 SV = 1 | 0.621895172 | 0.005701382 | Down |
|  | D7Y521 | *pnp* | Polyribonucleotide nucleotidyltransferase OS = *Escherichia coli* (strain MS 115-1) OX = 749537 GN = pnp PE = 3 SV = 1 | 0.621068854 | 0.000166875 | Down |
|  | U9Y2M9 | HMPREF1589_03441 | Fimbrial protein OS = *Escherichia coli* 113290 OX = 1268976 GN = HMPREF1589_03441 PE = 4 SV = 1 | 0.619867185 | 0.002268492 | Down |
|  | W1X365 | Q609_ECAC01377G0008 | D-tagatose-1,6-bisphosphate aldolase subunit kbaZ (Fragment) OS = *Escherichia coli* DORA_A_5_14_21 OX = 1403943 GN = Q609_ECAC01377G0008 PE = 4 SV = 1 | 0.619538375 | 0.000861943 | Down |
|  | B7MEE9 | *gatA* | Galactitol-specific enzyme IIA component of PTS OS = *Escherichia coli* O45 : K1 (strain S88 / ExPEC) OX = 585035 GN = gatA PE = 4 SV = 1 | 0.619407411 | 0.000108114 | Down |
|  | A0A789MBN3 | *gatB* | PTS galactitol transporter subunit IIB OS = *Escherichia coli* OX = 562 GN = gatB PE = 4 SV = 1 | 0.617092407 | 0.023009912 | Down |
|  | P0AB15 | *yccJ* | Uncharacterized protein YccJ OS = *Escherichia coli* O6 : H1 (strain CFT073 / ATCC 700928 / UPEC) OX = 199310 GN = yccJ PE = 4 SV = 1 | 0.616978246 | 0.003396636 | Down |
|  | A0A765T0J1 | *pnp* | Polyribonucleotide nucleotidyltransferase OS = *Escherichia coli* OX = 562 GN = pnp PE = 3 SV = 1 | 0.615684818 | 0.000327801 | Down |
|  | A0A7H9LQH0 | HV146_06765 | PhoH family protein OS = *Escherichia coli* OX = 562 GN = HV146_06765 PE = 3 SV = 1 | 0.609825417 | 0.001358958 | Down |
|  | A0A6D0UCH6 | *gatZ* | D-tagatose-1,6-bisphosphate aldolase subunit GatZ OS = *Escherichia coli* OX = 562 GN = gatZ PE = 3 SV = 1 | 0.606628728 | 0.003175537 | Down |
|  | P52129 | *rnlA* | mRNA endoribonuclease toxin LS OS = *Escherichia coli* (strain K12) OX = 83333 GN = rnlA PE = 1 SV = 2 | 0.605611913 | 0.000893198 | Down |
|  | P06149 | *dld* | Quinone-dependent D-lactate dehydrogenase OS = *Escherichia coli* (strain K12) OX = 83333 GN = dld PE = 1 SV = 3 | 0.600994797 | 0.000480558 | Down |
|  | A0A7H9LVC8 | *gatZ* | D-tagatose-1,6-bisphosphate aldolase subunit GatZ OS = *Escherichia coli* OX = 562 GN = gatZ PE = 3 SV = 1 | 0.600351243 | 0.000413302 | Down |
|  | A0A5F1E0Y2 | CQB02_07555 | Sulfurtransferase OS = *Escherichia coli* OX = 562 GN = CQB02_07555 PE = 4 SV = 1 | 0.597863561 | 0.006399557 | Down |
|  | A0A368IN84 | A6592_18150 | Peptidyl-prolyl cis-trans isomerase OS = *Escherichia coli* OX = 562 GN = A6592_18150 PE = 3 SV = 1 | 0.593267361 | 0.00197564 | Down |
|  | A0A1E5WU20 | DNQ45_24495 | PTS galactitol transporter subunit IIB OS = *Escherichia coli* OX = 562 GN = DNQ45_24495 PE = 4 SV = 1 | 0.589552245 | 0.000451462 | Down |
|  | A0A377CD10 | *gatZ* | D-tagatose-1,6-bisphosphate aldolase subunit GatZ OS = *Escherichia coli* OX = 562 GN = gatZ PE = 3 SV = 1 | 0.589507349 | 3.07482E-05 | Down |
|  | C5A130 | *lamB* | Maltoporin OS = *Escherichia coli* (strain K12 / MC4100 / BW2952) OX = 595496 GN = lamB PE = 3 SV = 1 | 0.589134942 | 0.027513366 | Down |
|  | A0A7L7XED2 | *deaD* | ATP-dependent RNA helicase DeaD OS = *Escherichia coli* O18ac : H14 OX = 2773705 GN = deaD PE = 3 SV = 1 | 0.587020186 | 0.000216127 | Down |
|  | A0A4Y8DX03 | BON63_04935 | SLC13 family permease OS = *Escherichia coli* OX = 562 GN = BON63_04935 PE = 4 SV = 1 | 0.583296278 | 0.020228308 | Down |
|  | E6BK96 | HMPREF9350_02703 | Uncharacterized protein OS = *Escherichia coli* MS 85-1 OX = 679202 GN = HMPREF9350_02703 PE = 4 SV = 1 | 0.582353297 | 0.002039265 | Down |
|  | A0A843NGY2 | *fghA* | S-formylglutathione hydrolase OS = *Escherichia coli* OX = 562 GN = fghA PE = 4 SV = 1 | 0.580692859 | 0.000117079 | Down |
|  | A0A6M0PUX4 | G4V03_05755 | Replication endonuclease OS = *Escherichia coli* OX = 562 GN = G4V03_05755 PE = 3 SV = 1 | 0.579851272 | 0.015123757 | Down |
|  | A0A377FAX0 | *flgD_1* | Basal-body rod modification protein FlgD OS = *Escherichia coli* OX = 562 GN = flgD_1 PE = 3 SV = 1 | 0.578263333 | 0.013593736 | Down |
|  | C0ST84 | *gatA* | Galactitol-specific enzyme IIA component of PTS OS = *Escherichia coli* O55 : H7 OX = 244320 GN = gatA PE = 4 SV = 1 | 0.575745107 | 0.04146617 | Down |
|  | A0A376I7H5 | *mgsA* | Methylglyoxal synthase OS = *Escherichia coli* OX = 562 GN = mgsA PE = 3 SV = 1 | 0.574497076 | 4.61119E-05 | Down |
|  | A0A5D8MVY1 | *deaD* | ATP-dependent RNA helicase DeaD OS = *Escherichia coli* OX = 562 GN = deaD PE = 3 SV = 1 | 0.568756719 | 0.000273948 | Down |
|  | A0A376TD77 | *gatZ_3* | Putative tagatose 6-phosphate kinase OS = *Escherichia coli* OX = 562 GN = gatZ_3 PE = 4 SV = 1 | 0.563924145 | 6.68838E-05 | Down |
|  | A0A641JB40 | *gutM* | Transcriptional regulator GutM OS = *Escherichia coli* OX = 562 GN = gutM PE = 4 SV = 1 | 0.560956496 | 0.017819396 | Down |
|  | A0A8A9FI50 | *gatZ* | Tagatose-bisphosphate aldolase subunit GatZ OS = *Escherichia coli* OX = 562 GN = gatZ PE = 4 SV = 1 | 0.560261545 | 0.000400361 | Down |
|  | A0A0P0SWP2 | *gatZ* | D-tagatose-1,6-bisphosphate aldolase subunit GatZ OS = *Escherichia coli* OX = 562 GN = gatZ PE = 3 SV = 1 | 0.557215714 | 0.000853067 | Down |
|  | A0A6L6RZ23 | *gatZ* | D-tagatose-1,6-bisphosphate aldolase subunit GatZ OS = *Escherichia coli* OX = 562 GN = gatZ PE = 3 SV = 1 | 0.551713047 | 0.000368958 | Down |
|  | A0A7D7I018 | *nemA* | N-ethylmaleimide reductase OS = *Escherichia coli* OX = 562 GN = nemA PE = 4 SV = 1 | 0.5478107 | 0.006872765 | Down |
|  | A0A8A5ITE9 | *yieF* | Class I chromate reductase YieF OS = *Escherichia coli* H20 OX = 2810409 GN = yieF PE = 4 SV = 1 | 0.545600814 | 6.79407E-06 | Down |
|  | A0A6M1HJW3 | G5632_09585 | Aldehyde ferredoxin oxidoreductase OS = *Escherichia coli* OX = 562 GN = G5632_09585 PE = 3 SV = 1 | 0.543764815 | 0.003340581 | Down |
|  | A0A6D0HB74 | *chbB* | PTS N,N'-diacetylchitobiose transporter subunit IIB OS = *Escherichia coli* OX = 562 GN = chbB PE = 4 SV = 1 | 0.541699033 | 0.003305276 | Down |
|  | A0A4Y8FPW6 | BON92_27575 | Cold-shock protein OS = *Escherichia coli* OX = 562 GN = BON92_27575 PE = 4 SV = 1 | 0.539864297 | 0.002353663 | Down |
|  | P06960 | *argF* | Ornithine carbamoyltransferase subunit F OS = *Escherichia coli* (strain K12) OX = 83333 GN = argF PE = 1 SV = 4 | 0.537831426 | 0.001204662 | Down |
|  | A0A6C9TKU4 | *hycE* | Hydrogenase large subunit OS = *Escherichia coli* OX = 562 GN = hycE PE = 4 SV = 1 | 0.536753972 | 0.004260742 | Down |
|  | D3GZF3 | *gatZ* | D-tagatose-1,6-bisphosphate aldolase subunit GatZ OS = *Escherichia coli* O44 : H18 (strain 042 / EAEC) OX = 216592 GN = gatZ PE = 3 SV = 1 | 0.5342803 | 0.00326864 | Down |
|  | A0A5P0JCN2 | *gatZ* | D-tagatose-1,6-bisphosphate aldolase subunit GatZ OS = *Escherichia coli* OX = 562 GN = gatZ PE = 3 SV = 1 | 0.532330637 | 9.02584E-05 | Down |
|  | A0A0J2BU24 | *zntR* | HTH-type transcriptional regulator ZntR OS = *Escherichia coli* OX = 562 GN = zntR PE = 4 SV = 1 | 0.52882261 | 0.002537964 | Down |
|  | A0A828R1M2 | EC3431_4715 | Formate hydrogenlyase subunit 7 OS = *Escherichia coli* 3431 OX = 670892 GN = EC3431_4715 PE = 4 SV = 1 | 0.526539247 | 0.02867257 | Down |
|  | A0A829IMX9 | G686_02181 | D-tagatose-1,6-bisphosphate aldolase subunit gatZ OS = *Escherichia coli* HVH 6 (3-8296502) OX = 1280961 GN = G686_02181 PE = 4 SV = 1 | 0.525472344 | 0.002323498 | Down |
|  | A0A5P0JHD6 | *inaA* | Lipopolysaccharide kinase InaA (Fragment) OS = *Escherichia coli* OX = 562 GN = inaA PE = 4 SV = 1 | 0.521045478 | 0.001105995 | Down |
|  | A0A8A5HPJ3 | JSU13_08840 | Uncharacterized protein OS = *Escherichia coli* O89m : H9 OX = 2810406 GN = JSU13_08840 PE = 4 SV = 1 | 0.514598398 | 0.014070586 | Down |
|  | A0A853WCM3 | BJI68_19085 | Uncharacterized protein OS = *Escherichia coli* OX = 562 GN = BJI68_19085 PE = 4 SV = 1 | 0.513912255 | 0.000185139 | Down |
|  | A0A826YKV9 | FA849_18875 | Formate dehydrogenase subunit alpha OS = *Escherichia coli* OX = 562 GN = FA849_18875 PE = 4 SV = 1 | 0.512549003 | 0.005153268 | Down |
|  | P0AD05 | *yecA* | Uncharacterized protein YecA OS = *Escherichia coli* (strain K12) OX = 83333 GN = yecA PE = 4 SV = 1 | 0.509515453 | 0.000182647 | Down |
|  | A0A7L5VD13 | *galP* | Galactose/proton symporter OS = *Escherichia coli* OX = 562 GN = galP PE = 3 SV = 1 | 0.501073276 | 0.014546911 | Down |
|  | A0A1M2HSR1 | *ygaP* | Inner membrane protein YgaP OS = *Escherichia coli* OX = 562 GN = ygaP PE = 4 SV = 1 | 0.500154905 | 2.33526E-05 | Down |
|  | A0A376I3P5 | *ushA* | Protein UshA [includes : UDP-sugar hydrolase 5'-nucleotidase] OS = *Escherichia coli* OX = 562 GN = ushA PE = 3 SV = 1 | 0.498166871 | 0.003386964 | Down |
|  | A0A2X3M5Q2 | *yafK* | Membrane protein OS = *Escherichia coli* OX = 562 GN = yafK PE = 3 SV = 1 | 0.496201297 | 0.0299299 | Down |
|  | A0A775TXC2 | GRC90_26205 | Type 1 fimbrial protein (Fragment) OS = *Escherichia coli* OX = 562 GN = GRC90_26205 PE = 4 SV = 1 | 0.495989839 | 0.004040876 | Down |
|  | A0A6D0H6A5 | *gatZ* | D-tagatose-1,6-bisphosphate aldolase subunit GatZ OS = *Escherichia coli* OX = 562 GN = gatZ PE = 3 SV = 1 | 0.4886395 | 0.008500662 | Down |
|  | A0A1X3I1D3 | EAMG_01047 | Glycine betaine-binding periplasmic protein OS = *Escherichia coli* M056 OX = 656415 GN = EAMG_01047 PE = 4 SV = 1 | 0.481725938 | 0.021138996 | Down |
|  | A0A6L4XP60 | *glnH* | Glutamine ABC transporter substrate-binding protein GlnH OS = *Escherichia coli* OX = 562 GN = glnH PE = 3 SV = 1 | 0.478400347 | 0.000233371 | Down |
|  | A0A3L2Q016 | D9J03_02630 | Tetratricopeptide repeat-containing protein OS = *Escherichia coli* OX = 562 GN = D9J03_02630 PE = 3 SV = 1 | 0.469426255 | 0.001996726 | Down |
|  | B6I140 | *ybeD* | UPF0250 protein YbeD OS = *Escherichia coli* (strain SE11) OX = 409438 GN = ybeD PE = 3 SV = 1 | 0.466684335 | 0.000712863 | Down |
|  | A0A6D0H698 | *rplX* | 50S ribosomal protein L24 OS = *Escherichia coli* OX = 562 GN = rplX PE = 3 SV = 1 | 0.45785461 | 0.004264916 | Down |
|  | A0A479JTS0 | *gatA* | Galactitol-specific enzyme IIA component of PTS OS = *Escherichia coli* OX = 562 GN = gatA PE = 4 SV = 1 | 0.45415326 | 1.19681E-05 | Down |
|  | A0A641J6U1 | *gatZ* | D-tagatose-1,6-bisphosphate aldolase subunit GatZ OS = *Escherichia coli* OX = 562 GN = gatZ PE = 3 SV = 1 | 0.448978914 | 3.443E-05 | Down |
|  | A0A2X5F0L7 | *rpsV* | 30S ribosomal protein S22 OS = *Escherichia coli* OX = 562 GN = rpsV PE = 3 SV = 1 | 0.445756075 | 0.007581629 | Down |
|  | A0A7B4LXL2 | HMV41_29505 | NAD(P)H-dependent oxidoreductase OS = *Escherichia coli* OX = 562 GN = HMV41_29505 PE = 4 SV = 1 | 0.434445256 | 0.000383363 | Down |
|  | A0A1Y2XL69 | AW059_18480 | Uncharacterized protein OS = *Escherichia coli* OX = 562 GN = AW059_18480 PE = 4 SV = 1 | 0.433900018 | 0.001468729 | Down |
|  | A0A0A0FJG0 | EL76_1121 | Formate hydrogenlyase subunit 6 OS = *Escherichia coli* G3/10 OX = 1455601 GN = EL76_1121 PE = 4 SV = 1 | 0.407333686 | 0.019261796 | Down |
|  | A0A8B3MB75 | CIG67_04390 | Uncharacterized protein OS = *Escherichia coli* OX = 562 GN = CIG67_04390 PE = 4 SV = 1 | 0.407060861 | 0.001743766 | Down |
|  | A0A5B9AHZ7 | *gatZ* | D-tagatose-1,6-bisphosphate aldolase subunit GatZ OS = *Escherichia coli* OX = 562 GN = gatZ PE = 3 SV = 1 | 0.394631805 | 0.000340403 | Down |
|  | A0A2X1K1Q5 | *gatZ* | D-tagatose-1,6-bisphosphate aldolase subunit GatZ OS = *Escherichia coli* OX = 562 GN = gatZ PE = 3 SV = 1 | 0.387219894 | 0.002800079 | Down |
|  | A0A855FRH0 | CT143_09980 | Formate dehydrogenase OS = *Escherichia coli* OX = 562 GN = CT143_09980 PE = 4 SV = 1 | 0.374088436 | 0.005458871 | Down |
|  | A0A5F1T6E1 | DAH34_09615 | Uncharacterized protein OS = *Escherichia coli* OX = 562 GN = DAH34_09615 PE = 4 SV = 1 | 0.360744249 | 0.001827141 | Down |
|  | P0AB34 | *bssS* | Biofilm regulator BssS OS = *Escherichia coli* O6 : H1 (strain CFT073 / ATCC 700928 / UPEC) OX = 199310 GN = bssS PE = 3 SV = 1 | 0.345194214 | 1.11273E-05 | Down |
|  | A0A7U9B1F9 | ECMG_03141 | Formate hydrogenlyase subunit 3 (FHL subunit 3) (Hydrogenase-3component C) (Fragment) OS = *Escherichia coli* TA143 OX = 656437 GN = ECMG_03141 PE = 4 SV = 1 | 0.31965054 | 0.026275388 | Down |
|  | A0A376M3X3 | *hycA* | Formate hydrogenlyase regulatory protein OS = *Escherichia coli* OX = 562 GN = hycA PE = 4 SV = 1 | 0.308844273 | 0.019521835 | Down |
|  | A0A2X3JZ76 | *smg* | Protein Smg OS = *Escherichia coli* OX = 562 GN = smg PE = 3 SV = 1 | 0.304665719 | 0.00165412 | Down |
|  | A0A376HX34 | *yhgF* | Putative transcription accessory protein OS = *Escherichia coli* OX = 562 GN = yhgF PE = 4 SV = 1 | 0.284489482 | 0.001231761 | Down |
|  | A0A837Y776 | AML23_24525 | Cold-shock protein OS = *Escherichia coli* OX = 562 GN = AML23_24525 PE = 4 SV = 1 | 0.229924148 | 0.000841937 | Down |
|  | A0A6L4XKW1 | GP710_07770 | Cold-shock protein OS = *Escherichia coli* OX = 562 GN = GP710_07770 PE = 4 SV = 1 | 0.204366804 | 0.004291097 | Down |
|  | A0A1U9SKI6 | A8C65_23405 | Cold shock protein YdfK OS = *Escherichia coli* OX = 562 GN = A8C65_23405 PE = 4 SV = 1 | 0.200691029 | 0.0018276 | Down |
|  | P76073 | *ynaE* | Uncharacterized protein YnaE OS = *Escherichia coli* (strain K12) OX = 83333 GN = ynaE PE = 2 SV = 2 | 0.154276723 | 0.002204808 | Down |
|  | P0A9Y0 | *cspA* | Cold shock protein CspA OS = *Escherichia coli* O6 : H1 (strain CFT073 / ATCC 700928 / UPEC) OX = 199310 GN = cspA PE = 3 SV = 2 | 0.144369723 | 0.000140018 | Down |
|  | A0A8B4P6I6 | *infB_2* | Translation initiation factor IF2-alpha OS = *Escherichia coli* OX = 562 GN = infB_2 PE = 4 SV = 1 | 0.142009075 | 0.028835878 | Down |
|  | A0A6M0PSK1 | *cspA* | RNA chaperone/antiterminator CspA OS = *Escherichia coli* OX = 562 GN = cspA PE = 4 SV = 1 | 0.112148965 | 0.000120858 | Down |
| S2-3 | A0A1M2ERV6 | BK375_16280 | Autotransporter domain-containing protein OS = *Escherichia coli* OX = 562 GN = BK375_16280 PE = 4 SV = 1 | 8.87946173 | 0.000274495 | Up |
|  | A0A8B4PN44 | *tnaA* | Tryptophanase OS = *Escherichia coli* OX = 562 GN = tnaA PE = 4 SV = 1 | 5.862578744 | 0.001317915 | Up |
|  | A0A0K9TJ89 | ERYG_00146 | Autotransporter beta-domain-containing protein OS = *Escherichia coli* M114 OX = 656416 GN = ERYG_00146 PE = 4 SV = 1 | 5.66789713 | 0.000184817 | Up |
|  | A0A8B5INY3 | *ag43* | Autotransporter adhesin Ag43 OS = *Escherichia coli* OX = 562 GN = ag43 PE = 4 SV = 1 | 4.775045913 | 9.20974E-07 | Up |
|  | A0A771BBG3 | *tnaA* | Tryptophanase OS = *Escherichia coli* OX = 562 GN = tnaA PE = 4 SV = 1 | 4.250832008 | 0.00304499 | Up |
|  | A0A8A5J2H5 | JSU10_11650 | LysR family transcriptional regulator OS = *Escherichia coli* H20 OX = 2810409 GN = JSU10_11650 PE = 4 SV = 1 | 3.992427187 | 0.013827608 | Up |
|  | A0A2X7H313 | *tnaA* | Tryptophanase OS = *Escherichia coli* OX = 562 GN = tnaA PE = 3 SV = 1 | 3.592706383 | 0.003823833 | Up |
|  | A0A376L5P7 | *ompW_1* | Outer membrane protein W OS = *Escherichia coli* OX = 562 GN = ompW_1 PE = 4 SV = 1 | 3.162393918 | 9.08147E-05 | Up |
|  | W9ADK8 | *ompF* | Outer membrane protein F OS = *Escherichia coli* O25b : H4-ST131 OX = 941322 GN = ompF PE = 3 SV = 1 | 3.001953477 | 0.039965724 | Up |
|  | A0A829JIU5 | G938_01661 | Sulfatase YdeN OS = *Escherichia coli* UMEA 3200-1 OX = 1281213 GN = G938_01661 PE = 4 SV = 1 | 2.82239731 | 0.000744956 | Up |
|  | A0A418U310 | *tnaA* | Tryptophanase OS = *Escherichia coli* OX = 562 GN = tnaA PE = 3 SV = 1 | 2.792988804 | 0.000641906 | Up |
|  | U9XXA4 | *xseB* | Exodeoxyribonuclease 7 small subunit OS = *Escherichia coli* 113290 OX = 1268976 GN = xseB PE = 3 SV = 1 | 2.77150855 | 0.001436436 | Up |
|  | A0A7A3AYV6 | *hemX* | Uroporphyrinogen-III C-methyltransferase OS = *Escherichia coli* OX = 562 GN = hemX PE = 4 SV = 1 | 2.750401805 | 0.000118294 | Up |
|  | A0A6L9DL34 | FZC17_12545 | Pirin family protein OS = *Escherichia coli* OX = 562 GN = FZC17_12545 PE = 3 SV = 1 | 2.616474188 | 0.000268687 | Up |
|  | A0A7L5V678 | *glpT* | Glycerol-3-phosphate transporter OS = *Escherichia coli* OX = 562 GN = glpT PE = 3 SV = 1 | 2.573091652 | 0.00103129 | Up |
|  | A0A826R875 | *envC* | Murein hydrolase activator EnvC OS = *Escherichia coli* OX = 562 GN = envC PE = 4 SV = 1 | 2.506490426 | 0.002555686 | Up |
|  | A0A7U9FZE8 | *tnaA* | Tryptophanase OS = *Escherichia coli* TA143 OX = 656437 GN = tnaA PE = 3 SV = 1 | 2.481503187 | 0.000952458 | Up |
|  | A0A7U9ASB9 | *tnaA* | Tryptophanase OS = *Escherichia coli* TA206 OX = 656440 GN = tnaA PE = 3 SV = 1 | 2.388051023 | 0.01254386 | Up |
|  | A0A376JKT8 | *wrbA_3* | Putative flavoprotein OS = *Escherichia coli* OX = 562 GN = wrbA_3 PE = 4 SV = 1 | 2.379810891 | 0.001548157 | Up |
|  | A0A6M0PU76 | *rplI* | 50S ribosomal protein L9 OS = *Escherichia coli* OX = 562 GN = rplI PE = 3 SV = 1 | 2.379766746 | 0.002718889 | Up |
|  | A0A7T8PQW7 | *waaO* | Lipopolysaccharide 3-alpha-galactosyltransferase OS = *Escherichia coli* OX = 562 GN = waaO PE = 4 SV = 1 | 2.329532264 | 0.003257656 | Up |
|  | S1IP87 | A1WU_02908 | Uncharacterized protein OS = *Escherichia coli* KTE108 OX = 1182704 GN = A1WU_02908 PE = 4 SV = 1 | 2.301479468 | 0.000649057 | Up |
|  | A0A853RYC6 | BHF52_02880 | Tryptophanase OS = *Escherichia coli* OX = 562 GN = BHF52_02880 PE = 4 SV = 1 | 2.298080801 | 0.001215682 | Up |
|  | A0A6C9IM12 | GKF86_27255 | SAM-dependent methyltransferase OS = *Escherichia coli* OX = 562 GN = GKF86_27255 PE = 4 SV = 1 | 2.292887956 | 0.001104896 | Up |
|  | A0A3Y1V1S8 | *galF* | Alpha-D-glucosyl-1-phosphate uridylyltransferase OS = *Escherichia coli* OX = 562 GN = galF PE = 3 SV = 1 | 2.269687556 | 0.000522059 | Up |
|  | Q46774 | *glpQ* | Glycerophosphoryl diester phosphodiesterase (Fragment) OS = *Escherichia coli* OX = 562 GN = glpQ PE = 4 SV = 1 | 2.238167144 | 0.002420701 | Up |
|  | A0A1E5M202 | *ydfZ* | Selenium carrying protein OS = *Escherichia coli* OX = 562 GN = ydfZ PE = 4 SV = 1 | 2.225834267 | 0.009291092 | Up |
|  | A0A377BSF6 | *ompW_1* | Outer membrane protein W OS = *Escherichia coli* OX = 562 GN = ompW_1 PE = 4 SV = 1 | 2.214445155 | 0.000661732 | Up |
|  | A0A2S8JZY6 | *asnA* | Aspartate--ammonia ligase OS = *Escherichia coli* OX = 562 GN = asnA PE = 3 SV = 1 | 2.16746481 | 0.001937954 | Up |
|  | A0A2I6IG80 | *ompW* | Outer membrane protein OmpW OS = *Escherichia coli* OX = 562 GN = ompW PE = 4 SV = 1 | 2.161932395 | 0.000992279 | Up |
|  | D7Y8S5 | *fucO* | Lactaldehyde reductase OS = *Escherichia coli* (strain MS 115-1) OX = 749537 GN = fucO PE = 4 SV = 1 | 2.108700673 | 0.001017407 | Up |
|  | A0A6C8U056 | GP906_04855 | Sulfatase-like hydrolase/transferase OS = *Escherichia coli* OX = 562 GN = GP906_04855 PE = 3 SV = 1 | 2.108124479 | 0.026499368 | Up |
|  | A0A2X1N950 | *mliC* | Putative lipoprotein OS = *Escherichia coli* OX = 562 GN = mliC PE = 4 SV = 1 | 2.08253863 | 0.038844995 | Up |
|  | A0A777SAD9 | *tnaA* | Tryptophanase OS = *Escherichia coli* OX = 562 GN = tnaA PE = 3 SV = 1 | 2.081587392 | 7.1953E-05 | Up |
|  | U9ZBZ0 | HMPREF1599_04467 | Universal stress family protein OS = *Escherichia coli* 907713 OX = 1268986 GN = HMPREF1599_04467 PE = 3 SV = 1 | 2.072107275 | 0.008264928 | Up |
|  | A0A3L0W5P1 | *rne* | Ribonuclease E OS = *Escherichia coli* OX = 562 GN = rne PE = 3 SV = 1 | 2.070098379 | 0.048827981 | Up |
|  | A0A3R0VNX2 | *cbpA* | Curved DNA-binding protein OS = *Escherichia coli* OX = 562 GN = cbpA PE = 3 SV = 1 | 2.063411103 | 0.027254748 | Up |
|  | A0A7B3MKM9 | HLZ50_09340 | DUF892 family protein OS = *Escherichia coli* OX = 562 GN = HLZ50_09340 PE = 4 SV = 1 | 2.061753517 | 0.021516462 | Up |
|  | A0A5P0J560 | *uspF* | Universal stress protein UspF OS = *Escherichia coli* OX = 562 GN = uspF PE = 3 SV = 1 | 2.059525212 | 0.000795849 | Up |
|  | A0A1X3LUN7 | EAZG_00482 | Citrate lyase alpha chain OS = *Escherichia coli* TA249 OX = 656441 GN = EAZG_00482 PE = 4 SV = 1 | 2.05927301 | 0.00021994 | Up |
|  | A0A376J8Y2 | *tnaB* | Aromatic amino acid permease OS = *Escherichia coli* OX = 562 GN = tnaB PE = 3 SV = 1 | 2.02051843 | 0.004096962 | Up |
|  | A0A5B9B029 | FTV90_14225 | SecY/secA suppressor protein OS = *Escherichia coli* OX = 562 GN = FTV90_14225 PE = 4 SV = 1 | 2.015786157 | 0.027888771 | Up |
|  | A0A376S463 | *tnaA* | Tryptophanase OS = *Escherichia coli* OX = 562 GN = tnaA PE = 4 SV = 1 | 1.987421624 | 0.002118863 | Up |
|  | A0A828JSI0 | EH88_005446 | Acetyltransferase OS = *Escherichia coli* OX = 562 GN = EH88_005446 PE = 4 SV = 1 | 1.918854367 | 0.00077755 | Up |
|  | A0A3U5UQ40 | *uspD* | Universal stress protein OS = *Escherichia coli* OX = 562 GN = uspD PE = 3 SV = 1 | 1.890533027 | 0.015574732 | Up |
|  | A0A6N8K9S4 | *ribD* | 5-amino-6-(5-phosphoribosylamino)uracil reductase (Fragment) OS = *Escherichia coli* OX = 562 GN = ribD PE = 4 SV = 1 | 1.883941935 | 0.019963602 | Up |
|  | A0A6N9MUA6 | *glpQ* | Glycerophosphodiester phosphodiesterase OS = *Escherichia coli* OX = 562 GN = glpQ PE = 4 SV = 1 | 1.879513287 | 0.002371945 | Up |
|  | A0A6M0PVJ7 | *pyrG* | CTP synthase OS = *Escherichia coli* OX = 562 GN = pyrG PE = 3 SV = 1 | 1.869156055 | 0.000679249 | Up |
|  | A0A7U2BDF2 | *ompW* | Outer membrane protein OmpW OS = *Escherichia coli* OX = 562 GN = ompW PE = 4 SV = 1 | 1.858104626 | 0.012191803 | Up |
|  | A0A377AU17 | *cbpA_2* | Chaperone modulatory protein CbpM OS = *Escherichia coli* OX = 562 GN = cbpA_2 PE = 3 SV = 1 | 1.837937333 | 0.001265749 | Up |
|  | A0A271QYN7 | BIZ41_14720 | Glutamate synthase large subunit OS = *Escherichia coli* OX = 562 GN = BIZ41_14720 PE = 3 SV = 1 | 1.837102408 | 0.00160108 | Up |
|  | A0A6N8PGB0 | FQ021_16075 | GntR family transcriptional regulator OS = *Escherichia coli* OX = 562 GN = FQ021_16075 PE = 4 SV = 1 | 1.818140881 | 0.005539353 | Up |
|  | A0A828MYB1 | *ibpA* | Heat shock chaperone IbpA OS = *Escherichia coli* OX = 562 GN = ibpA PE = 4 SV = 1 | 1.808219699 | 0.003347313 | Up |
|  | A0A5B1FAQ8 | *glpT* | GlpT protein OS = *Escherichia coli* OX = 562 GN = glpT PE = 3 SV = 1 | 1.802890867 | 0.004493594 | Up |
|  | A0A418GR70 | *fadB* | Fatty acid oxidation complex subunit alpha FadB (Fragment) OS = *Escherichia coli* OX = 562 GN = fadB PE = 4 SV = 1 | 1.788554338 | 0.010229422 | Up |
|  | A0A0K4EBA5 | *ccpA* | Transcriptional regulator YcjW OS = *Escherichia coli* OX = 562 GN = ccpA PE = 4 SV = 1 | 1.788459211 | 0.008615857 | Up |
|  | A0A6M0PS89 | G4V03_04295 | Succinate dehydrogenase iron-sulfur subunit OS = *Escherichia coli* OX = 562 GN = G4V03_04295 PE = 3 SV = 1 | 1.782366125 | 0.026865973 | Up |
|  | A0A660HE83 | A9X72_14540 | Uncharacterized protein OS = *Escherichia coli* OX = 562 GN = A9X72_14540 PE = 4 SV = 1 | 1.770660441 | 3.48505E-05 | Up |
|  | A0A6N8PVJ3 | *ackA* | Acetate kinase OS = *Escherichia coli* OX = 562 GN = ackA PE = 3 SV = 1 | 1.766959881 | 0.007617257 | Up |
|  | A0A826JV02 | *fadD* | Long-chain-fatty-acid--CoA ligase FadD OS = *Escherichia coli* OX = 562 GN = fadD PE = 4 SV = 1 | 1.760508121 | 0.00396026 | Up |
|  | W9AMC1 | *tnaA* | Tryptophanase OS = *Escherichia coli* O25b : H4-ST131 OX = 941322 GN = tnaA PE = 3 SV = 1 | 1.757276507 | 0.018781513 | Up |
|  | A0A828FSI3 | *cybC* | Cytochrome b562 OS = *Escherichia coli* OX = 562 GN = cybC PE = 4 SV = 1 | 1.749825778 | 0.02005756 | Up |
|  | A0A826LKU7 | C5542_001496 | HlyD family secretion protein OS = *Escherichia coli* OX = 562 GN = C5542_001496 PE = 4 SV = 1 | 1.745816903 | 0.001196549 | Up |
|  | A0A377CFY3 | *hchA* | Protein/nucleic acid deglycase HchA OS = *Escherichia coli* OX = 562 GN = hchA PE = 2 SV = 1 | 1.745586921 | 0.019887194 | Up |
|  | A0A6N8PP51 | GRW77_00150 | 2-amino-thiazoline-4-carboxylic acid hydrolase OS = *Escherichia coli* OX = 562 GN = GRW77_00150 PE = 4 SV = 1 | 1.745030807 | 0.030738645 | Up |
|  | A0A855W463 | DB282_27280 | Protease (Fragment) OS = *Escherichia coli* OX = 562 GN = DB282_27280 PE = 4 SV = 1 | 1.726103682 | 0.017427364 | Up |
|  | A0A6C9LHP4 | *yedE* | Selenium metabolism membrane protein YedE/FdhT OS = *Escherichia coli* OX = 562 GN = yedE PE = 3 SV = 1 | 1.720279064 | 0.007351691 | Up |
|  | A0A8A8PXU7 | *purA* | Adenylosuccinate synthase OS = *Escherichia coli* OX = 562 GN = purA PE = 4 SV = 1 | 1.718095035 | 0.024981378 | Up |
|  | A0A827TFN0 | F9407_14020 | AAA family ATPase OS = *Escherichia coli* OX = 562 GN = F9407_14020 PE = 4 SV = 1 | 1.71514132 | 0.002195241 | Up |
|  | A0A2A3VUL7 | *tnaA* | Tryptophanase OS = *Escherichia coli* OX = 562 GN = tnaA PE = 3 SV = 1 | 1.705962126 | 0.024979918 | Up |
|  | A0A7A3ATP6 | *cbpM* | Chaperone modulatory protein CbpM OS = *Escherichia coli* OX = 562 GN = cbpM PE = 3 SV = 1 | 1.705335546 | 0.013439336 | Up |
|  | A0A5F1DS00 | *sdhB* | Succinate dehydrogenase iron-sulfur subunit OS = *Escherichia coli* OX = 562 GN = sdhB PE = 3 SV = 1 | 1.702242788 | 0.04876199 | Up |
|  | A0A6L4XKT9 | GP710_08630 | Putative NAD(P)H nitroreductase OS = *Escherichia coli* OX = 562 GN = GP710_08630 PE = 3 SV = 1 | 1.685435584 | 0.001108817 | Up |
|  | A0A5D8QXC9 | *ompT* | Omptin family outer membrane protease OmpT (Fragment) OS = *Escherichia coli* OX = 562 GN = ompT PE = 4 SV = 1 | 1.68442997 | 0.00231207 | Up |
|  | Q8XAW4 | Z2210 | Putative sulfatase OS = *Escherichia coli* O157 : H7 OX = 83334 GN = Z2210 PE = 3 SV = 2 | 1.679190966 | 0.000690736 | Up |
|  | Q5IIG4 | *aspC* | Aspartate amino transferase (Fragment) OS = *Escherichia coli* OX = 562 GN = aspC PE = 4 SV = 1 | 1.675076073 | 0.034640769 | Up |
|  | A0A6G4BZV9 | *tnaA* | Tryptophanase OS = *Escherichia coli* OX = 562 GN = tnaA PE = 3 SV = 1 | 1.674422257 | 0.000771871 | Up |
|  | A0A7U9QDI6 | *torS* | Histidine kinase OS = *Escherichia coli* O145 : H28 OX = 1078034 GN = torS PE = 4 SV = 1 | 1.671797989 | 6.69914E-05 | Up |
|  | A0A5C9AIS4 | FWK02_15270 | DUF406 domain-containing protein (Fragment) OS = *Escherichia coli* OX = 562 GN = FWK02_15270 PE = 3 SV = 1 | 1.662480044 | 0.001875971 | Up |
|  | A0A777RRA0 | GFY48_05910 | Aldo/keto reductase OS = *Escherichia coli* OX = 562 GN = GFY48_05910 PE = 4 SV = 1 | 1.659826324 | 0.028072385 | Up |
|  | A0A828L8H3 | *cydB* | Cytochrome d ubiquinol oxidase subunit II OS = *Escherichia coli* OX = 562 GN = cydB PE = 4 SV = 1 | 1.65383549 | 0.000385164 | Up |
|  | A0A6N8QK59 | GRW57_23620 | Phosphoglycolate phosphatase (Fragment) OS = *Escherichia coli* OX = 562 GN = GRW57_23620 PE = 4 SV = 1 | 1.651049347 | 0.031744024 | Up |
|  | P0A952 | *speG* | Spermidine N(1)-acetyltransferase OS = *Escherichia coli* O157 : H7 OX = 83334 GN = speG PE = 3 SV = 2 | 1.649773802 | 0.006955612 | Up |
|  | A0A376I8B9 | *cydA* | Cytochrome d ubiquinol oxidase subunit 1 OS = *Escherichia coli* OX = 562 GN = cydA PE = 3 SV = 1 | 1.645567722 | 0.006561841 | Up |
|  | W1ETT4 | unknow | Shikimate kinase I OS = *Escherichia coli* ISC7 OX = 1432555 PE = 4 SV = 1 | 1.643294003 | 0.005994991 | Up |
|  | D2SX86 | *fimC* | Type 1 fimbrial chaperone protein (Fragment) OS = *Escherichia coli* OX = 562 GN = fimC PE = 3 SV = 1 | 1.638631107 | 0.005569257 | Up |
|  | A0A403LMR9 | *leuS* | Leucine--tRNA ligase OS = *Escherichia coli* OX = 562 GN = leuS PE = 3 SV = 1 | 1.637032399 | 0.00711111 | Up |
|  | A0A4Y5R2G0 | *blaPER* | Beta-lactamase (Fragment) OS = *Escherichia coli* OX = 562 GN = blaPER PE = 3 SV = 1 | 1.635122555 | 0.009170775 | Up |
|  | Q8XCG1 | *flhB* | Flagellar biosynthetic protein FlhB OS = *Escherichia coli* O157 : H7 OX = 83334 GN = flhB PE = 3 SV = 2 | 1.634351017 | 0.025335189 | Up |
|  | A0A7I8Z834 | *glpQ* | GlpQ protein OS = *Escherichia coli* OX = 562 GN = glpQ PE = 4 SV = 1 | 1.633704968 | 0.001480303 | Up |
|  | A0A5B9AIC4 | FTV93_04135 | DUF1315 family protein OS = *Escherichia coli* OX = 562 GN = FTV93_04135 PE = 4 SV = 1 | 1.633461158 | 0.022849042 | Up |
|  | A0A7H9LNK0 | *cadA* | Lysine decarboxylase CadA OS = *Escherichia coli* OX = 562 GN = cadA PE = 3 SV = 1 | 1.63304246 | 0.008354495 | Up |
|  | A0A2X3A7G0 | *maeB* | NADP-dependent malic enzyme OS = *Escherichia coli* OX = 562 GN = maeB PE = 3 SV = 1 | 1.632914192 | 0.026634008 | Up |
|  | A0A843M827 | D9F92_10695 | Uncharacterized protein OS = *Escherichia coli* OX = 562 GN = D9F92_10695 PE = 4 SV = 1 | 1.630927297 | 0.002491918 | Up |
|  | A8A6H0 | *tnaA* | Tryptophanase OS = *Escherichia coli* O9 : H4 (strain HS) OX = 331112 GN = tnaA PE = 3 SV = 1 | 1.629284652 | 0.006458652 | Up |
|  | A0A6M0PS67 | *glnS* | Glutamine--tRNA ligase OS = *Escherichia coli* OX = 562 GN = glnS PE = 3 SV = 1 | 1.627397246 | 0.023565141 | Up |
|  | P0AED2 | *uspA* | Universal stress protein A OS = *Escherichia coli* O157 : H7 OX = 83334 GN = uspA PE = 3 SV = 2 | 1.62015069 | 0.008607017 | Up |
|  | L4J2M7 | *rodZ* | Cytoskeleton protein RodZ OS = *Escherichia coli* KTE146 OX = 1182725 GN = rodZ PE = 3 SV = 1 | 1.618632913 | 1.27811E-05 | Up |
|  | A0A2A2BPI3 | BFL24_10370 | Sulfatase OS = *Escherichia coli* OX = 562 GN = BFL24_10370 PE = 3 SV = 1 | 1.61641377 | 0.001248029 | Up |
|  | A0A376HT67 | *gor* | Glutathione reductase OS = *Escherichia coli* OX = 562 GN = gor PE = 3 SV = 1 | 1.616371202 | 0.008043184 | Up |
|  | A0A771MQY9 | *kbl* | 2-amino-3-ketobutyrate coenzyme A ligase OS = *Escherichia coli* OX = 562 GN = kbl PE = 3 SV = 1 | 1.613130789 | 0.046735522 | Up |
|  | A0A854ADD1 | BMT50_17580 | Autonomous glycyl radical cofactor GrcA OS = *Escherichia coli* OX = 562 GN = BMT50_17580 PE = 4 SV = 1 | 1.608577726 | 0.045576888 | Up |
|  | A0A827NRZ2 | *hdhA* | 7-alpha-hydroxysteroid dehydrogenase OS = *Escherichia coli* OX = 562 GN = hdhA PE = 4 SV = 1 | 1.605099736 | 0.010460306 | Up |
|  | A0A6M0PUJ1 | G4V03_08605 | Fumarate hydratase class I OS = *Escherichia coli* OX = 562 GN = G4V03_08605 PE = 3 SV = 1 | 1.604302304 | 0.004308078 | Up |
|  | E6BDM7 | *dps* | DNA protection during starvation protein OS = *Escherichia coli* MS 85-1 OX = 679202 GN = dps PE = 3 SV = 1 | 1.602901712 | 0.008471983 | Up |
|  | A0A376NYE6 | *nrfA* | Cytochrome c-552 OS = *Escherichia coli* OX = 562 GN = nrfA PE = 3 SV = 1 | 1.595323647 | 0.016851054 | Up |
|  | A0A826RS52 | FPS82_03185 | DUF2756 family protein OS = *Escherichia coli* OX = 562 GN = FPS82_03185 PE = 4 SV = 1 | 1.595104236 | 0.015577897 | Up |
|  | A0A2T3THJ9 | C7B02_06005 | Monooxygenase OS = *Escherichia coli* OX = 562 GN = C7B02_06005 PE = 4 SV = 1 | 1.593867195 | 0.002277006 | Up |
|  | E2QDH5 | unknow | Uncharacterized protein OS = *Escherichia coli* LF82 OX = 591946 PE = 4 SV = 1 | 1.590857845 | 0.003938469 | Up |
|  | A0A2G9ABS0 | *sufD* | Fe-S cluster assembly protein SufD OS = *Escherichia coli* OX = 562 GN = sufD PE = 3 SV = 1 | 1.590196905 | 0.037295552 | Up |
|  | F4SG82 | ECHG_02138 | Glycerophosphoryl diester phosphodiesterase GlpQ OS = *Escherichia coli* H736 OX = 656414 GN = ECHG_02138 PE = 4 SV = 1 | 1.587826223 | 0.007770778 | Up |
|  | A0A826MHE4 | *ldtB* | L,D-transpeptidase OS = *Escherichia coli* OX = 562 GN = ldtB PE = 4 SV = 1 | 1.585337358 | 0.0193185 | Up |
|  | A0A2S5UBH6 | C4M78_14045 | Uncharacterized protein OS = *Escherichia coli* OX = 562 GN = C4M78_14045 PE = 4 SV = 1 | 1.58454607 | 0.029074731 | Up |
|  | A0A6N6YDL1 | *glpQ* | Glycerophosphodiester phosphodiesterase OS = *Escherichia coli* OX = 562 GN = glpQ PE = 4 SV = 1 | 1.581552212 | 0.001312548 | Up |
|  | A0A6D0EJA7 | GQM09_23615 | Glutamine--fructose-6-phosphate aminotransferase [isomerizing] (Fragment) OS = *Escherichia coli* OX = 562 GN = GQM09_23615 PE = 4 SV = 1 | 1.58027545 | 0.000430008 | Up |
|  | A0A2P6IUD5 | *rfaF* | ADP-heptose--LPS heptosyltransferase RfaF OS = *Escherichia coli* OX = 562 GN = rfaF PE = 4 SV = 1 | 1.579933863 | 0.006876489 | Up |
|  | A0A827N447 | *pgaA* | Poly-beta-1,6 N-acetyl-D-glucosamine export porin PgaA OS = *Escherichia coli* OX = 562 GN = pgaA PE = 4 SV = 1 | 1.577089465 | 0.000390318 | Up |
|  | A0A6L4XMK4 | GP710_03455 | Fumarate hydratase class I OS = *Escherichia coli* OX = 562 GN = GP710_03455 PE = 3 SV = 1 | 1.575339697 | 0.033991137 | Up |
|  | A0A376S994 | *rmlB_1* | dTDP-D-glucose 4,6-dehydratase rmlB OS = *Escherichia coli* OX = 562 GN = rmlB_1 PE = 4 SV = 1 | 1.574981452 | 6.69608E-05 | Up |
|  | D8AE21 | HMPREF9530_04824 | Uncharacterized protein OS = *Escherichia coli* (strain MS 21-1) OX = 749527 GN = HMPREF9530_04824 PE = 4 SV = 1 | 1.574674998 | 0.000523112 | Up |
|  | A0A3L0VV29 | D9F05_05105 | Glycerophosphodiester phosphodiesterase OS = *Escherichia coli* OX = 562 GN = D9F05_05105 PE = 4 SV = 1 | 1.565582022 | 0.000166134 | Up |
|  | A0A843ZG16 | E4K54_28035 | GTP-binding protein (Fragment) OS = *Escherichia coli* OX = 562 GN = E4K54_28035 PE = 4 SV = 1 | 1.558635091 | 0.028643728 | Up |
|  | A0A7H9LUK6 | *glpC* | Anaerobic glycerol-3-phosphate dehydrogenase subunit C OS = *Escherichia coli* OX = 562 GN = glpC PE = 4 SV = 1 | 1.557492155 | 0.001252618 | Up |
|  | A0A376PP12 | *yrfD* | DNA utilization protein HofM OS = *Escherichia coli* OX = 562 GN = yrfD PE = 4 SV = 1 | 1.555788157 | 0.017776967 | Up |
|  | A0A826ZD52 | *hdhA* | 7-alpha-hydroxysteroid dehydrogenase OS = *Escherichia coli* OX = 562 GN = hdhA PE = 4 SV = 1 | 1.550073104 | 0.019591599 | Up |
|  | A0A826QPY9 | *menE* | O-succinylbenzoate--CoA ligase OS = *Escherichia coli* OX = 562 GN = menE PE = 4 SV = 1 | 1.549776503 | 0.005468077 | Up |
|  | A0A0K3PZ85 | *rmuC* | DNA recombination protein RmuC OS = *Escherichia coli* OX = 562 GN = rmuC PE = 3 SV = 1 | 1.549638557 | 0.00142194 | Up |
|  | A0A0A1A5H6 | *rihC* | Non-specific ribonucleoside hydrolase RihC OS = *Escherichia coli* OX = 562 GN = rihC PE = 3 SV = 1 | 1.545302781 | 0.000366806 | Up |
|  | I2UGI9 | EC40522_5562 | tRNA ligases class II (D, K and N) OS = *Escherichia coli* 4.0522 OX = 869681 GN = EC40522_5562 PE = 4 SV = 1 | 1.536620703 | 0.039204437 | Up |
|  | A0A765X6R8 | GGB84_001845 | L,D-transpeptidase family protein OS = *Escherichia coli* OX = 562 GN = GGB84_001845 PE = 3 SV = 1 | 1.536297288 | 0.010730287 | Up |
|  | B6I4S1 | *zapB* | Cell division protein ZapB OS = *Escherichia coli* (strain SE11) OX = 409438 GN = zapB PE = 3 SV = 1 | 1.535340513 | 0.003710038 | Up |
|  | A0A7L5V1W3 | *mscK* | Mechanosensitive channel MscK OS = *Escherichia coli* OX = 562 GN = mscK PE = 3 SV = 1 | 1.53385862 | 0.027439281 | Up |
|  | A0A7H2C767 | *clpB* | Chaperone protein ClpB OS = *Escherichia coli* (strain K12) OX = 83333 GN = clpB PE = 3 SV = 1 | 1.531933265 | 0.014964168 | Up |
|  | A0A1X3LWJ5 | EAZG_01135 | Uncharacterized protein OS = *Escherichia coli* TA249 OX = 656441 GN = EAZG_01135 PE = 4 SV = 1 | 1.528341423 | 0.037613071 | Up |
|  | D8ADF8 | *moaB* | Molybdenum cofactor biosynthesis protein B OS = *Escherichia coli* (strain MS 21-1) OX = 749527 GN = moaB PE = 3 SV = 1 | 1.527824067 | 0.01597927 | Up |
|  | Q1R585 | *yhjA* | Probable cytochrome C peroxidase OS = *Escherichia coli* (strain UTI89 / UPEC) OX = 364106 GN = yhjA PE = 4 SV = 1 | 1.523913797 | 0.029997476 | Up |
|  | A0A7Z1F0N1 | APX88_03850 | Fumarate hydratase class I OS = *Escherichia coli* OX = 562 GN = APX88_03850 PE = 3 SV = 1 | 1.522797009 | 0.01898364 | Up |
|  | A0A831EYL4 | *yqhD* | Alcohol dehydrogenase OS = *Escherichia coli* OX = 562 GN = yqhD PE = 4 SV = 1 | 1.521466789 | 0.003195866 | Up |
|  | A0A6D0USM6 | *glpQ* | Glycerophosphodiester phosphodiesterase (Fragment) OS = *Escherichia coli* OX = 562 GN = glpQ PE = 4 SV = 1 | 1.51860434 | 0.004475857 | Up |
|  | A0A6D0FG95 | *flgM* | Anti-sigma-28 factor OS = *Escherichia coli* OX = 562 GN = flgM PE = 3 SV = 1 | 1.515346184 | 0.023481467 | Up |
|  | A0A828S740 | ECSTEC7V_3840 | Enhancing lycopene biosynthesis protein 2 OS = *Escherichia coli* STEC_7v OX = 754082 GN = ECSTEC7V_3840 PE = 4 SV = 1 | 1.514953261 | 0.000971784 | Up |
|  | A0A8B5PFJ8 | *tnaA* | Tryptophanase OS = *Escherichia coli* OX = 562 GN = tnaA PE = 4 SV = 1 | 1.514895431 | 0.03161663 | Up |
|  | A0A3W5XXF0 | *cydA* | Cytochrome bd-I ubiquinol oxidase subunit CydA OS = *Escherichia coli* OX = 562 GN = cydA PE = 3 SV = 1 | 1.513667881 | 0.030432564 | Up |
|  | A0A827LJ87 | *yqhD* | Alcohol dehydrogenase OS = *Escherichia coli* OX = 562 GN = yqhD PE = 4 SV = 1 | 1.508178025 | 0.027639921 | Up |
|  | A0A7H9QZ14 | *yahK* | NADPH-dependent aldehyde reductase YahK OS = *Escherichia coli* OX = 562 GN = yahK PE = 3 SV = 1 | 1.507455235 | 0.0049474 | Up |
|  | A0A789MAE1 | *katG* | Catalase-peroxidase OS = *Escherichia coli* OX = 562 GN = katG PE = 3 SV = 1 | 1.500649456 | 0.023673699 | Up |
|  | A0A789R943 | *kdsD* | Arabinose 5-phosphate isomerase OS = *Escherichia coli* OX = 562 GN = kdsD PE = 3 SV = 1 | 1.494135291 | 0.007141749 | Up |
|  | A0A1Q6BB69 | *hemL* | Glutamate-1-semialdehyde 2,1-aminomutase OS = *Escherichia coli* OX = 562 GN = hemL PE = 3 SV = 1 | 1.493750421 | 0.043325611 | Up |
|  | A0A6M0Q0B7 | *purC* | Phosphoribosylaminoimidazole-succinocarboxamide synthase OS = *Escherichia coli* OX = 562 GN = purC PE = 3 SV = 1 | 1.491206924 | 0.02729306 | Up |
|  | A0A777HIK4 | *glpT* | Glycerol-3-phosphate transporter OS = *Escherichia coli* OX = 562 GN = glpT PE = 3 SV = 1 | 1.486417466 | 0.020834893 | Up |
|  | A0A827QN65 | GQW07_19440 | Inhibitor of g-type lysozyme OS = *Escherichia coli* OX = 562 GN = GQW07_19440 PE = 4 SV = 1 | 1.482348506 | 0.003059655 | Up |
|  | A0A827IXR6 | GIB53_25780 | IS1 family transposase OS = *Escherichia coli* OX = 562 GN = GIB53_25780 PE = 4 SV = 1 | 1.479512472 | 0.023589704 | Up |
|  | A0A3W2VQN2 | *ldtE* | L,D-transpeptidase LdtE OS = *Escherichia coli* OX = 562 GN = ldtE PE = 3 SV = 1 | 1.475419077 | 7.8426E-05 | Up |
|  | A0A7U1VED7 | JNN48_04730 | Peptidoglycan DD-metalloendopeptidase family protein OS = *Escherichia coli* OX = 562 GN = JNN48_04730 PE = 4 SV = 1 | 1.475264349 | 0.003437917 | Up |
|  | A0A1X3LME0 | EAXG_05135 | Protease 7 (Protease VII) (Omptin) (Outermembrane protein 3B) (Protease A) OS = *Escherichia coli* TA054 OX = 656433 GN = EAXG_05135 PE = 3 SV = 1 | 1.473880372 | 0.01022983 | Up |
|  | A0A0K3UQX2 | ERS085366_02842 | Fels-1 Propage domain-containing protein OS = *Escherichia coli* OX = 562 GN = ERS085366_02842 PE = 4 SV = 1 | 1.472517447 | 0.005317472 | Up |
|  | A0A6D0M5I7 | *ompX* | Outer membrane protein X (Fragment) OS = *Escherichia coli* OX = 562 GN = ompX PE = 3 SV = 1 | 1.471765098 | 0.028713311 | Up |
|  | A0A377E1X8 | *rcsF* | RcsF--phosphorelay glucose and zinc sensor OS = *Escherichia coli* OX = 562 GN = rcsF PE = 4 SV = 1 | 1.469719422 | 0.00295163 | Up |
|  | E1IW65 | *allR* | HTH-type transcriptional repressor AllR OS = *Escherichia coli* MS 145-7 OX = 679204 GN = allR PE = 4 SV = 1 | 1.468444723 | 0.023633334 | Up |
|  | S1IQ10 | A1WU_00391 | RNase E specificity factor CsrD OS = *Escherichia coli* KTE108 OX = 1182704 GN = A1WU_00391 PE = 4 SV = 1 | 1.467520877 | 0.001056097 | Up |
|  | A0A646J549 | *mug* | G/U mismatch-specific DNA glycosylase OS = *Escherichia coli* OX = 562 GN = mug PE = 3 SV = 1 | 1.467166642 | 0.005564826 | Up |
|  | A0A6C8TFT0 | *eutC* | Ethanolamine ammonia-lyase small subunit OS = *Escherichia coli* OX = 562 GN = eutC PE = 3 SV = 1 | 1.464781524 | 9.80552E-06 | Up |
|  | A0A1X3KRU5 | *nudJ* | Phosphatase NudJ OS = *Escherichia coli* H605 OX = 656410 GN = nudJ PE = 3 SV = 1 | 1.461941918 | 0.015478501 | Up |
|  | A0A2J1D5R7 | *rpoB* | DNA-directed RNA polymerase (Fragment) OS = *Escherichia coli* OX = 562 GN = rpoB PE = 4 SV = 1 | 1.458382339 | 0.040692803 | Up |
|  | A0A2X7FCL6 | SAMEA3753300_01799 | Nucleotide di-P-sugar epimerase or dehydratase OS = *Escherichia coli* OX = 562 GN = SAMEA3753300_01799 PE = 4 SV = 1 | 1.455780165 | 0.002470012 | Up |
|  | A0A828UTS3 | *nrfA* | Formate-dependent cytochrome c nitrite reductase, c552 subunit OS = *Escherichia coli* 3.2608 OX = 869679 GN = nrfA PE = 4 SV = 1 | 1.454110274 | 0.01601323 | Up |
|  | A0A827L415 | BRV34_001673 | NAD(P)/FAD-dependent oxidoreductase OS = *Escherichia coli* OX = 562 GN = BRV34_001673 PE = 4 SV = 1 | 1.45221021 | 0.015802001 | Up |
|  | A0A3Y3V6Q4 | GKF89_16485 | Uncharacterized protein OS = *Escherichia coli* OX = 562 GN = GKF89_16485 PE = 4 SV = 1 | 1.452009895 | 0.002722197 | Up |
|  | A0A376W770 | *eco_2* | Ecotin OS = *Escherichia coli* OX = 562 GN = eco_2 PE = 3 SV = 1 | 1.451168449 | 0.018139069 | Up |
|  | E8Z5G3 | *pgm* | Pgm (Fragment) OS = *Escherichia coli* OX = 562 GN = pgm PE = 4 SV = 1 | 1.4511027 | 0.010567805 | Up |
|  | A0A829GBE0 | WG3_02894 | Arginine ABC transporter permease ArtQ OS = *Escherichia coli* KTE36 OX = 1169353 GN = WG3_02894 PE = 4 SV = 1 | 1.449644581 | 0.003836427 | Up |
|  | A0A826SG38 | *glpD* | Glycerol-3-phosphate dehydrogenase OS = *Escherichia coli* OX = 562 GN = glpD PE = 4 SV = 1 | 1.449186551 | 0.003003828 | Up |
|  | A0A773MCR6 | *tdk* | Thymidine kinase OS = *Escherichia coli* OX = 562 GN = tdk PE = 3 SV = 1 | 1.448425136 | 0.038076857 | Up |
|  | A0A828AVB4 | A8W81_002270 | Oxidative stress defense protein OS = *Escherichia coli* OX = 562 GN = A8W81_002270 PE = 4 SV = 1 | 1.448120529 | 0.01415702 | Up |
|  | A0A6D0GV09 | *glmS* | Glutamine--fructose-6-phosphate aminotransferase [isomerizing] OS = *Escherichia coli* OX = 562 GN = glmS PE = 3 SV = 1 | 1.447121503 | 0.010969583 | Up |
|  | A0A7D7DG69 | *zraP* | Zinc resistance-associated protein OS = *Escherichia coli* OX = 562 GN = zraP PE = 3 SV = 1 | 1.445581562 | 0.032181912 | Up |
|  | A0A5B9AYS4 | FTV90_17080 | Molybdopterin-dependent oxidoreductase OS = *Escherichia coli* OX = 562 GN = FTV90_17080 PE = 4 SV = 1 | 1.439834245 | 0.017119724 | Up |
|  | A0A793U2L0 | *mrr* | Methylated adenine and cytosine restriction protein OS = *Escherichia coli* OX = 562 GN = mrr PE = 4 SV = 1 | 1.437783813 | 0.000320005 | Up |
|  | A0A1V2GDA3 | BXT93_15460 | Uncharacterized protein OS = *Escherichia coli* OX = 562 GN = BXT93_15460 PE = 3 SV = 1 | 1.437227882 | 0.015285039 | Up |
|  | D3GU02 | *dcuR* | Transcriptional regulatory protein OS = *Escherichia coli* O44 : H18 (strain 042 / EAEC) OX = 216592 GN = dcuR PE = 4 SV = 1 | 1.435530907 | 0.016597241 | Up |
|  | A0A0K4G9W2 | *greB* | Transcription elongation factor GreB OS = *Escherichia coli* OX = 562 GN = greB PE = 3 SV = 1 | 1.435463833 | 0.000686829 | Up |
|  | C4ZW75 | *dctA* | C4-dicarboxylate transport protein OS = *Escherichia coli* (strain K12 / MC4100 / BW2952) OX = 595496 GN = dctA PE = 3 SV = 1 | 1.433873156 | 0.027521915 | Up |
|  | A0A6N8Q227 | *yoaH* | UPF0181 protein YoaH OS = *Escherichia coli* OX = 562 GN = yoaH PE = 3 SV = 1 | 1.432673944 | 0.003464989 | Up |
|  | A0A7H9QSS3 | HVX31_03840 | Oxidative stress defense protein OS = *Escherichia coli* OX = 562 GN = HVX31_03840 PE = 4 SV = 1 | 1.4321065 | 0.02677947 | Up |
|  | A0A7I8ZNN1 | *ydcH* | YdcH protein OS = *Escherichia coli* OX = 562 GN = ydcH PE = 4 SV = 1 | 1.430080951 | 0.028905642 | Up |
|  | A0A6D0C6T7 | *cadA* | Lysine decarboxylase CadA OS = *Escherichia coli* OX = 562 GN = cadA PE = 3 SV = 1 | 1.42890849 | 0.025639311 | Up |
|  | A0A829FR97 | WCS_00361 | Phage protein OS = *Escherichia coli* KTE14 OX = 1169333 GN = WCS_00361 PE = 4 SV = 1 | 1.427465692 | 0.007477596 | Up |
|  | A0A829CUZ6 | ECMP0215528_1535 | Outer membrane insertion C-terminal signal domain protein OS = *Escherichia coli* MP021552.8 OX = 1116133 GN = ECMP0215528_1535 PE = 4 SV = 1 | 1.422189214 | 0.001161974 | Up |
|  | A0A2T1LIJ2 | C6985_13475 | Glycerophosphodiester phosphodiesterase OS = *Escherichia coli* OX = 562 GN = C6985_13475 PE = 4 SV = 1 | 1.419667232 | 0.00657461 | Up |
|  | A0A838ALF5 | *dhaM* | Dihydroxyacetone kinase subunit DhaM OS = *Escherichia coli* OX = 562 GN = dhaM PE = 4 SV = 1 | 1.415755739 | 0.002958299 | Up |
|  | A0A4T5JV04 | *mglB* | D-galactose-binding periplasmic protein OS = *Escherichia coli* OX = 562 GN = mglB PE = 3 SV = 1 | 1.415546871 | 0.010308482 | Up |
|  | A0A827RNQ4 | GQW80_12065 | Helix-turn-helix domain-containing protein OS = *Escherichia coli* OX = 562 GN = GQW80_12065 PE = 4 SV = 1 | 1.415515861 | 0.015115237 | Up |
|  | A0A7H9QR77 | HVX31_00565 | Uncharacterized protein OS = *Escherichia coli* OX = 562 GN = HVX31_00565 PE = 4 SV = 1 | 1.414870159 | 0.01842262 | Up |
|  | A0A377LDT9 | *murR_1* | MurR/RpiR family transcriptional regulator OS = *Escherichia coli* OX = 562 GN = murR_1 PE = 4 SV = 1 | 1.414055795 | 0.009989766 | Up |
|  | A0A828R0G1 | *mnmC* | UPF0209 protein yfcK OS = *Escherichia coli* 3431 OX = 670892 GN = mnmC PE = 4 SV = 1 | 1.413297556 | 0.006786008 | Up |
|  | A0A6M0PS45 | *pgm* | Alpha-D-glucose phosphate-specific phosphoglucomutase OS = *Escherichia coli* OX = 562 GN = pgm PE = 3 SV = 1 | 1.413290331 | 0.030641114 | Up |
|  | A0A777TQ89 | *feoB* | Ferrous iron transport protein B OS = *Escherichia coli* OX = 562 GN = feoB PE = 3 SV = 1 | 1.412715771 | 0.011392567 | Up |
|  | A0A828KM58 | HCF72_003305 | Putative DNA-binding transcriptional regulator OS = *Escherichia coli* OX = 562 GN = HCF72_003305 PE = 4 SV = 1 | 1.411709811 | 0.007551363 | Up |
|  | A0A271QSQ8 | *katG* | Catalase-peroxidase OS = *Escherichia coli* OX = 562 GN = katG PE = 3 SV = 1 | 1.410204024 | 0.002371092 | Up |
|  | A0A6L7A3K9 | *nanA* | N-acetylneuraminate lyase (Fragment) OS = *Escherichia coli* OX = 562 GN = nanA PE = 3 SV = 1 | 1.408507183 | 0.00091285 | Up |
|  | A0A6L4XNV3 | *dacC* | Serine-type D-Ala-D-Ala carboxypeptidase OS = *Escherichia coli* OX = 562 GN = dacC PE = 3 SV = 1 | 1.407649574 | 0.001160493 | Up |
|  | A0A7I6H769 | EcHS_A2224 | Uncharacterized protein OS = *Escherichia coli* O9 : H4 (strain HS) OX = 331112 GN = EcHS_A2224 PE = 4 SV = 1 | 1.403643136 | 0.00020522 | Up |
|  | A0A376MVG2 | *phoQ* | Sensor histidine protein kinase/phosphatase PhoQ OS = *Escherichia coli* OX = 562 GN = phoQ PE = 4 SV = 1 | 1.402689625 | 0.004191695 | Up |
|  | A0A7D7DU33 | *hchA* | Protein deglycase HchA OS = *Escherichia coli* OX = 562 GN = hchA PE = 4 SV = 1 | 1.402669841 | 0.003358591 | Up |
|  | A0A376ZTY7 | *ispE* | 4-diphosphocytidyl-2-C-methyl-D-erythritol kinase OS = *Escherichia coli* OX = 562 GN = ispE PE = 3 SV = 1 | 1.400562442 | 0.004737148 | Up |
|  | A0A2X1MYJ8 | *glmS* | Glutamine--fructose-6-phosphate aminotransferase [isomerizing] OS = *Escherichia coli* OX = 562 GN = glmS PE = 3 SV = 1 | 1.399712844 | 0.013788882 | Up |
|  | A0A829JLQ8 | G938_00500 | Peptidyl-prolyl cis-trans isomerase D OS = *Escherichia coli* UMEA 3200-1 OX = 1281213 GN = G938_00500 PE = 4 SV = 1 | 1.399560377 | 0.016477911 | Up |
|  | K4XKM3 | *tnaA* | Tryptophanase/L-cysteine desulfhydrase, PLP-dependent OS = *Escherichia coli* O111 : H11 str. CVM9455 OX = 1165939 GN = tnaA PE = 4 SV = 1 | 1.398948243 | 0.031108742 | Up |
|  | A0A140N469 | ECBD_0292 | Glycerophosphoryl diester phosphodiesterase OS = *Escherichia coli* (strain B / BL21-DE3) OX = 469008 GN = ECBD_0292 PE = 4 SV = 1 | 1.39872721 | 0.012455857 | Up |
|  | A0A377AWG1 | *dacC* | Serine-type D-Ala-D-Ala carboxypeptidase OS = *Escherichia coli* OX = 562 GN = dacC PE = 3 SV = 1 | 1.397742171 | 0.001655349 | Up |
|  | A0A827KTD0 | *minD* | Septum site-determining protein MinD OS = *Escherichia coli* OX = 562 GN = minD PE = 4 SV = 1 | 1.397702311 | 0.013666911 | Up |
|  | A0A6N8PRW7 | *cmk* | Cytidylate kinase OS = *Escherichia coli* OX = 562 GN = cmk PE = 3 SV = 1 | 1.39731058 | 0.000409779 | Up |
|  | A0A2X7H6G4 | *fimD_5* | Outer membrane usher protein FimD OS = *Escherichia coli* OX = 562 GN = fimD_5 PE = 3 SV = 1 | 1.396594511 | 0.006793448 | Up |
|  | A0A826NBH0 | CXJ73_004090 | Putative transporter OS = *Escherichia coli* OX = 562 GN = CXJ73_004090 PE = 4 SV = 1 | 1.394714714 | 0.012564564 | Up |
|  | A0A2U2VLW9 | *glpQ* | Glycerophosphodiester phosphodiesterase OS = *Escherichia coli* OX = 562 GN = glpQ PE = 4 SV = 1 | 1.393260771 | 0.034820874 | Up |
|  | A0A777GNP1 | GF699_00235 | Methyl-accepting chemotaxis protein II OS = *Escherichia coli* OX = 562 GN = GF699_00235 PE = 4 SV = 1 | 1.393023153 | 0.006952539 | Up |
|  | A0A0H2Z3V4 | *gor* | Glutathione reductase Gor OS = *Escherichia coli* O1 : K1 / APEC OX = 405955 GN = gor PE = 3 SV = 1 | 1.390735468 | 0.003560898 | Up |
|  | A0A2X1KKJ6 | *ydgH_1* | Protein YdgH OS = *Escherichia coli* OX = 562 GN = ydgH_1 PE = 4 SV = 1 | 1.390228121 | 0.000840018 | Up |
|  | A0A2X6RA66 | *bssR* | Biofilm formation regulator BssR OS = *Escherichia coli* OX = 562 GN = bssR PE = 4 SV = 1 | 1.390073728 | 0.000382624 | Up |
|  | A0A8A8NRS0 | *gntP* | Gluconate permease GntP OS = *Escherichia coli* OX = 562 GN = gntP PE = 4 SV = 1 | 1.389987457 | 0.010126814 | Up |
|  | A0A6N6X641 | *glpC* | Anaerobic glycerol-3-phosphate dehydrogenase subunit C (Fragment) OS = *Escherichia coli* OX = 562 GN = glpC PE = 4 SV = 1 | 1.385693586 | 0.008395214 | Up |
|  | A0A6D0EIL2 | *nrdD* | Anaerobic ribonucleoside-triphosphate reductase (Fragment) OS = *Escherichia coli* OX = 562 GN = nrdD PE = 4 SV = 1 | 1.385157031 | 0.031814963 | Up |
|  | A0A822UG86 | *ybhF_2* | Putative type I secretion system, ATP-binding protein OS = *Escherichia coli* OX = 562 GN = ybhF_2 PE = 4 SV = 1 | 1.384639774 | 0.001372527 | Up |
|  | A0A377LAY0 | *mioC_1* | Flavodoxin OS = *Escherichia coli* OX = 562 GN = mioC_1 PE = 4 SV = 1 | 1.382137225 | 0.046730182 | Up |
|  | A0A376I2K4 | *aspS* | Aspartate--tRNA ligase OS = *Escherichia coli* OX = 562 GN = aspS PE = 3 SV = 1 | 1.381454534 | 0.01937706 | Up |
|  | A0A1J1DKQ8 | *gnd* | 6-phosphogluconate dehydrogenase, decarboxylating OS = *Escherichia coli* OX = 562 GN = gnd PE = 3 SV = 1 | 1.380952201 | 0.008290699 | Up |
|  | A0A830VKC2 | *oppD* | Murein tripeptide/oligopeptide ABC transporter ATP binding protein OppD OS = *Escherichia coli* OX = 562 GN = oppD PE = 4 SV = 1 | 1.379713503 | 0.039907272 | Up |
|  | A0A377L8B3 | *livF_1* | Leucine/isoleucine/valine transporter ATP-binding subunit OS = *Escherichia coli* OX = 562 GN = livF_1 PE = 4 SV = 1 | 1.378375247 | 0.005014421 | Up |
|  | A0A376VYA9 | *yaeP* | UPF0253 protein YaeP OS = *Escherichia coli* OX = 562 GN = yaeP PE = 3 SV = 1 | 1.376942281 | 0.008950124 | Up |
|  | A0A5B9AYS1 | *ansB* | L-asparaginase 2 OS = *Escherichia coli* OX = 562 GN = ansB PE = 3 SV = 1 | 1.376155373 | 0.022484819 | Up |
|  | A0A0E0XTU6 | *uxaC* | Uronate isomerase OS = *Escherichia coli* O104 : H4 (strain 2011C-3493) OX = 1133852 GN = uxaC PE = 3 SV = 1 | 1.375356878 | 0.011916039 | Up |
|  | A0A6D0ELA1 | GQM09_28295 | Peptidase Do (Fragment) OS = *Escherichia coli* OX = 562 GN = GQM09_28295 PE = 4 SV = 1 | 1.374648624 | 0.02022283 | Up |
|  | A0A1X3KRB9 | *yceH* | UPF0502 protein YceH OS = *Escherichia coli* H605 OX = 656410 GN = yceH PE = 3 SV = 1 | 1.374642496 | 0.016933701 | Up |
|  | A0A0B1LVL6 | PU06_29190 | Toxin YhaV (Fragment) OS = *Escherichia coli* OX = 562 GN = PU06_29190 PE = 4 SV = 1 | 1.374545456 | 0.001737519 | Up |
|  | A0A6M0PYN9 | *glpQ* | Glycerophosphodiester phosphodiesterase OS = *Escherichia coli* OX = 562 GN = glpQ PE = 4 SV = 1 | 1.374301047 | 0.005647005 | Up |
|  | E6BGX8 | *glyA* | Serine hydroxymethyltransferase OS = *Escherichia coli* MS 85-1 OX = 679202 GN = glyA PE = 3 SV = 1 | 1.373127271 | 0.004766543 | Up |
|  | A0A827ELZ6 | B6R17_001942 | Class II glutamine amidotransferase OS = *Escherichia coli* OX = 562 GN = B6R17_001942 PE = 4 SV = 1 | 1.372389557 | 0.004561209 | Up |
|  | A0A377DBF7 | *bfr* | Bacterioferritin OS = *Escherichia coli* OX = 562 GN = bfr PE = 3 SV = 1 | 1.371132617 | 0.005584071 | Up |
|  | A0A777CQL7 | *pdxK* | Pyridoxine/pyridoxal/pyridoxamine kinase OS = *Escherichia coli* OX = 562 GN = pdxK PE = 3 SV = 1 | 1.370232387 | 0.005378319 | Up |
|  | A0A6M0PU94 | *ytfE* | Iron-sulfur cluster repair protein YtfE OS = *Escherichia coli* OX = 562 GN = ytfE PE = 3 SV = 1 | 1.370071898 | 0.014951717 | Up |
|  | W1WA20 | Q609_ECAC02517G0001 | Uncharacterized protein (Fragment) OS = *Escherichia coli* DORA_A_5_14_21 OX = 1403943 GN = Q609_ECAC02517G0001 PE = 4 SV = 1 | 1.36965299 | 0.036800195 | Up |
|  | A0A6M7H4E4 | *nanK* | N-acetylmannosamine kinase OS = *Escherichia coli* O157 : H7 OX = 83334 GN = nanK PE = 3 SV = 1 | 1.366594851 | 0.009776792 | Up |
|  | F4SIU8 | ECHG_01354 | Toxin-antitoxin system, toxin component, HipA family OS = *Escherichia coli* H736 OX = 656414 GN = ECHG_01354 PE = 3 SV = 1 | 1.366150864 | 1.76779E-05 | Up |
|  | D8AEF0 | HMPREF9530_04954 | Sugar-binding domain protein OS = *Escherichia coli* (strain MS 21-1) OX = 749527 GN = HMPREF9530_04954 PE = 4 SV = 1 | 1.366107484 | 0.000332758 | Up |
|  | A0A836N789 | AB05_4678 | Oxidoreductase, NAD-binding Rossmann fold family protein OS = *Escherichia coli* 2-460-02_S1_C1 OX = 1444044 GN = AB05_4678 PE = 4 SV = 1 | 1.365184759 | 0.04725424 | Up |
|  | A0A7H9LX11 | *fucO* | Lactaldehyde reductase OS = *Escherichia coli* OX = 562 GN = fucO PE = 4 SV = 1 | 1.364076264 | 0.006749529 | Up |
|  | A0A2T1LIF6 | C6985_13480 | Glycerol-3-phosphate transporter OS = *Escherichia coli* OX = 562 GN = C6985_13480 PE = 3 SV = 1 | 1.363347972 | 0.023929606 | Up |
|  | A0A2H4TXE8 | CV83915_03928 | Glycerophosphodiester phosphodiesterase OS = *Escherichia coli* OX = 562 GN = CV83915_03928 PE = 4 SV = 1 | 1.361672818 | 0.001565877 | Up |
|  | P76549 | *yffR* | Uncharacterized protein YffR OS = *Escherichia coli* (strain K12) OX = 83333 GN = yffR PE = 3 SV = 1 | 1.360500054 | 0.002550011 | Up |
|  | A0A080IHA6 | AC28_4007 | Uncharacterized protein OS = *Escherichia coli* 1-250-04_S3_C2 OX = 1444163 GN = AC28_4007 PE = 4 SV = 1 | 1.360435404 | 0.0038545 | Up |
|  | A0A827HLW1 | *surE* | 5'/3'-nucleotidase SurE OS = *Escherichia coli* OX = 562 GN = surE PE = 4 SV = 1 | 1.360258933 | 5.66798E-05 | Up |
|  | A0A376U1R7 | *nhaR* | Transcriptional activator NhaR OS = *Escherichia coli* OX = 562 GN = nhaR PE = 3 SV = 1 | 1.35945829 | 0.015202944 | Up |
|  | A0A7B5NX36 | *dgoD* | D-galactonate dehydratase OS = *Escherichia coli* OX = 562 GN = dgoD PE = 3 SV = 1 | 1.359456786 | 0.0182794 | Up |
|  | F4TBP9 | ECJG_00122 | Phosphoglucomutase, alpha-D-glucose phosphate-specific OS = *Escherichia coli* M718 OX = 656419 GN = ECJG_00122 PE = 3 SV = 1 | 1.353839681 | 0.005370612 | Up |
|  | A0A843MJ33 | *nfuA* | Iron-sulfur cluster biogenesis protein NfuA OS = *Escherichia coli* OX = 562 GN = nfuA PE = 4 SV = 1 | 1.353124695 | 0.015806652 | Up |
|  | A0A4S3Z4X7 | CDL57_00615 | DUF1870 family protein OS = *Escherichia coli* OX = 562 GN = CDL57_00615 PE = 4 SV = 1 | 1.352421129 | 0.003219079 | Up |
|  | A0A826QVU1 | *uvrY* | Two-component system response regulator UvrY OS = *Escherichia coli* OX = 562 GN = uvrY PE = 4 SV = 1 | 1.351052714 | 0.028972412 | Up |
|  | A0A829AE96 | A1Y7_02739 | Response regulator in two-component system withYehU OS = *Escherichia coli* KTE119 OX = 1182710 GN = A1Y7_02739 PE = 4 SV = 1 | 1.349717963 | 0.00995051 | Up |
|  | A0A0K3T4P0 | ERS085366_02366 | Putative lipoprotein OS = *Escherichia coli* OX = 562 GN = ERS085366_02366 PE = 4 SV = 1 | 1.348115452 | 0.007862809 | Up |
|  | A0A6M0PW36 | *fdx* | 2Fe-2S ferredoxin OS = *Escherichia coli* OX = 562 GN = fdx PE = 3 SV = 1 | 1.345017191 | 0.019673157 | Up |
|  | A0A6N4KJZ2 | BON98_19775 | Uncharacterized protein OS = *Escherichia coli* OX = 562 GN = BON98_19775 PE = 4 SV = 1 | 1.342350719 | 0.004592678 | Up |
|  | A0A6N8PZ81 | *argH* | Argininosuccinate lyase OS = *Escherichia coli* OX = 562 GN = argH PE = 3 SV = 1 | 1.341758894 | 0.019389117 | Up |
|  | A0A376HW66 | *yajI* | DUF3251 domain-containing protein OS = *Escherichia coli* OX = 562 GN = yajI PE = 4 SV = 1 | 1.340728667 | 0.049151286 | Up |
|  | A0A377HG86 | *qseG_2* | Putative lipoprotein OS = *Escherichia coli* OX = 562 GN = qseG_2 PE = 4 SV = 1 | 1.338752303 | 7.17204E-05 | Up |
|  | A0A6N8Q014 | *groL* | 60 kDa chaperonin OS = *Escherichia coli* OX = 562 GN = groL PE = 3 SV = 1 | 1.338430069 | 0.021592065 | Up |
|  | A0A7D7HDD9 | *kdpB* | Potassium-transporting ATPase ATP-binding subunit OS = *Escherichia coli* OX = 562 GN = kdpB PE = 3 SV = 1 | 1.337970581 | 0.012422306 | Up |
|  | Q0TJT3 | *pgl* | 6-phosphogluconolactonase OS = *Escherichia coli* O6 : K15 : H31 (strain 536 / UPEC) OX = 362663 GN = pgl PE = 3 SV = 1 | 1.337202794 | 0.013507949 | Up |
|  | A0A6D0GXH7 | *pepA* | Probable cytosol aminopeptidase OS = *Escherichia coli* OX = 562 GN = pepA PE = 3 SV = 1 | 1.336804783 | 0.026193811 | Up |
|  | A0A366YTV4 | DS966_02535 | NADP-dependent oxaloacetate-decarboxylating malate dehydrogenase OS = *Escherichia coli* OX = 562 GN = DS966_02535 PE = 3 SV = 1 | 1.3354511 | 0.02171496 | Up |
|  | A0A826V574 | wzzB | LPS O-antigen chain length determinant protein WzzB OS = *Escherichia coli* OX = 562 GN = wzzB PE = 4 SV = 1 | 1.335397154 | 0.028651665 | Up |
|  | A0A376HTN8 | yidR | Putative ATP/GTP-binding protein OS = *Escherichia coli* OX = 562 GN = yidR PE = 4 SV = 1 | 1.334072544 | 0.016551021 | Up |
|  | A0A7U3FRC7 | BWZ24_13830 | Zn-dependent oxidoreductase OS = *Escherichia coli* OX = 562 GN = BWZ24_13830 PE = 3 SV = 1 | 1.332943201 | 0.008379607 | Up |
|  | A0A0K4P4P1 | *kipI* | 5-oxoprolinase subunit PxpB OS = *Escherichia coli* OX = 562 GN = kipI PE = 4 SV = 1 | 1.332860312 | 0.005117258 | Up |
|  | A0A2T1LPS0 | *dnaK* | Chaperone protein DnaK OS = *Escherichia coli* OX = 562 GN = dnaK PE = 2 SV = 1 | 1.331634119 | 0.013001684 | Up |
|  | A0A376P2Q1 | *sucC_2* | Succinyl-CoA synthetase beta chain with ATP-grasp domain OS = *Escherichia coli* OX = 562 GN = sucC_2 PE = 4 SV = 1 | 1.330130509 | 0.040874166 | Up |
|  | A0A2S5UB44 | *proY* | Proline-specific permease ProY OS = *Escherichia coli* OX = 562 GN = proY PE = 4 SV = 1 | 1.329505933 | 0.011895356 | Up |
|  | A0A417ZT69 | *yajL* | Oxidative stress resistance chaperone OS = *Escherichia coli* OX = 562 GN = yajL PE = 4 SV = 1 | 1.329163843 | 0.042139268 | Up |
|  | A0A7H9LV91 | *ldtB* | L,D-transpeptidase OS = *Escherichia coli* OX = 562 GN = ldtB PE = 3 SV = 1 | 1.326120518 | 0.000642093 | Up |
|  | A0A376RLB5 | *artI* | Arginine ABC transporter, substrate-binding protein OS = *Escherichia coli* OX = 562 GN = artI PE = 3 SV = 1 | 1.325420649 | 0.020055308 | Up |
|  | A0A2U2VVI3 | E2123_08350 | ABC transporter substrate-binding protein OS = *Escherichia coli* OX = 562 GN = E2123_08350 PE = 4 SV = 1 | 1.32294378 | 0.000826408 | Up |
|  | A0A3L5AFZ3 | AAS29_000798 | DUF1471 domain-containing protein OS = *Escherichia coli* OX = 562 GN = AAS29_000798 PE = 4 SV = 1 | 1.3227201 | 0.001589996 | Up |
|  | A0A7H9LXR6 | *glcB* | Malate synthase G OS = *Escherichia coli* OX = 562 GN = glcB PE = 3 SV = 1 | 1.322309746 | 0.031293052 | Up |
|  | A0A7I8Z9P3 | ETECE36_02358 | Cytochrome c-type biogenesis protein OS = *Escherichia coli* OX = 562 GN = ETECE36_02358 PE = 3 SV = 1 | 1.321289213 | 0.022921866 | Up |
|  | A0A377DEX1 | *dnaK_4* | Chaperone protein DnaK (Heat shock protein 70) (Heat shock 70 kDaprotein) (HSP70) OS = *Escherichia coli* OX = 562 GN = dnaK_4 PE = 4 SV = 1 | 1.320827104 | 0.014209491 | Up |
|  | A0A2T1LLF6 | C6985_07380 | Outer membrane protein OmpW OS = *Escherichia coli* OX = 562 GN = C6985_07380 PE = 4 SV = 1 | 1.320140599 | 0.017315702 | Up |
|  | A0A5D8RSR4 | E0I42_27095 | Lipoprotein (Fragment) OS = *Escherichia coli* OX = 562 GN = E0I42_27095 PE = 4 SV = 1 | 1.319440459 | 0.01085157 | Up |
|  | A0A5P0J6D0 | EIZ93_05025 | EAL domain-containing protein OS = *Escherichia coli* OX = 562 GN = EIZ93_05025 PE = 4 SV = 1 | 1.319023518 | 0.001969168 | Up |
|  | E6BHM5 | *sbcD* | Nuclease SbcCD subunit D OS = *Escherichia coli* MS 85-1 OX = 679202 GN = sbcD PE = 3 SV = 1 | 1.318625903 | 5.83808E-05 | Up |
|  | A0A2J1D5L3 | *dnaK* | Molecular chaperone DnaK (Fragment) OS = *Escherichia coli* OX = 562 GN = dnaK PE = 4 SV = 1 | 1.31818713 | 0.010218485 | Up |
|  | A0A7A6P9I7 | *dapA* | 4-hydroxy-tetrahydrodipicolinate synthase OS = *Escherichia coli* OX = 562 GN = dapA PE = 3 SV = 1 | 1.317724741 | 0.042431387 | Up |
|  | A0A0L1C3K3 | *fumB* | Fumarate hydratase class I OS = *Escherichia coli* OX = 562 GN = fumB PE = 3 SV = 1 | 1.317542084 | 0.023583836 | Up |
|  | A0A377KE75 | NCTC9075_06183 | Putative lipoprotein OS = *Escherichia coli* OX = 562 GN = NCTC9075_06183 PE = 4 SV = 1 | 1.315206266 | 0.000411217 | Up |
|  | A0A6N0IKN9 | *ag43* | Autotransporter adhesin Ag43 OS = *Escherichia coli* OX = 562 GN = ag43 PE = 4 SV = 1 | 1.313481004 | 0.035504668 | Up |
|  | A0A6H3L0X3 | ELU83_09620 | MmcQ/YjbR family DNA-binding protein OS = *Escherichia coli* OX = 562 GN = ELU83_09620 PE = 4 SV = 1 | 1.311195925 | 0.037295967 | Up |
|  | L7UYD3 | unknow | OmpC porin (Fragment) OS = *Escherichia coli* OX = 562 PE = 3 SV = 1 | 1.310234032 | 0.000422945 | Up |
|  | A0A6G4BWX1 | *eco* | Ecotin OS = *Escherichia coli* OX = 562 GN = eco PE = 3 SV = 1 | 1.310050836 | 0.019819161 | Up |
|  | A0A418H9N3 | *dnaN* | Beta sliding clamp (Fragment) OS = *Escherichia coli* OX = 562 GN = dnaN PE = 3 SV = 1 | 1.309885179 | 0.004611287 | Up |
|  | A0A5N3CXS1 | *pspC* | Envelope stress response membrane protein PspC OS = *Escherichia coli* OX = 562 GN = pspC PE = 4 SV = 1 | 1.30830747 | 0.030619763 | Up |
|  | A0A376LG23 | *groL* | 60 kDa chaperonin OS = *Escherichia coli* OX = 562 GN = groL PE = 3 SV = 1 | 1.307820034 | 0.009626128 | Up |
|  | A0A376KZC4 | *ddlA_1* | D-alanine--D-alanine ligase OS = *Escherichia coli* OX = 562 GN = ddlA_1 PE = 4 SV = 1 | 1.306664476 | 0.03326625 | Up |
|  | A0A7B4HW57 | HMV41_29060 | Glycine--tRNA ligase beta subunit (Fragment) OS = *Escherichia coli* OX = 562 GN = HMV41_29060 PE = 3 SV = 1 | 1.305756418 | 0.00439949 | Up |
|  | A0A376S3L5 | *chiA* | Bifunctional chitinase/lysozyme OS = *Escherichia coli* OX = 562 GN = chiA PE = 4 SV = 1 | 1.30515543 | 0.000311911 | Up |
|  | A0A3L9GVF5 | *tatA* | Sec-independent protein translocase protein TatA OS = *Escherichia coli* OX = 562 GN = tatA PE = 3 SV = 1 | 1.304520057 | 0.000522625 | Up |
|  | A0A3L0W067 | D9F05_14910 | Uncharacterized protein OS = *Escherichia coli* OX = 562 GN = D9F05_14910 PE = 4 SV = 1 | 1.304508807 | 0.011119902 | Up |
|  | A0A3K0JQE8 | D9J61_18285 | FAD-binding protein OS = *Escherichia coli* OX = 562 GN = D9J61_18285 PE = 4 SV = 1 | 1.303661193 | 0.009922838 | Up |
|  | A0A376PXV3 | *ybjD* | ATP-dependent endonuclease OS = *Escherichia coli* OX = 562 GN = ybjD PE = 4 SV = 1 | 1.303413801 | 0.000222185 | Up |
|  | A0A7B5PWP4 | HNC52_18380 | C-type cytochrome OS = *Escherichia coli* OX = 562 GN = HNC52_18380 PE = 4 SV = 1 | 1.30301808 | 0.010917556 | Up |
|  | A0A6L9DEU3 | *ybgC* | Tol-pal system-associated acyl-CoA thioesterase OS = *Escherichia coli* OX = 562 GN = ybgC PE = 3 SV = 1 | 1.302928346 | 0.000299368 | Up |
|  | A0A2X3M0D2 | *ompA_3* | Outer membrane protein A OS = *Escherichia coli* OX = 562 GN = ompA_3 PE = 4 SV = 1 | 1.302900906 | 0.019397952 | Up |
|  | A0A6N8PGH4 | *srkA* | Stress response kinase A OS = *Escherichia coli* OX = 562 GN = srkA PE = 3 SV = 1 | 1.302098525 | 0.00046658 | Up |
|  | Q0TAX9 | *glmU* | Bifunctional protein GlmU OS = *Escherichia coli* O6 : K15 : H31 (strain 536 / UPEC) OX = 362663 GN = glmU PE = 3 SV = 1 | 1.301037941 | 0.000399323 | Up |
|  | A0A376I976 | *yajQ* | UPF0234 protein YajQ OS = *Escherichia coli* OX = 562 GN = yajQ PE = 3 SV = 1 | 1.300820562 | 0.024777897 | Up |
|  | A0A7U2IP63 | *fucU* | L-fucose mutarotase OS = *Escherichia coli* OX = 562 GN = fucU PE = 3 SV = 1 | 1.299837466 | 0.007924011 | Up |
|  | A0A6D0IS19 | *nagK* | N-acetyl-D-glucosamine kinase OS = *Escherichia coli* OX = 562 GN = nagK PE = 3 SV = 1 | 1.29963429 | 0.010728563 | Up |
|  | A0A2X7I0D2 | *pabB* | Aminodeoxychorismate synthase OS = *Escherichia coli* OX = 562 GN = pabB PE = 3 SV = 1 | 1.299273181 | 0.002697733 | Up |
|  | C3SII8 | *sthA* | Soluble pyridine nucleotide transhydrogenase OS = *Escherichia coli* OX = 562 GN = sthA PE = 3 SV = 1 | 1.299246865 | 0.003339121 | Up |
|  | A0A6H3L0C8 | *pgaB* | Poly-beta-1,6-N-acetyl-D-glucosamine N-deacetylase PgaB OS = *Escherichia coli* OX = 562 GN = pgaB PE = 4 SV = 1 | 1.297385538 | 0.002233769 | Up |
|  | A0A2X5XUM8 | *tam* | Trans-aconitate 2-methyltransferase OS = *Escherichia coli* OX = 562 GN = tam PE = 3 SV = 1 | 1.295747849 | 0.035582479 | Up |
|  | A0A2T1LQ55 | C6985_00975 | Ferrichrome porin FhuA OS = *Escherichia coli* OX = 562 GN = C6985_00975 PE = 3 SV = 1 | 1.295728446 | 0.045513724 | Up |
|  | W1W0H4 | Q609_ECAC02897G0001 | Uncharacterized protein (Fragment) OS = *Escherichia coli* DORA_A_5_14_21 OX = 1403943 GN = Q609_ECAC02897G0001 PE = 4 SV = 1 | 1.291963864 | 0.028278475 | Up |
|  | A0A826LIN3 | C5542_002251 | DUF1198 domain-containing protein OS = *Escherichia coli* OX = 562 GN = C5542_002251 PE = 4 SV = 1 | 1.291466359 | 0.02145366 | Up |
|  | A0A6N8QP55 | *ansB* | L-asparaginase 2 (Fragment) OS = *Escherichia coli* OX = 562 GN = ansB PE = 3 SV = 1 | 1.291013218 | 0.029068771 | Up |
|  | A0A7U2WD15 | *idnO* | Gluconate 5-dehydrogenase OS = *Escherichia coli* O84 : H7 OX = 2697517 GN = idnO PE = 3 SV = 1 | 1.290816993 | 0.001572916 | Up |
|  | P64465 | *ydfZ* | Putative selenoprotein YdfZ OS = *Escherichia coli* O157 : H7 OX = 83334 GN = ydfZ PE = 3 SV = 1 | 1.290226204 | 0.015766422 | Up |
|  | Q56UC7 | unknow | Host specificity protein (Fragment) OS = *Escherichia coli* OX = 562 PE = 4 SV = 1 | 1.290178536 | 0.046332303 | Up |
|  | A0A827E0G2 | CX692_001784 | Tryptophanase OS = *Escherichia coli* OX = 562 GN = CX692_001784 PE = 4 SV = 1 | 1.288902512 | 0.036994948 | Up |
|  | A0A826WZK7 | *modE* | Molybdenum-dependent transcriptional regulator OS = *Escherichia coli* OX = 562 GN = modE PE = 4 SV = 1 | 1.287791455 | 0.019879036 | Up |
|  | A0A377A8R1 | *pldA* | Phospholipase A1 OS = *Escherichia coli* OX = 562 GN = pldA PE = 3 SV = 1 | 1.28734479 | 0.000403446 | Up |
|  | S1J706 | A1WU_00826 | Uncharacterized protein OS = *Escherichia coli* KTE108 OX = 1182704 GN = A1WU_00826 PE = 4 SV = 1 | 1.284709512 | 0.003063086 | Up |
|  | A0A827KNR7 | *eutL* | Ethanolamine utilization microcompartment protein EutL OS = *Escherichia coli* OX = 562 GN = eutL PE = 4 SV = 1 | 1.281922879 | 0.00065363 | Up |
|  | A0A6N8Q371 | *rnr* | Ribonuclease R OS = *Escherichia coli* OX = 562 GN = rnr PE = 3 SV = 1 | 1.281309755 | 0.001060085 | Up |
|  | A0A3L9IA13 | EAI46_09730 | LysR family transcriptional regulator OS = *Escherichia coli* OX = 562 GN = EAI46_09730 PE = 3 SV = 1 | 1.280178635 | 0.035483615 | Up |
|  | A0A3L0W137 | *groL* | 60 kDa chaperonin OS = *Escherichia coli* OX = 562 GN = groL PE = 3 SV = 1 | 1.279896239 | 0.01729061 | Up |
|  | A0A6D0M0X1 | GRW80_28915 | UDP-3-O-(3-hydroxymyristoyl)glucosamine N-acyltransferase (Fragment) OS = *Escherichia coli* OX = 562 GN = GRW80_28915 PE = 4 SV = 1 | 1.279627619 | 0.022269076 | Up |
|  | A0A6L4XPH0 | *leuS* | Leucine--tRNA ligase OS = *Escherichia coli* OX = 562 GN = leuS PE = 3 SV = 1 | 1.278848462 | 0.005061399 | Up |
|  | D3QJP5 | *yohN* | Uncharacterized protein OS = *Escherichia coli* O55 : H7 (strain CB9615 / EPEC) OX = 701177 GN = yohN PE = 4 SV = 1 | 1.278837542 | 0.012457148 | Up |
|  | A0A826X2M8 | *cspD* | Cold shock-like protein CspD OS = *Escherichia coli* OX = 562 GN = cspD PE = 4 SV = 1 | 1.277535571 | 0.031671105 | Up |
|  | A0A844UUA4 | *galF* | UTP--glucose-1-phosphate uridylyltransferase GalF (Fragment) OS = *Escherichia coli* OX = 562 GN = galF PE = 4 SV = 1 | 1.277065207 | 0.014226171 | Up |
|  | A0A6L4XKF2 | *sppA* | Protease 4 OS = *Escherichia coli* OX = 562 GN = sppA PE = 3 SV = 1 | 1.277043512 | 0.019532188 | Up |
|  | L3CC44 | A13W_00503 | Isomerase/hydrolase OS = *Escherichia coli* KTE193 OX = 1181739 GN = A13W_00503 PE = 4 SV = 1 | 1.27658415 | 0.006185645 | Up |
|  | A0A376I3W0 | *bglX_1* | Periplasmic beta-glucosidase OS = *Escherichia coli* OX = 562 GN = bglX_1 PE = 3 SV = 1 | 1.274852223 | 0.000144024 | Up |
|  | A0A376HZX6 | *cobT* | Nicotinate-nucleotide--dimethylbenzimidazole phosphoribosyltransferase OS = *Escherichia coli* OX = 562 GN = cobT PE = 3 SV = 1 | 1.274316708 | 0.039065238 | Up |
|  | A0A826X499 | CSE52_001296 | Fumarate hydratase OS = *Escherichia coli* OX = 562 GN = CSE52_001296 PE = 4 SV = 1 | 1.27414089 | 0.031445151 | Up |
|  | A0A830VI41 | GF698_09710 | Amino-acid N-acetyltransferase OS = *Escherichia coli* OX = 562 GN = GF698_09710 PE = 4 SV = 1 | 1.273512509 | 0.009281061 | Up |
|  | A0A7I8HMX8 | blaCTX-M-157 | Extended-spectrum beta-lactamase OS = *Escherichia coli* OX = 562 GN = blaCTX-M-157 PE = 4 SV = 1 | 1.273335483 | 0.034643866 | Up |
|  | P0AE89 | *cpxR* | Transcriptional regulatory protein CpxR OS = *Escherichia coli* O157 : H7 OX = 83334 GN = cpxR PE = 3 SV = 1 | 1.273313515 | 0.001970101 | Up |
|  | A0A376I4U7 | NCTC9094_03243 | Putative fatty acid binding protein OS = *Escherichia coli* OX = 562 GN = NCTC9094_03243 PE = 4 SV = 1 | 1.273259585 | 0.042136018 | Up |
|  | P0ABE2 | *bolA* | DNA-binding transcriptional regulator BolA OS = *Escherichia coli* (strain K12) OX = 83333 GN = bolA PE = 1 SV = 1 | 1.272480354 | 0.023604716 | Up |
|  | A0A376D3K9 | *ispD* | Bifunctional enzyme IspD/IspF OS = *Escherichia coli* OX = 562 GN = ispD PE = 3 SV = 1 | 1.272099253 | 0.020610696 | Up |
|  | U9XLC8 | HMPREF1589_05305 | Formate dehydrogenase, gamma subunit OS = *Escherichia coli* 113290 OX = 1268976 GN = HMPREF1589_05305 PE = 3 SV = 1 | 1.270720086 | 0.001621054 | Up |
|  | A0A7T2N4B4 | *fliA* | RNA polymerase sigma factor FliA OS = *Escherichia coli* OX = 562 GN = fliA PE = 4 SV = 1 | 1.270363837 | 0.007035794 | Up |
|  | A0A2X7IEI8 | *kdsB* | 3-deoxy-manno-octulosonate cytidylyltransferase OS = *Escherichia coli* OX = 562 GN = kdsB PE = 3 SV = 1 | 1.270204827 | 0.028819042 | Up |
|  | A0A827LBS1 | *focA* | Formate transporter FocA OS = *Escherichia coli* OX = 562 GN = focA PE = 4 SV = 1 | 1.270192567 | 0.001240445 | Up |
|  | A0A2A3WWM5 | BB545_05105 | NADH oxidoreductase OS = *Escherichia coli* OX = 562 GN = BB545_05105 PE = 4 SV = 1 | 1.269952831 | 0.02399803 | Up |
|  | A0A7H9QJD6 | *tusA* | Sulfur carrier protein TusA OS = *Escherichia coli* OX = 562 GN = tusA PE = 3 SV = 1 | 1.269669505 | 0.000178589 | Up |
|  | A0A2T3TC58 | *ydcF* | YdcF family protein OS = *Escherichia coli* OX = 562 GN = ydcF PE = 4 SV = 1 | 1.269015032 | 0.000566076 | Up |
|  | A0A5D8SZL8 | E0I42_08635 | NAD(P)H oxidoreductase OS = *Escherichia coli* OX = 562 GN = E0I42_08635 PE = 4 SV = 1 | 1.26839825 | 0.008715479 | Up |
|  | A0A826LFD7 | C5542_001192 | DeoR/GlpR transcriptional regulator OS = *Escherichia coli* OX = 562 GN = C5542_001192 PE = 4 SV = 1 | 1.267381624 | 3.71758E-05 | Up |
|  | A0A2I8SX17 | C2U48_31755 | DUF3313 domain-containing protein OS = *Escherichia coli* OX = 562 GN = C2U48_31755 PE = 4 SV = 1 | 1.266884687 | 0.008204125 | Up |
|  | A0A3L0VY65 | *mglB* | D-galactose-binding periplasmic protein OS = *Escherichia coli* OX = 562 GN = mglB PE = 3 SV = 1 | 1.26643596 | 0.027267349 | Up |
|  | A0A6C9QTS1 | *eutM* | Ethanolamine utilization microcompartment protein EutM OS = *Escherichia coli* OX = 562 GN = eutM PE = 4 SV = 1 | 1.265767672 | 0.000313473 | Up |
|  | A0A376ZHZ8 | *mdaB* | Modulator of drug activity B OS = *Escherichia coli* OX = 562 GN = mdaB PE = 4 SV = 1 | 1.265517383 | 0.006864401 | Up |
|  | A0A862ZHA6 | *glpA* | Anaerobic glycerol-3-phosphate dehydrogenase subunit A OS = *Escherichia coli* O145 OX = 1055538 GN = glpA PE = 4 SV = 1 | 1.263999696 | 0.01252959 | Up |
|  | A0A831F693 | *allB* | Allantoinase AllB OS = *Escherichia coli* OX = 562 GN = allB PE = 4 SV = 1 | 1.263090837 | 0.002100866 | Up |
|  | A0A827RU54 | *bioD* | ATP-dependent dethiobiotin synthetase BioD OS = *Escherichia coli* OX = 562 GN = bioD PE = 4 SV = 1 | 1.262344561 | 0.010155291 | Up |
|  | A0A6M1HKQ4 | G5632_14320 | Protein QmcA OS = *Escherichia coli* OX = 562 GN = G5632_14320 PE = 3 SV = 1 | 1.262133003 | 0.008787697 | Up |
|  | A0A7D7DVZ1 | *cusR* | Copper response regulator transcription factor CusR OS = *Escherichia coli* OX = 562 GN = cusR PE = 4 SV = 1 | 1.260944061 | 0.00183786 | Up |
|  | F4T6A9 | ECIG_04883 | Sulfofructose kinase OS = *Escherichia coli* M605 OX = 656417 GN = ECIG_04883 PE = 3 SV = 1 | 1.260885173 | 0.018498008 | Up |
|  | A0A1X3IZZ0 | ECXG_00371 | Regulatory protein SdiA OS = *Escherichia coli* TA447 OX = 656447 GN = ECXG_00371 PE = 4 SV = 1 | 1.260417297 | 0.005434796 | Up |
|  | A0A8A8NQQ1 | *idnD* | L-idonate 5-dehydrogenase OS = *Escherichia coli* OX = 562 GN = idnD PE = 4 SV = 1 | 1.25951462 | 0.01123417 | Up |
|  | A0A2W6Q640 | *grcA* | Autonomous glycyl radical cofactor OS = *Escherichia coli* OX = 562 GN = grcA PE = 3 SV = 1 | 1.258336149 | 0.044724642 | Up |
|  | A0A829DWE3 | *yciS* | Inner membrane protein yciS OS = *Escherichia coli* 2875150 OX = 1116036 GN = yciS PE = 4 SV = 1 | 1.258014285 | 0.003341927 | Up |
|  | A0A2X1NZD1 | *yhbL_1* | Isoprenoid biosynthesis protein with amidotransferase-like domain OS = *Escherichia coli* OX = 562 GN = yhbL_1 PE = 4 SV = 1 | 1.257771164 | 0.003934446 | Up |
|  | A0A829DRF6 | *mazG* | Nucleoside triphosphate pyrophosphohydrolase OS = *Escherichia coli* 2875150 OX = 1116036 GN = mazG PE = 4 SV = 1 | 1.256046305 | 0.003452106 | Up |
|  | A0A6D0DAQ1 | FQ021_12490 | DUF1877 family protein OS = *Escherichia coli* OX = 562 GN = FQ021_12490 PE = 4 SV = 1 | 1.256027477 | 0.000635497 | Up |
|  | A0A768K1M3 | *yejL* | UPF0352 protein YejL OS = *Escherichia coli* OX = 562 GN = yejL PE = 3 SV = 1 | 1.255966722 | 0.002022357 | Up |
|  | A0A485JCI0 | *glpQ* | Glycerophosphodiester phosphodiesterase OS = *Escherichia coli* OX = 562 GN = glpQ PE = 4 SV = 1 | 1.25412153 | 0.000520221 | Up |
|  | A0A3W5Y3R9 | *dkgB* | 2,5-didehydrogluconate reductase DkgB OS = *Escherichia coli* OX = 562 GN = dkgB PE = 4 SV = 1 | 1.252409221 | 0.038834514 | Up |
|  | A0A376JV02 | *cutA* | Divalent-cation tolerance protein CutA OS = *Escherichia coli* OX = 562 GN = cutA PE = 3 SV = 1 | 1.25160548 | 0.04139236 | Up |
|  | A0A827UGM1 | *argE* | Acetylornithine deacetylase OS = *Escherichia coli* OX = 562 GN = argE PE = 4 SV = 1 | 1.24998304 | 0.007655231 | Up |
|  | A0A6G4BN73 | *purT* | Formate-dependent phosphoribosylglycinamide formyltransferase OS = *Escherichia coli* OX = 562 GN = purT PE = 3 SV = 1 | 1.249827119 | 0.002082747 | Up |
|  | F4T7V4 | *frdD* | Fumarate reductase subunit D OS = *Escherichia coli* M605 OX = 656417 GN = frdD PE = 3 SV = 1 | 1.249761462 | 0.005161352 | Up |
|  | A0A241QPT0 | *kdbD* | Histidine kinase OS = *Escherichia coli* OX = 562 GN = kdbD PE = 4 SV = 1 | 1.248036939 | 0.004270688 | Up |
|  | A0A0H3PT65 | ECH7EC869_1354 | Putative transcriptional accessory protein OS = *Escherichia coli* O157 : H7 (strain EC869) OX = 478008 GN = ECH7EC869_1354 PE = 4 SV = 1 | 1.247427917 | 0.040846731 | Up |
|  | A0A826VXD8 | *dpiB* | Sensor histidine kinase DpiB OS = *Escherichia coli* OX = 562 GN = dpiB PE = 4 SV = 1 | 1.246805177 | 0.012349256 | Up |
|  | A0A6N8NDP3 | *grcA* | Autonomous glycyl radical cofactor OS = *Escherichia coli* OX = 562 GN = grcA PE = 3 SV = 1 | 1.246366108 | 0.019892819 | Up |
|  | A0A8A5ILA3 | *maeB* | NADP-dependent oxaloacetate-decarboxylating malate dehydrogenase OS = *Escherichia coli* O176 : H45 OX = 2810408 GN = maeB PE = 4 SV = 1 | 1.246016195 | 0.005085875 | Up |
|  | A0A6N8K5V0 | GQF59_28550 | DNA replication protein DnaC (Fragment) OS = *Escherichia coli* OX = 562 GN = GQF59_28550 PE = 4 SV = 1 | 1.244755784 | 0.018723124 | Up |
|  | A0A6C9FXZ7 | *nnrD* | Bifunctional NAD(P)H-hydrate repair enzyme OS = *Escherichia coli* OX = 562 GN = nnrD PE = 3 SV = 1 | 1.244449643 | 0.000105581 | Up |
|  | A0A7D7PKU0 | *cpdB* | 2',3'-cyclic-nucleotide 2'-phosphodiesterase/3'-nucleotidase OS = *Escherichia coli* OX = 562 GN = cpdB PE = 3 SV = 1 | 1.243053825 | 0.017183712 | Up |
|  | D8A5G6 | *atoS* | Histidine kinase OS = *Escherichia coli* (strain MS 21-1) OX = 749527 GN = atoS PE = 4 SV = 1 | 1.242166364 | 0.029694387 | Up |
|  | A0A8A7UCV4 | *thrS* | Threonine--tRNA ligase OS = *Escherichia coli* OX = 562 GN = thrS PE = 4 SV = 1 | 1.241596123 | 0.025892731 | Up |
|  | A0A862ZK76 | *ansB* | L-asparaginase 2 OS = *Escherichia coli* O145 OX = 1055538 GN = ansB PE = 4 SV = 1 | 1.240651617 | 0.004516363 | Up |
|  | A0A1X3LKC0 | EAXG_01325 | Putative outer membrane protein OS = *Escherichia coli* TA054 OX = 656433 GN = EAXG_01325 PE = 4 SV = 1 | 1.240465396 | 0.026329948 | Up |
|  | Q8XAA8 | Z3776 | Ancillary SecYEG translocon subunit OS = *Escherichia coli* O157 : H7 OX = 83334 GN = Z3776 PE = 3 SV = 2 | 1.239686765 | 0.042933337 | Up |
|  | A0A5F1DTT1 | *upp* | Uracil phosphoribosyltransferase OS = *Escherichia coli* OX = 562 GN = upp PE = 3 SV = 1 | 1.239176654 | 0.011140491 | Up |
|  | V0ALG0 | HMPREF1620_00678 | Protein ErfK/srfK OS = *Escherichia coli* 909945-2 OX = 1269007 GN = HMPREF1620_00678 PE = 3 SV = 1 | 1.239164191 | 0.005312763 | Up |
|  | A0A795GC53 | HNO08_10605 | FMN_red domain-containing protein OS = *Escherichia coli* OX = 562 GN = HNO08_10605 PE = 4 SV = 1 | 1.237094116 | 0.040880286 | Up |
|  | A0A3L5JMX3 | D9J03_16065 | Alcohol dehydrogenase OS = *Escherichia coli* OX = 562 GN = D9J03_16065 PE = 3 SV = 1 | 1.2368761 | 0.015836493 | Up |
|  | A0A376RTV4 | *mrdB* | Multifunctional fusion protein OS = *Escherichia coli* OX = 562 GN = mrdB PE = 3 SV = 1 | 1.236466197 | 0.020867259 | Up |
|  | A0A827LNK9 | *oppA* | Oligopeptide ABC transporter substrate-binding protein OppA OS = *Escherichia coli* OX = 562 GN = oppA PE = 4 SV = 1 | 1.235069315 | 0.004744313 | Up |
|  | A0A827L354 | *lysA* | Diaminopimelate decarboxylase OS = *Escherichia coli* OX = 562 GN = lysA PE = 4 SV = 1 | 1.233429045 | 0.016796472 | Up |
|  | C4ZT96 | *frsA* | Esterase FrsA OS = *Escherichia coli* (strain K12 / MC4100 / BW2952) OX = 595496 GN = frsA PE = 3 SV = 1 | 1.233297644 | 0.004259815 | Up |
|  | Q1R3V3 | *argC* | N-acetyl-gamma-glutamyl-phosphate reductase OS = *Escherichia coli* (strain UTI89 / UPEC) OX = 364106 GN = argC PE = 3 SV = 1 | 1.231810231 | 0.009012267 | Up |
|  | B1LNX6 | *dcd* | dCTP deaminase OS = *Escherichia coli* (strain SMS-3-5 / SECEC) OX = 439855 GN = dcd PE = 3 SV = 1 | 1.231167781 | 0.002449698 | Up |
|  | A0A3L9GWX8 | *htpG* | Chaperone protein HtpG OS = *Escherichia coli* OX = 562 GN = htpG PE = 3 SV = 1 | 1.230052984 | 0.008455242 | Up |
|  | A0A827VF59 | F2N31_00175 | [protein-PII] uridylyltransferase OS = *Escherichia coli* OX = 562 GN = F2N31_00175 PE = 4 SV = 1 | 1.229486099 | 0.000828394 | Up |
|  | A0A4Q0E774 | *fabD* | Malonyl CoA-acyl carrier protein transacylase OS = *Escherichia coli* OX = 562 GN = fabD PE = 3 SV = 1 | 1.229484888 | 0.016907949 | Up |
|  | A0A1Y2XU03 | AW059_04955 | DNA-binding response regulator OS = *Escherichia coli* OX = 562 GN = AW059_04955 PE = 4 SV = 1 | 1.229282642 | 0.000269924 | Up |
|  | A0A6N8PP74 | *tusE* | Sulfurtransferase OS = *Escherichia coli* OX = 562 GN = tusE PE = 3 SV = 1 | 1.22906131 | 0.024325269 | Up |
|  | A0A5B9AWW1 | *yajQ* | UPF0234 protein YajQ OS = *Escherichia coli* OX = 562 GN = yajQ PE = 3 SV = 1 | 1.228571745 | 0.011058755 | Up |
|  | A0A7D7PI10 | *cobS* | Adenosylcobinamide-GDP ribazoletransferase OS = *Escherichia coli* OX = 562 GN = cobS PE = 3 SV = 1 | 1.226292698 | 0.00074907 | Up |
|  | P76177 | *ydgH* | Protein YdgH OS = *Escherichia coli* (strain K12) OX = 83333 GN = ydgH PE = 1 SV = 1 | 1.225562039 | 0.010666516 | Up |
|  | A0A8B3HZ19 | EIA13_03215 | Cyclopropane fatty acyl phospholipid synthase OS = *Escherichia coli* OX = 562 GN = EIA13_03215 PE = 4 SV = 1 | 1.225478541 | 0.001153472 | Up |
|  | A0A417ZTA1 | D3O91_18030 | Na+/H+ antiporter OS = *Escherichia coli* OX = 562 GN = D3O91_18030 PE = 3 SV = 1 | 1.225261938 | 0.001854989 | Up |
|  | A0A6D0H6B2 | *yraP* | Divisome-associated lipoprotein YraP OS = *Escherichia coli* OX = 562 GN = yraP PE = 4 SV = 1 | 1.224435177 | 0.008184105 | Up |
|  | A0A2U9LBT1 | AM464_00550 | Alpha-amylase OS = *Escherichia coli* OX = 562 GN = AM464_00550 PE = 3 SV = 1 | 1.224379723 | 0.036666302 | Up |
|  | P11072 | *lit* | Cell death peptidase OS = *Escherichia coli* (strain K12) OX = 83333 GN = lit PE = 3 SV = 2 | 1.223438259 | 0.004214227 | Up |
|  | A0A0F3TEV8 | *pntA* | NAD(P) transhydrogenase subunit alpha OS = *Escherichia coli* OX = 562 GN = pntA PE = 3 SV = 1 | 1.223328328 | 0.003492305 | Up |
|  | A0A376RBM1 | *frlR* | DNA-binding transcriptional regulator FrlR OS = *Escherichia coli* OX = 562 GN = frlR PE = 4 SV = 1 | 1.223020581 | 0.009331211 | Up |
|  | A0A6D1D2E5 | G5595_24695 | Transcriptional repressor PurR (Fragment) OS = *Escherichia coli* OX = 562 GN = G5595_24695 PE = 4 SV = 1 | 1.22269684 | 0.044697268 | Up |
|  | A0A0K5BCV2 | *gcvA* | Putative transcriptional regulator of glycine cleavage system OS = *Escherichia coli* OX = 562 GN = gcvA PE = 3 SV = 1 | 1.221782449 | 0.000334126 | Up |
|  | A0A376VH61 | *napH* | Ferredoxin-type protein NapH OS = *Escherichia coli* OX = 562 GN = napH PE = 4 SV = 1 | 1.220532466 | 0.003027036 | Up |
|  | A0A376SIQ2 | *glyA_2* | Serine hydroxymethyltransferase OS = *Escherichia coli* OX = 562 GN = glyA_2 PE = 4 SV = 1 | 1.220310471 | 0.044443634 | Up |
|  | A0A6D0F9L4 | *uxuR* | Uxu operon transcriptional regulator OS = *Escherichia coli* OX = 562 GN = uxuR PE = 4 SV = 1 | 1.220143742 | 0.000849593 | Up |
|  | A0A403LPP3 | DLX40_17495 | DeoR/GlpR transcriptional regulator OS = *Escherichia coli* OX = 562 GN = DLX40_17495 PE = 4 SV = 1 | 1.219876848 | 0.011823991 | Up |
|  | A0A7A2WXH1 | *pntB* | Re/Si-specific NAD(P)(+) transhydrogenase subunit beta OS = *Escherichia coli* OX = 562 GN = pntB PE = 4 SV = 1 | 1.219273983 | 0.000359294 | Up |
|  | A0A454A4V4 | ECP_1768 | Uncharacterized protein YebO OS = *Escherichia coli* O6 : K15 : H31 (strain 536 / UPEC) OX = 362663 GN = ECP_1768 PE = 4 SV = 1 | 1.219070138 | 0.000328438 | Up |
|  | A0A2T1LH34 | *gshA* | Glutamate--cysteine ligase OS = *Escherichia coli* OX = 562 GN = gshA PE = 3 SV = 1 | 1.219034823 | 0.005908157 | Up |
|  | A0A2X7FW49 | SAMEA3753300_00011 | DNA injection protein OS = *Escherichia coli* OX = 562 GN = SAMEA3753300_00011 PE = 4 SV = 1 | 1.217618482 | 0.024779088 | Up |
|  | V9H1J8 | *cat* | Chloramphenicol acetyltransferase OS = *Escherichia coli* OX = 562 GN = cat PE = 3 SV = 1 | 1.217394667 | 0.021478708 | Up |
|  | A0A6M1HH28 | G5632_02220 | RpoE-regulated lipoprotein OS = *Escherichia coli* OX = 562 GN = G5632_02220 PE = 4 SV = 1 | 1.217349905 | 0.03430343 | Up |
|  | A0A345DL33 | *traI* | Multifunctional conjugation protein TraI OS = *Escherichia coli* OX = 562 GN = traI PE = 4 SV = 1 | 1.216112694 | 0.004111266 | Up |
|  | A0A826HVW3 | *htpG* | Molecular chaperone HtpG OS = *Escherichia coli* O108 : H9 OX = 2100504 GN = htpG PE = 4 SV = 1 | 1.215473943 | 0.048370356 | Up |
|  | A0A4U9U1E4 | *cpdB* | 2',3'-cyclic-nucleotide 2'-phosphodiesterase/3'-nucleotidase OS = *Escherichia coli* OX = 562 GN = cpdB PE = 3 SV = 1 | 1.215241664 | 0.03354798 | Up |
|  | P77293 | *yfdH* | Prophage bactoprenol glucosyl transferase homolog OS = *Escherichia coli* (strain K12) OX = 83333 GN = yfdH PE = 1 SV = 1 | 1.214741588 | 0.038346662 | Up |
|  | A0A376VC73 | *glgA_2* | Glycogen synthase OS = *Escherichia coli* OX = 562 GN = glgA_2 PE = 4 SV = 1 | 1.213607529 | 0.034766994 | Up |
|  | B1XGY3 | *argG* | Argininosuccinate synthase OS = *Escherichia coli* (strain K12 / DH10B) OX = 316385 GN = argG PE = 3 SV = 1 | 1.213522876 | 0.026351457 | Up |
|  | A0A7U2ATC7 | *dgcZ* | Diguanylate cyclase DgcZ OS = *Escherichia coli* OX = 562 GN = dgcZ PE = 4 SV = 1 | 1.213442126 | 0.000273939 | Up |
|  | W1WJ79 | Q609_ECAC02049G0001 | Nitrogen regulation protein NRII (Fragment) OS = *Escherichia coli* DORA_A_5_14_21 OX = 1403943 GN = Q609_ECAC02049G0001 PE = 4 SV = 1 | 1.212686712 | 0.029472361 | Up |
|  | A0A2W6Q4G2 | DNQ45_07465 | Type I site-specific deoxyribonuclease OS = *Escherichia coli* OX = 562 GN = DNQ45_07465 PE = 3 SV = 1 | 1.2125605 | 0.016717503 | Up |
|  | A0A829L0M0 | HMPREF1602_03474 | Uncharacterized protein OS = *Escherichia coli* 907889 OX = 1268989 GN = HMPREF1602_03474 PE = 4 SV = 1 | 1.212490551 | 0.024791058 | Up |
|  | Q1RA34 | *rfbB* | dTDP-glucose 4,6-dehydratase OS = *Escherichia coli* (strain UTI89 / UPEC) OX = 364106 GN = rfbB PE = 3 SV = 1 | 1.212282345 | 0.039469259 | Up |
|  | A0A2X3K8E1 | *rpoC_5* | DNA-directed RNA polymerase OS = *Escherichia coli* OX = 562 GN = rpoC_5 PE = 4 SV = 1 | 1.212142 | 0.028092734 | Up |
|  | A0A853WGK5 | *copA* | Cu+ exporting ATPase OS = *Escherichia coli* OX = 562 GN = copA PE = 4 SV = 1 | 1.211259681 | 0.015952606 | Up |
|  | A0A6G6KXJ4 | *rsuA* | Pseudouridine synthase OS = *Escherichia coli* OX = 562 GN = rsuA PE = 3 SV = 1 | 1.211230373 | 0.003548159 | Up |
|  | A0A7I9AJI9 | *appY_1* | AppY_1 protein OS = *Escherichia coli* OX = 562 GN = appY_1 PE = 4 SV = 1 | 1.210670536 | 0.036291092 | Up |
|  | A0A7L5L5F3 | *aroB* | 3-dehydroquinate synthase OS = *Escherichia coli* OX = 562 GN = aroB PE = 3 SV = 1 | 1.210659178 | 0.004176064 | Up |
|  | A0A659GVV1 | *tnaA* | Tryptophanase OS = *Escherichia coli* OX = 562 GN = tnaA PE = 3 SV = 1 | 1.210252036 | 0.01374365 | Up |
|  | A0A6L7A9I6 | *modA* | Molybdate ABC transporter substrate-binding protein (Fragment) OS = *Escherichia coli* OX = 562 GN = modA PE = 3 SV = 1 | 1.209935282 | 0.041271895 | Up |
|  | A0A1X3KIE5 | EATG_02172 | Transcriptional regulatory protein OS = *Escherichia coli* H605 OX = 656410 GN = EATG_02172 PE = 4 SV = 1 | 1.20928838 | 0.00041483 | Up |
|  | A0A6N8Q332 | GRW77_20505 | 2',3'-cyclic-nucleotide 2'-phosphodiesterase/3'-nucleotidase OS = *Escherichia coli* OX = 562 GN = GRW77_20505 PE = 3 SV = 1 | 1.207759963 | 0.02294214 | Up |
|  | A0A5F1DV27 | *glmM* | Phosphoglucosamine mutase OS = *Escherichia coli* OX = 562 GN = glmM PE = 3 SV = 1 | 1.207586166 | 0.004815872 | Up |
|  | A0A0K9TBS3 | ERYG_02569 | Phosphotransferase system OS = *Escherichia coli* M114 OX = 656416 GN = ERYG_02569 PE = 4 SV = 1 | 1.207507113 | 0.012737681 | Up |
|  | A0A2X1Q327 | *yjjK_4* | Putative ABC transporter ATP-binding protein YjjK OS = *Escherichia coli* OX = 562 GN = yjjK_4 PE = 4 SV = 1 | 1.20686659 | 0.032261041 | Up |
|  | A0A2U9KS47 | *narX_2* | Histidine kinase OS = *Escherichia coli* OX = 562 GN = narX_2 PE = 4 SV = 1 | 1.206621076 | 0.01869994 | Up |
|  | A0A830TAZ3 | *flgE* | Flagellar hook protein FlgE OS = *Escherichia coli* OX = 562 GN = flgE PE = 4 SV = 1 | 1.206544796 | 0.009871573 | Up |
|  | A0A376L9J5 | *ypdA* | Histidine kinase OS = *Escherichia coli* OX = 562 GN = ypdA PE = 4 SV = 1 | 1.206447646 | 0.038560985 | Up |
|  | A0A2Y0SKJ1 | *prlF* | Antitoxin PrlF OS = *Escherichia coli* OX = 562 GN = prlF PE = 4 SV = 1 | 1.206175493 | 0.000636202 | Up |
|  | A0A7D7I6K9 | *pmbA* | Metalloprotease PmbA OS = *Escherichia coli* OX = 562 GN = pmbA PE = 3 SV = 1 | 1.205937951 | 0.017869068 | Up |
|  | A0A5D8SZC0 | *sbcC* | Nuclease SbcCD subunit C OS = *Escherichia coli* OX = 562 GN = sbcC PE = 3 SV = 1 | 1.205927102 | 7.26755E-05 | Up |
|  | A0A7H9LUI2 | *lolB* | Outer-membrane lipoprotein LolB OS = *Escherichia coli* OX = 562 GN = lolB PE = 3 SV = 1 | 1.20589479 | 0.036031661 | Up |
|  | A0A484Y4B5 | *udp_1* | Uridine phosphorylase OS = *Escherichia coli* OX = 562 GN = udp_1 PE = 4 SV = 1 | 1.205746368 | 0.046947612 | Up |
|  | A0A826XFR5 | FA868_13820 | ATP-dependent DNA helicase OS = *Escherichia coli* OX = 562 GN = FA868_13820 PE = 4 SV = 1 | 1.2053596 | 0.040551615 | Up |
|  | A0A1Q4PJK3 | *ypfN* | UPF0370 protein YpfN OS = *Escherichia coli* OX = 562 GN = ypfN PE = 3 SV = 1 | 1.20531947 | 0.000207794 | Up |
|  | P23331 | *tdk* | Thymidine kinase OS = *Escherichia coli* (strain K12) OX = 83333 GN = tdk PE = 3 SV = 1 | 1.205157015 | 0.003127068 | Up |
|  | P61950 | *fldA* | Flavodoxin 1 OS = *Escherichia coli* O6 : H1 (strain CFT073 / ATCC 700928 / UPEC) OX = 199310 GN = fldA PE = 3 SV = 2 | 1.204793419 | 0.00423771 | Up |
|  | A0A6N6XAM7 | GP662_31915 | Glucose-1-phosphate thymidylyltransferase (Fragment) OS = *Escherichia coli* OX = 562 GN = GP662_31915 PE = 3 SV = 1 | 1.204764264 | 0.007369108 | Up |
|  | D6J6Q8 | ECEG_03416 | PKS_ER domain-containing protein OS = *Escherichia coli* B354 OX = 550677 GN = ECEG_03416 PE = 3 SV = 1 | 1.203269987 | 0.014015865 | Up |
|  | A0A826W9B9 | FC554_15080 | HPr family phosphocarrier protein OS = *Escherichia coli* OX = 562 GN = FC554_15080 PE = 4 SV = 1 | 1.203167756 | 0.000765164 | Up |
|  | A0A5F1T656 | *grpE* | Protein GrpE OS = *Escherichia coli* OX = 562 GN = grpE PE = 3 SV = 1 | 1.203099744 | 0.002553469 | Up |
|  | E2QDK2 | *metG* | Methionine--tRNA ligase OS = *Escherichia coli* LF82 OX = 591946 GN = metG PE = 3 SV = 1 | 1.202754192 | 0.023741662 | Up |
|  | A0A6G4C3L9 | G4V04_14210 | Sulfatase-like hydrolase/transferase OS = *Escherichia coli* OX = 562 GN = G4V04_14210 PE = 3 SV = 1 | 1.202430476 | 0.000381643 | Up |
|  | A0A376LAI3 | *aroF* | Phospho-2-dehydro-3-deoxyheptonate aldolase OS = *Escherichia coli* OX = 562 GN = aroF PE = 3 SV = 1 | 1.202322621 | 0.028093373 | Up |
|  | A0A0A0H460 | EL80_1222 | Ethanolamine utilization protein EutK OS = *Escherichia coli* OX = 562 GN = EL80_1222 PE = 4 SV = 1 | 1.202027332 | 0.003829172 | Up |
|  | A0A5B9AL91 | *sfsA* | Sugar fermentation stimulation protein A OS = *Escherichia coli* OX = 562 GN = sfsA PE = 3 SV = 1 | 1.201746765 | 0.001143582 | Up |
|  | A0A826X793 | *mscM* | Miniconductance mechanosensitive channel MscM OS = *Escherichia coli* OX = 562 GN = mscM PE = 4 SV = 1 | 0.833198642 | 0.025142961 | Down |
|  | A0A826VW02 | *gpmM* | 2,3-bisphosphoglycerate-independent phosphoglycerate mutase OS = *Escherichia coli* OX = 562 GN = gpmM PE = 4 SV = 1 | 0.832743587 | 0.021865228 | Down |
|  | A0A6N8QIT2 | *ileS* | Isoleucine--tRNA ligase (Fragment) OS = *Escherichia coli* OX = 562 GN = ileS PE = 3 SV = 1 | 0.832736794 | 0.007700885 | Down |
|  | A0A827X5P6 | *ahpF* | Alkyl hydroperoxide reductase subunit F OS = *Escherichia coli* OX = 562 GN = ahpF PE = 4 SV = 1 | 0.832491268 | 0.003171929 | Down |
|  | A0A193LMB8 | GJ11_00930 | Chaperone protein Skp OS = *Escherichia coli* OX = 562 GN = GJ11_00930 PE = 3 SV = 1 | 0.832142106 | 0.006526226 | Down |
|  | A0A6D0J7W9 | *xseA* | Exodeoxyribonuclease 7 large subunit OS = *Escherichia coli* OX = 562 GN = xseA PE = 3 SV = 1 | 0.832125022 | 0.008215718 | Down |
|  | A0A376ZME3 | *efp* | Elongation factor P OS = *Escherichia coli* OX = 562 GN = efp PE = 3 SV = 1 | 0.8317854 | 0.011716905 | Down |
|  | A0A1X3KR39 | EATG_00585 | Putative ATP-binding component of a transport system OS = *Escherichia coli* H605 OX = 656410 GN = EATG_00585 PE = 4 SV = 1 | 0.831552919 | 0.036453461 | Down |
|  | A0A0K9T7F8 | ERYG_05682 | Uncharacterized protein OS = *Escherichia coli* M114 OX = 656416 GN = ERYG_05682 PE = 4 SV = 1 | 0.831186649 | 0.034834403 | Down |
|  | A0A0E1T1H7 | *caiB* | L-carnitine CoA-transferase OS = *Escherichia coli* 53638 OX = 344610 GN = caiB PE = 3 SV = 1 | 0.830675604 | 0.013189325 | Down |
|  | Q9LA28 | *tyrR* | Transcriptional regulatory protein TyrR OS = *Escherichia coli* O157 : H7 OX = 83334 GN = tyrR PE = 4 SV = 2 | 0.830529211 | 0.000832972 | Down |
|  | A0A4D9Z8J3 | ELX70_02700 | LysR family transcriptional regulator OS = *Escherichia coli* OX = 562 GN = ELX70_02700 PE = 3 SV = 1 | 0.830194406 | 0.007564231 | Down |
|  | A0A838AWA8 | *lpxB* | Lipid-A-disaccharide synthase OS = *Escherichia coli* OX = 562 GN = lpxB PE = 4 SV = 1 | 0.829915748 | 0.007707922 | Down |
|  | A0A3K2V1M3 | *mocA* | Molybdenum cofactor cytidylyltransferase OS = *Escherichia coli* OX = 562 GN = mocA PE = 4 SV = 1 | 0.829845421 | 0.030068464 | Down |
|  | A0A2Y0BSI6 | *yhbY* | Putative RNA-binding protein OS = *Escherichia coli* OX = 562 GN = yhbY PE = 4 SV = 1 | 0.829672179 | 0.017200945 | Down |
|  | A0A376HSY3 | *gpmI* | 2,3-bisphosphoglycerate-independent phosphoglycerate mutase OS = *Escherichia coli* OX = 562 GN = gpmI PE = 3 SV = 1 | 0.829144552 | 0.00421147 | Down |
|  | A0A6M0PR83 | *rpsB* | 30S ribosomal protein S2 OS = *Escherichia coli* OX = 562 GN = rpsB PE = 3 SV = 1 | 0.828886585 | 0.009528871 | Down |
|  | A0A2T1LHI4 | *pepB* | Peptidase B OS = *Escherichia coli* OX = 562 GN = pepB PE = 3 SV = 1 | 0.82857966 | 0.007523609 | Down |
|  | W8ZI01 | EC958_1807 | Probable dimethyl sulfoxide reductase chain YnfF OS = *Escherichia coli* O25b : H4-ST131 OX = 941322 GN = EC958_1807 PE = 4 SV = 1 | 0.828394309 | 0.02408034 | Down |
|  | A0A6L7A5B5 | *nirD* | Nitrite reductase small subunit NirD (Fragment) OS = *Escherichia coli* OX = 562 GN = nirD PE = 4 SV = 1 | 0.828226139 | 0.042816104 | Down |
|  | A0A3G4RTB5 | D0368_00202 | Sulfonamide-resistant dihydropteroate synthase Sul2 OS = *Escherichia coli* OX = 562 GN = D0368_00202 PE = 4 SV = 1 | 0.828134563 | 0.024534969 | Down |
|  | A0A6M0PWP9 | G4V03_12435 | Nucleoside-specific channel-forming protein Tsx OS = *Escherichia coli* OX = 562 GN = G4V03_12435 PE = 3 SV = 1 | 0.828065846 | 0.04011567 | Down |
|  | A0A2X1N9Q1 | *yhbN* | Lipopolysaccharide transport periplasmic protein LptA OS = *Escherichia coli* OX = 562 GN = yhbN PE = 4 SV = 1 | 0.828011302 | 0.01189808 | Down |
|  | A0A377CHB4 | *aceF_2* | Acetyltransferase component of pyruvate dehydrogenase complex OS = *Escherichia coli* OX = 562 GN = aceF_2 PE = 3 SV = 1 | 0.827923896 | 0.000960333 | Down |
|  | L3CFU3 | A13W_00233 | Glutaredoxin-2 OS = *Escherichia coli* KTE193 OX = 1181739 GN = A13W_00233 PE = 4 SV = 1 | 0.827859602 | 0.006635665 | Down |
|  | A0A1X3LVF6 | EAZG_00618 | Galactose-1-phosphate uridylyltransferase OS = *Escherichia coli* TA249 OX = 656441 GN = EAZG_00618 PE = 3 SV = 1 | 0.827315262 | 0.004125624 | Down |
|  | A0A376Z9V2 | *rbsC* | D-ribose ABC transporter permease OS = *Escherichia coli* OX = 562 GN = rbsC PE = 4 SV = 1 | 0.82724434 | 0.011982846 | Down |
|  | P0ACV8 | *ymjA* | Uncharacterized protein YmjA OS = *Escherichia coli* (strain K12) OX = 83333 GN = ymjA PE = 4 SV = 1 | 0.826945321 | 0.002320358 | Down |
|  | A0A3L0VT52 | *lon* | Lon protease OS = *Escherichia coli* OX = 562 GN = lon PE = 2 SV = 1 | 0.826757523 | 0.032972368 | Down |
|  | A0A0K4HJP4 | *ycgC* | Phosphoenolpyruvate--glycerone phosphotransferase OS = *Escherichia coli* OX = 562 GN = ycgC PE = 3 SV = 1 | 0.826731617 | 0.01627571 | Down |
|  | A0A3U1V7R9 | EHD79_09920 | Inositol-1-monophosphatase OS = *Escherichia coli* OX = 562 GN = EHD79_09920 PE = 3 SV = 1 | 0.825353379 | 0.003161499 | Down |
|  | A0A377FA98 | NCTC9706_00114 | IS186, transposase OS = *Escherichia coli* OX = 562 GN = NCTC9706_00114 PE = 4 SV = 1 | 0.825302652 | 0.000442862 | Down |
|  | A0A0A0FCP4 | EL76_2820 | Dihydroxyacetone kinase subunit K OS = *Escherichia coli* G3/10 OX = 1455601 GN = EL76_2820 PE = 4 SV = 1 | 0.825235857 | 0.006361683 | Down |
|  | V0SFH5 | *clpP* | ATP-dependent Clp protease proteolytic subunit OS = *Escherichia coli* 907672 OX = 1268982 GN = clpP PE = 3 SV = 1 | 0.824759233 | 0.000405071 | Down |
|  | A0A377ABT5 | *rplF* | 50S ribosomal protein L6 OS = *Escherichia coli* OX = 562 GN = rplF PE = 3 SV = 1 | 0.824058976 | 0.017530266 | Down |
|  | A0A7U9A075 | ECFG_04560 | YajO OS = *Escherichia coli* FVEC1302 OX = 656379 GN = ECFG_04560 PE = 4 SV = 1 | 0.82386818 | 0.001054372 | Down |
|  | A0A377D334 | *trpC* | Multifunctional fusion protein OS = *Escherichia coli* OX = 562 GN = trpC PE = 3 SV = 1 | 0.823412131 | 0.003345289 | Down |
|  | A0A2I6IHS3 | BVL39_24760 | Phospho-2-dehydro-3-deoxyheptonate aldolase OS = *Escherichia coli* OX = 562 GN = BVL39_24760 PE = 3 SV = 1 | 0.823359714 | 0.001086204 | Down |
|  | A0A3L0VYM4 | D9F05_06435 | AMP nucleosidase OS = *Escherichia coli* OX = 562 GN = D9F05_06435 PE = 4 SV = 1 | 0.823141821 | 0.036071697 | Down |
|  | A0A5N8HK80 | FVB16_31090 | Single-stranded DNA-binding protein SSB1 (Fragment) OS = *Escherichia coli* OX = 562 GN = FVB16_31090 PE = 3 SV = 1 | 0.822945072 | 0.000910564 | Down |
|  | A0A826XBG0 | *gatB* | PTS galactitol transporter subunit IIB OS = *Escherichia coli* OX = 562 GN = gatB PE = 4 SV = 1 | 0.822778397 | 0.040375938 | Down |
|  | A0A376HWP7 | *srlD* | Sorbitol-6-phosphate 2-dehydrogenase (Glucitol-6-phosphate dehydrogenase) OS = *Escherichia coli* OX = 562 GN = srlD PE = 3 SV = 1 | 0.821720241 | 0.037048805 | Down |
|  | A0A5N3D643 | *dld* | Quinone-dependent D-lactate dehydrogenase OS = *Escherichia coli* OX = 562 GN = dld PE = 3 SV = 1 | 0.821430888 | 0.011314754 | Down |
|  | A0A1X3KG34 | *bipA* | 50S ribosomal subunit assembly factor BipA OS = *Escherichia coli* H605 OX = 656410 GN = bipA PE = 3 SV = 1 | 0.821310595 | 0.012010997 | Down |
|  | Q0TCE3 | *rplW* | 50S ribosomal protein L23 OS = *Escherichia coli* O6 : K15 : H31 (strain 536 / UPEC) OX = 362663 GN = rplW PE = 3 SV = 1 | 0.820382581 | 0.032034743 | Down |
|  | A0A376JGA1 | *rplT* | 50S ribosomal protein L20 OS = *Escherichia coli* OX = 562 GN = rplT PE = 3 SV = 1 | 0.820163666 | 0.036496011 | Down |
|  | A0A826Y2T3 | *potC* | Spermidine/putrescine ABC transporter permease PotC OS = *Escherichia coli* OX = 562 GN = potC PE = 4 SV = 1 | 0.819739679 | 0.035147003 | Down |
|  | P52627 | *fliZ* | Regulator of sigma S factor FliZ OS = *Escherichia coli* (strain K12) OX = 83333 GN = fliZ PE = 2 SV = 1 | 0.8190146 | 0.021762519 | Down |
|  | A0A345ESS5 | *rcsB* | Transcriptional regulatory protein RcsB OS = *Escherichia coli* OX = 562 GN = rcsB PE = 3 SV = 1 | 0.818908096 | 0.005468885 | Down |
|  | A0A8B5F6C9 | BON70_13510 | 23S rRNA pseudouridylate synthase OS = *Escherichia coli* OX = 562 GN = BON70_13510 PE = 4 SV = 1 | 0.818713384 | 0.023897817 | Down |
|  | A0A6N8PVK8 | *rpsB* | 30S ribosomal protein S2 OS = *Escherichia coli* OX = 562 GN = rpsB PE = 3 SV = 1 | 0.818637413 | 0.036512162 | Down |
|  | A0A826X5I1 | *uvrA* | Excinuclease ABC subunit UvrA OS = *Escherichia coli* OX = 562 GN = uvrA PE = 4 SV = 1 | 0.818586934 | 0.007813037 | Down |
|  | A0A376Q1Q5 | *uvrA* | UvrABC system protein A OS = *Escherichia coli* OX = 562 GN = uvrA PE = 3 SV = 1 | 0.818397823 | 0.000299925 | Down |
|  | F4NR29 | *btuB* | Vitamin B12 transporter BtuB OS = *Escherichia coli* D9 OX = 556266 GN = btuB PE = 3 SV = 1 | 0.818278351 | 0.00019674 | Down |
|  | A0A5B9AQ57 | *endA* | Deoxyribonuclease I OS = *Escherichia coli* OX = 562 GN = endA PE = 3 SV = 1 | 0.817609145 | 0.006311882 | Down |
|  | A0A0K4PAK9 | *ackA* | Acetate kinase OS = *Escherichia coli* OX = 562 GN = ackA PE = 3 SV = 1 | 0.817068629 | 0.009572481 | Down |
|  | A0A6D0H0Q9 | GRW77_16270 | Sugar kinase OS = *Escherichia coli* OX = 562 GN = GRW77_16270 PE = 4 SV = 1 | 0.817009639 | 0.000155008 | Down |
|  | S1ECA0 | A1UI_03248 | DUF927 domain-containing protein OS = *Escherichia coli* KTE73 OX = 1182680 GN = A1UI_03248 PE = 4 SV = 1 | 0.817003303 | 0.004887075 | Down |
|  | A0A3Z6G1M5 | *mukB* | Chromosome partition protein MukB OS = *Escherichia coli* OX = 562 GN = mukB PE = 3 SV = 1 | 0.816426257 | 0.020156424 | Down |
|  | A7ZQ46 | *rplS* | 50S ribosomal protein L19 OS = *Escherichia coli* O139 : H28 (strain E24377A / ETEC) OX = 331111 GN = rplS PE = 3 SV = 1 | 0.816340739 | 0.03203921 | Down |
|  | A0A193LRH5 | GJ11_08715 | Cytochrome B OS = *Escherichia coli* OX = 562 GN = GJ11_08715 PE = 4 SV = 1 | 0.816292654 | 0.009673941 | Down |
|  | W1F112 | unknow | Uncharacterized protein OS = *Escherichia coli* ISC7 OX = 1432555 PE = 4 SV = 1 | 0.816145458 | 0.007776866 | Down |
|  | A0A0B5HCB2 | *crp* | Catabolite activator protein OS = *Escherichia coli* OX = 562 GN = crp PE = 4 SV = 1 | 0.816042533 | 0.008756493 | Down |
|  | A0A4Z0K042 | *atpH* | ATP synthase subunit delta OS = *Escherichia coli* OX = 562 GN = atpH PE = 3 SV = 1 | 0.815988521 | 1.85663E-05 | Down |
|  | A0A376WBS3 | *rplJ* | 50S ribosomal protein L10 OS = *Escherichia coli* OX = 562 GN = rplJ PE = 3 SV = 1 | 0.815141694 | 0.011784709 | Down |
|  | A0A377KA41 | *ybeD* | Protein YbeD OS = *Escherichia coli* OX = 562 GN = ybeD PE = 3 SV = 1 | 0.814229102 | 0.01426754 | Down |
|  | A0A6D1AHG7 | G3563_28575 | Phosphotriesterase (Fragment) OS = *Escherichia coli* OX = 562 GN = G3563_28575 PE = 3 SV = 1 | 0.81327182 | 0.009632001 | Down |
|  | A0A5D8SN25 | *purK* | N5-carboxyaminoimidazole ribonucleotide synthase OS = *Escherichia coli* OX = 562 GN = purK PE = 3 SV = 1 | 0.813141107 | 0.027689699 | Down |
|  | A0A2X1N7G4 | *gcvP_2* | Glycine dehydrogenase OS = *Escherichia coli* OX = 562 GN = gcvP_2 PE = 4 SV = 1 | 0.813015001 | 0.046028459 | Down |
|  | A0A2Y8JQV3 | *ycjG* | Dipeptide epimerase OS = *Escherichia coli* OX = 562 GN = ycjG PE = 3 SV = 1 | 0.812388517 | 0.005652476 | Down |
|  | P46887 | *yecH* | Uncharacterized protein YecH OS = *Escherichia coli* (strain K12) OX = 83333 GN = yecH PE = 4 SV = 2 | 0.81219728 | 0.031608688 | Down |
|  | A0A5F1T115 | DAH34_20040 | Conjugative transfer relaxase/helicase TraI OS = *Escherichia coli* OX = 562 GN = DAH34_20040 PE = 4 SV = 1 | 0.812190159 | 0.028909221 | Down |
|  | P11349 | *narH* | Respiratory nitrate reductase 1 beta chain OS = *Escherichia coli* (strain K12) OX = 83333 GN = narH PE = 1 SV = 3 | 0.812166814 | 0.008420261 | Down |
|  | A0A3P4Z1A7 | *dhaL* | PEP-dependent dihydroxyacetone kinase, ADP-binding subunit DhaL OS = *Escherichia coli* OX = 562 GN = dhaL PE = 4 SV = 1 | 0.811983625 | 0.000112719 | Down |
|  | A0A6N8QR01 | GRW57_33070 | Tagatose-bisphosphate aldolase (Fragment) OS = *Escherichia coli* OX = 562 GN = GRW57_33070 PE = 4 SV = 1 | 0.8109541 | 0.002296507 | Down |
|  | A0A5F0Q1S1 | ELT49_20835 | YicC family protein OS = *Escherichia coli* OX = 562 GN = ELT49_20835 PE = 4 SV = 1 | 0.810661572 | 0.000900777 | Down |
|  | I2STI4 | *phsB* | Thiosulfate reductase electron transport protein phsb OS = *Escherichia coli* 1.2264 OX = 869675 GN = phsB PE = 4 SV = 1 | 0.810358384 | 0.005355384 | Down |
|  | A0A377CHE3 | *aceE_1* | Pyruvate dehydrogenase E1 component OS = *Escherichia coli* OX = 562 GN = aceE_1 PE = 4 SV = 1 | 0.810358073 | 0.014688149 | Down |
|  | A0A7U9FWK5 | ECKG_01746 | Chemotaxis protein CheA OS = *Escherichia coli* TA206 OX = 656440 GN = ECKG_01746 PE = 4 SV = 1 | 0.809813819 | 0.00729853 | Down |
|  | A0A7L5L228 | *pta* | Phosphate acetyltransferase OS = *Escherichia coli* OX = 562 GN = pta PE = 3 SV = 1 | 0.808867777 | 3.92871E-05 | Down |
|  | A1AGC3 | *rpsI* | 30S ribosomal protein S9 OS = *Escherichia coli* O1 : K1 / APEC OX = 405955 GN = rpsI PE = 3 SV = 1 | 0.808182141 | 0.037841831 | Down |
|  | A0A827EG33 | *citE* | Citrate (Pro-3S)-lyase subunit beta OS = *Escherichia coli* OX = 562 GN = citE PE = 4 SV = 1 | 0.808109274 | 0.030504124 | Down |
|  | A0A7B5B9V3 | *clpX* | ATP-dependent Clp protease ATP-binding subunit ClpX OS = *Escherichia coli* OX = 562 GN = clpX PE = 3 SV = 1 | 0.807953547 | 0.000958321 | Down |
|  | A0A222QNW9 | A610_2752 | Cytochrome c-type protein OS = *Escherichia coli* NCCP15648 OX = 1200752 GN = A610_2752 PE = 3 SV = 1 | 0.807642136 | 0.030574526 | Down |
|  | A0A2Y0KUW1 | *mgtA* | Magnesium-transporting ATPase, P-type 1 OS = *Escherichia coli* OX = 562 GN = mgtA PE = 3 SV = 1 | 0.807545744 | 0.035296288 | Down |
|  | A0A210C8F2 | *pepB* | Peptidase B OS = *Escherichia coli* OX = 562 GN = pepB PE = 3 SV = 1 | 0.806588032 | 0.000126754 | Down |
|  | A0A773MCP9 | *yegD* | Molecular chaperone OS = *Escherichia coli* OX = 562 GN = yegD PE = 3 SV = 1 | 0.806496736 | 0.026838493 | Down |
|  | A0A6M1I489 | G5632_29080 | GMP synthase (glutamine-hydrolyzing) (Fragment) OS = *Escherichia coli* OX = 562 GN = G5632_29080 PE = 4 SV = 1 | 0.805954939 | 0.009359055 | Down |
|  | A0A789RQS2 | *rpsD* | 30S ribosomal protein S4 OS = *Escherichia coli* OX = 562 GN = rpsD PE = 3 SV = 1 | 0.805626827 | 0.015963376 | Down |
|  | A0A0K4MBS7 | *trmJ* | tRNA (cytidine/uridine-2'-O-)-methyltransferase TrmJ OS = *Escherichia coli* OX = 562 GN = trmJ PE = 3 SV = 1 | 0.80553823 | 0.009057735 | Down |
|  | A0A7D5L4D1 | *cgtA* | GTPase Obg OS = *Escherichia coli* OX = 562 GN = cgtA PE = 3 SV = 1 | 0.805511457 | 0.018393067 | Down |
|  | A0A7L7E9P5 | *pepN* | Aminopeptidase N OS = *Escherichia coli* OX = 562 GN = pepN PE = 3 SV = 1 | 0.805480952 | 0.031895196 | Down |
|  | A0A827G257 | *map* | Type I methionyl aminopeptidase OS = *Escherichia coli* OX = 562 GN = map PE = 4 SV = 1 | 0.805468033 | 0.010819288 | Down |
|  | A0A2H9EVR4 | *aceE* | Pyruvate dehydrogenase E1 component OS = *Escherichia coli* OX = 562 GN = aceE PE = 4 SV = 1 | 0.805448517 | 0.001637932 | Down |
|  | A0A6M1HI81 | *ruvX* | Putative pre-16S rRNA nuclease OS = *Escherichia coli* OX = 562 GN = ruvX PE = 3 SV = 1 | 0.804922674 | 0.002963463 | Down |
|  | A0A7A2WVB2 | AAS29_000632 | DedA family protein OS = *Escherichia coli* OX = 562 GN = AAS29_000632 PE = 4 SV = 1 | 0.804589668 | 0.04186964 | Down |
|  | A0A4Y9XHJ6 | *gatZ* | D-tagatose-1,6-bisphosphate aldolase subunit GatZ OS = *Escherichia coli* OX = 562 GN = gatZ PE = 3 SV = 1 | 0.804112779 | 0.038728444 | Down |
|  | A0A377CSN6 | *rplB* | 50S ribosomal protein L2 OS = *Escherichia coli* OX = 562 GN = rplB PE = 3 SV = 1 | 0.803993874 | 0.021907012 | Down |
|  | A0A7U9LLG9 | *uxaC* | Uronate isomerase OS = *Escherichia coli* O145 : H28 OX = 1078034 GN = uxaC PE = 3 SV = 1 | 0.80368555 | 0.011885491 | Down |
|  | A0A4P8BZI1 | CCU01_012670 | Methionine-R-sulfoxide reductase OS = *Escherichia coli* O145 : NM OX = 991919 GN = CCU01_012670 PE = 4 SV = 1 | 0.803672151 | 0.003296002 | Down |
|  | A0A376I4P1 | *gltP_2* | Proton/glutamate-aspartate symporter OS = *Escherichia coli* OX = 562 GN = gltP_2 PE = 3 SV = 1 | 0.80334894 | 0.005592558 | Down |
|  | A0A376Q109 | *aceF* | Acetyltransferase component of pyruvate dehydrogenase complex OS = *Escherichia coli* OX = 562 GN = aceF PE = 3 SV = 1 | 0.80296137 | 0.000136206 | Down |
|  | A0A6M1HUT8 | *map* | Methionine aminopeptidase (Fragment) OS = *Escherichia coli* OX = 562 GN = map PE = 3 SV = 1 | 0.802403807 | 0.002702786 | Down |
|  | A0A0A0G354 | *dbpA* | ATP-dependent RNA helicase DbpA OS = *Escherichia coli* OX = 562 GN = dbpA PE = 3 SV = 1 | 0.802318047 | 0.002662164 | Down |
|  | B7UK44 | *rplC* | 50S ribosomal protein L3 OS = *Escherichia coli* O127 : H6 (strain E2348/69 / EPEC) OX = 574521 GN = rplC PE = 3 SV = 1 | 0.801564386 | 0.032184645 | Down |
|  | A0A2X9QCI8 | *hnr* | Regulator of RpoS OS = *Escherichia coli* OX = 562 GN = hnr PE = 3 SV = 1 | 0.80145991 | 0.00969787 | Down |
|  | A0A2T1LIT3 | *glnS* | Glutamine--tRNA ligase OS = *Escherichia coli* OX = 562 GN = glnS PE = 3 SV = 1 | 0.801345698 | 0.004083818 | Down |
|  | A0A838AU16 | *rne* | Ribonuclease E OS = *Escherichia coli* OX = 562 GN = rne PE = 4 SV = 1 | 0.801242608 | 0.03512651 | Down |
|  | A0A5D8SK84 | *alsE* | D-allulose-6-phosphate 3-epimerase OS = *Escherichia coli* OX = 562 GN = alsE PE = 3 SV = 1 | 0.801204768 | 0.009924655 | Down |
|  | A0A0K3STS8 | *flgK* | Flagellar hook-associated protein 1 OS = *Escherichia coli* OX = 562 GN = flgK PE = 3 SV = 1 | 0.800627295 | 0.006955731 | Down |
|  | A0A4T4Z8E0 | *yjgA* | UPF0307 protein YjgA OS = *Escherichia coli* OX = 562 GN = yjgA PE = 3 SV = 1 | 0.800264609 | 0.000587583 | Down |
|  | D6JGC2 | ECEG_03823 | Elongation factor Tu (Fragment) OS = *Escherichia coli* B354 OX = 550677 GN = ECEG_03823 PE = 4 SV = 1 | 0.800162524 | 0.043616331 | Down |
|  | A0A899NBG1 | *trfA_3* | Plasmid replication initiator protein TrfA OS = *Escherichia coli* OX = 562 GN = trfA_3 PE = 4 SV = 1 | 0.800043142 | 0.047252269 | Down |
|  | B6HZL7 | *nusB* | Transcription antitermination protein NusB OS = *Escherichia coli* (strain SE11) OX = 409438 GN = nusB PE = 3 SV = 1 | 0.800013309 | 0.001184525 | Down |
|  | E6BD92 | HMPREF9350_00052 | Phospho-2-dehydro-3-deoxyheptonate aldolase OS = *Escherichia coli* MS 85-1 OX = 679202 GN = HMPREF9350_00052 PE = 3 SV = 1 | 0.798858044 | 0.038180703 | Down |
|  | A0A826X8H6 | CSE52_003051 | YchJ family protein OS = *Escherichia coli* OX = 562 GN = CSE52_003051 PE = 4 SV = 1 | 0.797917886 | 0.042485803 | Down |
|  | A0A836NFZ9 | *grxA* | Glutaredoxin, GrxA family OS = *Escherichia coli* 2-460-02_S1_C1 OX = 1444044 GN = grxA PE = 4 SV = 1 | 0.79565777 | 0.008307319 | Down |
|  | A0A827BBU1 | *gatZ* | Tagatose-bisphosphate aldolase subunit GatZ OS = *Escherichia coli* OX = 562 GN = gatZ PE = 4 SV = 1 | 0.795621609 | 0.013137897 | Down |
|  | A0A3K2YEL7 | D9J61_17275 | 2-oxoglutarate reductase OS = *Escherichia coli* OX = 562 GN = D9J61_17275 PE = 3 SV = 1 | 0.794636738 | 0.014468613 | Down |
|  | B1X6D5 | *tsaC* | Threonylcarbamoyl-AMP synthase OS = *Escherichia coli* (strain K12 / DH10B) OX = 316385 GN = tsaC PE = 3 SV = 1 | 0.79428534 | 0.004545863 | Down |
|  | A0A845P5Y3 | GUB92_17650 | Cytochrome o ubiquinol oxidase subunit III OS = *Escherichia coli* OX = 562 GN = GUB92_17650 PE = 4 SV = 1 | 0.794200298 | 0.011142123 | Down |
|  | P0AD50 | *yfiA* | Ribosome-associated factor Y OS = *Escherichia coli* O6 : H1 (strain CFT073 / ATCC 700928 / UPEC) OX = 199310 GN = yfiA PE = 3 SV = 2 | 0.7941895 | 0.029828937 | Down |
|  | A0A1M2EKK4 | BK375_23085 | DrsE domain-containing protein OS = *Escherichia coli* OX = 562 GN = BK375_23085 PE = 4 SV = 1 | 0.794058578 | 0.01183406 | Down |
|  | D6JGR5 | ECEG_04110 | Uncharacterized protein OS = *Escherichia coli* B354 OX = 550677 GN = ECEG_04110 PE = 4 SV = 1 | 0.794041042 | 0.005225682 | Down |
|  | A0A2X7Q036 | *pgk* | Phosphoglycerate kinase OS = *Escherichia coli* OX = 562 GN = pgk PE = 3 SV = 1 | 0.793214385 | 0.0018961 | Down |
|  | A0A0U4BD45 | *gapA* | Glyceraldehyde-3-phosphate dehydrogenase OS = *Escherichia coli* OX = 562 GN = gapA PE = 3 SV = 1 | 0.79292 | 0.0345304 | Down |
|  | P0A6W5 | *greA* | Transcription elongation factor GreA OS = *Escherichia coli* (strain K12) OX = 83333 GN = greA PE = 1 SV = 1 | 0.792722965 | 0.045839536 | Down |
|  | A0A6L4XH05 | *gcvT* | Aminomethyltransferase OS = *Escherichia coli* OX = 562 GN = gcvT PE = 3 SV = 1 | 0.792682608 | 0.011696364 | Down |
|  | P32053 | *intA* | Prophage integrase IntA OS = *Escherichia coli* (strain K12) OX = 83333 GN = intA PE = 1 SV = 2 | 0.792181984 | 0.001639475 | Down |
|  | A0A1D3KW65 | *rpsS* | 30S ribosomal protein S19 OS = *Escherichia coli* OX = 562 GN = rpsS PE = 3 SV = 1 | 0.791950514 | 0.00924335 | Down |
|  | A0A2W6PJW8 | DNQ45_29425 | RNA chaperone ProQ (Fragment) OS = *Escherichia coli* OX = 562 GN = DNQ45_29425 PE = 3 SV = 1 | 0.791713779 | 0.007519686 | Down |
|  | A0A7A2WX01 | *yebG* | DNA damage-inducible protein YebG OS = *Escherichia coli* OX = 562 GN = yebG PE = 4 SV = 1 | 0.791606596 | 0.020072438 | Down |
|  | A0A829L4W9 | HMPREF1602_03860 | 6-phosphofructokinase OS = *Escherichia coli* 907889 OX = 1268989 GN = HMPREF1602_03860 PE = 4 SV = 1 | 0.791533321 | 0.011639265 | Down |
|  | A0A7U1WB44 | JNN57_14335 | ATP-binding protein OS = *Escherichia coli* OX = 562 GN = JNN57_14335 PE = 4 SV = 1 | 0.791286732 | 0.042816341 | Down |
|  | A0A6C9KSF1 | *flgI* | Flagellar P-ring protein OS = *Escherichia coli* OX = 562 GN = flgI PE = 3 SV = 1 | 0.791014816 | 0.01365568 | Down |
|  | A0A8B4NPI8 | *prfB* | Peptide chain release factor 2 OS = *Escherichia coli* OX = 562 GN = prfB PE = 4 SV = 1 | 0.790798557 | 0.040631625 | Down |
|  | A0A827DZ73 | A2F99_001257 | LysR family transcriptional regulator OS = *Escherichia coli* OX = 562 GN = A2F99_001257 PE = 4 SV = 1 | 0.790760371 | 0.011205519 | Down |
|  | A0A5B9AS13 | *rpsH* | 30S ribosomal protein S8 OS = *Escherichia coli* OX = 562 GN = rpsH PE = 3 SV = 1 | 0.790233417 | 0.007547581 | Down |
|  | A0A828HF64 | *gatB* | PTS galactitol transporter subunit IIB OS = *Escherichia coli* OX = 562 GN = gatB PE = 4 SV = 1 | 0.790149992 | 0.000257491 | Down |
|  | A0A8A5IMA1 | JSU10_12210 | SymE family type I addiction module toxin OS = *Escherichia coli* H20 OX = 2810409 GN = JSU10_12210 PE = 4 SV = 1 | 0.790038569 | 0.033972924 | Down |
|  | A0A8A5IAW5 | *acrB* | Efflux RND transporter permease AcrB OS = *Escherichia coli* O176 : H45 OX = 2810408 GN = acrB PE = 4 SV = 1 | 0.789264494 | 0.007898667 | Down |
|  | A0A5D8STB8 | *cheR* | Chemotaxis protein methyltransferase OS = *Escherichia coli* OX = 562 GN = cheR PE = 4 SV = 1 | 0.788290747 | 0.029967422 | Down |
|  | A0A3A6RSW6 | D3Y67_32810 | Phosphocarrier protein Hpr (Fragment) OS = *Escherichia coli* OX = 562 GN = D3Y67_32810 PE = 4 SV = 1 | 0.788223145 | 0.005340652 | Down |
|  | A0A2T1LCI8 | *rplK* | 50S ribosomal protein L11 OS = *Escherichia coli* OX = 562 GN = rplK PE = 3 SV = 1 | 0.788062244 | 0.013653348 | Down |
|  | A0A2X7G578 | *ndh* | NADH dehydrogenase OS = *Escherichia coli* OX = 562 GN = ndh PE = 4 SV = 1 | 0.787620391 | 0.000806881 | Down |
|  | A0A6N9S2P9 | *tsf* | Elongation factor Ts OS = *Escherichia coli* OX = 562 GN = tsf PE = 3 SV = 1 | 0.786755605 | 0.011383867 | Down |
|  | A0A5R8TDT4 | EWT59_07740 | 30S ribosomal protein S12 methylthiotransferase accessory protein YcaO OS = *Escherichia coli* O25b : H4 OX = 941280 GN = EWT59_07740 PE = 4 SV = 1 | 0.786721651 | 0.000404911 | Down |
|  | A0A376I0W6 | *cheZ* | Protein phosphatase CheZ OS = *Escherichia coli* OX = 562 GN = cheZ PE = 3 SV = 1 | 0.786661097 | 0.039847549 | Down |
|  | A0A7U9BHM8 | ECOG_05349 | Elongation factor Tu (EF-Tu) (P-43) OS = *Escherichia coli* H299 OX = 656393 GN = ECOG_05349 PE = 4 SV = 1 | 0.786528872 | 0.031091047 | Down |
|  | C3SRM0 | ECs4127 | Biotin carboxyl carrier protein of acetyl-CoA carboxylase OS = *Escherichia coli* OX = 562 GN = ECs4127 PE = 4 SV = 1 | 0.786398139 | 0.000585555 | Down |
|  | D3QWK1 | *gmhB* | D,D-heptose 1,7-bisphosphate phosphatase OS = *Escherichia coli* O55 : H7 (strain CB9615 / EPEC) OX = 701177 GN = gmhB PE = 3 SV = 1 | 0.78575734 | 0.010351393 | Down |
|  | A0A5F1E363 | CQB02_04100 | tRNA/rRNA methyltransferase OS = *Escherichia coli* OX = 562 GN = CQB02_04100 PE = 4 SV = 1 | 0.785456138 | 0.009380156 | Down |
|  | U9Z5W5 | HMPREF1589_00327 | Uncharacterized protein OS = *Escherichia coli* 113290 OX = 1268976 GN = HMPREF1589_00327 PE = 4 SV = 1 | 0.785317757 | 0.031155701 | Down |
|  | Q8XEE3 | *nirB* | Nitrite reductase (NAD(P)H) subunit OS = *Escherichia coli* O157 : H7 OX = 83334 GN = nirB PE = 4 SV = 2 | 0.784930736 | 0.017240548 | Down |
|  | U9XL08 | HMPREF1589_04929 | Uncharacterized protein OS = *Escherichia coli* 113290 OX = 1268976 GN = HMPREF1589_04929 PE = 4 SV = 1 | 0.784644351 | 0.049982058 | Down |
|  | A0A7B4HWA4 | HMV41_29580 | Bifunctional tRNA (Adenosine(37)-C2)-methyltransferase TrmG/ribosomal RNA large subunit methyltransferase RlmN (Fragment) OS = *Escherichia coli* OX = 562 GN = HMV41_29580 PE = 4 SV = 1 | 0.784495783 | 0.0053578 | Down |
|  | A0A854RJR3 | *clpP* | ATP-dependent Clp endopeptidase, proteolytic subunit ClpP OS = *Escherichia coli* OX = 562 GN = clpP PE = 4 SV = 1 | 0.783340033 | 0.002989848 | Down |
|  | A0A827VIM6 | *yeiB* | DUF418 domain-containing protein OS = *Escherichia coli* OX = 562 GN = yeiB PE = 4 SV = 1 | 0.783225781 | 0.009328953 | Down |
|  | A0A1X3LY07 | EAZG_01691 | Putative oxidoreductase Fe-S subunit OS = *Escherichia coli* TA249 OX = 656441 GN = EAZG_01691 PE = 4 SV = 1 | 0.782246378 | 0.044846037 | Down |
|  | A0A7U0H0D9 | *topA* | DNA topoisomerase 1 OS = *Escherichia coli* OX = 562 GN = topA PE = 3 SV = 1 | 0.782228813 | 0.004828408 | Down |
|  | P76015 | *dhaK* | PEP-dependent dihydroxyacetone kinase, dihydroxyacetone-binding subunit DhaK OS = *Escherichia coli* (strain K12) OX = 83333 GN = dhaK PE = 1 SV = 2 | 0.781608908 | 0.003331555 | Down |
|  | V0YFL6 | *rpmC* | 50S ribosomal protein L29 OS = *Escherichia coli* 908525 OX = 1268995 GN = rpmC PE = 3 SV = 1 | 0.781192155 | 0.011464618 | Down |
|  | Q1R633 | *rpsM* | 30S ribosomal protein S13 OS = *Escherichia coli* (strain UTI89 / UPEC) OX = 364106 GN = rpsM PE = 3 SV = 1 | 0.780814614 | 0.006178014 | Down |
|  | F4TAV4 | *lon* | Lon protease OS = *Escherichia coli* M718 OX = 656419 GN = lon PE = 2 SV = 1 | 0.780734054 | 0.004943268 | Down |
|  | A0A6L7E791 | GRW81_27750 | IclR family transcriptional regulator (Fragment) OS = *Escherichia coli* OX = 562 GN = GRW81_27750 PE = 4 SV = 1 | 0.780370776 | 0.019181618 | Down |
|  | A0A5E8NUZ0 | ACN68_19100 | Peptidyl-prolyl cis-trans isomerase OS = *Escherichia coli* OX = 562 GN = ACN68_19100 PE = 3 SV = 1 | 0.779769484 | 0.002313568 | Down |
|  | A0A2X1MZY4 | *macB_2* | Macrolide export ATP-binding/permease protein OS = *Escherichia coli* OX = 562 GN = macB_2 PE = 4 SV = 1 | 0.779733158 | 0.006618631 | Down |
|  | A0A827CGX5 | *nemA* | N-ethylmaleimide reductase OS = *Escherichia coli* OX = 562 GN = nemA PE = 4 SV = 1 | 0.779715919 | 0.012316641 | Down |
|  | V0YJG2 | HMPREF1611_03527 | Isoaspartyl dipeptidase OS = *Escherichia coli* 908573 OX = 1268998 GN = HMPREF1611_03527 PE = 3 SV = 1 | 0.779214152 | 0.003555444 | Down |
|  | A0A6G6L238 | *yihT* | Sulfofructosephosphate aldolase OS = *Escherichia coli* OX = 562 GN = yihT PE = 3 SV = 1 | 0.77838787 | 0.00496084 | Down |
|  | D6IFY3 | ECDG_04365 | ATP synthase F0 OS = *Escherichia coli* B185 OX = 550676 GN = ECDG_04365 PE = 4 SV = 1 | 0.777449889 | 0.018282494 | Down |
|  | D0Z6U7 | unknow | Aminoglycoside 3'-phosphotransferase OS = *Escherichia coli* O78 : H11 (strain H10407 / ETEC) OX = 316401 PE = 3 SV = 1 | 0.777212248 | 0.012239111 | Down |
|  | A0A6C9XEF8 | GP906_06025 | YjjI family glycine radical enzyme OS = *Escherichia coli* OX = 562 GN = GP906_06025 PE = 4 SV = 1 | 0.777166342 | 0.018847587 | Down |
|  | A0A810UTR3 | *manX* | PTS system mannose-specific IIAB component OS = *Escherichia coli* OX = 562 GN = manX PE = 4 SV = 1 | 0.777110735 | 0.006356479 | Down |
|  | A0A5B9ARY7 | *rpsE* | 30S ribosomal protein S5 OS = *Escherichia coli* OX = 562 GN = rpsE PE = 3 SV = 1 | 0.776941661 | 0.008656859 | Down |
|  | E0WA13 | *gapA* | GapA (Fragment) OS = *Escherichia coli* OX = 562 GN = gapA PE = 4 SV = 1 | 0.776407811 | 0.01575093 | Down |
|  | A0A6D0FKH7 | *rffA* | dTDP-4-amino-4,6-dideoxygalactose transaminase OS = *Escherichia coli* OX = 562 GN = rffA PE = 3 SV = 1 | 0.776390999 | 0.002505343 | Down |
|  | P37760 | *rfbD* | dTDP-4-dehydrorhamnose reductase OS = *Escherichia coli* (strain K12) OX = 83333 GN = rfbD PE = 3 SV = 2 | 0.776354384 | 0.011099885 | Down |
|  | A0A2T1LEM2 | *rplN* | 50S ribosomal protein L14 OS = *Escherichia coli* OX = 562 GN = rplN PE = 3 SV = 1 | 0.775548002 | 0.03307817 | Down |
|  | P0AEH3 | *elaA* | Protein ElaA OS = *Escherichia coli* (strain K12) OX = 83333 GN = elaA PE = 1 SV = 1 | 0.775522021 | 0.002005943 | Down |
|  | E0WA12 | *gapA* | GapA (Fragment) OS = *Escherichia coli* OX = 562 GN = gapA PE = 4 SV = 1 | 0.775500974 | 0.018631733 | Down |
|  | B1XGK1 | *nadE* | NH(3)-dependent NAD(+) synthetase OS = *Escherichia coli* (strain K12 / DH10B) OX = 316385 GN = nadE PE = 3 SV = 1 | 0.775478948 | 0.003167403 | Down |
|  | A0A7B4HUY5 | HMV41_25160 | NCS2 family permease (Fragment) OS = *Escherichia coli* OX = 562 GN = HMV41_25160 PE = 4 SV = 1 | 0.775441679 | 0.027834626 | Down |
|  | A0A210BJJ3 | A8C65_07375 | Formate C-acetyltransferase OS = *Escherichia coli* OX = 562 GN = A8C65_07375 PE = 4 SV = 1 | 0.77537321 | 0.023437315 | Down |
|  | A0A0T5XRJ0 | *rplU* | 50S ribosomal protein L21 OS = *Escherichia coli* OX = 562 GN = rplU PE = 3 SV = 1 | 0.775257361 | 0.027754619 | Down |
|  | A0A775TLG3 | GRD59_23335 | RepB family plasmid replication initiator protein OS = *Escherichia coli* OX = 562 GN = GRD59_23335 PE = 4 SV = 1 | 0.775226145 | 0.032103949 | Down |
|  | E0WA03 | *gapA* | GapA (Fragment) OS = *Escherichia coli* OX = 562 GN = gapA PE = 4 SV = 1 | 0.774977911 | 0.002257642 | Down |
|  | A0A5F1DUB8 | *tdcC* | Threonine/serine transporter TdcC OS = *Escherichia coli* OX = 562 GN = tdcC PE = 3 SV = 1 | 0.774635152 | 0.008479922 | Down |
|  | B7UQ77 | *emtA* | Endo-type membrane-bound lytic murein transglycosylase A OS = *Escherichia coli* O127 : H6 (strain E2348/69 / EPEC) OX = 574521 GN = emtA PE = 3 SV = 1 | 0.774571846 | 0.00285049 | Down |
|  | D6JF44 | *rbfA* | Ribosome-binding factor A OS = *Escherichia coli* B354 OX = 550677 GN = rbfA PE = 3 SV = 1 | 0.774321689 | 0.00454897 | Down |
|  | A0A3L0W8F0 | *gpmI* | 2,3-bisphosphoglycerate-independent phosphoglycerate mutase OS = *Escherichia coli* OX = 562 GN = gpmI PE = 3 SV = 1 | 0.774246757 | 0.014972853 | Down |
|  | A0A0H3EJY2 | NRG857_12315 | Putative oxidoreductase Fe-S binding subunit OS = *Escherichia coli* O83 : H1 (strain NRG 857C / AIEC) OX = 685038 GN = NRG857_12315 PE = 4 SV = 1 | 0.773793839 | 0.043957471 | Down |
|  | I2SXA9 | *glk* | Glucokinase OS = *Escherichia coli* 1.2264 OX = 869675 GN = glk PE = 3 SV = 1 | 0.773678407 | 0.025708656 | Down |
|  | A0A6L4XIJ4 | GP710_11370 | Nitrate/nitrite transporter OS = *Escherichia coli* OX = 562 GN = GP710_11370 PE = 3 SV = 1 | 0.7735566 | 0.036539522 | Down |
|  | A0A0J8XKA8 | *metC* | Cystathionine beta-lyase OS = *Escherichia coli* OX = 562 GN = metC PE = 3 SV = 1 | 0.773471925 | 0.000348579 | Down |
|  | A0A376PPP7 | *tdcG* | L-serine dehydratase OS = *Escherichia coli* OX = 562 GN = tdcG PE = 3 SV = 1 | 0.772976628 | 0.007401758 | Down |
|  | A0A271QLV6 | *yaiI* | UPF0178 protein YaiI OS = *Escherichia coli* OX = 562 GN = yaiI PE = 3 SV = 1 | 0.772680304 | 0.035293268 | Down |
|  | A1Z1Z5 | *fllA55* | Flagellin (Fragment) OS = *Escherichia coli* OX = 562 GN = fllA55 PE = 3 SV = 1 | 0.772598966 | 0.021456545 | Down |
|  | P0ADZ5 | *rpsO* | 30S ribosomal protein S15 OS = *Escherichia coli* O6 : H1 (strain CFT073 / ATCC 700928 / UPEC) OX = 199310 GN = rpsO PE = 3 SV = 2 | 0.771464045 | 0.027671751 | Down |
|  | A0A192CHB0 | WLH_04039 | DnaK OS = *Escherichia coli* O25b : H4 OX = 941280 GN = WLH_04039 PE = 4 SV = 1 | 0.771238013 | 0.003566333 | Down |
|  | A0A7H9LUA1 | *ndh* | NADH-quinone dehydrogenase OS = *Escherichia coli* OX = 562 GN = ndh PE = 4 SV = 1 | 0.771128541 | 0.000176119 | Down |
|  | A0A8A5IM57 | *tdcE* | 2-ketobutyrate formate-lyase/pyruvate formate-lyase OS = *Escherichia coli* O176 : H45 OX = 2810408 GN = tdcE PE = 4 SV = 1 | 0.770010857 | 0.046001538 | Down |
|  | A0A5C9A7M4 | *dinD* | DNA damage-inducible protein D (Fragment) OS = *Escherichia coli* OX = 562 GN = dinD PE = 4 SV = 1 | 0.769211068 | 0.019625975 | Down |
|  | D8A1D9 | HMPREF9530_00303 | Toxin-antitoxin system, toxin component, Fic domain protein OS = *Escherichia coli* (strain MS 21-1) OX = 749527 GN = HMPREF9530_00303 PE = 4 SV = 1 | 0.769084025 | 0.00388067 | Down |
|  | A0A376MCS7 | *deoC* | Deoxyribose-phosphate aldolase OS = *Escherichia coli* OX = 562 GN = deoC PE = 3 SV = 1 | 0.768690236 | 0.018804367 | Down |
|  | A0A3Q0N3B6 | CR539_22890 | Chromosome segregation protein SMC OS = *Escherichia coli* OX = 562 GN = CR539_22890 PE = 4 SV = 1 | 0.767409314 | 0.021116964 | Down |
|  | Q8X712 | *gntT* | High-affinity transport of gluconate / gluconate permease OS = *Escherichia coli* O157 : H7 OX = 83334 GN = gntT PE = 4 SV = 2 | 0.767056658 | 0.003179894 | Down |
|  | A0A7A7AT69 | *rpsK* | 30S ribosomal protein S11 OS = *Escherichia coli* OX = 562 GN = rpsK PE = 3 SV = 1 | 0.766648657 | 0.014647516 | Down |
|  | A0A7U9APQ3 | ECKG_03384 | GTPase subunit of restriction endonuclease OS = *Escherichia coli* TA206 OX = 656440 GN = ECKG_03384 PE = 4 SV = 1 | 0.766566163 | 0.042059098 | Down |
|  | A0A828FPP9 | AAS29_000306 | Non-heme ferritin-like protein OS = *Escherichia coli* OX = 562 GN = AAS29_000306 PE = 4 SV = 1 | 0.766270718 | 0.00483678 | Down |
|  | A0A7H9QPU0 | *galK* | Galactokinase OS = *Escherichia coli* OX = 562 GN = galK PE = 3 SV = 1 | 0.766252114 | 0.01042502 | Down |
|  | A0A1X3HZY6 | EAMG_03073 | EAL domain containing protein involved in flagellar function OS = *Escherichia coli* M056 OX = 656415 GN = EAMG_03073 PE = 4 SV = 1 | 0.76587246 | 0.017052105 | Down |
|  | A0A826X348 | *grxD* | Monothiol glutaredoxin 4 OS = *Escherichia coli* OX = 562 GN = grxD PE = 4 SV = 1 | 0.765837876 | 0.026921911 | Down |
|  | A0A418GSC4 | D3C88_00795 | Aminotransferase class V-fold PLP-dependent enzyme (Fragment) OS = *Escherichia coli* OX = 562 GN = D3C88_00795 PE = 3 SV = 1 | 0.765242872 | 0.014855876 | Down |
|  | A0A6C9DPQ6 | GKF72_09365 | EF2563 family selenium-dependent molybdenum hydroxylase system protein OS = *Escherichia coli* OX = 562 GN = GKF72_09365 PE = 4 SV = 1 | 0.764850533 | 0.016830903 | Down |
|  | A0A786U474 | HIR31_002459 | Uncharacterized lipoprotein YifL OS = *Escherichia coli* OX = 562 GN = HIR31_002459 PE = 4 SV = 1 | 0.764807599 | 0.023109252 | Down |
|  | A0A2X1PTA3 | *rpmJ_1* | 50S ribosomal protein L36 OS = *Escherichia coli* OX = 562 GN = rpmJ_1 PE = 1 SV = 1 | 0.764618733 | 0.007857987 | Down |
|  | P07650 | *deoA* | Thymidine phosphorylase OS = *Escherichia coli* (strain K12) OX = 83333 GN = deoA PE = 1 SV = 3 | 0.764496091 | 0.001848531 | Down |
|  | A0A5D8SP90 | E0I42_21390 | Peptidyl-prolyl cis-trans isomerase OS = *Escherichia coli* OX = 562 GN = E0I42_21390 PE = 3 SV = 1 | 0.764203786 | 0.000161863 | Down |
|  | A0A8A8PXR3 | *aspA* | Aspartate ammonia-lyase OS = *Escherichia coli* OX = 562 GN = aspA PE = 4 SV = 1 | 0.764096868 | 0.046348946 | Down |
|  | A0A377CPF6 | *rhlB* | ATP-dependent RNA helicase RhlB OS = *Escherichia coli* OX = 562 GN = rhlB PE = 3 SV = 1 | 0.763815897 | 0.000194491 | Down |
|  | P02359 | *rpsG* | 30S ribosomal protein S7 OS = *Escherichia coli* (strain K12) OX = 83333 GN = rpsG PE = 1 SV = 3 | 0.763795386 | 0.008049001 | Down |
|  | A0A6M0PR26 | *ribD* | Riboflavin biosynthesis protein RibD OS = *Escherichia coli* OX = 562 GN = ribD PE = 3 SV = 1 | 0.763727106 | 0.002859941 | Down |
|  | B7UHJ6 | *pyrG* | CTP synthase OS = *Escherichia coli* O127 : H6 (strain E2348/69 / EPEC) OX = 574521 GN = pyrG PE = 3 SV = 1 | 0.763343081 | 0.000321313 | Down |
|  | A0A7B2TEE0 | *rpmE* | 50S ribosomal protein L31 OS = *Escherichia coli* OX = 562 GN = rpmE PE = 3 SV = 1 | 0.763322375 | 0.038498749 | Down |
|  | A0A7A6MWE8 | *rplD* | 50S ribosomal protein L4 OS = *Escherichia coli* OX = 562 GN = rplD PE = 3 SV = 1 | 0.763276974 | 0.014661526 | Down |
|  | A0A2H4TNY6 | *nagB* | Glucosamine-6-phosphate deaminase OS = *Escherichia coli* OX = 562 GN = nagB PE = 3 SV = 1 | 0.762092924 | 0.006498857 | Down |
|  | A0A2H3MBD6 | CO706_08145 | Galactitol-1-phosphate 5-dehydrogenase OS = *Escherichia coli* OX = 562 GN = CO706_08145 PE = 3 SV = 1 | 0.761935521 | 0.008328115 | Down |
|  | A0A8A5HAP0 | *rbsB* | Ribose ABC transporter substrate-binding protein RbsB OS = *Escherichia coli* O89m : H10 OX = 2810405 GN = rbsB PE = 4 SV = 1 | 0.76144632 | 0.027744963 | Down |
|  | A0A828B9X1 | *nudK* | GDP-mannose pyrophosphatase NudK OS = *Escherichia coli* OX = 562 GN = nudK PE = 4 SV = 1 | 0.761189285 | 0.005010437 | Down |
|  | A0A7U2Z0M6 | *rpmA* | 50S ribosomal protein L27 OS = *Escherichia coli* OX = 562 GN = rpmA PE = 3 SV = 1 | 0.760913881 | 0.010480093 | Down |
|  | A0A2X1JEL2 | *rplX* | 50S ribosomal protein L24 OS = *Escherichia coli* OX = 562 GN = rplX PE = 3 SV = 1 | 0.760288232 | 0.00744494 | Down |
|  | A0A0K4GWR9 | *speA* | Biosynthetic arginine decarboxylase OS = *Escherichia coli* OX = 562 GN = speA PE = 3 SV = 1 | 0.760232772 | 0.001172308 | Down |
|  | P76046 | *ycjX* | Uncharacterized protein YcjX OS = *Escherichia coli* (strain K12) OX = 83333 GN = ycjX PE = 1 SV = 1 | 0.760091964 | 0.001640012 | Down |
|  | A0A2Y0WCD5 | *ubiT* | Ubiquinone biosynthesis accessory factor UbiT OS = *Escherichia coli* OX = 562 GN = ubiT PE = 3 SV = 1 | 0.759116842 | 0.010229842 | Down |
|  | A0A774NC69 | GFY48_24340 | S-formylglutathione hydrolase OS = *Escherichia coli* OX = 562 GN = GFY48_24340 PE = 3 SV = 1 | 0.757732826 | 0.008962353 | Down |
|  | A0A3W4D6A0 | *ispF* | 2-C-methyl-D-erythritol 2,4-cyclodiphosphate synthase OS = *Escherichia coli* OX = 562 GN = ispF PE = 3 SV = 1 | 0.757709466 | 0.014609525 | Down |
|  | A0A7H9LVW9 | *ssnA* | Putative aminohydrolase SsnA OS = *Escherichia coli* OX = 562 GN = ssnA PE = 4 SV = 1 | 0.756326398 | 0.009289309 | Down |
|  | A0A5N8HMR5 | *guaA* | GMP synthase (glutamine-hydrolyzing) (Fragment) OS = *Escherichia coli* OX = 562 GN = guaA PE = 4 SV = 1 | 0.755347719 | 0.002186195 | Down |
|  | A0A7H9QPB1 | *pspE* | Thiosulfate sulfurtransferase PspE OS = *Escherichia coli* OX = 562 GN = pspE PE = 4 SV = 1 | 0.754852386 | 0.020333491 | Down |
|  | B1LDE8 | *speA* | Biosynthetic arginine decarboxylase OS = *Escherichia coli* (strain SMS-3-5 / SECEC) OX = 439855 GN = speA PE = 3 SV = 1 | 0.754641261 | 0.000151027 | Down |
|  | A0A829JPY3 | H000_05313 | Transposase OS = *Escherichia coli* UMEA 3899-1 OX = 1281275 GN = H000_05313 PE = 4 SV = 1 | 0.754505234 | 0.001943803 | Down |
|  | P02942 | *tsr* | Methyl-accepting chemotaxis protein I OS = *Escherichia coli* (strain K12) OX = 83333 GN = tsr PE = 1 SV = 2 | 0.754503541 | 0.012346497 | Down |
|  | A0A827G2Z0 | *alsR* | Als operon DNA-binding transcriptional repressor AlsR OS = *Escherichia coli* OX = 562 GN = alsR PE = 4 SV = 1 | 0.753820354 | 0.013070035 | Down |
|  | A0A0K4GBA4 | *glpE* | Thiosulfate sulfurtransferase GlpE OS = *Escherichia coli* OX = 562 GN = glpE PE = 3 SV = 1 | 0.753405178 | 0.013873463 | Down |
|  | A0A831DZJ5 | HMV95_06490 | Peptidylprolyl isomerase OS = *Escherichia coli* OX = 562 GN = HMV95_06490 PE = 4 SV = 1 | 0.753050512 | 0.018813342 | Down |
|  | A0A828PCN4 | A5U30_005570 | Sialate O-acetylesterase OS = *Escherichia coli* OX = 562 GN = A5U30_005570 PE = 4 SV = 1 | 0.752877626 | 0.009730575 | Down |
|  | A0A3L3AP41 | *lpxT* | Lipid A 1-diphosphate synthase OS = *Escherichia coli* OX = 562 GN = lpxT PE = 3 SV = 1 | 0.752725832 | 0.003121172 | Down |
|  | A0A376U3J7 | *yjiY* | Putative carbon starvation protein OS = *Escherichia coli* OX = 562 GN = yjiY PE = 3 SV = 1 | 0.752596161 | 0.04771505 | Down |
|  | A0A376RDW4 | *arcB_4* | Aerobic respiration control protein (Bifunctional two-component sensor kinase/response regulator) OS = *Escherichia coli* OX = 562 GN = arcB_4 PE = 4 SV = 1 | 0.751229788 | 0.013580699 | Down |
|  | Q8XEF0 | *gltB* | Glutamate synthase, large subunit OS = *Escherichia coli* O157 : H7 OX = 83334 GN = gltB PE = 3 SV = 2 | 0.751012745 | 0.022331746 | Down |
|  | A0A3T9CJN2 | *nusA* | Transcription termination/antitermination protein NusA OS = *Escherichia coli* OX = 562 GN = nusA PE = 3 SV = 1 | 0.750686799 | 0.001823292 | Down |
|  | A0A823ADS0 | DM968_00965 | Tryptophan permease OS = *Escherichia coli* OX = 562 GN = DM968_00965 PE = 4 SV = 1 | 0.750603122 | 0.038137605 | Down |
|  | A0A826URB7 | *exbB* | Tol-pal system-associated acyl-CoA thioesterase OS = *Escherichia coli* OX = 562 GN = exbB PE = 4 SV = 1 | 0.750541523 | 0.003670102 | Down |
|  | A0A2X3LQH0 | *rplR* | 50S ribosomal protein L18 OS = *Escherichia coli* OX = 562 GN = rplR PE = 3 SV = 1 | 0.750400347 | 0.0320032 | Down |
|  | A0A7U8ZI69 | ECGG_03202 | Iron(III) dicitrate transport system OS = *Escherichia coli* FVEC1412 OX = 656380 GN = ECGG_03202 PE = 4 SV = 1 | 0.750154958 | 0.029362143 | Down |
|  | A0A831B9J4 | HJS37_002968 | TIGR01620 family protein OS = *Escherichia coli* OX = 562 GN = HJS37_002968 PE = 4 SV = 1 | 0.749931662 | 0.000227381 | Down |
|  | A0A787CMN5 | *rpsF* | 30S ribosomal protein S6 OS = *Escherichia coli* OX = 562 GN = rpsF PE = 3 SV = 1 | 0.749687443 | 0.012771666 | Down |
|  | A0A6N8Q549 | GRW77_32280 | Tagatose-bisphosphate aldolase subunit GatZ (Fragment) OS = *Escherichia coli* OX = 562 GN = GRW77_32280 PE = 4 SV = 1 | 0.749590606 | 0.003521535 | Down |
|  | A0A376S092 | *hcrA_2* | Selenate reductase subunit YgfN OS = *Escherichia coli* OX = 562 GN = hcrA_2 PE = 4 SV = 1 | 0.749294148 | 0.040158487 | Down |
|  | A0A6D0EN39 | GQM09_30140 | Aspartate--tRNA ligase (Fragment) OS = *Escherichia coli* OX = 562 GN = GQM09_30140 PE = 4 SV = 1 | 0.749166464 | 0.022860399 | Down |
|  | A0A826TRD1 | *efp* | Elongation factor P OS = *Escherichia coli* OX = 562 GN = efp PE = 4 SV = 1 | 0.748485066 | 0.007010478 | Down |
|  | B1LR79 | *rplI* | 50S ribosomal protein L9 OS = *Escherichia coli* (strain SMS-3-5 / SECEC) OX = 439855 GN = rplI PE = 3 SV = 1 | 0.748067283 | 0.034750758 | Down |
|  | A9X4P5 | *gyrB* | DNA topoisomerase (ATP-hydrolyzing) (Fragment) OS = *Escherichia coli* OX = 562 GN = gyrB PE = 3 SV = 1 | 0.747747108 | 0.007632956 | Down |
|  | A0A827G2C2 | *malP* | Maltodextrin phosphorylase OS = *Escherichia coli* OX = 562 GN = malP PE = 4 SV = 1 | 0.74714121 | 0.014252149 | Down |
|  | A0A828B2R6 | A8W81_004159 | Altronate dehydratase OS = *Escherichia coli* OX = 562 GN = A8W81_004159 PE = 4 SV = 1 | 0.746425745 | 0.005425148 | Down |
|  | A0A0J1XV59 | *infA* | Translation initiation factor IF-1 OS = *Escherichia coli* OX = 562 GN = infA PE = 3 SV = 1 | 0.74642357 | 0.037045878 | Down |
|  | P69229 | *baeR* | Transcriptional regulatory protein BaeR OS = *Escherichia coli* O6 : H1 (strain CFT073 / ATCC 700928 / UPEC) OX = 199310 GN = baeR PE = 1 SV = 1 | 0.745801863 | 0.001702445 | Down |
|  | A0A376U932 | *tyrS_3* | Tyrosyl-tRNA synthetase OS = *Escherichia coli* OX = 562 GN = tyrS_3 PE = 3 SV = 1 | 0.745358778 | 0.014861201 | Down |
|  | A0A0K3TCZ7 | *adiA* | Biodegradative arginine decarboxylase OS = *Escherichia coli* OX = 562 GN = adiA PE = 3 SV = 1 | 0.745211754 | 0.000877376 | Down |
|  | A0A1V2GF16 | BXT93_12685 | Iron-sulfur cluster assembly scaffold protein IscU (Fragment) OS = *Escherichia coli* OX = 562 GN = BXT93_12685 PE = 3 SV = 1 | 0.74502993 | 0.001212634 | Down |
|  | A0A6D0ELG5 | GQM09_27360 | Branched chain amino acid aminotransferase (Fragment) OS = *Escherichia coli* OX = 562 GN = GQM09_27360 PE = 3 SV = 1 | 0.744660951 | 0.002345289 | Down |
|  | A0A6N6Y316 | *cheW* | Chemotaxis protein CheW OS = *Escherichia coli* OX = 562 GN = cheW PE = 4 SV = 1 | 0.744482337 | 0.031250993 | Down |
|  | A0A5D8RK51 | E0I42_27350 | Restriction endonuclease subunit R (Fragment) OS = *Escherichia coli* OX = 562 GN = E0I42_27350 PE = 4 SV = 1 | 0.744187894 | 0.020890114 | Down |
|  | Q8XE60 | *lon* | Lon protease OS = *Escherichia coli* O157 : H7 OX = 83334 GN = lon PE = 2 SV = 2 | 0.743828019 | 0.001025844 | Down |
|  | E1IV54 | *dsbD* | Thiol : disulfide interchange protein DsbD OS = *Escherichia coli* MS 145-7 OX = 679204 GN = dsbD PE = 3 SV = 1 | 0.743365385 | 0.020870361 | Down |
|  | A0A2X3M923 | *pepE* | Peptidase E OS = *Escherichia coli* OX = 562 GN = pepE PE = 3 SV = 1 | 0.74263169 | 0.00894559 | Down |
|  | A0A4Z0T8U9 | DAH37_27480 | Protein flxA OS = *Escherichia coli* OX = 562 GN = DAH37_27480 PE = 4 SV = 1 | 0.742046766 | 0.032167634 | Down |
|  | A0A210BXS2 | *yncD* | Iron outer membrane transporter OS = *Escherichia coli* OX = 562 GN = yncD PE = 3 SV = 1 | 0.741662822 | 0.013234398 | Down |
|  | A0A837MF64 | SM09_00856 | Pyruvate dehydrogenase E1 component OS = *Escherichia coli* OX = 562 GN = SM09_00856 PE = 4 SV = 1 | 0.741562508 | 0.00018502 | Down |
|  | P64476 | *ydiH* | Uncharacterized protein YdiH OS = *Escherichia coli* (strain K12) OX = 83333 GN = ydiH PE = 4 SV = 2 | 0.741461846 | 0.00107241 | Down |
|  | P0ACM9 | *yihL* | Uncharacterized HTH-type transcriptional regulator YihL OS = *Escherichia coli* (strain K12) OX = 83333 GN = yihL PE = 4 SV = 1 | 0.740737803 | 0.007331407 | Down |
|  | A0A444R4K3 | EPS76_27405 | Site-specific DNA recombinase OS = *Escherichia coli* OX = 562 GN = EPS76_27405 PE = 3 SV = 1 | 0.740368321 | 0.007941736 | Down |
|  | A0A377A2N6 | *prc_5* | Tail-specific protease OS = *Escherichia coli* OX = 562 GN = prc_5 PE = 4 SV = 1 | 0.740298969 | 0.00313916 | Down |
|  | A0A826X6M0 | *dcrB* | DcrB family lipoprotein OS = *Escherichia coli* OX = 562 GN = dcrB PE = 4 SV = 1 | 0.739839008 | 0.000102443 | Down |
|  | A0A5D8QGS0 | E0I42_29450 | Uncharacterized protein (Fragment) OS = *Escherichia coli* OX = 562 GN = E0I42_29450 PE = 4 SV = 1 | 0.738980374 | 0.005062883 | Down |
|  | B7UFB0 | *gatB* | Galactitol-specific enzyme IIB component of PTS OS = *Escherichia coli* O127 : H6 (strain E2348/69 / EPEC) OX = 574521 GN = gatB PE = 4 SV = 1 | 0.738957819 | 0.040688652 | Down |
|  | A0A6G2G671 | *scpB* | Methylmalonyl-CoA decarboxylase OS = *Escherichia coli* OX = 562 GN = scpB PE = 3 SV = 1 | 0.738821321 | 0.046089356 | Down |
|  | A0A1X9TR86 | *rihA* | Pyrimidine-specific ribonucleoside hydrolase RihA OS = *Escherichia coli* OX = 562 GN = rihA PE = 3 SV = 1 | 0.737666054 | 0.002516567 | Down |
|  | A0A2T1LHJ7 | *iscA* | Iron-binding protein IscA OS = *Escherichia coli* OX = 562 GN = iscA PE = 3 SV = 1 | 0.737367288 | 3.12068E-05 | Down |
|  | A0A826NJ86 | *infB* | Translation initiation factor IF-2 OS = *Escherichia coli* O157 OX = 1045010 GN = infB PE = 4 SV = 1 | 0.737121441 | 0.00134748 | Down |
|  | A0A829DLS5 | EC2875150_4547 | Binding--dependent transport system inner membrane component family protein OS = *Escherichia coli* 2875150 OX = 1116036 GN = EC2875150_4547 PE = 4 SV = 1 | 0.73605833 | 0.022554716 | Down |
|  | A0A2T1LPG8 | C6985_01380 | Cytosol nonspecific dipeptidase OS = *Escherichia coli* OX = 562 GN = C6985_01380 PE = 4 SV = 1 | 0.73548267 | 0.018348579 | Down |
|  | A0A7H5D8I1 | *lpp* | Major outer membrane lipoprotein Lpp OS = *Escherichia coli* OX = 562 GN = lpp PE = 3 SV = 1 | 0.734532835 | 0.028311176 | Down |
|  | Q47308 | *aph* | Aminoglycoside 3'-phosphotransferase OS = *Escherichia coli* OX = 562 GN = aph PE = 3 SV = 1 | 0.733617292 | 0.002755941 | Down |
|  | A0A0K5M4M1 | *pmbA* | Metalloprotease PmbA OS = *Escherichia coli* OX = 562 GN = pmbA PE = 3 SV = 1 | 0.731380718 | 0.01467664 | Down |
|  | A0A826RU97 | FPS82_07065 | Aldo/keto reductase family oxidoreductase OS = *Escherichia coli* OX = 562 GN = FPS82_07065 PE = 4 SV = 1 | 0.730569876 | 0.000899593 | Down |
|  | A0A2X1PFM4 | *ygiB* | UPF0441 protein YgiB OS = *Escherichia coli* OX = 562 GN = ygiB PE = 3 SV = 1 | 0.730463676 | 0.001441699 | Down |
|  | Q8XAW8 | *rbsD* | D-ribose pyranase OS = *Escherichia coli* O157 : H7 OX = 83334 GN = rbsD PE = 3 SV = 2 | 0.729348047 | 0.007894182 | Down |
|  | A0A660HCD7 | A9X72_10295 | Ferritin OS = *Escherichia coli* OX = 562 GN = A9X72_10295 PE = 3 SV = 1 | 0.729081312 | 0.000247848 | Down |
|  | P29744 | *flgL* | Flagellar hook-associated protein 3 OS = *Escherichia coli* (strain K12) OX = 83333 GN = flgL PE = 3 SV = 2 | 0.728462446 | 0.005455216 | Down |
|  | A0A7A2WWV5 | *hycB* | 4Fe-4S dicluster domain-containing protein OS = *Escherichia coli* OX = 562 GN = hycB PE = 4 SV = 1 | 0.727728361 | 0.049144231 | Down |
|  | A0A827JUP0 | *gcvP* | Aminomethyl-transferring glycine dehydrogenase OS = *Escherichia coli* OX = 562 GN = gcvP PE = 4 SV = 1 | 0.727713641 | 0.008961531 | Down |
|  | A0A827VKR7 | F2N31_10265 | Isopentenyl-diphosphate delta-isomerase OS = *Escherichia coli* OX = 562 GN = F2N31_10265 PE = 4 SV = 1 | 0.72726357 | 0.000424894 | Down |
|  | W1ETG5 | unknow | Uncharacterized protein OS = *Escherichia coli* ISC7 OX = 1432555 PE = 4 SV = 1 | 0.727234694 | 0.017208991 | Down |
|  | A0A0A0H5V9 | *fliP* | Flagellar biosynthetic protein FliP OS = *Escherichia coli* OX = 562 GN = fliP PE = 3 SV = 1 | 0.727169746 | 0.000397291 | Down |
|  | A0A377K5F9 | *flhE* | Flagellar protein FlhE OS = *Escherichia coli* OX = 562 GN = flhE PE = 4 SV = 1 | 0.724608227 | 0.004608697 | Down |
|  | P75726 | *citF* | Citrate lyase alpha chain OS = *Escherichia coli* (strain K12) OX = 83333 GN = citF PE = 2 SV = 1 | 0.72458468 | 0.016437511 | Down |
|  | A0A377CXM6 | *folK_1* | 2-amino-4-hydroxy-6-hydroxymethyldihydropteridine pyrophosphokinase OS = *Escherichia coli* OX = 562 GN = folK_1 PE = 3 SV = 1 | 0.724425391 | 0.002543069 | Down |
|  | A0A8B5MA57 | *gatZ* | Tagatose-bisphosphate aldolase subunit GatZ OS = *Escherichia coli* OX = 562 GN = gatZ PE = 4 SV = 1 | 0.724222589 | 0.024811421 | Down |
|  | E6BD97 | *galE* | UDP-glucose 4-epimerase OS = *Escherichia coli* MS 85-1 OX = 679202 GN = galE PE = 3 SV = 1 | 0.724162346 | 0.009177412 | Down |
|  | Q8XDE8 | *pncB* | Nicotinate phosphoribosyltransferase OS = *Escherichia coli* O157 : H7 OX = 83334 GN = pncB PE = 3 SV = 3 | 0.724135909 | 0.011279008 | Down |
|  | A0A7L5VD19 | *pgk* | Phosphoglycerate kinase OS = *Escherichia coli* OX = 562 GN = pgk PE = 3 SV = 1 | 0.723656949 | 0.022487723 | Down |
|  | A0A2X1K179 | *vacJ_2* | Lipoprotein OS = *Escherichia coli* OX = 562 GN = vacJ_2 PE = 4 SV = 1 | 0.723592449 | 0.007465116 | Down |
|  | A0A5F1T4G6 | DAH34_13570 | Superoxide dismutase OS = *Escherichia coli* OX = 562 GN = DAH34_13570 PE = 3 SV = 1 | 0.723491573 | 0.043664884 | Down |
|  | A0A029HTA9 | *rcsB* | Capsular synthesis regulator component B OS = *Escherichia coli* 2-005-03_S4_C3 OX = 1444258 GN = rcsB PE = 4 SV = 1 | 0.723249357 | 0.000322937 | Down |
|  | A0A3L0WY87 | *infB* | Translation initiation factor IF-2 OS = *Escherichia coli* OX = 562 GN = infB PE = 3 SV = 1 | 0.722612469 | 0.031513181 | Down |
|  | A0A831BLF9 | *fabZ* | 3-hydroxyacyl-ACP dehydratase FabZ OS = *Escherichia coli* OX = 562 GN = fabZ PE = 4 SV = 1 | 0.722456722 | 0.021033756 | Down |
|  | A0A6A0Q765 | *malK* | Maltose/maltodextrin ABC transporter ATP-binding protein MalK OS = *Escherichia coli* OX = 562 GN = malK PE = 4 SV = 1 | 0.720133158 | 0.016301771 | Down |
|  | A0A827PK17 | *fecA* | Fe(3+) dicitrate transport protein FecA OS = *Escherichia coli* OX = 562 GN = fecA PE = 4 SV = 1 | 0.720037135 | 0.014657534 | Down |
|  | A0A024L1I7 | *cheB* | Protein-glutamate methylesterase/protein-glutamine glutaminase OS = *Escherichia coli* OX = 562 GN = cheB PE = 3 SV = 1 | 0.719569665 | 0.003915068 | Down |
|  | A0A768EGW4 | HMT45_14985 | PTS galactitol transporter subunit IIA OS = *Escherichia coli* OX = 562 GN = HMT45_14985 PE = 4 SV = 1 | 0.719041589 | 0.001939082 | Down |
|  | A0A7U3BCZ4 | *flgF* | Flagellar basal body protein OS = *Escherichia coli* OX = 562 GN = flgF PE = 3 SV = 1 | 0.718756388 | 0.021186635 | Down |
|  | A0A6N4KV03 | BON98_05250 | EIICB-Glc OS = *Escherichia coli* OX = 562 GN = BON98_05250 PE = 4 SV = 1 | 0.718153167 | 0.005153882 | Down |
|  | A0A826Q325 | *citE* | Citrate (Pro-3S)-lyase subunit beta OS = *Escherichia coli* OX = 562 GN = citE PE = 4 SV = 1 | 0.717817911 | 0.004989439 | Down |
|  | A0A478KX94 | BvCmsH19A_01829 | DNA injection protein OS = *Escherichia coli* OX = 562 GN = BvCmsH19A_01829 PE = 4 SV = 1 | 0.717761717 | 0.001355667 | Down |
|  | A0A1U9SZS3 | *ribH* | 6,7-dimethyl-8-ribityllumazine synthase OS = *Escherichia coli* OX = 562 GN = ribH PE = 3 SV = 1 | 0.717252076 | 0.026630779 | Down |
|  | A0A770C125 | *malK* | Maltose/maltodextrin import ATP-binding protein MalK OS = *Escherichia coli* OX = 562 GN = malK PE = 4 SV = 1 | 0.717229841 | 0.0051406 | Down |
|  | A0A2T1LFH3 | *parC* | DNA topoisomerase 4 subunit A OS = *Escherichia coli* OX = 562 GN = parC PE = 3 SV = 1 | 0.716729865 | 1.74663E-05 | Down |
|  | P0AFW6 | *rnk* | Regulator of nucleoside diphosphate kinase OS = *Escherichia coli* O157 : H7 OX = 83334 GN = rnk PE = 3 SV = 1 | 0.716683602 | 0.0043308 | Down |
|  | A0A1M0D457 | *ycbX* | 6-N-hydroxylaminopurine resistance protein YcbX OS = *Escherichia coli* OX = 562 GN = ycbX PE = 4 SV = 1 | 0.71608418 | 0.001183197 | Down |
|  | A0A403LLF2 | *nikR* | Nickel-responsive regulator OS = *Escherichia coli* OX = 562 GN = nikR PE = 3 SV = 1 | 0.715878547 | 0.010467157 | Down |
|  | P0AE68 | *cheY* | Chemotaxis protein CheY OS = *Escherichia coli* O157 : H7 OX = 83334 GN = cheY PE = 3 SV = 2 | 0.715257793 | 0.01484459 | Down |
|  | A0A0L1C331 | *aphA* | Class B acid phosphatase OS = *Escherichia coli* OX = 562 GN = aphA PE = 3 SV = 1 | 0.713890078 | 0.002714561 | Down |
|  | A0A7L5L2P8 | *rpsP* | 30S ribosomal protein S16 OS = *Escherichia coli* OX = 562 GN = rpsP PE = 3 SV = 1 | 0.713341392 | 0.000676208 | Down |
|  | A0A4P0YY48 | *pepD_1* | Aminoacyl-histidine dipeptidase OS = *Escherichia coli* OX = 562 GN = pepD_1 PE = 4 SV = 1 | 0.713067995 | 0.001115344 | Down |
|  | P0A9Q8 | *adhE* | Aldehyde-alcohol dehydrogenase OS = *Escherichia coli* O157 : H7 OX = 83334 GN = adhE PE = 3 SV = 2 | 0.712626046 | 0.009020241 | Down |
|  | A0A827ABQ5 | CN875_002057 | NADP(H)-dependent aldo-keto reductase OS = *Escherichia coli* OX = 562 GN = CN875_002057 PE = 4 SV = 1 | 0.712387038 | 0.009691076 | Down |
|  | D3GY01 | EC042_4857 | Putative glycoprotein/receptor OS = *Escherichia coli* O44 : H18 (strain 042 / EAEC) OX = 216592 GN = EC042_4857 PE = 4 SV = 1 | 0.712142775 | 0.001874998 | Down |
|  | Q8X5W6 | *malM* | Periplasmic protein of mal regulon OS = *Escherichia coli* O157 : H7 OX = 83334 GN = malM PE = 4 SV = 2 | 0.711813295 | 0.022944475 | Down |
|  | P39384 | *yjiM* | Putative dehydratase subunit YjiM OS = *Escherichia coli* (strain K12) OX = 83333 GN = yjiM PE = 3 SV = 2 | 0.711796803 | 0.001274566 | Down |
|  | A0A2T1LE54 | *rpsL* | 30S ribosomal protein S12 OS = *Escherichia coli* OX = 562 GN = rpsL PE = 3 SV = 1 | 0.711595923 | 0.016183759 | Down |
|  | A0A799XZ20 | HL593_24860 | IS66-like element ISEc23 family transposase (Fragment) OS = *Escherichia coli* OX = 562 GN = HL593_24860 PE = 4 SV = 1 | 0.711385992 | 0.043884213 | Down |
|  | A0A8B5MAI7 | *yeaE* | Methylglyoxal reductase YeaE OS = *Escherichia coli* OX = 562 GN = yeaE PE = 4 SV = 1 | 0.710733163 | 0.000270629 | Down |
|  | A0A2X1N3J8 | *aspA* | Aspartate ammonia-lyase OS = *Escherichia coli* OX = 562 GN = aspA PE = 3 SV = 1 | 0.710350046 | 0.012471733 | Down |
|  | A0A376I0H2 | *pta* | Phosphate acetyltransferase OS = *Escherichia coli* OX = 562 GN = pta PE = 3 SV = 1 | 0.709827756 | 0.000587232 | Down |
|  | D3QQ79 | *rne* | Ribonuclease E OS = *Escherichia coli* O55 : H7 (strain CB9615 / EPEC) OX = 701177 GN = rne PE = 3 SV = 1 | 0.709622439 | 0.018068687 | Down |
|  | A0A5D8MJ02 | E0I42_15750 | PTS glucitol/sorbitol transporter subunit IIA OS = *Escherichia coli* OX = 562 GN = E0I42_15750 PE = 4 SV = 1 | 0.709026563 | 0.007886561 | Down |
|  | P24203 | *yjiA* | P-loop guanosine triphosphatase YjiA OS = *Escherichia coli* (strain K12) OX = 83333 GN = yjiA PE = 1 SV = 3 | 0.708721276 | 0.011183496 | Down |
|  | A0A7I7HZN9 | *citD* | Citrate lyase acyl carrier protein OS = *Escherichia coli* OX = 562 GN = citD PE = 4 SV = 1 | 0.708663085 | 0.029504241 | Down |
|  | W1X0Z6 | Q609_ECAC01641G0005 | PTS system maltose-and glucose-specific EIICB component OS = *Escherichia coli* DORA_A_5_14_21 OX = 1403943 GN = Q609_ECAC01641G0005 PE = 4 SV = 1 | 0.708245916 | 0.022739134 | Down |
|  | A0A2H9EUJ5 | *ndk* | Nucleoside diphosphate kinase OS = *Escherichia coli* OX = 562 GN = ndk PE = 3 SV = 1 | 0.707923636 | 0.006561067 | Down |
|  | A0A810TKM9 | *chbF* | 6-phospho-beta-glucosidase OS = *Escherichia coli* OX = 562 GN = chbF PE = 4 SV = 1 | 0.707835681 | 0.036957668 | Down |
|  | A0A829DGU5 | EC2875150_2933 | Haloacid dehalogenase-like hydrolase family protein OS = *Escherichia coli* 2875150 OX = 1116036 GN = EC2875150_2933 PE = 4 SV = 1 | 0.707565725 | 0.00200932 | Down |
|  | A0A8B3HX17 | EIA13_06520 | Maltoporin OS = *Escherichia coli* OX = 562 GN = EIA13_06520 PE = 4 SV = 1 | 0.707436102 | 0.030005287 | Down |
|  | A0A0H2V4K9 | *pepD* | Aminoacyl-histidine dipeptidase OS = *Escherichia coli* O6 : H1 (strain CFT073 / ATCC 700928 / UPEC) OX = 199310 GN = pepD PE = 4 SV = 1 | 0.706911511 | 0.007969269 | Down |
|  | A0A7H9QNW5 | *pepN* | Aminopeptidase N OS = *Escherichia coli* OX = 562 GN = pepN PE = 3 SV = 1 | 0.705682357 | 0.000677548 | Down |
|  | A0A234XVQ4 | D4V09_11885 | Class B acid phosphatase OS = *Escherichia coli* OX = 562 GN = D4V09_11885 PE = 3 SV = 1 | 0.705335252 | 0.023436927 | Down |
|  | A0A2T1LKD4 | C6985_09835 | Aldehyde ferredoxin oxidoreductase OS = *Escherichia coli* OX = 562 GN = C6985_09835 PE = 3 SV = 1 | 0.705152836 | 0.003294426 | Down |
|  | A0A789MBR7 | HIO80_003831 | YjjI family glycine radical enzyme OS = *Escherichia coli* OX = 562 GN = HIO80_003831 PE = 4 SV = 1 | 0.705087809 | 0.000365627 | Down |
|  | P0AB72 | *fbaA* | Fructose-bisphosphate aldolase class 2 OS = *Escherichia coli* O157 : H7 OX = 83334 GN = fbaA PE = 3 SV = 2 | 0.704413679 | 0.005334087 | Down |
|  | A0A826YZR0 | FEL34_22920 | HNH endonuclease OS = *Escherichia coli* OX = 562 GN = FEL34_22920 PE = 4 SV = 1 | 0.702897913 | 0.010613811 | Down |
|  | A0A831FLZ4 | *lon* | Endopeptidase La OS = *Escherichia coli* OX = 562 GN = lon PE = 4 SV = 1 | 0.702216268 | 0.012535192 | Down |
|  | A0A2W6PV85 | DNQ45_27140 | Nucleoside-specific channel-forming protein Tsx OS = *Escherichia coli* OX = 562 GN = DNQ45_27140 PE = 3 SV = 1 | 0.701865388 | 0.007763974 | Down |
|  | A0A6D0JE36 | GP946_28495 | ATP-binding cassette domain-containing protein (Fragment) OS = *Escherichia coli* OX = 562 GN = GP946_28495 PE = 4 SV = 1 | 0.701786711 | 0.013199507 | Down |
|  | A0A7A9N155 | *hydA* | D-phenylhydantoinase OS = *Escherichia coli* OX = 562 GN = hydA PE = 3 SV = 1 | 0.701670521 | 0.044416825 | Down |
|  | A0A7I9ASU8 | ETECE925_04375 | Dynamin-type G domain-containing protein OS = *Escherichia coli* OX = 562 GN = ETECE925_04375 PE = 4 SV = 1 | 0.70165833 | 0.00365883 | Down |
|  | Q6JZC5 | *trpB* | Tryptophan synthase beta chain (Fragment) OS = *Escherichia coli* OX = 562 GN = trpB PE = 3 SV = 1 | 0.701314911 | 0.000161457 | Down |
|  | A0A5F1DL70 | *hslU* | ATP-dependent protease ATPase subunit HslU OS = *Escherichia coli* OX = 562 GN = hslU PE = 2 SV = 1 | 0.700721634 | 0.001014873 | Down |
|  | A0A7U8X814 | ECFG_02822 | Aminopeptidase N OS = *Escherichia coli* FVEC1302 OX = 656379 GN = ECFG_02822 PE = 3 SV = 1 | 0.699713578 | 0.006127861 | Down |
|  | A0A2T1LHC8 | C6985_14235 | Phosphoenolpyruvate-protein phosphotransferase OS = *Escherichia coli* OX = 562 GN = C6985_14235 PE = 3 SV = 1 | 0.699300687 | 0.021143126 | Down |
|  | A0A826X2W6 | CSE52_002661 | Uncharacterized protein OS = *Escherichia coli* OX = 562 GN = CSE52_002661 PE = 4 SV = 1 | 0.69919062 | 0.023519388 | Down |
|  | A0A376RJ77 | *trg* | Methyl-accepting chemotaxis protein III (Ribose an galactose chemoreceptor protein) OS = *Escherichia coli* OX = 562 GN = trg PE = 4 SV = 1 | 0.698858961 | 0.006827701 | Down |
|  | A0A376HYQ1 | *trpB* | Tryptophan synthase beta chain OS = *Escherichia coli* OX = 562 GN = trpB PE = 3 SV = 1 | 0.697935471 | 0.001560839 | Down |
|  | V0SH21 | HMPREF1595_03595 | Putative selenate reductase, YgfK subunit OS = *Escherichia coli* 907672 OX = 1268982 GN = HMPREF1595_03595 PE = 4 SV = 1 | 0.697867312 | 1.71766E-05 | Down |
|  | A0A4Y8FSS6 | BON92_21880 | ATP-dependent Clp protease ATP-binding subunit ClpA OS = *Escherichia coli* OX = 562 GN = BON92_21880 PE = 3 SV = 1 | 0.697558169 | 2.13643E-06 | Down |
|  | A0A6N7KEC5 | *dmlA* | D-malate dehydrogenase [decarboxylating] OS = *Escherichia coli* OX = 562 GN = dmlA PE = 3 SV = 1 | 0.697268713 | 0.003124225 | Down |
|  | P0AD05 | *yecA* | Uncharacterized protein YecA OS = *Escherichia coli* (strain K12) OX = 83333 GN = yecA PE = 4 SV = 1 | 0.696692199 | 0.039068876 | Down |
|  | A0A6N8Q4X1 | GRW77_29845 | Tagatose-bisphosphate aldolase (Fragment) OS = *Escherichia coli* OX = 562 GN = GRW77_29845 PE = 4 SV = 1 | 0.696415802 | 0.006297522 | Down |
|  | A0A485DVY2 | *dnaJ_2* | Chaperone protein DnaJ OS = *Escherichia coli* OX = 562 GN = dnaJ_2 PE = 3 SV = 1 | 0.695602399 | 0.00311785 | Down |
|  | A0A826XAI7 | *aphA* | Acid phosphatase AphA OS = *Escherichia coli* OX = 562 GN = aphA PE = 4 SV = 1 | 0.69556698 | 0.000407462 | Down |
|  | Q6VMV4 | *fliC* | Flagellin OS = *Escherichia coli* OX = 562 GN = fliC PE = 3 SV = 1 | 0.695119568 | 0.036400596 | Down |
|  | A0A7A3AF22 | HJ940_004371 | YfeK family protein OS = *Escherichia coli* OX = 562 GN = HJ940_004371 PE = 4 SV = 1 | 0.69510737 | 0.023195918 | Down |
|  | A0A829FJ04 | WCS_01189 | Nitrite extrusion protein 1 OS = *Escherichia coli* KTE14 OX = 1169333 GN = WCS_01189 PE = 4 SV = 1 | 0.694396498 | 0.004532239 | Down |
|  | A0A828S3C4 | *rpoN* | RNA polymerase sigma-54 factor OS = *Escherichia coli* STEC_7v OX = 754082 GN = rpoN PE = 4 SV = 1 | 0.693754451 | 0.004357394 | Down |
|  | A0A3Q0MWV8 | *flgE* | Flagellar hook protein FlgE OS = *Escherichia coli* OX = 562 GN = flgE PE = 3 SV = 1 | 0.693239799 | 0.001764136 | Down |
|  | A0A376I0Z6 | *trg_2* | Methyl-accepting chemotaxis protein III (Ribose an galactose chemoreceptor protein) OS = *Escherichia coli* OX = 562 GN = trg_2 PE = 4 SV = 1 | 0.692947165 | 0.040360585 | Down |
|  | A0A831FCK9 | *gatD* | Galactitol-1-phosphate 5-dehydrogenase OS = *Escherichia coli* OX = 562 GN = gatD PE = 4 SV = 1 | 0.692791774 | 0.002993972 | Down |
|  | A0A6C9ILR5 | GKF86_28375 | TonB-dependent receptor plug domain-containing protein (Fragment) OS = *Escherichia coli* OX = 562 GN = GKF86_28375 PE = 4 SV = 1 | 0.691833293 | 0.022602374 | Down |
|  | A0A5F1DXH6 | CQB02_08155 | Alkene reductase OS = *Escherichia coli* OX = 562 GN = CQB02_08155 PE = 4 SV = 1 | 0.690701441 | 0.002839227 | Down |
|  | A0A4Q0BG29 | ELY48_00195 | Thymidylate kinase OS = *Escherichia coli* OX = 562 GN = ELY48_00195 PE = 4 SV = 1 | 0.690469418 | 0.023791416 | Down |
|  | A0A831DIL6 | HMU48_28895 | RNA chaperone Hfq OS = *Escherichia coli* OX = 562 GN = HMU48_28895 PE = 4 SV = 1 | 0.689603299 | 0.00767772 | Down |
|  | A0A6M0PZE3 | *accC* | Biotin carboxylase OS = *Escherichia coli* OX = 562 GN = accC PE = 4 SV = 1 | 0.689198264 | 0.002852606 | Down |
|  | A0A826WYJ8 | CSE52_000824 | 2-hydroxyacyl-CoA dehydratase subunit D OS = *Escherichia coli* OX = 562 GN = CSE52_000824 PE = 4 SV = 1 | 0.688252376 | 0.012975826 | Down |
|  | A0A6M0PTH7 | *manX* | EIIAB-Man OS = *Escherichia coli* OX = 562 GN = manX PE = 4 SV = 1 | 0.687287244 | 0.006523338 | Down |
|  | A0A417ZQI7 | *pspA* | Phage shock protein PspA OS = *Escherichia coli* OX = 562 GN = pspA PE = 3 SV = 1 | 0.685686258 | 0.000454848 | Down |
|  | A0A6L4XIH0 | GP710_11515 | EAL domain-containing protein OS = *Escherichia coli* OX = 562 GN = GP710_11515 PE = 4 SV = 1 | 0.685511563 | 0.005817027 | Down |
|  | A0A6L4XPN6 | *galK* | Galactokinase OS = *Escherichia coli* OX = 562 GN = galK PE = 3 SV = 1 | 0.683609299 | 0.011010537 | Down |
|  | B7NGD6 | *rpsR* | 30S ribosomal protein S18 OS = *Escherichia coli* O17 : K52 : H18 (strain UMN026 / ExPEC) OX = 585056 GN = rpsR PE = 3 SV = 1 | 0.683349369 | 0.015897926 | Down |
|  | A0A3G8RE03 | *caiF* | CaiF/GrlA family transcriptional regulator OS = *Escherichia coli* OX = 562 GN = caiF PE = 4 SV = 1 | 0.68252752 | 0.023783783 | Down |
|  | A0A1V3W1U0 | BMT91_06785 | Transposase OS = *Escherichia coli* OX = 562 GN = BMT91_06785 PE = 3 SV = 1 | 0.682181457 | 0.010251509 | Down |
|  | A0A826YR84 | *srlB* | PTS glucitol/sorbitol transporter subunit IIA OS = *Escherichia coli* OX = 562 GN = srlB PE = 4 SV = 1 | 0.682113372 | 0.008052368 | Down |
|  | A0A6M0PZZ0 | *rhlB* | ATP-dependent RNA helicase RhlB OS = *Escherichia coli* OX = 562 GN = rhlB PE = 3 SV = 1 | 0.681527352 | 0.003564061 | Down |
|  | A0A777TQ67 | GF199_03260 | Aldo/keto reductase OS = *Escherichia coli* OX = 562 GN = GF199_03260 PE = 4 SV = 1 | 0.680948796 | 0.012674028 | Down |
|  | A0A0H3EKR0 | NRG857_10630 | Galactitol-specific PTS system component IIA OS = *Escherichia coli* O83 : H1 (strain NRG 857C / AIEC) OX = 685038 GN = NRG857_10630 PE = 4 SV = 1 | 0.679607995 | 0.000127938 | Down |
|  | A0A376MTF2 | *nagB_1* | Glucosamine-6-phosphate isomerase OS = *Escherichia coli* OX = 562 GN = nagB_1 PE = 4 SV = 1 | 0.679519754 | 0.001009069 | Down |
|  | A0A828S4S8 | *lacF* | Cellobiose-specific phosphotransferase enzyme IIA component OS = *Escherichia coli* STEC_7v OX = 754082 GN = lacF PE = 4 SV = 1 | 0.67851438 | 0.011216884 | Down |
|  | A0A376TX29 | *tdcE_5* | Keto-acid formate acetyltransferase OS = *Escherichia coli* OX = 562 GN = tdcE_5 PE = 4 SV = 1 | 0.676929131 | 0.000373666 | Down |
|  | A0A2X7EYV8 | *rplQ* | 50S ribosomal protein L17 OS = *Escherichia coli* OX = 562 GN = rplQ PE = 3 SV = 1 | 0.675423551 | 0.016657174 | Down |
|  | A0A826X6Z1 | *mepS* | Bifunctional murein DD-endopeptidase/murein LD-carboxypeptidase OS = *Escherichia coli* OX = 562 GN = mepS PE = 4 SV = 1 | 0.674643595 | 0.003650294 | Down |
|  | A0A6M1HYJ4 | *acrA* | Multidrug efflux RND transporter periplasmic adaptor subunit AcrA (Fragment) OS = *Escherichia coli* OX = 562 GN = acrA PE = 3 SV = 1 | 0.674400743 | 0.020837806 | Down |
|  | A0A0K4LWZ9 | *ompX_1* | Outer membrane protein X OS = *Escherichia coli* OX = 562 GN = ompX_1 PE = 3 SV = 1 | 0.6741389 | 0.046204642 | Down |
|  | A0A383G4P5 | *ppc* | Phosphoenolpyruvate carboxylase OS = *Escherichia coli* OX = 562 GN = ppc PE = 3 SV = 1 | 0.673633096 | 0.001112035 | Down |
|  | A0A7U9B3A0 | ECMG_00535 | Chemotaxis protein MotB OS = *Escherichia coli* TA143 OX = 656437 GN = ECMG_00535 PE = 3 SV = 1 | 0.67342865 | 0.010056335 | Down |
|  | A0A447Y522 | *glnA_1* | Glutamine synthetase OS = *Escherichia coli* OX = 562 GN = glnA_1 PE = 3 SV = 1 | 0.673190578 | 0.005334655 | Down |
[truncated: 31,810 more chars]
