# Supplementary material for: In vivo fitness of sul gene-dependent sulfonamide-resistant Escherichia coli in the mammalian gut
Source: mSystems. 2024 Aug 14;9(9):e00836-24. doi: 10.1128/msystems.00836-24 (PMC11406977; doi:10.1128/msystems.00836-24)
Supplement: Table S3 — Differentially expressed protein information related to biological process ontology of compensatory mutant strains S2-1, S2-2, and S2-3. [file msystems.00836-24-s0003.docx]

**Table S3** Differentially expressed protein information related to biological process ontology of compensatory mutant strains S2-1, S2-2, and S2-3

| **Strain** | **Term** | **Description** | ***P*-value** | **Protein List** | **Number** |
| --- | --- | --- | --- | --- | --- |
| S2-1 | GO : 0009310 | amine catabolic process | 0 | A0A6G4BZV9; A0A771BBG3; A0A777SAD9; A0A7U9ASB9; A0A7U9FZE8; A0A827E0G2; A0A853RYC6; A0A8B4PN44; A0A8B5PFJ8; P0A952; A0A6C8TFT0; A0A6C9QTS1 | 12 |
|  | GO : 0042402 | cellular biogenic amine catabolic process | 0 | A0A6G4BZV9; A0A771BBG3; A0A777SAD9; A0A7U9ASB9; A0A7U9FZE8; A0A827E0G2; A0A853RYC6; A0A8B4PN44; A0A8B5PFJ8; P0A952; A0A6C8TFT0; A0A6C9QTS1 | 12 |
|  | GO : 0044248 | cellular catabolic process | 0 | A0A831F693; A0A0E1SXQ0; A0A827JUP0; A0A828URM2; P28904; A0A140NGD0; A0A6D0FFI4; A0A376Q109; A0A4V4SBS9; A0A0K3QKD8; P0AB72; B7MGL2; A0A2X7IQM9; A0A827RWR5; A0A837MF64; A0A8B4IWC9; P76440; U9XXA4; A0A5D8MVY1; A0A377CPF6; A0A6M0PZZ0; A0A7L7XED2; D7Y521; P52129; A0A6M0PZM1; A0A6N8QE93; A0A765T0J1; B6I4S5; F4TAV4; Q8XE60; A0A3L0VT52; A0A831FLZ4; A0A854RJR3; A0A0K4GWR9; B1LDE8; A0A6D0C6T7; A0A7H9LNK0; A0A843M746; A0A6G4BZV9; A0A771BBG3; A0A777SAD9; A0A7U9ASB9; A0A7U9FZE8; A0A827E0G2; A0A853RYC6; A0A8B4PN44; A0A8B5PFJ8; A0A2Y8JQV3; A0A7B5NX36; A0A192EWN6; A0A4U9U1E4; A0A376I3P5; A0A6N8Q332; A0A7D7PKU0; A0A829DGU5; A0A827UE97; B7UQ77; A0A2H4TNY6; A0A6L7A3K9; A0A6M7H4E4; A0A8A5HUG8; A0A377CD10; A0A2X1K1Q5; A0A0P0SWP2; A0A5B9AHZ7; D6I9G0; D3GZF3; A0A4P0YBN2; A0A5P0JCN2; A0A641J6U1; A0A6D0UCH6; A0A6L6RZ23; A0A6N8P9J3; A0A827BBU1; A0A829IMX9; A0A8A9FI50; A0A8B5MA57; A0A6D0GV09; A0A792T099; A0A827NKN5; A0A7D7HRL8; A0A826ZD52; A0A827NRZ2; A0A271QSQ8; A0A789MAE1; A0A4P0YY48; A0A6D0GXH7; A0A827HLW1; A0A7I6H1X7; A0A7U2WD15; A0A8A8NQQ1; A0A7D7I018; A0A827CGX5; A0A774NC69; A0A843NGY2; A0A777RRA0; A0A827EK03; A0A8A5IM57; F4T6A9 | 99 |
|  | GO : 0009056 | catabolic process | 0 | A0A831F693; A0A0E1SXQ0; A0A827JUP0; A0A828URM2; P28904; A0A140NGD0; A0A6D0FFI4; A0A8A5HUG8; A0A376Q109; A0A4V4SBS9; A0A0K3QKD8; P0AB72; B7MGL2; A0A2X7IQM9; A0A827RWR5; A0A837MF64; A0A8B4IWC9; P76440; U9XXA4; A0A5D8MVY1; A0A377CPF6; A0A6M0PZZ0; A0A7L7XED2; D7Y521; P52129; A0A6M0PZM1; A0A6N8QE93; A0A765T0J1; B6I4S5; F4TAV4; Q8XE60; A0A3L0VT52; A0A831FLZ4; A0A854RJR3; A0A0K4GWR9; B1LDE8; A0A6D0C6T7; A0A7H9LNK0; A0A843M746; A0A6G4BZV9; A0A771BBG3; A0A777SAD9; A0A7U9ASB9; A0A7U9FZE8; A0A827E0G2; A0A853RYC6; A0A8B4PN44; A0A8B5PFJ8; P0A952; A0A2Y8JQV3; A0A7B5NX36; A0A192EWN6; A0A4U9U1E4; A0A376I3P5; A0A6N8Q332; A0A7D7PKU0; A0A829DGU5; A0A827UE97; B7UQ77; A0A2H4TNY6; A0A6L7A3K9; A0A6M7H4E4; A0A7H9LX11; A0A377CD10; A0A2X1K1Q5; A0A0P0SWP2; A0A5B9AHZ7; D6I9G0; D3GZF3; A0A4P0YBN2; A0A5P0JCN2; A0A641J6U1; A0A6D0UCH6; A0A6L6RZ23; A0A6N8P9J3; A0A827BBU1; A0A829IMX9; A0A8A9FI50; A0A8B5MA57; A0A6D0GV09; A0A792T099; A0A827NKN5; A0A7D7HRL8; A0A827TFN0; A0A826ZD52; A0A827NRZ2; A0A1X3LVF6; A0A6C8RUQ2; A0A7T6C0A4; A0A271QSQ8; A0A789MAE1; A0A4P0YY48; A0A6D0GXH7; A0A827HLW1; A0A7I6H1X7; A0A7U2WD15; A0A8A8NQQ1; A0A7D7I018; A0A827CGX5; A0A774NC69; A0A843NGY2; A0A6C8TFT0; A0A6C9QTS1; A0A777RRA0; A0A827EK03; A0A8A5IM57; F4T6A9 | 107 |
|  | GO : 1901575 | organic substance catabolic process | 0 | A0A831F693; A0A0E1SXQ0; A0A827JUP0; A0A828URM2; P28904; A0A140NGD0; A0A6D0FFI4; A0A8A5HUG8; A0A376Q109; A0A4V4SBS9; A0A0K3QKD8; P0AB72; B7MGL2; A0A2X7IQM9; A0A827RWR5; A0A837MF64; A0A8B4IWC9; P76440; U9XXA4; A0A5D8MVY1; A0A377CPF6; A0A6M0PZZ0; A0A7L7XED2; D7Y521; P52129; A0A6M0PZM1; A0A6N8QE93; A0A765T0J1; B6I4S5; F4TAV4; Q8XE60; A0A3L0VT52; A0A831FLZ4; A0A854RJR3; A0A0K4GWR9; B1LDE8; A0A6D0C6T7; A0A7H9LNK0; A0A843M746; A0A6G4BZV9; A0A771BBG3; A0A777SAD9; A0A7U9ASB9; A0A7U9FZE8; A0A827E0G2; A0A853RYC6; A0A8B4PN44; A0A8B5PFJ8; P0A952; A0A2Y8JQV3; A0A7B5NX36; A0A192EWN6; A0A4U9U1E4; A0A376I3P5; A0A6N8Q332; A0A7D7PKU0; A0A829DGU5; A0A827UE97; A0A2H4TNY6; A0A6L7A3K9; A0A6M7H4E4; A0A7H9LX11; A0A377CD10; A0A2X1K1Q5; A0A0P0SWP2; A0A5B9AHZ7; D6I9G0; D3GZF3; A0A4P0YBN2; A0A5P0JCN2; A0A641J6U1; A0A6D0UCH6; A0A6L6RZ23; A0A6N8P9J3; A0A827BBU1; A0A829IMX9; A0A8A9FI50; A0A8B5MA57; A0A6D0GV09; A0A792T099; A0A827NKN5; A0A7D7HRL8; A0A827TFN0; A0A826ZD52; A0A827NRZ2; A0A1X3LVF6; A0A6C8RUQ2; A0A7T6C0A4; A0A4P0YY48; A0A6D0GXH7; A0A827HLW1; A0A7I6H1X7; A0A7U2WD15; A0A8A8NQQ1; A0A7D7I018; A0A827CGX5; A0A774NC69; A0A843NGY2; A0A6C8TFT0; A0A6C9QTS1; A0A777RRA0; A0A827EK03; A0A8A5IM57; F4T6A9 | 104 |
|  | GO : 0006576 | cellular biogenic amine metabolic process | 0 | A0A377D334; A0A6M0PTL1; A0A6G4BZV9; A0A771BBG3; A0A777SAD9; A0A7U9ASB9; A0A7U9FZE8; A0A827E0G2; A0A853RYC6; A0A8B4PN44; A0A8B5PFJ8; P0A952; A0A0K4GWR9; B1LDE8; A0A6C8TFT0; A0A6C9QTS1 | 16 |
|  | GO : 0042436 | indole-containing compound catabolic process | 0 | A0A6G4BZV9; A0A771BBG3; A0A777SAD9; A0A7U9ASB9; A0A7U9FZE8; A0A827E0G2; A0A853RYC6; A0A8B4PN44; A0A8B5PFJ8 | 9 |
|  | GO : 0009074 | aromatic amino acid family catabolic process | 0 | A0A6G4BZV9; A0A771BBG3; A0A777SAD9; A0A7U9ASB9; A0A7U9FZE8; A0A827E0G2; A0A853RYC6; A0A8B4PN44; A0A8B5PFJ8 | 9 |
|  | GO : 0046218 | indolalkylamine catabolic process | 0 | A0A6G4BZV9; A0A771BBG3; A0A777SAD9; A0A7U9ASB9; A0A7U9FZE8; A0A827E0G2; A0A853RYC6; A0A8B4PN44; A0A8B5PFJ8 | 9 |
|  | GO : 0006569 | tryptophan catabolic process | 0 | A0A6G4BZV9; A0A771BBG3; A0A777SAD9; A0A7U9ASB9; A0A7U9FZE8; A0A827E0G2; A0A853RYC6; A0A8B4PN44; A0A8B5PFJ8 | 9 |
|  | GO : 0042430 | indole-containing compound metabolic process | 0 | A0A377D334; A0A6M0PTL1; A0A6G4BZV9; A0A771BBG3; A0A777SAD9; A0A7U9ASB9; A0A7U9FZE8; A0A827E0G2; A0A853RYC6; A0A8B4PN44; A0A8B5PFJ8 | 11 |
|  | GO : 0006586 | indolalkylamine metabolic process | 0 | A0A377D334; A0A6M0PTL1; A0A6G4BZV9; A0A771BBG3; A0A777SAD9; A0A7U9ASB9; A0A7U9FZE8; A0A827E0G2; A0A853RYC6; A0A8B4PN44; A0A8B5PFJ8 | 11 |
|  | GO : 0006568 | tryptophan metabolic process | 0 | A0A377D334; A0A6M0PTL1; A0A6G4BZV9; A0A771BBG3; A0A777SAD9; A0A7U9ASB9; A0A7U9FZE8; A0A827E0G2; A0A853RYC6; A0A8B4PN44; A0A8B5PFJ8 | 11 |
|  | GO : 0009308 | amine metabolic process | 0 | A0A377D334; A0A6M0PTL1; A0A6G4BZV9; A0A771BBG3; A0A777SAD9; A0A7U9ASB9; A0A7U9FZE8; A0A827E0G2; A0A853RYC6; A0A8B4PN44; A0A8B5PFJ8; P0A952; A0A0K4GWR9; B1LDE8; B1LQY8; A0A6C8TFT0; A0A6C9QTS1 | 17 |
|  | GO : 0044106 | cellular amine metabolic process | 0 | A0A377D334; A0A6M0PTL1; A0A6G4BZV9; A0A771BBG3; A0A777SAD9; A0A7U9ASB9; A0A7U9FZE8; A0A827E0G2; A0A853RYC6; A0A8B4PN44; A0A8B5PFJ8; P0A952; A0A0K4GWR9; B1LDE8; A0A6C8TFT0; A0A6C9QTS1 | 16 |
|  | GO : 0019402 | galactitol metabolic process | 0 | A0A377CD10; A0A2X1K1Q5; A0A0P0SWP2; A0A5B9AHZ7; D6I9G0; D3GZF3; A0A4P0YBN2; A0A5P0JCN2; A0A641J6U1; A0A6D0UCH6; A0A6L6RZ23; A0A6N8P9J3; A0A827BBU1; A0A829IMX9; A0A8A9FI50; A0A8B5MA57; A0A6N8Q549; A0A789MBN3; A0A826XBG0 | 19 |
|  | GO : 0019404 | galactitol catabolic process | 0 | A0A377CD10; A0A2X1K1Q5; A0A0P0SWP2; A0A5B9AHZ7; D6I9G0; D3GZF3; A0A4P0YBN2; A0A5P0JCN2; A0A641J6U1; A0A6D0UCH6; A0A6L6RZ23; A0A6N8P9J3; A0A827BBU1; A0A829IMX9; A0A8A9FI50; A0A8B5MA57 | 16 |
|  | GO : 2001058 | D-tagatose 6-phosphate metabolic process | 0 | A0A377CD10; A0A2X1K1Q5; A0A0P0SWP2; A0A5B9AHZ7; D6I9G0; D3GZF3; A0A5P0JCN2; A0A641J6U1; A0A6D0UCH6; A0A6L6RZ23; A0A6N8P9J3; A0A827BBU1; A0A829IMX9; A0A8A9FI50; A0A8B5MA57 | 15 |
|  | GO : 2001059 | D-tagatose 6-phosphate catabolic process | 0 | A0A377CD10; A0A2X1K1Q5; A0A0P0SWP2; A0A5B9AHZ7; D6I9G0; D3GZF3; A0A5P0JCN2; A0A641J6U1; A0A6D0UCH6; A0A6L6RZ23; A0A6N8P9J3; A0A827BBU1; A0A829IMX9; A0A8A9FI50; A0A8B5MA57 | 15 |
|  | GO : 0044282 | small molecule catabolic process | 0 | A0A0E1SXQ0; A0A827JUP0; A0A828URM2; A0A0K4GWR9; B1LDE8; A0A6D0C6T7; A0A7H9LNK0; A0A843M746; A0A6G4BZV9; A0A771BBG3; A0A777SAD9; A0A7U9ASB9; A0A7U9FZE8; A0A827E0G2; A0A853RYC6; A0A8B4PN44; A0A8B5PFJ8; A0A2Y8JQV3; A0A7B5NX36; A0A827UE97; A0A2H4TNY6; A0A6L7A3K9; A0A6M7H4E4; A0A7H9LX11; A0A8A5HUG8; A0A377CD10; A0A2X1K1Q5; A0A0P0SWP2; A0A5B9AHZ7; D6I9G0; D3GZF3; A0A4P0YBN2; A0A5P0JCN2; A0A641J6U1; A0A6D0UCH6; A0A6L6RZ23; A0A6N8P9J3; A0A827BBU1; A0A829IMX9; A0A8A9FI50; A0A8B5MA57; A0A6D0GV09; A0A792T099; A0A827NKN5; A0A7D7HRL8; A0A826ZD52; A0A827NRZ2; A0A1X3LVF6; A0A6C8RUQ2; A0A7T6C0A4; A0A827HLW1; A0A7U2WD15; A0A8A8NQQ1; A0A774NC69; A0A843NGY2; A0A6C8TFT0; A0A6C9QTS1; A0A777RRA0; A0A827EK03; A0A8A5IM57; F4T6A9 | 61 |
| S2-2 | GO : 0042430 | indole-containing compound metabolic process | 0 | A0A377D334; A0A6M0PTL1; A0A7U9AWI8; A0A6G4BZV9; A0A771BBG3; A0A777SAD9; A0A7U9ASB9; A0A7U9FZE8; A0A827E0G2; A0A853RYC6; A0A8B4PN44; A0A8B5PFJ8 | 12 |
|  | GO : 0006586 | indolalkylamine metabolic process | 0 | A0A377D334; A0A6M0PTL1; A0A7U9AWI8; A0A6G4BZV9; A0A771BBG3; A0A777SAD9; A0A7U9ASB9; A0A7U9FZE8; A0A827E0G2; A0A853RYC6; A0A8B4PN44; A0A8B5PFJ8 | 12 |
|  | GO : 0006568 | tryptophan metabolic process | 0 | A0A377D334; A0A6M0PTL1; A0A7U9AWI8; A0A6G4BZV9; A0A771BBG3; A0A777SAD9; A0A7U9ASB9; A0A7U9FZE8; A0A827E0G2; A0A853RYC6; A0A8B4PN44; A0A8B5PFJ8 | 12 |
|  | GO : 0044248 | cellular catabolic process | 0 | P15977; A0A831F693; A0A0E1SXQ0; A0A828URM2; P28904; A0A140NGD0; A0A6D0FFI4; A0A417ZXJ2; A0A0E0XTU6; A0A3L2NTY7; A0A376Q109; A0A4V4SBS9; A0A0K3QKD8; P0AB72; B7MGL2; A0A484Y9W9; A0A6L4XG12; A0A837MF64; P76440; U9XXA4; A0A5D8MVY1; A0A377CPF6; A0A7L7XED2; D3QQ79; D7Y521; P52129; A0A3L0VZ91; A0A6M0PZM1; A0A6N8QE93; A0A765T0J1; B6I4S5; F4TAV4; Q8XE60; A0A3L0VT52; A0A831FLZ4; A0A854RJR3; A0A6D0C6T7; A0A7H9LNK0; A0A6N8NDP3; A0A854ADD1; A0A6G4BZV9; A0A771BBG3; A0A777SAD9; A0A7U9ASB9; A0A7U9FZE8; A0A827E0G2; A0A853RYC6; A0A8B4PN44; A0A8B5PFJ8; A0A2Y8JQV3; A0A4U9U1E4; A0A376I3P5; A0A6N8Q332; A0A7D7PKU0; A0A829DGU5; A0A827UE97; U9Y7D9; A0A6L7A3K9; A0A6M7H4E4; A0A377CD10; A0A2X1K1Q5; A0A0P0SWP2; A0A5B9AHZ7; D6I9G0; D3GZF3; A0A4Y9XHJ6; A0A4P0YBN2; A0A5P0JCN2; A0A641J6U1; A0A6D0UCH6; A0A6L6RZ23; A0A6N8P9J3; A0A827BBU1; A0A829IMX9; A0A8A9FI50; A0A8B5MA57; A0A827HIH8; A0A6L4XM71; A0A771MQY9; A0A6D0GV09; A0A0K4PX90; P76015; A0A826ZD52; A0A827NRZ2; A0A271QSQ8; A0A376MN46; A0A789MAE1; A0A6D0EZ53; A0A4P0YY48; A0A6D0GXH7; A0A827HLW1; A0A7U2WD15; A0A8A8NQQ1; A0A7D7I018; A0A827CGX5; A0A774NC69; A0A843NGY2; A0A777RRA0 | 98 |
|  | GO : 0009056 | catabolic process | 0 | P15977; A0A831F693; A0A0E1SXQ0; A0A828URM2; P28904; A0A140NGD0; A0A6D0FFI4; A0A417ZXJ2; A0A0E0XTU6; A0A3L2NTY7; A0A376Q109; A0A4V4SBS9; A0A0K3QKD8; P0AB72; B7MGL2; A0A484Y9W9; A0A6L4XG12; A0A837MF64; P76440; U9XXA4; A0A5D8MVY1; A0A377CPF6; A0A7L7XED2; D3QQ79; D7Y521; P52129; A0A3L0VZ91; A0A6M0PZM1; A0A6N8QE93; A0A765T0J1; B6I4S5; F4TAV4; Q8XE60; A0A3L0VT52; A0A831FLZ4; A0A854RJR3; A0A6D0C6T7; A0A7H9LNK0; A0A6N8NDP3; A0A854ADD1; A0A6G4BZV9; A0A771BBG3; A0A777SAD9; A0A7U9ASB9; A0A7U9FZE8; A0A827E0G2; A0A853RYC6; A0A8B4PN44; A0A8B5PFJ8; P0A952; A0A2Y8JQV3; A0A4U9U1E4; A0A376I3P5; A0A6N8Q332; A0A7D7PKU0; A0A4C4J6A1; A0A829DGU5; A0A827UE97; U9Y7D9; A0A6L7A3K9; A0A6M7H4E4; A0A377CD10; A0A2X1K1Q5; A0A0P0SWP2; A0A5B9AHZ7; D6I9G0; D3GZF3; A0A4Y9XHJ6; A0A4P0YBN2; A0A5P0JCN2; A0A641J6U1; A0A6D0UCH6; A0A6L6RZ23; A0A6N8P9J3; A0A827BBU1; A0A829IMX9; A0A8A9FI50; A0A8B5MA57; A0A827HIH8; A0A6L4XM71; A0A771MQY9; A0A6D0GV09; A0A0K4PX90; P76015; A0A827TFN0; A0A826ZD52; A0A827NRZ2; A0A1X3LVF6; A0A6C8RUQ2; A0A7T6C0A4; A0A271QSQ8; A0A376MN46; A0A789MAE1; A0A6D0EZ53; A0A4P0YY48; A0A6D0GXH7; A0A827HLW1; A0A7U2WD15; A0A8A8NQQ1; A0A7D7I018; A0A827CGX5; A0A774NC69; A0A843NGY2; A0A6C9QTS1; A0A777RRA0 | 105 |
|  | GO : 0009310 | amine catabolic process | 0 | A0A6G4BZV9; A0A771BBG3; A0A777SAD9; A0A7U9ASB9; A0A7U9FZE8; A0A827E0G2; A0A853RYC6; A0A8B4PN44; A0A8B5PFJ8; P0A952; A0A6C9QTS1 | 11 |
|  | GO : 0042402 | cellular biogenic amine catabolic process | 0 | A0A6G4BZV9; A0A771BBG3; A0A777SAD9; A0A7U9ASB9; A0A7U9FZE8; A0A827E0G2; A0A853RYC6; A0A8B4PN44; A0A8B5PFJ8; P0A952; A0A6C9QTS1 | 11 |
|  | GO : 1901575 | organic substance catabolic process | 0 | P15977; A0A831F693; A0A0E1SXQ0; A0A828URM2; P28904; A0A140NGD0; A0A6D0FFI4; A0A417ZXJ2; A0A0E0XTU6; A0A3L2NTY7; A0A376Q109; A0A4V4SBS9; A0A0K3QKD8; P0AB72; B7MGL2; A0A484Y9W9; A0A6L4XG12; A0A837MF64; P76440; U9XXA4; A0A5D8MVY1; A0A377CPF6; A0A7L7XED2; D3QQ79; D7Y521; P52129; A0A3L0VZ91; A0A6M0PZM1; A0A6N8QE93; A0A765T0J1; B6I4S5; F4TAV4; Q8XE60; A0A3L0VT52; A0A831FLZ4; A0A854RJR3; A0A6D0C6T7; A0A7H9LNK0; A0A6N8NDP3; A0A854ADD1; A0A6G4BZV9; A0A771BBG3; A0A777SAD9; A0A7U9ASB9; A0A7U9FZE8; A0A827E0G2; A0A853RYC6; A0A8B4PN44; A0A8B5PFJ8; P0A952; A0A2Y8JQV3; A0A4U9U1E4; A0A376I3P5; A0A6N8Q332; A0A7D7PKU0; A0A4C4J6A1; A0A829DGU5; A0A827UE97; U9Y7D9; A0A6L7A3K9; A0A6M7H4E4; A0A377CD10; A0A2X1K1Q5; A0A0P0SWP2; A0A5B9AHZ7; D6I9G0; D3GZF3; A0A4Y9XHJ6; A0A4P0YBN2; A0A5P0JCN2; A0A641J6U1; A0A6D0UCH6; A0A6L6RZ23; A0A6N8P9J3; A0A827BBU1; A0A829IMX9; A0A8A9FI50; A0A8B5MA57; A0A827HIH8; A0A6L4XM71; A0A771MQY9; A0A6D0GV09; A0A0K4PX90; P76015; A0A827TFN0; A0A826ZD52; A0A827NRZ2; A0A1X3LVF6; A0A6C8RUQ2; A0A7T6C0A4; A0A6D0EZ53; A0A4P0YY48; A0A6D0GXH7; A0A827HLW1; A0A7U2WD15; A0A8A8NQQ1; A0A7D7I018; A0A827CGX5; A0A774NC69; A0A843NGY2; A0A6C9QTS1; A0A777RRA0 | 102 |
|  | GO : 0042436 | indole-containing compound catabolic process | 0 | A0A6G4BZV9; A0A771BBG3; A0A777SAD9; A0A7U9ASB9; A0A7U9FZE8; A0A827E0G2; A0A853RYC6; A0A8B4PN44; A0A8B5PFJ8 | 9 |
|  | GO : 0009074 | aromatic amino acid family catabolic process | 0 | A0A6G4BZV9; A0A771BBG3; A0A777SAD9; A0A7U9ASB9; A0A7U9FZE8; A0A827E0G2; A0A853RYC6; A0A8B4PN44; A0A8B5PFJ8 | 9 |
|  | GO : 0046218 | indolalkylamine catabolic process | 0 | A0A6G4BZV9; A0A771BBG3; A0A777SAD9; A0A7U9ASB9; A0A7U9FZE8; A0A827E0G2; A0A853RYC6; A0A8B4PN44; A0A8B5PFJ8 | 9 |
|  | GO : 0006569 | tryptophan catabolic process | 0 | A0A6G4BZV9; A0A771BBG3; A0A777SAD9; A0A7U9ASB9; A0A7U9FZE8; A0A827E0G2; A0A853RYC6; A0A8B4PN44; A0A8B5PFJ8 | 9 |
|  | GO : 0006059 | hexitol metabolic process | 0 | A0A6N8Q549; A0A789MBN3; A0A826XBG0; A0A377CD10; A0A2X1K1Q5; A0A0P0SWP2; A0A5B9AHZ7; D6I9G0; D3GZF3; A0A4Y9XHJ6; A0A4P0YBN2; A0A5P0JCN2; A0A641J6U1; A0A6D0UCH6; A0A6L6RZ23; A0A6N8P9J3; A0A827BBU1; A0A829IMX9; A0A8A9FI50; A0A8B5MA57; A0A0H2VDQ8; A0A2T1LK09; A0A791VCX8; A0A853WIQ8 | 24 |
|  | GO : 0019402 | galactitol metabolic process | 0 | A0A377CD10; A0A2X1K1Q5; A0A0P0SWP2; A0A5B9AHZ7; D6I9G0; D3GZF3; A0A4Y9XHJ6; A0A4P0YBN2; A0A5P0JCN2; A0A641J6U1; A0A6D0UCH6; A0A6L6RZ23; A0A6N8P9J3; A0A827BBU1; A0A829IMX9; A0A8A9FI50; A0A8B5MA57; A0A6N8Q549; A0A789MBN3; A0A826XBG0 | 20 |
|  | GO : 0009308 | amine metabolic process | 0 | A0A377D334; A0A6M0PTL1; A0A7U9AWI8; A0A140N3E6; A0A6G4BZV9; A0A771BBG3; A0A777SAD9; A0A7U9ASB9; A0A7U9FZE8; A0A827E0G2; A0A853RYC6; A0A8B4PN44; A0A8B5PFJ8; P0A952; B1LQY8; A0A6C9QTS1 | 16 |
|  | GO : 2001058 | D-tagatose 6-phosphate metabolic process | 0 | A0A377CD10; A0A2X1K1Q5; A0A0P0SWP2; A0A5B9AHZ7; D6I9G0; D3GZF3; A0A4Y9XHJ6; A0A5P0JCN2; A0A641J6U1; A0A6D0UCH6; A0A6L6RZ23; A0A6N8P9J3; A0A827BBU1; A0A829IMX9; A0A8A9FI50; A0A8B5MA57 | 16 |
|  | GO : 2001059 | D-tagatose 6-phosphate catabolic process | 0 | A0A377CD10; A0A2X1K1Q5; A0A0P0SWP2; A0A5B9AHZ7; D6I9G0; D3GZF3; A0A4Y9XHJ6; A0A5P0JCN2; A0A641J6U1; A0A6D0UCH6; A0A6L6RZ23; A0A6N8P9J3; A0A827BBU1; A0A829IMX9; A0A8A9FI50; A0A8B5MA57 | 16 |
|  | GO : 0019404 | galactitol catabolic process | 0 | A0A377CD10; A0A2X1K1Q5; A0A0P0SWP2; A0A5B9AHZ7; D6I9G0; D3GZF3; A0A4Y9XHJ6; A0A4P0YBN2; A0A5P0JCN2; A0A641J6U1; A0A6D0UCH6; A0A6L6RZ23; A0A6N8P9J3; A0A827BBU1; A0A829IMX9; A0A8A9FI50; A0A8B5MA57 | 17 |
|  | GO : 0019407 | hexitol catabolic process | 0 | A0A377CD10; A0A2X1K1Q5; A0A0P0SWP2; A0A5B9AHZ7; D6I9G0; D3GZF3; A0A4Y9XHJ6; A0A4P0YBN2; A0A5P0JCN2; A0A641J6U1; A0A6D0UCH6; A0A6L6RZ23; A0A6N8P9J3; A0A827BBU1; A0A829IMX9; A0A8A9FI50; A0A8B5MA57 | 17 |
|  | GO : 0019400 | alditol metabolic process | 0 | A0A0A0FCP4; A0A3P4Z1A7; A0A0K4GBA4; A0A6N8Q549; A0A789MBN3; A0A826XBG0; A0A377CD10; A0A2X1K1Q5; A0A0P0SWP2; A0A5B9AHZ7; D6I9G0; D3GZF3; A0A4Y9XHJ6; A0A4P0YBN2; A0A5P0JCN2; A0A641J6U1; A0A6D0UCH6; A0A6L6RZ23; A0A6N8P9J3; A0A827BBU1; A0A829IMX9; A0A8A9FI50; A0A8B5MA57; A0A0K4PX90; P76015; A0A0H2VDQ8; A0A2T1LK09; A0A791VCX8; A0A853WIQ8 | 29 |
| S2-3 | GO : 0009310 | amine catabolic process | 0 | A0A6G4BZV9; A0A771BBG3; A0A777SAD9; A0A7U9ASB9; A0A7U9FZE8; A0A827E0G2; A0A853RYC6; A0A8B4PN44; A0A8B5PFJ8; P0A952; A0A6C8TFT0; A0A6C9QTS1 | 12 |
|  | GO : 0042402 | cellular biogenic amine catabolic process | 0 | A0A6G4BZV9; A0A771BBG3; A0A777SAD9; A0A7U9ASB9; A0A7U9FZE8; A0A827E0G2; A0A853RYC6; A0A8B4PN44; A0A8B5PFJ8; P0A952; A0A6C8TFT0; A0A6C9QTS1 | 12 |
|  | GO : 0044248 | cellular catabolic process | 0 | P15977; A0A831F693; A0A0E1SXQ0; A0A6L4XH05; A0A827JUP0; A0A828URM2; A0A6L7E3U8; A0A0E0XTU6; A0A376Q109; A0A377CHB4; A0A2X7Q036; P0AB72; A0A376HSY3; I2SXA9; A0A3L0W8F0; A0A7L5VD19; A0A826VW02; A0A837MF64; A0A829DRF6; A0A7A9N155; U9XXA4; A0A6D0J7W9; A0A5D8MVY1; A0A377CPF6; A0A6M0PZZ0; A0A7L7XED2; D3QQ79; D7Y521; A0A3L0W5P1; P52129; A0A6M0PZM1; A0A6N8Q371; A0A6N8QE93; A0A765T0J1; A0A838AU16; B6I4S5; F4TAV4; Q8XE60; A0A3L0VT52; A0A831FLZ4; A0A854RJR3; A0A0K4GWR9; B1LDE8; A0A2X1N7G4; A0A6D0C6T7; A0A7H9LNK0; A0A827VKB6; A0A843M746; A0A6N8NDP3; A0A854ADD1; A0A6G4BZV9; A0A771BBG3; A0A777SAD9; A0A7U9ASB9; A0A7U9FZE8; A0A827E0G2; A0A853RYC6; A0A8B4PN44; A0A8B5PFJ8; A0A2Y8JQV3; A0A7B5NX36; A0A4U9U1E4; A0A484Y4B5; A0A376I3P5; A0A6N8Q332; A0A7D7PKU0; A0A376MCS7; A0A829DGU5; A0A827UE97; B7UQ77; A0A417ZT69; A0A2H4TNY6; A0A6L7A3K9; A0A6M7H4E4; A0A377CD10; A0A2X1K1Q5; A0A0P0SWP2; A0A5B9AHZ7; D6I9G0; D3GZF3; A0A4Y9XHJ6; A0A4P0YBN2; A0A5P0JCN2; A0A641J6U1; A0A6D0UCH6; A0A6L6RZ23; A0A6N8P9J3; A0A827BBU1; A0A829IMX9; A0A831FCK9; A0A8A9FI50; A0A8B5MA57; A0A771MQY9; A0A6D0GV09; P76015; A0A0K4HJP4; A0A862ZHA6; A0A7U9LLG9; A0A826ZD52; A0A827NRZ2; A0A4Y5R2G0; A0A271QSQ8; A0A789MAE1; A0A4P0YY48; A0A6D0GXH7; A0A829L4W9; A0A7L5L228; A0A3A6RSW6; A0A827HLW1; A0A7U2WD15; A0A8A8NQQ1; A0A7D7I018; A0A827CGX5; A0A774NC69; A0A843NGY2; A0A3W5Y3R9; A0A777RRA0; A0A827EK03; A0A8A5IM57; F4T6A9; A0A6G6L238 | 121 |
|  | GO : 1901575 | organic substance catabolic process | 0 | P15977; A0A831F693; A0A0E1SXQ0; A0A6L4XH05; A0A827JUP0; A0A828URM2; A0A6L7E3U8; A0A376HSY3; A0A3L0W8F0; A0A826VW02; A0A829L4W9; A0A0E0XTU6; A0A376Q109; A0A377CHB4; A0A2X7Q036; P0AB72; I2SXA9; A0A7L5VD19; A0A837MF64; A0A829DRF6; A0A7A9N155; U9XXA4; A0A6D0J7W9; A0A5D8MVY1; A0A377CPF6; A0A6M0PZZ0; A0A7L7XED2; D3QQ79; D7Y521; A0A3L0W5P1; P52129; A0A6M0PZM1; A0A6N8Q371; A0A6N8QE93; A0A765T0J1; A0A838AU16; B6I4S5; F4TAV4; Q8XE60; A0A3L0VT52; A0A831FLZ4; A0A854RJR3; A0A0K4GWR9; B1LDE8; A0A2X1N7G4; A0A6D0C6T7; A0A7H9LNK0; A0A827VKB6; A0A843M746; A0A6N8NDP3; A0A854ADD1; A0A6G4BZV9; A0A771BBG3; A0A777SAD9; A0A7U9ASB9; A0A7U9FZE8; A0A827E0G2; A0A853RYC6; A0A8B4PN44; A0A8B5PFJ8; P0A952; A0A2Y8JQV3; A0A7B5NX36; A0A4U9U1E4; A0A484Y4B5; A0A376I3P5; A0A6N8Q332; A0A7D7PKU0; A0A376MCS7; A0A829DGU5; A0A827UE97; A0A377A8R1; A0A417ZT69; A0A2H4TNY6; A0A6L7A3K9; A0A6M7H4E4; A0A7H9LX11; Q8XAW8; A0A377CD10; A0A2X1K1Q5; A0A0P0SWP2; A0A5B9AHZ7; D6I9G0; D3GZF3; A0A4Y9XHJ6; A0A4P0YBN2; A0A5P0JCN2; A0A641J6U1; A0A6D0UCH6; A0A6L6RZ23; A0A6N8P9J3; A0A827BBU1; A0A829IMX9; A0A831FCK9; A0A8A9FI50; A0A8B5MA57; A0A771MQY9; A0A6D0GV09; P76015; A0A0K4HJP4; A0A862ZHA6; A0A7U9LLG9; A0A827TFN0; A0A826ZD52; A0A827NRZ2; A0A4Y5R2G0; A0A1X3LVF6; A0A7H9QPU0; A0A4P0YY48; A0A6D0GXH7; A0A7L5L228; A0A3A6RSW6; A0A827HLW1; A0A7U2WD15; A0A8A8NQQ1; A0A7D7I018; A0A827CGX5; A0A774NC69; A0A843NGY2; A0A6C8TFT0; A0A6C9QTS1; A0A3U1V7R9; A0A3W5Y3R9; A0A777RRA0; A0A827EK03; A0A8A5IM57; F4T6A9; A0A6G6L238 | 128 |
|  | GO : 0009056 | catabolic process | 0 | P15977; A0A831F693; A0A0E1SXQ0; A0A6L4XH05; A0A827JUP0; A0A828URM2; A0A6L7E3U8; A0A376HSY3; A0A3L0W8F0; A0A826VW02; A0A829L4W9; A0A0E0XTU6; A0A376Q109; A0A377CHB4; A0A2X7Q036; P0AB72; I2SXA9; A0A7L5VD19; A0A837MF64; A0A829DRF6; A0A7A9N155; U9XXA4; A0A6D0J7W9; A0A5D8MVY1; A0A377CPF6; A0A6M0PZZ0; A0A7L7XED2; D3QQ79; D7Y521; A0A3L0W5P1; P52129; A0A6M0PZM1; A0A6N8Q371; A0A6N8QE93; A0A765T0J1; A0A838AU16; B6I4S5; F4TAV4; Q8XE60; A0A3L0VT52; A0A831FLZ4; A0A854RJR3; A0A0K4GWR9; B1LDE8; A0A2X1N7G4; A0A6D0C6T7; A0A7H9LNK0; A0A827VKB6; A0A843M746; A0A6N8NDP3; A0A854ADD1; A0A6G4BZV9; A0A771BBG3; A0A777SAD9; A0A7U9ASB9; A0A7U9FZE8; A0A827E0G2; A0A853RYC6; A0A8B4PN44; A0A8B5PFJ8; P0A952; A0A2Y8JQV3; A0A7B5NX36; A0A4U9U1E4; A0A484Y4B5; A0A376I3P5; A0A6N8Q332; A0A7D7PKU0; A0A376MCS7; A0A829DGU5; A0A827UE97; A0A377A8R1; B7UQ77; A0A417ZT69; A0A2H4TNY6; A0A6L7A3K9; A0A6M7H4E4; A0A7H9LX11; Q8XAW8; A0A377CD10; A0A2X1K1Q5; A0A0P0SWP2; A0A5B9AHZ7; D6I9G0; D3GZF3; A0A4Y9XHJ6; A0A4P0YBN2; A0A5P0JCN2; A0A641J6U1; A0A6D0UCH6; A0A6L6RZ23; A0A6N8P9J3; A0A827BBU1; A0A829IMX9; A0A831FCK9; A0A8A9FI50; A0A8B5MA57; A0A771MQY9; A0A6D0GV09; P76015; A0A0K4HJP4; A0A862ZHA6; A0A7U9LLG9; A0A827TFN0; A0A826ZD52; A0A827NRZ2; A0A4Y5R2G0; A0A1X3LVF6; A0A7H9QPU0; A0A271QSQ8; A0A789MAE1; A0A4P0YY48; A0A6D0GXH7; A0A7L5L228; A0A3A6RSW6; A0A827HLW1; A0A7U2WD15; A0A8A8NQQ1; A0A7D7I018; A0A827CGX5; A0A774NC69; A0A843NGY2; A0A6C8TFT0; A0A6C9QTS1; A0A3U1V7R9; A0A3W5Y3R9; A0A777RRA0; A0A827EK03; A0A8A5IM57; F4T6A9; A0A6G6L238 | 131 |
|  | GO : 0044282 | small molecule catabolic process | 0 | A0A0E1SXQ0; A0A6L4XH05; A0A827JUP0; A0A828URM2; A0A376HSY3; A0A3L0W8F0; A0A826VW02; A0A829L4W9; A0A0E0XTU6; A0A0K4GWR9; B1LDE8; A0A2X1N7G4; A0A6D0C6T7; A0A7H9LNK0; A0A827VKB6; A0A843M746; A0A6N8NDP3; A0A854ADD1; A0A6G4BZV9; A0A771BBG3; A0A777SAD9; A0A7U9ASB9; A0A7U9FZE8; A0A827E0G2; A0A853RYC6; A0A8B4PN44; A0A8B5PFJ8; A0A2Y8JQV3; A0A7B5NX36; A0A827UE97; A0A417ZT69; A0A2H4TNY6; A0A6L7A3K9; A0A6M7H4E4; A0A7H9LX11; Q8XAW8; A0A377CD10; A0A2X1K1Q5; A0A0P0SWP2; A0A5B9AHZ7; D6I9G0; D3GZF3; A0A4Y9XHJ6; A0A4P0YBN2; A0A5P0JCN2; A0A641J6U1; A0A6D0UCH6; A0A6L6RZ23; A0A6N8P9J3; A0A827BBU1; A0A829IMX9; A0A831FCK9; A0A8A9FI50; A0A8B5MA57; A0A771MQY9; A0A6D0GV09; P76015; A0A0K4HJP4; A0A862ZHA6; A0A7U9LLG9; A0A826ZD52; A0A827NRZ2; A0A1X3LVF6; A0A7H9QPU0; A0A7L5L228; A0A827HLW1; A0A829DRF6; A0A7U2WD15; A0A8A8NQQ1; A0A774NC69; A0A843NGY2; A0A6C8TFT0; A0A6C9QTS1; A0A3U1V7R9; A0A3W5Y3R9; A0A777RRA0; A0A827EK03; A0A8A5IM57; F4T6A9; A0A6G6L238 | 80 |
|  | GO : 0042436 | indole-containing compound catabolic process | 0 | A0A6G4BZV9; A0A771BBG3; A0A777SAD9; A0A7U9ASB9; A0A7U9FZE8; A0A827E0G2; A0A853RYC6; A0A8B4PN44; A0A8B5PFJ8 | 9 |
|  | GO : 0009074 | aromatic amino acid family catabolic process | 0 | A0A6G4BZV9; A0A771BBG3; A0A777SAD9; A0A7U9ASB9; A0A7U9FZE8; A0A827E0G2; A0A853RYC6; A0A8B4PN44; A0A8B5PFJ8 | 9 |
|  | GO : 0046218 | indolalkylamine catabolic process | 0 | A0A6G4BZV9; A0A771BBG3; A0A777SAD9; A0A7U9ASB9; A0A7U9FZE8; A0A827E0G2; A0A853RYC6; A0A8B4PN44; A0A8B5PFJ8 | 9 |
|  | GO : 0006569 | tryptophan catabolic process | 0 | A0A6G4BZV9; A0A771BBG3; A0A777SAD9; A0A7U9ASB9; A0A7U9FZE8; A0A827E0G2; A0A853RYC6; A0A8B4PN44; A0A8B5PFJ8 | 9 |
|  | GO : 1901606 | alpha-amino acid catabolic process | 0 | A0A0E1SXQ0; A0A6L4XH05; A0A827JUP0; A0A828URM2; A0A0K4GWR9; B1LDE8; A0A2X1N7G4; A0A6D0C6T7; A0A7H9LNK0; A0A827VKB6; A0A843M746; A0A6N8NDP3; A0A854ADD1; A0A6G4BZV9; A0A771BBG3; A0A777SAD9; A0A7U9ASB9; A0A7U9FZE8; A0A827E0G2; A0A853RYC6; A0A8B4PN44; A0A8B5PFJ8; A0A771MQY9; A0A6D0GV09; A0A7L5L228; A0A827EK03; A0A8A5IM57 | 27 |
|  | GO : 0009063 | cellular amino acid catabolic process | 0 | A0A0E1SXQ0; A0A6L4XH05; A0A827JUP0; A0A828URM2; A0A0K4GWR9; B1LDE8; A0A2X1N7G4; A0A6D0C6T7; A0A7H9LNK0; A0A827VKB6; A0A843M746; A0A6N8NDP3; A0A854ADD1; A0A6G4BZV9; A0A771BBG3; A0A777SAD9; A0A7U9ASB9; A0A7U9FZE8; A0A827E0G2; A0A853RYC6; A0A8B4PN44; A0A8B5PFJ8; A0A771MQY9; A0A6D0GV09; A0A7L5L228; A0A827EK03; A0A8A5IM57; A0A2Y8JQV3; A0A7B5NX36 | 29 |
|  | GO : 0009308 | amine metabolic process | 0 | A0A377D334; A0A6G4BZV9; A0A771BBG3; A0A777SAD9; A0A7U9ASB9; A0A7U9FZE8; A0A827E0G2; A0A853RYC6; A0A8B4PN44; A0A8B5PFJ8; P0A952; A0A0K4GWR9; B1LDE8; B1LQY8; A0A4Y5R2G0; A0A6C8TFT0; A0A6C9QTS1 | 17 |
|  | GO : 0019404 | galactitol catabolic process | 0 | A0A377CD10; A0A2X1K1Q5; A0A0P0SWP2; A0A5B9AHZ7; D6I9G0; D3GZF3; A0A4Y9XHJ6; A0A4P0YBN2; A0A5P0JCN2; A0A641J6U1; A0A6D0UCH6; A0A6L6RZ23; A0A6N8P9J3; A0A827BBU1; A0A829IMX9; A0A831FCK9; A0A8A9FI50; A0A8B5MA57 | 18 |
|  | GO : 0006576 | cellular biogenic amine metabolic process | 0 | A0A377D334; A0A6G4BZV9; A0A771BBG3; A0A777SAD9; A0A7U9ASB9; A0A7U9FZE8; A0A827E0G2; A0A853RYC6; A0A8B4PN44; A0A8B5PFJ8; P0A952; A0A0K4GWR9; B1LDE8; A0A6C8TFT0; A0A6C9QTS1 | 15 |
|  | GO : 0019407 | hexitol catabolic process | 0 | A0A377CD10; A0A2X1K1Q5; A0A0P0SWP2; A0A5B9AHZ7; D6I9G0; D3GZF3; A0A4Y9XHJ6; A0A4P0YBN2; A0A5P0JCN2; A0A641J6U1; A0A6D0UCH6; A0A6L6RZ23; A0A6N8P9J3; A0A827BBU1; A0A829IMX9; A0A831FCK9; A0A8A9FI50; A0A8B5MA57 | 18 |
|  | GO : 0044106 | cellular amine metabolic process | 0 | A0A377D334; A0A6G4BZV9; A0A771BBG3; A0A777SAD9; A0A7U9ASB9; A0A7U9FZE8; A0A827E0G2; A0A853RYC6; A0A8B4PN44; A0A8B5PFJ8; P0A952; A0A0K4GWR9; B1LDE8; A0A4Y5R2G0; A0A6C8TFT0; A0A6C9QTS1 | 16 |
|  | GO : 0042430 | indole-containing compound metabolic process | 0 | A0A377D334; A0A6G4BZV9; A0A771BBG3; A0A777SAD9; A0A7U9ASB9; A0A7U9FZE8; A0A827E0G2; A0A853RYC6; A0A8B4PN44; A0A8B5PFJ8 | 10 |
|  | GO : 0006586 | indolalkylamine metabolic process | 0 | A0A377D334; A0A6G4BZV9; A0A771BBG3; A0A777SAD9; A0A7U9ASB9; A0A7U9FZE8; A0A827E0G2; A0A853RYC6; A0A8B4PN44; A0A8B5PFJ8 | 10 |
|  | GO : 0006568 | tryptophan metabolic process | 0 | A0A377D334; A0A6G4BZV9; A0A771BBG3; A0A777SAD9; A0A7U9ASB9; A0A7U9FZE8; A0A827E0G2; A0A853RYC6; A0A8B4PN44; A0A8B5PFJ8 | 10 |
