# Supplementary material for: In vivo fitness of sul gene-dependent sulfonamide-resistant Escherichia coli in the mammalian gut
Source: mSystems. 2024 Aug 14;9(9):e00836-24. doi: 10.1128/msystems.00836-24 (PMC11406977; doi:10.1128/msystems.00836-24)
Supplement: Table S4 — Differentially expressed protein information related to cellular component ontology of compensatory mutant strains S2-1, S2-2, and S2-3. [file msystems.00836-24-s0004.docx]

**Table S4** Differentially expressed protein information related to cellular component ontology of compensatory mutant strains S2-1, S2-2, and S2-3

| **Strain** | **Term** | **Description** | ***P*-value** | **Protein List** | **Number** |
| --- | --- | --- | --- | --- | --- |
| S2-1 | GO : 0060187 | cell pole | 0 | A0A417ZQI7; A0A6G4BZV9; A0A771BBG3; A0A777SAD9; A0A7U9ASB9; A0A7U9FZE8; A0A853RYC6; A0A8B4PN44; A0A8B5PFJ8 | 9 |
|  | GO : 0031234 | extrinsic component of cytoplasmic side of plasma membrane | 0.002 | P06149; A0A5N3D643; A0A417ZQI7; A0A6A0Q765; A0A770C125 | 5 |
|  | GO : 0009424 | bacterial-type flagellum hook | 0.02 | P29744; A0A2A3WKJ5; A0A5D8S3Y2; A0A7U3BCZ4 | 4 |
|  | GO : 0009421 | bacterial-type flagellum filament cap | 0.03 | A0A2A3WKJ5; A0A5D8S3Y2 | 2 |
|  | GO : 0009331 | glycerol-3-phosphate dehydrogenase complex | 0.034 | A0A6L9DJA7; A0A792T099; A0A826SG38; A0A827NKN5 | 4 |
|  | GO : 0019897 | extrinsic component of plasma membrane | 0.041 | P06149; A0A5N3D643; A0A417ZQI7; A0A6A0Q765; A0A770C125 | 5 |
|  | GO : 0019898 | extrinsic component of membrane | 0.043 | P06149; A0A5N3D643; A0A417ZQI7; A0A6A0Q765; A0A770C125; A0A8B4IWC9 | 6 |
|  | GO : 0044461 | bacterial-type flagellum part | 0.07 | P04949; A0A4C4K7Q3; B3SGP3; A0A2A3WKJ5; A0A5D8S3Y2; P29744; A0A7U3BCZ4; A0A3Q0MWV8; A0A0A0H5V9; J7QSF1; A0A6D0G0H1 | 11 |
|  | GO : 0044463 | cell projection part | 0.07 | P04949; A0A4C4K7Q3; B3SGP3; A0A2A3WKJ5; A0A5D8S3Y2; P29744; A0A7U3BCZ4; A0A3Q0MWV8; A0A0A0H5V9; J7QSF1; A0A6D0G0H1 | 11 |
|  | GO : 1905368 | peptidase complex | 0.079 | A0A5F1DL70; B6I4S5 | 2 |
|  | GO : 0000502 | proteasome complex | 0.079 | B6I4S5; A0A5F1DL70 | 2 |
|  | GO : 0009376 | HslUV protease complex | 0.079 | A0A5F1DL70; B6I4S5 | 2 |
|  | GO : 1905369 | endopeptidase complex | 0.079 | A0A5F1DL70; B6I4S5 | 2 |
|  | GO : 0031597 | cytosolic proteasome complex | 0.079 | A0A5F1DL70; B6I4S5 | 2 |
|  | GO : 0042995 | cell projection | 0.099 | C6UY58; U9Y2M9; P04949; A0A4C4K7Q3; B3SGP3; A0A2A3WKJ5; A0A5D8S3Y2; P29744; A0A7U3BCZ4; A0A3Q0MWV8; A0A0A0H5V9; J7QSF1; A0A6D0G0H1 | 13 |
|  | GO : 0070069 | cytochrome complex | 0.103 | A0A845P5Y3; A0A376I8B9; A0A828L8H3 | 3 |
|  | GO : 0009288 | bacterial-type flagellum | 0.124 | P04949; A0A4C4K7Q3; B3SGP3; A0A2A3WKJ5; A0A5D8S3Y2; P29744; A0A7U3BCZ4; A0A3Q0MWV8; A0A0A0H5V9; J7QSF1; A0A6D0G0H1; C6UY58 | 12 |
|  | GO : 0043231 | intracellular membrane-bounded organelle | 0.14 | A0A7T8PQW7; A0A853WGK5 | 2 |
|  | GO : 0043227 | membrane-bounded organelle | 0.145 | A0A853WGK5; A0A7T8PQW7; A0A8A8YI47 | 3 |
|  | GO : 0005667 | transcription factor complex | 0.172 | A0A0B1LVL6 | 1 |
| S2-2 | GO : 0060187 | cell pole | 0 | A0A417ZQI7; A0A6G4BZV9; A0A771BBG3; A0A777SAD9; A0A7U9ASB9; A0A7U9FZE8; A0A853RYC6; A0A8B4PN44; A0A8B5PFJ8 | 9 |
|  | GO : 0031234 | extrinsic component of cytoplasmic side of plasma membrane | 0.002 | P06149; A0A5N3D643; A0A417ZQI7; A0A6A0Q765; A0A770C125 | 5 |
|  | GO : 0019897 | extrinsic component of plasma membrane | 0.008 | P06149; A0A5N3D643; A0A417ZQI7; A0A6A0Q765; A0A770C125; A0A2T1LFH3 | 6 |
|  | GO : 0009346 | citrate lyase complex | 0.009 | P75726; A0A1X3LUN7; A0A6D0ILH3; A0A826Q325 | 4 |
|  | GO : 0070069 | cytochrome complex | 0.017 | A0A376I8B9; A0A3W5XXF0; A0A6D0DK91; A0A828L8H3 | 4 |
|  | GO : 1990060 | maltose transport complex | 0.035 | A0A6A0Q765; A0A770C125; A0A829DLS5 | 3 |
|  | GO : 0019898 | extrinsic component of membrane | 0.037 | A0A2T1LFH3; P06149; A0A5N3D643; A0A417ZQI7; A0A6A0Q765; A0A770C125 | 6 |
|  | GO : 0005887 | integral component of plasma membrane | 0.047 | P11350; A0A7H9QSS3; A0A828AVB4; A0A6A0Q765; A0A770C125; A0A829DLS5; P06149; A0A5N3D643; A0A3L9GVF5; A0A376HGP0; A0A376J8Y2; A0A376UFH3; P0A844; A0A5F1DU45; A0A3W5XXF0; A0A6D0DK91; A0A6L4XNV3; A0A6P1KG06; A0A7L5VD13; A0A826L3Z6; A0A826VXD8; A0A828L8H3; A0A829DGU5; A0A829FJ04; A0A829GBE0; A0A853WGK5 | 26 |
|  | GO : 0030288 | outer membrane-bounded periplasmic space | 0.062 | A0A376RLB5; P0ACD8; A0A0L1C331; A0A2A3WTJ2; A0A376HZ91; A0A4T5JV04; A0A6L4XP60; A0A790HG40; A0A7A2X3S7; A0A7D5H5I7; A0A7D7PKU0; A0A826KAA6; A0A826R875; A0A826TZQ7; A0A828NK69; A0A828UTS3; I2STI4; J7QZG7 | 18 |
|  | GO : 1905368 | peptidase complex | 0.073 | A0A5F1DL70; B6I4S5 | 2 |
|  | GO : 0000502 | proteasome complex | 0.073 | B6I4S5; A0A5F1DL70 | 2 |
|  | GO : 0009376 | HslUV protease complex | 0.073 | A0A5F1DL70; B6I4S5 | 2 |
|  | GO : 1905369 | endopeptidase complex | 0.073 | A0A5F1DL70; B6I4S5 | 2 |
|  | GO : 0031597 | cytosolic proteasome complex | 0.073 | A0A5F1DL70; B6I4S5 | 2 |
|  | GO : 0009898 | cytoplasmic side of plasma membrane | 0.081 | P06149; A0A5N3D643; A0A417ZQI7; A0A6A0Q765; A0A770C125; D3QQ79 | 6 |
|  | GO : 0098562 | cytoplasmic side of membrane | 0.081 | D3QQ79; P06149; A0A5N3D643; A0A417ZQI7; A0A6A0Q765; A0A770C125 | 6 |
|  | GO : 0031226 | intrinsic component of plasma membrane | 0.086 | P06149; A0A5N3D643; A0A3L9GVF5; A0A376HGP0; A0A376J8Y2; P11350; A0A376UFH3; P0A844; A0A5F1DU45; A0A3W5XXF0; A0A6D0DK91; A0A6L4XNV3; A0A6P1KG06; A0A7L5VD13; A0A826L3Z6; A0A826VXD8; A0A828L8H3; A0A829DGU5; A0A829FJ04; A0A829GBE0; A0A853WGK5; A0A7H9QSS3; A0A828AVB4; A0A6A0Q765; A0A770C125; A0A829DLS5 | 26 |
|  | GO : 0042597 | periplasmic space | 0.11 | A0A376RLB5; P0ACD8; A0A0L1C331; A0A2A3WTJ2; A0A376HZ91; A0A4T5JV04; A0A6L4XP60; A0A790HG40; A0A7A2X3S7; A0A7D5H5I7; A0A7D7PKU0; A0A826KAA6; A0A826R875; A0A826TZQ7; A0A828NK69; A0A828UTS3; I2STI4; J7QZG7; A0A376NYE6; Q8X5W6; A0A4T4XPV4; A0A0V9G8Z5; A0A2X1N9Q1; A0A2T1LHD8; A0A826MHE4; A0A826TR55; A0A828FSI3; A0A853WCM3 | 28 |
|  | GO : 0043231 | intracellular membrane-bounded organelle | 0.131 | A0A7T8PQW7; A0A853WGK5 | 2 |
|  | GO : 0033281 | TAT protein transport complex | 0.131 | A0A3L9GVF5; P0A844 | 2 |
| S2-3 | GO : 0060187 | cell pole | 0 | A0A417ZQI7; A0A6G4BZV9; A0A771BBG3; A0A777SAD9; A0A7U9ASB9; A0A7U9FZE8; A0A822UG86; A0A827KTD0; A0A853RYC6; A0A8B4PN44; A0A8B5PFJ8 | 11 |
|  | GO : 1990060 | maltose transport complex | 0.001 | A0A6A0Q765; A0A770C125; A0A828NV63; A0A829DCS4; A0A829DLS5 | 5 |
|  | GO : 0043226 | organelle | 0.002 | A0A853WGK5; C6UY58; A0A376I0W6; A0A417ZT69; A0A7T8PQW7; A0A2X1JEL2; P02359; A0A5B9AS13; B1LR79; A0A377ABT5; B7UK44; Q1R633; A0A5R8TDT4; A7ZQ46; A1AGC3; P0ADZ5; A0A376I523; A0A0T5XRJ0; B7NGD6; V0YFL6; A0A376JGA1; A0A2X7EYV8; A0A2T1LCI8; A0A2X3LQH0; Q0TCE3; A0A2X5F0L7; A0A2X1PTA3; A0A2T1LFH3; A0A7U0H0D9; A0A793U2L0; A0A0A0H460; A0A6C9QTS1; P0A7M2; A0A6D0H698; A0A6M0PU76; A0A7A6MWE8; A0A7B2TEE0; A0A7U2Z0M6; A0A6M0PR83; A0A6N8PVK8; A0A787CMN5; A0A789RQS2; A0A7A7AT69; A0A7L5L2P8; A0A822UG86; A0A6M0PZR8; P04949; A0A4C4K7Q3; A1Z1Z5; Q6VMV4; B3SGP3; A0A2A3WKJ5; A0A5D8S3Y2; P29744; A0A0K3STS8; A0A7U3BCZ4; A0A3Q0MWV8; A0A0A0H5V9; J7QSF1; A0A6D0G0H1; A0A830TAZ3; A0A377CSN6; A0A376WBS3; A0A2T1LEM2; A0A5B9ARY7; A0A2T1LE54; A0A1D3KW65 | 67 |
|  | GO : 0009346 | citrate lyase complex | 0.003 | P75726; A0A1X3LUN7; A0A6D0ILH3; A0A826Q325; A0A827EG33 | 5 |
|  | GO : 0043228 | non-membrane-bounded organelle | 0.003 | C6UY58; A0A376I0W6; A0A2X1JEL2; P02359; A0A5B9AS13; B1LR79; A0A377ABT5; B7UK44; Q1R633; A0A5R8TDT4; A7ZQ46; A1AGC3; P0ADZ5; A0A376I523; A0A0T5XRJ0; B7NGD6; V0YFL6; A0A376JGA1; A0A2X7EYV8; A0A2T1LCI8; A0A2X3LQH0; Q0TCE3; A0A2X5F0L7; A0A2X1PTA3; A0A2T1LFH3; A0A7U0H0D9; A0A793U2L0; A0A0A0H460; A0A6C9QTS1; P0A7M2; A0A6D0H698; A0A6M0PU76; A0A7A6MWE8; A0A7B2TEE0; A0A7U2Z0M6; A0A6M0PR83; A0A6N8PVK8; A0A787CMN5; A0A789RQS2; A0A7A7AT69; A0A7L5L2P8; A0A822UG86; A0A377CSN6; A0A376WBS3; A0A2T1LEM2; A0A5B9ARY7; A0A2T1LE54; A0A1D3KW65; A0A6M0PZR8; P04949; A0A4C4K7Q3; A1Z1Z5; Q6VMV4; B3SGP3; A0A2A3WKJ5; A0A5D8S3Y2; P29744; A0A0K3STS8; A0A7U3BCZ4; A0A3Q0MWV8; A0A0A0H5V9; J7QSF1; A0A6D0G0H1; A0A830TAZ3 | 64 |
|  | GO : 0009898 | cytoplasmic side of plasma membrane | 0.004 | P06149; A0A5N3D643; A0A417ZQI7; A0A6A0Q765; A0A770C125; P11349; D3QQ79; A0A3L0W5P1; A0A827KTD0; A0A838AU16 | 10 |
|  | GO : 0098562 | cytoplasmic side of membrane | 0.004 | D3QQ79; A0A3L0W5P1; A0A827KTD0; A0A838AU16; P06149; A0A5N3D643; A0A417ZQI7; A0A6A0Q765; A0A770C125; P11349 | 10 |
|  | GO : 0005840 | ribosome | 0.005 | P0A7M2; A0A6D0H698; A0A6M0PU76; A0A7A6MWE8; A0A7B2TEE0; A0A7U2Z0M6; P02359; A0A6M0PR83; A0A6N8PVK8; A0A787CMN5; A0A789RQS2; A0A7A7AT69; A0A7L5L2P8; A0A822UG86; A0A377CSN6; A0A376WBS3; A0A2T1LEM2; A0A5B9ARY7; A0A2T1LE54; A0A1D3KW65; A0A2X1JEL2; A0A5B9AS13; B1LR79; A0A377ABT5; B7UK44; Q1R633; A0A5R8TDT4; A7ZQ46; A1AGC3; P0ADZ5; A0A376I523; A0A0T5XRJ0; B7NGD6; V0YFL6; A0A376JGA1; A0A2X7EYV8; A0A2T1LCI8; A0A2X3LQH0; Q0TCE3; A0A2X5F0L7; A0A2X1PTA3 | 41 |
|  | GO : 1990904 | ribonucleoprotein complex | 0.007 | A0A2X1JEL2; P02359; A0A5B9AS13; B1LR79; A0A377ABT5; B7UK44; Q1R633; A0A5R8TDT4; A7ZQ46; A1AGC3; P0ADZ5; A0A376I523; A0A0T5XRJ0; B7NGD6; V0YFL6; A0A376JGA1; A0A2X7EYV8; A0A2T1LCI8; A0A2X3LQH0; Q0TCE3; A0A2X5F0L7; A0A2X1PTA3; A0A377CSN6; A0A376WBS3; A0A2T1LEM2; A0A5B9ARY7; A0A2T1LE54; A0A1D3KW65; P0A7M2; A0A6D0H698; A0A6M0PU76; A0A7A6MWE8; A0A7B2TEE0; A0A7U2Z0M6; A0A6M0PR83; A0A6N8PVK8; A0A787CMN5; A0A789RQS2; A0A7A7AT69; A0A7L5L2P8; A0A822UG86 | 41 |
|  | GO : 0044444 | cytoplasmic part | 0.008 | A0A6M0PZR8; A0A417ZT69; P15977; P07650; P76015; P02359; P0A6W5; P69222; P24203; L3CFU3; P37760; P23331; P0A7M2; P0ABE2; P0ACV8; P26608; A0A1E5M202; A0A1M2EKK4; A0A2J1D5L3; A0A376I976; A0A376ZHZ8; A0A377KA41; A0A3A6RSW6; A0A417ZQI7; A0A4P0YBN2; A0A4P0YY48; A0A4P8BZI1; A0A4T4Z8E0; A0A5B9AWW1; A0A5B9B029; A0A5F0Q1S1; A0A660HCD7; A0A660HE83; A0A6D0HB74; A0A6D0LQA8; A0A6G2G671; A0A6G4BN73; A0A6G4BZV9; A0A6G6KXJ4; A0A6L4XH05; A0A6L4XKB9; A0A6L7E3U8; A0A6M0PSK1; A0A6M0PZE3; A0A6M0PZM1; A0A6N4KV03; A0A6N6XAM7; A0A6N6Y316; A0A6N8NDP3; A0A6N8PRW7; A0A6N8PVJ3; A0A6N8Q371; A0A6N8QE93; A0A6N9S2P9; A0A768K1M3; A0A771BBG3; A0A771MQY9; A0A777CE63; A0A777CQL7; A0A777RRA0; A0A777SAD9; A0A7A2V838; A0A7A2WX01; A0A7B5B9V3; A0A7D5L4D1; A0A7D7I018; A0A7H9LQH0; A0A7H9LX11; A0A7H9QJD6; A0A7H9QPU0; A0A7I9AJI9; A0A7I9ASU8; A0A7L5L228; A0A7L5VD19; A0A7L7XED2; A0A7T2N4B4; A0A7U0H0D9; A0A7U9A075; A0A7U9ASB9; A0A7U9FZE8; A0A810UTR3; A0A826HVW3; A0A826NJ86; A0A826RU97; A0A826TRD1; A0A826X2M8; A0A826X348; A0A826X499; A0A826YKV9; A0A826ZD52; A0A827ABQ5; A0A827CGX5; A0A827G257; A0A827KTD0; A0A827LJ87; A0A827NRZ2; A0A827VKB6; A0A827X5P6; A0A828FPP9; A0A828HF64; A0A828MYB1; A0A828S4S8; A0A828S740; A0A828URM2; A0A831DIL6; A0A831FCK9; A0A831FLZ4; A0A837MF64; A0A837Y776; A0A843M746; A0A843MJ33; A0A853RYC6; A0A853WKY2; A0A854ADD1; A0A854RJR3; A0A862ZHA6; A0A8A5ILA3; A0A8A5IM57; A0A8A8PXR3; A0A8A8PXU7; A0A8B4PN44; A0A8B5PFJ8; B1LQY8; B6I140; D3GY01; D6JGR5; P0AB15; P64465; U9XK73; A0A2X1JEL2; A0A5B9AS13; B1LR79; A0A377ABT5; B7UK44; Q1R633; A0A5R8TDT4; A7ZQ46; A1AGC3; P0ADZ5; A0A376I523; A0A0T5XRJ0; B7NGD6; V0YFL6; A0A376JGA1; A0A2X7EYV8; A0A2T1LCI8; A0A2X3LQH0; Q0TCE3; A0A2X5F0L7; A0A2X1PTA3; A0A7T8PQW7; C3SRM0; A0A826SG38; P75726; A0A1X3LUN7; A0A6D0ILH3; A0A826Q325; A0A827EG33; A0A376Q109; A0A377CHB4; A0A377CSN6; A0A376WBS3; A0A2T1LEM2; A0A5B9ARY7; A0A2T1LE54; A0A1D3KW65; A0A6D0H698; A0A6M0PU76; A0A7A6MWE8; A0A7B2TEE0; A0A7U2Z0M6; A0A6M0PR83; A0A6N8PVK8; A0A787CMN5; A0A789RQS2; A0A7A7AT69; A0A7L5L2P8; A0A822UG86; A0A829L4W9; A0A5F1DL70; B6I4S5 | 181 |
|  | GO : 0009424 | bacterial-type flagellum hook | 0.009 | P29744; A0A0K3STS8; A0A2A3WKJ5; A0A5D8S3Y2; A0A7U3BCZ4 | 5 |
|  | GO : 0031234 | extrinsic component of cytoplasmic side of plasma membrane | 0.009 | P06149; A0A5N3D643; A0A417ZQI7; A0A6A0Q765; A0A770C125 | 5 |
|  | GO : 0005887 | integral component of plasma membrane | 0.01 | P11349; A0A7H9QSS3; A0A828AVB4; A0A829JLQ8; A0A6A0Q765; A0A770C125; A0A828NV63; A0A829DCS4; A0A829DLS5; P02942; P06149; A0A5N3D643; A0A3L9GVF5; A0A376RTV4; A0A376HGP0; A0A376J8Y2; A0A3L3AP41; A0A4Z0TMS0; A0A3W5XXF0; A0A6L4XNV3; A0A6N4KV03; A0A777GNP1; A0A7D7HDD9; A0A7H9LUA1; A0A7L5VD13; A0A810UTR3; A0A823ADS0; A0A826VXD8; A0A826X793; A0A828L8H3; A0A829DGU5; A0A829DWE3; A0A829FJ04; A0A829GBE0; A0A845P5Y3; A0A853WGK5; A0A8A5IAW5 | 37 |
|  | GO : 0044422 | organelle part | 0.011 | P04949; A0A4C4K7Q3; A1Z1Z5; Q6VMV4; B3SGP3; A0A2A3WKJ5; A0A5D8S3Y2; P29744; A0A0K3STS8; A0A7U3BCZ4; A0A3Q0MWV8; A0A0A0H5V9; J7QSF1; A0A6D0G0H1; A0A830TAZ3; P0A7M2; A0A6D0H698; A0A6M0PU76; A0A7A6MWE8; A0A7B2TEE0; A0A7U2Z0M6; P02359; A0A6M0PR83; A0A6N8PVK8; A0A787CMN5; A0A789RQS2; A0A7A7AT69; A0A7L5L2P8; A0A822UG86; A0A377CSN6; A0A376WBS3; A0A2T1LEM2; A0A5B9ARY7; A0A2T1LE54; A0A1D3KW65; A0A6M0PZR8 | 36 |
|  | GO : 0055052 | ATP-binding cassette (ABC) transporter complex; substrate-binding subunit-containing | 0.011 | A0A828NV63; A0A829DCS4; A0A829DLS5; A0A829GBE0 | 4 |
|  | GO : 0044391 | ribosomal subunit | 0.013 | A0A377CSN6; A0A376WBS3; A0A2T1LEM2; A0A5B9ARY7; A0A2T1LE54; A0A1D3KW65; P0A7M2; A0A6D0H698; A0A6M0PU76; A0A7A6MWE8; A0A7B2TEE0; A0A7U2Z0M6; P02359; A0A6M0PR83; A0A6N8PVK8; A0A787CMN5; A0A789RQS2; A0A7A7AT69; A0A7L5L2P8; A0A822UG86 | 20 |
|  | GO : 0043229 | intracellular organelle | 0.014 | A0A853WGK5; A0A417ZT69; A0A7T8PQW7; A0A2X1JEL2; P02359; A0A5B9AS13; B1LR79; A0A377ABT5; B7UK44; Q1R633; A0A5R8TDT4; A7ZQ46; A1AGC3; P0ADZ5; A0A376I523; A0A0T5XRJ0; B7NGD6; V0YFL6; A0A376JGA1; A0A2X7EYV8; A0A2T1LCI8; A0A2X3LQH0; Q0TCE3; A0A2X5F0L7; A0A2X1PTA3; A0A2T1LFH3; A0A7U0H0D9; A0A793U2L0; A0A0A0H460; A0A6C9QTS1; P0A7M2; A0A6D0H698; A0A6M0PU76; A0A7A6MWE8; A0A7B2TEE0; A0A7U2Z0M6; A0A6M0PR83; A0A6N8PVK8; A0A787CMN5; A0A789RQS2; A0A7A7AT69; A0A7L5L2P8; A0A822UG86; A0A6M0PZR8; A0A377CSN6; A0A376WBS3; A0A2T1LEM2; A0A5B9ARY7; A0A2T1LE54; A0A1D3KW65 | 50 |
|  | GO : 0031226 | intrinsic component of plasma membrane | 0.016 | P02942; P06149; A0A5N3D643; A0A3L9GVF5; A0A376RTV4; A0A376HGP0; A0A376J8Y2; A0A3L3AP41; A0A4Z0TMS0; A0A3W5XXF0; A0A6L4XNV3; A0A6N4KV03; A0A777GNP1; A0A7D7HDD9; A0A7H9LUA1; A0A7L5VD13; A0A810UTR3; A0A823ADS0; A0A826VXD8; A0A826X793; A0A828L8H3; A0A829DGU5; A0A829DWE3; A0A829FJ04; A0A829GBE0; A0A845P5Y3; A0A853WGK5; A0A8A5IAW5; P11349; A0A7H9QSS3; A0A828AVB4; A0A829JLQ8; A0A6A0Q765; A0A770C125; A0A828NV63; A0A829DCS4; A0A829DLS5; A0A827KTD0 | 38 |
|  | GO : 0043232 | intracellular non-membrane-bounded organelle | 0.02 | A0A2X1JEL2; P02359; A0A5B9AS13; B1LR79; A0A377ABT5; B7UK44; Q1R633; A0A5R8TDT4; A7ZQ46; A1AGC3; P0ADZ5; A0A376I523; A0A0T5XRJ0; B7NGD6; V0YFL6; A0A376JGA1; A0A2X7EYV8; A0A2T1LCI8; A0A2X3LQH0; Q0TCE3; A0A2X5F0L7; A0A2X1PTA3; A0A2T1LFH3; A0A7U0H0D9; A0A793U2L0; A0A0A0H460; A0A6C9QTS1; P0A7M2; A0A6D0H698; A0A6M0PU76; A0A7A6MWE8; A0A7B2TEE0; A0A7U2Z0M6; A0A6M0PR83; A0A6N8PVK8; A0A787CMN5; A0A789RQS2; A0A7A7AT69; A0A7L5L2P8; A0A822UG86; A0A377CSN6; A0A376WBS3; A0A2T1LEM2; A0A5B9ARY7; A0A2T1LE54; A0A1D3KW65; A0A6M0PZR8 | 47 |
|  | GO : 0098552 | side of membrane | 0.02 | A0A2X1N950; D3QQ79; A0A3L0W5P1; A0A827KTD0; A0A838AU16; P06149; A0A5N3D643; A0A417ZQI7; A0A6A0Q765; A0A770C125; P11349; A0A7H9QSS3; A0A828AVB4; A0A829JLQ8; A0A377E1X8 | 15 |
