# Supplementary material for: In vivo fitness of sul gene-dependent sulfonamide-resistant Escherichia coli in the mammalian gut
Source: mSystems. 2024 Aug 14;9(9):e00836-24. doi: 10.1128/msystems.00836-24 (PMC11406977; doi:10.1128/msystems.00836-24)
Supplement: Supplemental tables — Table S5 and TS6. [file msystems.00836-24-s0005.docx]

**Table S5** Differentially expressed protein information related to molecular function ontology of the compensatory mutant strains S2-1, S2-2, and S2-3

| **Strain** | **Term** | **Description** | ***P*-value** | **Protein List** | **Number** |
| --- | --- | --- | --- | --- | --- |
| S2-1 | GO : 0009034 | tryptophanase activity | 0 | A0A2A3VUL7; A0A2X7H313; A8A6H0; A0A418U310; A0A659GVV1; W9AMC1; A0A377BSB0; A0A376S463; A0A6G4BZV9; A0A771BBG3; A0A777SAD9; A0A7U9ASB9; A0A7U9FZE8; A0A827E0G2; A0A853RYC6; A0A8B4PN44; A0A8B5PFJ8 | 17 |
|  | GO : 0008081 | phosphoric diester hydrolase activity | 0 | A0A192EWN6; A0A4U9U1E4; A0A6N8Q332; A0A7D7PKU0; A0A2U2VLW9; A0A485JCI0; A0A3L0VV29; A0A6N6YDL1; A0A6N9MUA6; A0A7D7DMB0; A0A7I8Z834; F4SG82; A0A2H4TXE8; A0A2T1LIJ2; Q46774; A0A6M0PYN9 | 16 |
|  | GO : 0080146 | L-cysteine desulfhydrase activity | 0 | A0A6G4BZV9; A0A771BBG3; A0A777SAD9; A0A7U9ASB9; A0A7U9FZE8; A0A853RYC6; A0A8B4PN44; A0A8B5PFJ8 | 8 |
|  | GO : 0008889 | glycerophosphodiester phosphodiesterase activity | 0 | A0A2U2VLW9; A0A485JCI0; A0A3L0VV29; A0A6N6YDL1; A0A6N9MUA6; A0A7D7DMB0; A0A7I8Z834 | 7 |
|  | GO : 0051213 | dioxygenase activity | 0 | A0A6G4BZV9; A0A771BBG3; A0A777SAD9; A0A7U9ASB9; A0A7U9FZE8; A0A827E0G2; A0A853RYC6; A0A8B4PN44; A0A8B5PFJ8 | 9 |
|  | GO : 0004333 | fumarate hydratase activity | 0.001 | P14407; A0A0L1C3K3; A0A5N8HAY0; A0A6L4XMK4; A0A6M0PUJ1; A0A7Z1F0N1; A0A826X499 | 7 |
|  | GO : 0016846 | carbon-sulfur lyase activity | 0.001 | A0A0J8XKA8; A0A6G4BZV9; A0A771BBG3; A0A777SAD9; A0A7U9ASB9; A0A7U9FZE8; A0A853RYC6; A0A8B4PN44; A0A8B5PFJ8 | 9 |
|  | GO : 0015605 | organophosphate ester transmembrane transporter activity | 0.001 | A0A2T1LIF6; A0A5B1FAQ8; A0A777HIK4; A0A7L5V678 | 4 |
|  | GO : 0015169 | glycerol-3-phosphate transmembrane transporter activity | 0.001 | A0A2T1LIF6; A0A5B1FAQ8; A0A777HIK4; A0A7L5V678 | 4 |
|  | GO : 0008254 | 3'-nucleotidase activity | 0.001 | A0A4U9U1E4; A0A6N8Q332; A0A7D7PKU0; A0A827HLW1 | 4 |
|  | GO : 0042578 | phosphoric ester hydrolase activity | 0.001 | A0A0L1C331; A0A234XVQ4; A0A826XAI7; C6UY58; F4SG82; A0A2H4TXE8; A0A2T1LIJ2; Q46774; A0A6M0PYN9; A0A827HLW1; A0A4U9U1E4; A0A6N8Q332; A0A7D7PKU0; A0A192EWN6; A0A2U2VLW9; A0A485JCI0; A0A3L0VV29; A0A6N6YDL1; A0A6N9MUA6; A0A7D7DMB0; A0A7I8Z834; A0A829DGU5; P77475; D3QWK1; A0A377B2G3 | 25 |
|  | GO : 0016830 | carbon-carbon lyase activity | 0.001 | P0AB72; A0A377D334; A0A6G2G671; A0A7D7HRL8; A0A6G4BN73; A0A843M746; A0A6L7A3K9; A0A0K4GWR9; B1LDE8; A0A1X3LUN7; A0A827L354; A0A828UQB5; A0A6D0C6T7; A0A7H9LNK0; A0A2X3A7G0; A0A5F1T0A9; A0A383G4P5; A0A2A3VUL7; A0A2X7H313; A8A6H0; A0A418U310; A0A659GVV1; W9AMC1; A0A377BSB0; A0A376S463; A0A6G4BZV9; A0A771BBG3; A0A777SAD9; A0A7U9ASB9; A0A7U9FZE8; A0A827E0G2; A0A853RYC6; A0A8B4PN44; A0A8B5PFJ8; A0A7U9FZE7; A0A6N7KEC5 | 36 |
|  | GO : 0103111 | D-glucosamine PTS permease activity | 0.002 | B7MEE9; A0A6D0HB74; A0A768EGW4; A0A789MBN3; A0A826XBG0; A0A826XI71; A0A826YR84; A0A828HF64; A0A828S4S8; A0A828UNV2; A0A8A8YI47 | 11 |
|  | GO : 0031420 | alkali metal ion binding | 0.002 | A0A376I0P4; A0A3L0W8Z5; A0A6G4BZV9; A0A771BBG3; A0A777SAD9; A0A7U9ASB9; A0A7U9FZE8; A0A853RYC6; A0A8B4PN44; A0A8B5PFJ8; A0A826XAA9 | 11 |
|  | GO : 0016699 | oxidoreductase activity; acting on hydrogen as donor; iron-sulfur protein as acceptor | 0.003 | A0A3Q0N2H5; W1F624; A0A2X6R2J2; P0ACD8; A0A6C9TKU4; A0A855FRH0 | 6 |
|  | GO : 0008901 | ferredoxin hydrogenase activity | 0.003 | A0A3Q0N2H5; W1F624; A0A2X6R2J2; P0ACD8; A0A6C9TKU4; A0A855FRH0 | 6 |
|  | GO : 0004176 | ATP-dependent peptidase activity | 0.003 | F4TAV4; Q8XE60; A0A3L0VT52; A0A827TFN0; A0A831FLZ4; A0A854RJR3 | 6 |
|  | GO : 0033748 | hydrogenase (acceptor) activity | 0.004 | A0A3Q0N2H5; W1F624; A0A2X6R2J2; P0ACD8 | 4 |
|  | GO : 0004834 | tryptophan synthase activity | 0.004 | Q6JZ44; Q6JZC5; A0A376HYQ1; A0A6M0PTL1 | 4 |
|  | GO : 0008663 | 2'; 3'-cyclic-nucleotide 2'-phosphodiesterase activity | 0.004 | A0A192EWN6; A0A4U9U1E4; A0A6N8Q332; A0A7D7PKU0 | 4 |
| S2-2 | GO : 0009034 | tryptophanase activity | 0.000 | A0A2A3VUL7; A0A2X7H313; A8A6H0; A0A418U310; W9AMC1; A0A377BSB0; A0A376S463; A0A6G4BZV9; A0A771BBG3; A0A777SAD9; A0A7U9ASB9; A0A7U9FZE8; A0A827E0G2; A0A853RYC6; A0A8B4PN44; A0A8B5PFJ8 | 16 |
|  | GO : 0080146 | L-cysteine desulfhydrase activity | 0.000 | A0A6G4BZV9; A0A771BBG3; A0A777SAD9; A0A7U9ASB9; A0A7U9FZE8; A0A853RYC6; A0A8B4PN44; A0A8B5PFJ8 | 8 |
|  | GO : 0008081 | phosphoric diester hydrolase activity | 0.000 | A0A4U9U1E4; A0A6N8Q332; A0A7D7PKU0; A0A2U2VLW9; A0A485JCI0; A0A3L0VV29; A0A6N6YDL1; A0A6N9MUA6; A0A7I8Z834; A0A140N3E6; F4SG82; A0A2H4TXE8; A0A2T1LIJ2; Q46774; A0A6M0PYN9 | 15 |
|  | GO : 0051213 | dioxygenase activity | 0.000 | A0A6L6I9I8; A0A6G4BZV9; A0A771BBG3; A0A777SAD9; A0A7U9ASB9; A0A7U9FZE8; A0A827E0G2; A0A853RYC6; A0A8B4PN44; A0A8B5PFJ8 | 10 |
|  | GO : 0004834 | tryptophan synthase activity | 0.000 | Q6JZ44; Q6JZC5; A0A376HYQ1; A0A6M0PTL1; A0A7U9AWI8 | 5 |
|  | GO : 0070001 | aspartic-type peptidase activity | 0.001 | A0A1X3LV10; A0A3L5HCC8; A0A5D8QXC9; A0A1X3LME0; A0A828GWE9; A0A855W463; A0A8B5F2G2 | 7 |
|  | GO : 0004190 | aspartic-type endopeptidase activity | 0.001 | A0A1X3LV10; A0A3L5HCC8; A0A5D8QXC9; A0A1X3LME0; A0A828GWE9; A0A855W463; A0A8B5F2G2 | 7 |
|  | GO : 0016846 | carbon-sulfur lyase activity | 0.001 | A0A0J8XKA8; A0A6G4BZV9; A0A771BBG3; A0A777SAD9; A0A7U9ASB9; A0A7U9FZE8; A0A853RYC6; A0A8B4PN44; A0A8B5PFJ8 | 9 |
|  | GO : 0004654 | polyribonucleotide nucleotidyltransferase activity | 0.001 | D7Y521; A0A3L0VZ91; A0A6N8QE93; A0A765T0J1 | 4 |
|  | GO : 0008254 | 3'-nucleotidase activity | 0.001 | A0A4U9U1E4; A0A6N8Q332; A0A7D7PKU0; A0A827HLW1 | 4 |
|  | GO : 0008889 | glycerophosphodiester phosphodiesterase activity | 0.001 | A0A2U2VLW9; A0A485JCI0; A0A3L0VV29; A0A6N6YDL1; A0A6N9MUA6; A0A7I8Z834 | 6 |
|  | GO : 0031420 | alkali metal ion binding | 0.001 | A0A3L0W8Z5; A0A6G4BZV9; A0A771BBG3; A0A777SAD9; A0A7U9ASB9; A0A7U9FZE8; A0A827J5L2; A0A853RYC6; A0A8B4PN44; A0A8B5PFJ8; A0A826XAA9 | 11 |
|  | GO : 0004175 | endopeptidase activity | 0.001 | F4TAV4; Q8XE60; A0A3L0VT52; A0A2T1LH30; A0A6D0ELA1; A0A827TFN0; A0A831FLZ4; A0A854RJR3; A0A1X3LV10; A0A3L5HCC8; A0A5D8QXC9; A0A1X3LME0; A0A828GWE9; A0A855W463; A0A8B5F2G2; A0A5C9ACD7; A0A2T1LHD8; B6I4S5; A0A6L4XNV3; A0A826X6Z1 | 20 |
|  | GO : 0005506 | iron ion binding | 0.001 | A0A6L4XKB9; A0A6L6I9I8; A0A843MJ33; E6BDM7; A0A376KS23; A0A377DBF7; A0A660HCD7; A0A828FPP9; P25516; A0A376NYE6; B7N7L8; A0A1V2GF16; A0A2T1LHJ7; A0A6L9C2L7; A0A7D5H5I7; A0A7Z8HVR4; A0A828FSI3; A0A828UTS3; A0A829KPZ7 | 19 |
|  | GO : 0004176 | ATP-dependent peptidase activity | 0.002 | F4TAV4; Q8XE60; A0A3L0VT52; A0A827TFN0; A0A831FLZ4; A0A854RJR3 | 6 |
|  | GO : 0020037 | heme binding | 0.003 | A0A271QSQ8; A0A376MN46; A0A376NYE6; Q1R585; P11350; A0A222QNW9; A0A3W5XXF0; A0A6D0DK91; A0A789MAE1; A0A7B5PWP4; A0A828FSI3; A0A828L8H3; A0A828UTS3 | 13 |
|  | GO : 0030955 | potassium ion binding | 0.003 | A0A3L0W8Z5; A0A6G4BZV9; A0A771BBG3; A0A777SAD9; A0A7U9ASB9; A0A7U9FZE8; A0A827J5L2; A0A853RYC6; A0A8B4PN44; A0A8B5PFJ8 | 10 |
|  | GO : 0046906 | tetrapyrrole binding | 0.004 | A0A271QSQ8; A0A376MN46; A0A376NYE6; Q1R585; P11350; A0A222QNW9; A0A3W5XXF0; A0A6D0DK91; A0A789MAE1; A0A7B5PWP4; A0A828FSI3; A0A828L8H3; A0A828UTS3; A0A6D0GV09 | 14 |
|  | GO : 0016829 | lyase activity | 0.004 | P25516; A0A0J8XKA8; P0AB72; A0A6L4XG12; P14407; A0A0L1C3K3; A0A5N8HAY0; A0A6L4XMK4; A0A7Z1F0N1; A0A826X499; A0A377D334; A0A376HXM1; Q6JZ44; Q6JZC5; A0A376HYQ1; A0A6M0PTL1; A0A7U9AWI8; A0A376S994; M9H7W0; A0A2T1LDM7; A0A6L7A3K9; A0A2X1N3J8; A0A8A8PXR3; P75726; A0A1X3LUN7; A0A6D0ILH3; A0A826Q325; A0A827L354; A0A828UQB5; A0A6D0C6T7; A0A7H9LNK0; A0A376I7H5; A0A383G4P5; A0A2A3VUL7; A0A2X7H313; A8A6H0; A0A418U310; W9AMC1; A0A377BSB0; A0A376S463; A0A6G4BZV9; A0A771BBG3; A0A777SAD9; A0A7U9ASB9; A0A7U9FZE8; A0A827E0G2; A0A853RYC6; A0A8B4PN44; A0A8B5PFJ8; A0A7U9FZE7; A0A377CFY3; A0A6N7KEC5; K4XKM3; A0A5B9AHZ7; P39384; A0A193LNB2; A0A376M3X3; A0A0A0FJG0; A0A5P0JCN2; A0A641J6U1; A0A6C9TKU4; A0A6D0ISL3; A0A6D0UCH6; A0A6L6RZ23; A0A6N8NDP3; A0A7D7DU33; A0A7I7HZN9; A0A7U9B1F9; A0A826YKV9; A0A828R1M2; A0A828S740; A0A854ADD1; A0A8A9FI50; A0A8B5MA57 | 74 |
|  | GO : 0004333 | fumarate hydratase activity | 0.004 | P14407; A0A0L1C3K3; A0A5N8HAY0; A0A6L4XMK4; A0A7Z1F0N1; A0A826X499 | 6 |
| S2-3 | GO : 0009034 | tryptophanase activity | 0 | A0A2A3VUL7; A0A2X7H313; A8A6H0; A0A418U310; A0A659GVV1; W9AMC1; A0A376S463; A0A6G4BZV9; A0A771BBG3; A0A777SAD9; A0A7U9ASB9; A0A7U9FZE8; A0A827E0G2; A0A853RYC6; A0A8B4PN44; A0A8B5PFJ8 | 16 |
|  | GO : 0005198 | structural molecule activity | 0 | A0A2X1JEL2; A0A377CSN6; A0A5B9ARY7; P02359; A0A5B9AS13; B1LR79; A0A377ABT5; B7UK44; Q1R633; A0A376WBS3; A7ZQ46; A1AGC3; P0ADZ5; A0A376I523; A0A2T1LEM2; A0A0T5XRJ0; A0A2T1LE54; B7NGD6; V0YFL6; A0A376JGA1; A0A2X7EYV8; A0A1D3KW65; A0A2T1LCI8; A0A2X3LQH0; P0A7M2; Q0TCE3; A0A2X5F0L7; A0A2X1PTA3; A0A6D0H698; A0A6M0PR83; A0A6M0PU76; A0A6N8PVK8; A0A787CMN5; A0A789RQS2; A0A7A6MWE8; A0A7A7AT69; A0A7B2TEE0; A0A7L5L2P8; A0A7U2Z0M6; P04949; A0A4C4K7Q3; A1Z1Z5; Q6VMV4; P29744; A0A0K3STS8; B3SGP3; A0A2T1LHJ7; A0A0A0H460; A0A6C9QTS1; A0A6L4XKB9; A0A827KNR7 | 51 |
|  | GO : 0008081 | phosphoric diester hydrolase activity | 0 | A0A4U9U1E4; A0A6N8Q332; A0A7D7PKU0; A0A2U2VLW9; A0A485JCI0; A0A3L0VV29; A0A6N6YDL1; A0A6N9MUA6; A0A7I8Z834; A0A1X3HZY6; F4SG82; A0A2H4TXE8; A0A2T1LIJ2; A0A140N469; Q46774; A0A6M0PYN9; A0A827VF59 | 17 |
|  | GO : 0080146 | L-cysteine desulfhydrase activity | 0 | A0A6G4BZV9; A0A771BBG3; A0A777SAD9; A0A7U9ASB9; A0A7U9FZE8; A0A853RYC6; A0A8B4PN44; A0A8B5PFJ8 | 8 |
|  | GO : 0003735 | structural constituent of ribosome | 0 | A0A2X1JEL2; A0A377CSN6; A0A5B9ARY7; P02359; A0A5B9AS13; B1LR79; A0A377ABT5; B7UK44; Q1R633; A0A376WBS3; A7ZQ46; A1AGC3; P0ADZ5; A0A376I523; A0A2T1LEM2; A0A0T5XRJ0; A0A2T1LE54; B7NGD6; V0YFL6; A0A376JGA1; A0A2X7EYV8; A0A1D3KW65; A0A2T1LCI8; A0A2X3LQH0; P0A7M2; Q0TCE3; A0A2X5F0L7; A0A2X1PTA3; A0A6D0H698; A0A6M0PR83; A0A6M0PU76; A0A6N8PVK8; A0A787CMN5; A0A789RQS2; A0A7A6MWE8; A0A7A7AT69; A0A7B2TEE0; A0A7L5L2P8; A0A7U2Z0M6 | 39 |
|  | GO : 0015144 | carbohydrate transmembrane transporter activity | 0.001 | A0A6N4KV03; A0A828NV63; A0A829DCS4; A0A1E5WU20; A0A417ZXX0; B7MEE9; A0A5D8MJ02; W1X0Z6; A0A0K9TBS3; B7UFB0; A0A3A6RSW6; A0A6D0HB74; A0A768EGW4; A0A826XBG0; A0A826XI71; A0A826YR84; A0A828HF64; A0A828S4S8; Q8X712; A0A8A8NRS0; A0A7L5VD13; A0A8A5HAP0; A0A6A0Q765; A0A770C125; A0A829DLS5; C5A130; A0A080IBY9; A0A8B3HX17; A0A777HIK4; A0A810UTR3; A0A383FA69; A0A3L0VXR4 | 32 |
|  | GO : 0019843 | rRNA binding | 0.001 | A0A376WBS3; A0A2T1LCI8; A0A6M0PU76; A0A787CMN5; A0A7A7AT69; A0A2X1JEL2; A0A377CSN6; A0A5B9ARY7; P02359; A0A5B9AS13; B1LR79; A0A377ABT5; B7UK44; Q1R633; P69222; P0ADZ5; A0A2T1LEM2; A0A0T5XRJ0; A0A2T1LE54; B7NGD6; A0A376JGA1; A0A1D3KW65; A0A2X3LQH0; P0A7M2; Q0TCE3; A0A0J1XV59; A0A6D0H698; A0A789RQS2; A0A7A6MWE8; A0A7B2TEE0; A0A7B4HWA4 | 31 |
|  | GO : 0051213 | dioxygenase activity | 0.001 | A0A6G4BZV9; A0A771BBG3; A0A777SAD9; A0A799XZ20; A0A7U9ASB9; A0A7U9FZE8; A0A827E0G2; A0A853RYC6; A0A8B4PN44; A0A8B5PFJ8 | 10 |
|  | GO : 0004176 | ATP-dependent peptidase activity | 0.002 | F4TAV4; Q8XE60; A0A3L0VT52; A0A7B5B9V3; A0A827TFN0; A0A831FLZ4; A0A854RJR3 | 7 |
|  | GO : 0015605 | organophosphate ester transmembrane transporter activity | 0.003 | A0A2T1LIF6; A0A5B1FAQ8; A0A777HIK4; A0A7L5V678 | 4 |
|  | GO : 0015169 | glycerol-3-phosphate transmembrane transporter activity | 0.003 | A0A2T1LIF6; A0A5B1FAQ8; A0A777HIK4; A0A7L5V678 | 4 |
|  | GO : 0008254 | 3'-nucleotidase activity | 0.003 | A0A4U9U1E4; A0A6N8Q332; A0A7D7PKU0; A0A827HLW1 | 4 |
|  | GO : 0042578 | phosphoric ester hydrolase activity | 0.003 | A0A0L1C331; A0A234XVQ4; A0A826XAI7; C6UY58; A0A376I0W6; F4SG82; A0A2H4TXE8; A0A2T1LIJ2; A0A140N469; Q46774; A0A6M0PYN9; A0A827VF59; A0A827HLW1; A0A4U9U1E4; A0A6N8Q332; A0A7D7PKU0; A0A2U2VLW9; A0A485JCI0; A0A3L0VV29; A0A6N6YDL1; A0A6N9MUA6; A0A7I8Z834; A0A3U1V7R9; A0A829DGU5; A0A6N8QK59; D3QWK1; A0A377B2G3; A0A7U9B8D8; A0A1X3HZY6 | 29 |
|  | GO : 0008815 | citrate (pro-3S)-lyase activity | 0.003 | P75726; A0A1X3LUN7; A0A6D0ILH3; A0A826Q325; A0A827EG33 | 5 |
|  | GO : 0016829 | lyase activity | 0.004 | A0A7L5L5F3; A0A376PPP7; A0A827VKB6; A0A843M746; A0A6M0PW36; A0A6N8PZ81; A0A0J8XKA8; A0A376MCS7; A0A418GR70; P0AB72; A0A0L1C3K3; A0A6L4XMK4; A0A6M0PUJ1; A0A7Z1F0N1; A0A826X499; A0A377D334; A0A6G2G671; A0A5D8SN25; A0A6G4BN73; Q6JZC5; A0A376HYQ1; Q1RA34; A0A376S994; A0A376D3K9; A0A3W4D6A0; A0A831BLF9; A0A6L7A3K9; A0A828B2R6; A0A0K4GWR9; B1LDE8; A0A0K3TCZ7; A0A2X1N3J8; A0A8A8PXR3; P75726; A0A1X3LUN7; A0A6D0ILH3; A0A826Q325; A0A827EG33; A0A827L354; A0A447XSI9; A0A7A6P9I7; A0A6C8TFT0; A0A7B5NX36; A0A6D0C6T7; A0A7H9LNK0; A0A376I7H5; A0A2X3A7G0; A0A8A5ILA3; A0A383G4P5; A0A2A3VUL7; A0A2X7H313; A8A6H0; A0A418U310; A0A659GVV1; W9AMC1; A0A376S463; A0A6G4BZV9; A0A771BBG3; A0A777SAD9; A0A7U9ASB9; A0A7U9FZE8; A0A827E0G2; A0A853RYC6; A0A8B4PN44; A0A8B5PFJ8; B7UQ77; A0A417ZT69; A0A377CFY3; A0A6N7KEC5; A0A6C9FXZ7; A0A6G6L238; K4XKM3; A0A5B9AHZ7; P39384; A0A376RTV4; A0A2X1N7G4; A0A376M3X3; A0A0A0FJG0; A0A5P0JCN2; A0A641J6U1; A0A6C9TKU4; A0A6D0UCH6; A0A6G6KXJ4; A0A6L6RZ23; A0A6N8NDP3; A0A7A2WWV5; A0A7D7DU33; A0A7I7HZN9; A0A7U9B1F9; A0A826YKV9; A0A828R1M2; A0A828S740; A0A854ADD1; A0A8A5IM57; A0A8A9FI50; A0A8B5F6C9; A0A8B5MA57 | 97 |
|  | GO : 0008889 | glycerophosphodiester phosphodiesterase activity | 0.006 | A0A2U2VLW9; A0A485JCI0; A0A3L0VV29; A0A6N6YDL1; A0A6N9MUA6; A0A7I8Z834 | 6 |
|  | GO : 0016846 | carbon-sulfur lyase activity | 0.007 | A0A0J8XKA8; A0A6G4BZV9; A0A771BBG3; A0A777SAD9; A0A7U9ASB9; A0A7U9FZE8; A0A853RYC6; A0A8B4PN44; A0A8B5PFJ8 | 9 |
|  | GO : 0004033 | aldo-keto reductase (NADP) activity | 0.009 | A0A3W5Y3R9; A0A777RRA0; A0A827LJ87; A0A831EYL4; A0A827ABQ5 | 5 |
|  | GO : 0005363 | maltose transmembrane transporter activity | 0.01 | A0A6A0Q765; A0A770C125; A0A829DLS5; C5A130; A0A080IBY9; A0A8B3HX17; A0A828NV63; A0A829DCS4 | 8 |
|  | GO : 0016830 | carbon-carbon lyase activity | 0.01 | A0A376MCS7; P0AB72; A0A377D334; A0A6G2G671; A0A5D8SN25; A0A6G4BN73; A0A827VKB6; A0A843M746; A0A6L7A3K9; A0A0K4GWR9; B1LDE8; A0A0K3TCZ7; P75726; A0A1X3LUN7; A0A6D0ILH3; A0A826Q325; A0A827EG33; A0A827L354; A0A6D0C6T7; A0A7H9LNK0; A0A2X3A7G0; A0A8A5ILA3; A0A383G4P5; A0A2A3VUL7; A0A2X7H313; A8A6H0; A0A418U310; A0A659GVV1; W9AMC1; A0A376S463; A0A6G4BZV9; A0A771BBG3; A0A777SAD9; A0A7U9ASB9; A0A7U9FZE8; A0A827E0G2; A0A853RYC6; A0A8B4PN44; A0A8B5PFJ8; A0A6N7KEC5; A0A6G6L238 | 41 |

**Table S6** Pathway enrichment analysis of differentially expressed proteins in compensatory mutant strains S2-1, S2-2, and S2-3

| **Strain** | **Pathway-Name** | ***P*-value** | **Protein List** | **Number** |
| --- | --- | --- | --- | --- |
| S2-1 | Galactose metabolism | 9.98e-07 | E6BD97; A0A828HF64; A0A5B9AHZ7; A0A3Y1V1S8; A0A6D0H6A5; A0A7H9LVC8; A0A789MBN3; A0A377CD10; A0A6L6RZ23; A0A5P0JCN2; A0A479JTS0; C0ST84; A0A6D0UCH6; A0A641J6U1; A0A4P0YBN2; A0A8A9FI50; A0A829IMX9; B7MEE9; A0A1X3LVF6; A0A2X1K1Q5; A0A1E5WU20; A0A826XBG0; A0A7B5NX36; D3GZF3; A0A827BBU1; A0A6N8P9J3; A0A844UUA4; A0A826WTR0; A0A8B5MA57; B7UFB0; A0A0H3EKR0; D6I9G0; W1X365; A0A768EGW4; A0A0P0SWP2; A0A1M2GPQ1 | 36 |
|  | Tryptophan metabolism | 1.13e-06 | A8A6H0; A0A2X7H313; A0A8B4PN44; A0A771BBG3; A0A377BSB0; A0A6G4BZV9; A0A7U9FZE8; A0A7U9ASB9; W9AMC1; A0A659GVV1; K4XKM3; A0A271QSQ8; A0A8B5PFJ8; A0A853RYC6; A0A418U310; A0A827E0G2; A0A789MAE1; A0A777SAD9; A0A2A3VUL7 | 19 |
|  | Nitrotoluene degradation | 0.000759 | P0ACD8; A0A827CGX5; A0A3Q0N2H5; A0A2X6R2J2; A0A5F1DXH6; W1F624; A0A7D7I018 | 7 |
|  | Glycerophospholipid metabolism | 0.000811 | F4SG82; A0A6M0PYN9; A0A6N6YDL1; A0A7I8Z834; A0A6D0USM6; A0A829DGU5; A0A2T1LIJ2; A0A2H4TXE8; A0A3L0VV29; A0A827NKN5; A0A826SG38; A0A485JCI0; A0A6L9DJA7; A0A792T099; A0A7D7DMB0; A0A6C8TFT0; A0A2U2VLW9; A0A6N9MUA6 | 18 |
|  | Flagellar assembly | 0.00495 | P29744; P26608; P04949; A0A2A3WKJ5; A0A828S3C4; A0A6D0FG95; A0A7U9B3A0; J7QSF1; A0A7U3BCZ4; A0A4C4K7Q3; A0A6D0G0H1; A0A377FAX0; A0A3Q0MWV8; A0A377K5F9; A0A5D1FPW1; A0A5D8S3Y2; A0A7T2N4B4; E9THW5; B3SGP3; A0A0A0H5V9 | 20 |
|  | Pyrimidine metabolism | 0.005795 | P76440; A0A827HLW1; A0A6N8Q332; A0A376D6E8; A0A4U9U1E4; A0A3L0VYM4; A0A6M0PVJ7; A0A6N8PRW7; A0A346GGF1; A0A7I6H1X7; B1LQY8; A0A192EWN6; A0A1X9TR86; A0A2X1JNR5; A0A4Y8G431; A0A6D0EIL2; A0A418GJM4; A0A2H9EUJ5; A0A7A2V838; A0A376I3P5; A0A7D7PKU0; A0A827UE97; A0A5F1DTT1; A0A5F1E0X3; A0A0A1A5H6 | 25 |
|  | RNA degradation | 0.013411 | A0A6N8QE93; A0A777CE63; A0A6M0PZZ0; A0A831DIL6; A0A5D8MVY1; A0A765T0J1; A0A7L7XED2; A0A6S4W2J6; A0A3L0WAU3; A0A3L0W137; A0A376LG23; D7Y521; A0A377CPF6 | 13 |
|  | Pyruvate; metabolism | 0.02109 | P06149; P14407; B1IY62; F4T7V4; A0A6M0PSQ9; A0A6L4XMK4; A0A2H9EVR4; A0A5F1T0A9; A0A6M0PUJ1; A0A837MF64; A0A826X499; A0A7Z1F0N1; A0A376I0P4; A0A376I0H2; A0A210BJJ3; C3SRM0; A0A376Q109; A0A377CFY3; A0A827RWR5; A0A2X3A7G0; A0A0L1C3K3; A0A383G4P5; A0A7Z8HVR4; A0A3L0VV53; A0A6N8PVJ3; A0A8B4IWC9; A0A3L0W8Z5; A0A6M0PZE3; A0A8A5IM57; A0A5N3D643; A0A7H9QZ14; U9YDR6 | 32 |
|  | Phosphotransferase system (PTS) | 0.021412 | A0A417ZXX0; A0A828HF64; A0A5D8MJ02; A0A789MBN3; A0A479JTS0; C0ST84; B7MEE9; A0A828UNV2; A0A1E5WU20; A0A828S4S8; A0A826XBG0; B7UFB0; A0A0H3EKR0; A0A826XI71; A0A768EGW4; A0A826YR84; A0A6D0HB74 | 17 |
|  | Cell cycle-Caulobacter | 0.069592 | V0SFH5; F4TAV4; A0A3L0VT52; Q8XE60; A0A831FLZ4; A0A854RJR3; A0A2T1LPX8 | 7 |
|  | MAPK signaling pathway-plant | 0.07864 | A0A853WGK5; A0A2H9EUJ5 | 2 |
|  | Ferroptosis | 0.07864 | P0A952; A0A826JV02 | 2 |
|  | Biofilm formation-Vibrio cholerae | 0.124077 | A0A831DIL6; A0A828S3C4; A0A6M0PZR8; A0A6M0PZM1; A0A6L4XLZ2; A0A827K1F8; A0A7T2N4B4 | 7 |
|  | Phenylalanine, tyrosine and tryptophan biosynthesis | 0.130292 | E6BD92; A0A376HYQ1; A0A377D334; Q6JZC5; Q6JZ44; A0A2I6IHS3; A0A6L4XR91; A0A6M0PTL1 | 8 |
|  | Citrate cycle (TCA cycle) | 0.137634 | P14407; B1IY62; F4T7V4; A0A6L4XMK4; A0A2H9EVR4; A0A6M0PUJ1; A0A837MF64; A0A826X499; A0A7Z1F0N1; A0A376Q109; A0A827RWR5; A0A0L1C3K3; A0A7Z8HVR4; A0A3L0VV53; A0A8B4IWC9 | 15 |
|  | Riboflavin metabolism | 0.138305 | A0A6D0IPV2; A0A826XAI7; A0A1U9SZS3; A0A0L1C331; A0A234XVQ4; A0A1X3KRU5 | 6 |
|  | Secondary bile acid biosynthesis | 0.139555 | A0A826ZD52; A0A827NRZ2 | 2 |
|  | Flavone and flavonol biosynthesis | 0.172145 | A0A8A5HUG8 | 1 |
|  | Lysosome | 0.172145 | A0A8A5HUG8 | 1 |
|  | Inositol phosphate metabolism | 0.206816 | A0A0K3QKD8; A0A4V4SBS9 | 2 |
| S2-2 | Galactose metabolism | 6.47013e-10 | E6BD97; A0A828HF64; A0A5B9AHZ7; A0A4Y9XHJ6; A0A3Y1V1S8; A0A6D0H6A5; A0A7H9LVC8; A0A2H3MBD6; A0A789MBN3; A0A377CD10; A0A6L6RZ23; A0A5P0JCN2; A0A479JTS0; C0ST84; A0A6D0UCH6; A0A641J6U1; A0A4P0YBN2; A0A8A9FI50; A0A829IMX9; B7MEE9; A0A1X3LVF6; A0A2X1K1Q5; A0A1E5WU20; E8Z5D7; A0A826XBG0; A0A6L4XPN6; A0A376I262; D3GZF3; A0A827BBU1; A0A6N8P9J3; A0A376ME67; A0A844UUA4; A0A826WTR0; A0A8B5MA57; B7UFB0; A0A0H3EKR0; D6I9G0; W1X365; A0A768EGW4; A0A0P0SWP2; A0A1M2GPQ1 | 41 |
|  | Tryptophan metabolism | 6.34698e-07 | A8A6H0; A0A2X7H313; A0A5D8SLG5; A0A8B4PN44; A0A771BBG3; A0A377BSB0; A0A6G4BZV9; A0A7U9FZE8; A0A7U9ASB9; W9AMC1; K4XKM3; A0A271QSQ8; A0A8B5PFJ8; A0A853RYC6; A0A418U310; A0A827E0G2; A0A789MAE1; A0A777SAD9; A0A2A3VUL7 | 19 |
|  | RNA degradation | 0.003590236 | A0A6N8QE93; A0A777CE63; A0A831DIL6; D3QQ79; A0A5D8MVY1; A0A765T0J1; A0A7L7XED2; A0A6S4W2J6; A0A3L0WAU3; A0A3L0W137; A0A376LG23; A0A3L0VZ91; D7Y521; A0A377CPF6 | 14 |
|  | Pyrimidine metabolism | 0.007423756 | B7UHJ6; P76440; A0A827HLW1; A0A6N8Q332; A0A376D6E8; A0A4U9U1E4; A0A3L0VYM4; A0A6M0PVJ7; A0A6N8PRW7; A0A346GGF1; I4T542; B1LQY8; A0A1X9TR86; A0A2X1JNR5; A0A6D0EIL2; A0A418GJM4; A0A2H9EUJ5; A0A7A2V838; A0A376I3P5; A0A7D7PKU0; A0A827UE97; A0A5F1DTT1; A0A5F1E0X3; A0A0A1A5H6 | 24 |
|  | Glycerophospholipid metabolism | 0.010648927 | F4SG82; A0A7H9LUK6; A0A6M0PYN9; A0A6N6YDL1; A0A7I8Z834; A0A6D0USM6; A0A829DGU5; A0A2T1LIJ2; A0A2H4TXE8; A0A3L0VV29; A0A826SG38; A0A826XA79; A0A485JCI0; A0A2U2VLW9; A0A6N9MUA6 | 15 |
|  | Phenylalanine, tyrosine and tryptophan biosynthesis | 0.048967884 | E6BD92; A0A376HYQ1; A0A377D334; A0A7U9AWI8; Q6JZC5; Q6JZ44; A0A2I6IHS3; A0A6L4XR91; A0A6M0PTL1 | 9 |
|  | Phosphotransferase system (PTS) | 0.061823197 | A0A417ZXX0; A0A828HF64; A0A789MBN3; A0A479JTS0; C0ST84; B7MEE9; A0A1E5WU20; A0A828S4S8; A0A826XBG0; A0A377B7Z2; A0A2T1LHC8; B7UFB0; A0A0H3EKR0; A0A768EGW4; A0A6D0HB74 | 15 |
|  | MAPK signaling pathway - plant | 0.073489624 | A0A853WGK5; A0A2H9EUJ5 | 2 |
|  | Ferroptosis | 0.073489624 | P0A952; A0A826JV02 | 2 |
|  | Nitrotoluene degradation | 0.094254363 | P0ACD8; A0A827CGX5; A0A5F1DXH6; A0A7D7I018 | 4 |
|  | Glycerolipid metabolism | 0.106954811 | P76015; A0A0A0FCP4; A0A0K4PX90; A0A826XA79; A0A376ME67; A0A3P4Z1A7; A0A7H9QZ14 | 7 |
|  | Carbon fixation pathways in prokaryotes | 0.107599585 | P25516; P14407; F4T7V4; A0A6L4XMK4; A0A6M0PS89; A0A5D8SLG5; A0A826X499; A0A7Z1F0N1; C3SRM0; Q6JZJ4; A0A0L1C3K3; A0A383G4P5; A0A7Z8HVR4; A0A6N8PVJ3; A0A6M0PZE3; A0A7D7DVA6 | 16 |
|  | Citrate cycle (TCA cycle) | 0.10946002 | P25516; P14407; F4T7V4; A0A6L4XMK4; A0A2H9EVR4; A0A6M0PS89; A0A837MF64; A0A826X499; A0A7Z1F0N1; Q6JZJ4; A0A376Q109; A0A0L1C3K3; A0A7Z8HVR4; A0A3L0VV53; A0A7D7DVA6 | 15 |
|  | Secondary bile acid biosynthesis | 0.130994184 | A0A826ZD52; A0A827NRZ2 | 2 |
|  | Cell cycle - Caulobacter | 0.144543077 | F4TAV4; A0A3L0VT52; Q8XE60; A0A831FLZ4; A0A854RJR3; A0A2T1LPX8 | 6 |
|  | Sphingolipid metabolism | 0.166034555 | A0A376ME67 | 1 |
|  | Glycosphingolipid biosynthesis - globo and isoglobo series | 0.166034555 | A0A376ME67 | 1 |
|  | Pentose and glucuronate interconversions | 0.187374412 | A0A3Y1V1S8; A0A3L2NTY7; A0A7T6C0A4; A0A0E0XTU6; A0A417ZXJ2; A0A791VCX8; A0A844UUA4; A0A0H2VDQ8; A0A6C8RUQ2 | 9 |
|  | Inositol phosphate metabolism | 0.194958206 | A0A0K3QKD8; A0A4V4SBS9 | 2 |
|  | Phenylpropanoid biosynthesis | 0.194958206 | A0A271QSQ8; A0A789MAE1 | 2 |
| S2-3 | Galactose metabolism | 3.16e-08 | E6BD97; F4TBP9; A0A828HF64; A0A5B9AHZ7; A0A4Y9XHJ6; A0A6M0PS45; A0A7H9QPU0; A0A3Y1V1S8; A0A6D0H6A5; A0A7H9LVC8; A0A2H3MBD6; A0A377CD10; A0A6L6RZ23; A0A5P0JCN2; A0A479JTS0; A0A829L4W9; C0ST84; A0A6D0UCH6; A0A641J6U1; A0A4P0YBN2; Q9KIQ1; E8Z5G3; A0A8A9FI50; I2SXA9; A0A829IMX9; B7MEE9; A0A1X3LVF6; A0A2X1K1Q5; A0A1E5WU20; A0A826XBG0; A0A7B5NX36; A0A6L4XPN6; D3GZF3; A0A827BBU1; A0A831FCK9; A0A6N8P9J3; A0A844UUA4; A0A826WTR0; A0A8B5MA57; B7UFB0; A0A0H3EKR0; D6I9G0; W1X365; A0A768EGW4; A0A0P0SWP2; A0A1M2GPQ1 | 46 |
|  | Tryptophan metabolism | 8.69e-05 | A8A6H0; A0A2X7H313; A0A8B4PN44; A0A771BBG3; A0A6G4BZV9; A0A7U9FZE8; A0A7U9ASB9; W9AMC1; A0A659GVV1; A0A418GR70; K4XKM3; A0A271QSQ8; A0A8B5PFJ8; A0A853RYC6; A0A418U310; A0A827E0G2; A0A789MAE1; A0A777SAD9; A0A2A3VUL7 | 19 |
|  | Ribosome | 0.000332 | Q1R633; B1LR79; B7UK44; A7ZQ46; P0ADZ5; P02359; B7NGD6; A1AGC3; Q0TCE3; P0A7M2; A0A376WBS3; A0A5B9AS13; A0A7L5L2P8; A0A6M0PU76; A0A7A7AT69; A0A7A6MWE8; A0A7U2Z0M6; V0YFL6; A0A7B2TEE0; A0A2X7EYV8; A0A377ABT5; A0A6M0PR83; A0A2X3LQH0; A0A2X1JEL2; A0A376I523; A0A2X1PTA3; A0A787CMN5; A0A376JGA1; A0A789RQS2; A0A2T1LCI8; A0A6N8PVK8; A0A5B9ARY7; A0A377CSN6; A0A0T5XRJ0; A0A2T1LE54; A0A6D0H698; A0A2T1LEM2; A0A1D3KW65 | 38 |
|  | RNA degradation | 0.000512 | A0A6N8QE93; A0A777CE63; A0A6M0PZZ0; A0A831DIL6; A0A838AU16; A0A6N8Q014; A0A829L4W9; D3QQ79; A0A2T1LPS0; A0A5D8MVY1; A0A765T0J1; A0A6N8Q371; A0A7L7XED2; A0A6S4W2J6; A0A3L0W137; A0A376LG23; A0A3L0W5P1; D7Y521; A0A377CPF6 | 19 |
|  | Flagellar assembly | 0.000569 | P29744; P26608; P52627; P04949; A0A2A3WKJ5; A0A828S3C4; Q8XCG1; Q6VMV4; A1Z1Z5; A0A6D0FG95; A0A7U9B3A0; J7QSF1; A0A7U3BCZ4; A0A4C4K7Q3; A0A0K3STS8; A0A6D0G0H1; A0A377FAX0; A0A3Q0MWV8; A0A377K5F9; A0A5D1FPW1; A0A830TAZ3; A0A5D8S3Y2; A0A7T2N4B4; E9THW5; B3SGP3; A0A0A0H5V9; A0A6C9KSF1 | 27 |
|  | Phosphotransferase system (PTS) | 0.010611 | A0A417ZXX0; A0A828HF64; A0A6M0PTH7; A0A5D8MJ02; A0A3A6RSW6; A0A479JTS0; C0ST84; Q9KIQ1; A0A810UTR3; B7MEE9; A0A1E5WU20; A0A828S4S8; A0A826XBG0; A0A2T1LHC8; A0A6N4KV03; B7UFB0; A0A0H3EKR0; A0A0K9TBS3; A0A826XI71; A0A768EGW4; A0A826YR84; A0A6D0HB74 | 22 |
|  | Amino sugar and nucleotide sugar metabolism | 0.014026 | Q0TAX9; E6BD97; F4TBP9; A0A5F1DV27; A0A6D0EJA7; A0A6M0PS45; A0A7H9QPU0; A0A6M0PTH7; A0A3Y1V1S8; A0A6D0GV09; A0A810UTR3; E8Z5G3; I2SXA9; A0A1X3LVF6; A0A6L4XPN6; A0A2H4TNY6; A0A6N4KV03; A0A376MTF2; A0A6L7A3K9; A0A6D0IS19; A0A376S3L5; A0A2X1MYJ8; A0A6L4XLZ2; A0A844UUA4; A0A6M7H4E4; A0A0K9TBS3; A0A1M2GPQ1 | 27 |
|  | Glycerophospholipid metabolism | 0.019408 | F4SG82; A0A7H9LUK6; A0A6M0PYN9; A0A6N6YDL1; A0A7I8Z834; A0A6D0USM6; A0A829DGU5; A0A2T1LIJ2; A0A2H4TXE8; A0A3L0VV29; A0A377A8R1; A0A826SG38; A0A485JCI0; A0A862ZHA6; A0A140N469; A0A6C8TFT0; A0A2U2VLW9; A0A6N9MUA6 | 18 |
|  | Bacterial chemotaxis | 0.026974 | C6UY58; P0AE68; P02942; A0A828NV63; A0A4T5JV04; A0A3L0VXR4; A0A3L0VY65; A0A5D8STB8; A0A8A5HAP0; A0A7U9B3A0; B2MWK7; A0A829DCS4; A0A024L1I7; A0A376I0Z6; A0A6M0PXN7; A0A6N6Y316; A0A376RJ77; A0A376I0W6; A0A777GNP1; A0A5D1FPW1; A0A5F1DKT1; A0A2T1LJI9; A0A4Y8FUT9; A0A383FA69 | 24 |
|  | Biofilm formation - Escherichia coli | 0.032126 | P52627; A0A1X3IZZ0; A0A1M2ERV6; A0A827G2C2; A0A826QVU1; A0A827N447; A0A6D0FG95; A0A6N0IKN9; A0A0B5HCB2; A0A6H3L0C8; A0A2J1DEI0; A0A8B5INY3; A0A0K5BCV2; A0A6L7E3U8; A0A029HTA9; A0A345ESS5; A0A6M0PZM1; A0A7T2N4B4; A0A0K9TJ89 | 19 |
|  | Two-component system | 0.05775 | P69229; C4ZW75; P0AE68; P0AE89; P75726; P02942; P04949; P11349; F4T7V4; A0A1X3IZZ0; D8A5G6; A0A2T1LLY9; A0A827VF59; A0A7D7DG69; A0A826QVU1; A0A2U9KS47; A0A376I8B9; A0A376MVG2; A0A826VXD8; A0A3W5XXF0; A0A5D8STB8; A0A6S4W0I6; A0A828S3C4; L7UYD3; Q6VMV4; A1Z1Z5; A0A6D0FG95; A0A0B5HCB2; A0A7U9QDI6; A0A0E2L4V5; W9ADK8; B2MWK7; A0A1X3LUN7; A0A829DWP8; A0A6D0ILH3; A0A4C4K7Q3; A0A024L1I7; A0A241QPT0; A0A7D7HDD9; A0A2K4PB62; A0A6L4XQM6; A0A376I0Z6; A0A0K4IU38; A0A6N6Y316; A0A376RJ77; A0A1Y2XU03; A0A447Y522; A0A828L8H3; A0A029HTA9; D3GU02; A0A345ESS5; A0A777GNP1; A0A7I7HZN9; A0A7I9AJI9; A0A5D1FPW1; A0A6M0PZM1; A0A6L4XLZ2; A0A7T2N4B4; A0A5F1DKT1; A0A2T1LJI9; A0A1X3KIE5; A0A7D7DVZ1; B3SGP3; A0A0K4UCX5; A0A4Y8FUT9; A0A826Q325; A0A827EG33 | 67 |
|  | Pyrimidine metabolism | 0.087336 | B7UHJ6; P07650; B1LNX6; P23331; A0A827HLW1; A0A6N8Q332; A0A773MCR6; A0A4U9U1E4; A0A3L0VYM4; A0A6M0PVJ7; A0A6N8PRW7; A0A346GGF1; I4T542; A0A829DRF6; B1LQY8; A0A7A9N155; A0A1X9TR86; A0A6D0EIL2; A0A2H9EUJ5; A0A7A2V838; A0A376I3P5; A0A7D7PKU0; A0A827UE97; A0A5F1DTT1; A0A5F1E0X3; A0A0A1A5H6 | 26 |
|  | Drug metabolism - other enzymes | 0.093513 | P07650; P23331; A0A773MCR6; A0A5N8HMR5; I4T542; A0A7A9N155; A0A271QSQ8; A0A2H9EUJ5; A0A827UE97; A0A789MAE1; A0A5F1E0X3 | 11 |
|  | Biofilm formation - Vibrio cholerae | 0.096167 | A0A826QVU1; A0A831DIL6; A0A828S3C4; A0A0B5HCB2; A0A6N4KV03; A0A6M0PZR8; A0A6M0PZM1; A0A6L4XLZ2; A0A7T2N4B4 | 9 |
|  | Biofilm formation - Pseudomonas aeruginosa | 0.116821 | A0A826QVU1; A0A6D0FG95; A0A0B5HCB2; A0A6M0PZM1; A0A7T2N4B4 | 5 |
|  | MAPK signaling pathway - plant | 0.133058 | A0A853WGK5; A0A2H9EUJ5 | 2 |
|  | Ferroptosis | 0.133058 | P0A952; A0A826JV02 | 2 |
|  | Streptomycin biosynthesis | 0.134072 | P37760; Q1RA34; F4TBP9; A0A6M0PS45; A0A3U1V7R9; A0A6N6XAM7; E8Z5G3; I2SXA9 | 8 |
|  | Cyanoamino acid metabolism | 0.137478 | E6BGX8; A0A5B9AYS1; A0A862ZK76; A0A6N8QP55; A0A376I3W0; A0A2S8JZY6 | 6 |
|  | Biosynthesis of nucleotide sugars | 0.160925 | Q0TAX9; P37760; E6BD97; Q1RA34; F4TBP9; A0A5F1DV27; A0A6D0EJA7; A0A6M0PS45; A0A7H9QPU0; A0A2X7IEI8; A0A3Y1V1S8; A0A6N6XAM7; A0A6D0GV09; A0A6D0FKH7; E8Z5G3; I2SXA9; A0A1X3LVF6; A0A6L4XPN6; D3QWK1; A0A6D0IS19; A0A2X1MYJ8; A0A6L4XLZ2; A0A844UUA4; A0A789R943; A0A1M2GPQ1 | 25 |
