# Supplementary material for: In vivo fitness of sul gene-dependent sulfonamide-resistant Escherichia coli in the mammalian gut
Source: mSystems. 2024 Aug 14;9(9):e00836-24. doi: 10.1128/msystems.00836-24 (PMC11406977; doi:10.1128/msystems.00836-24)
Supplement: Tables S7 and S8 — Primers, strains, and plasmids. [file msystems.00836-24-s0006.docx]

**Table S7** Primer sequences

| No. | Primer names | Sequence (5’-3’) | References |
| --- | --- | --- | --- |
| 1 | Sul1-KpnI-F | GGGGTACCATGGTGACGGTGTTCGGCATTCTG | This work |
| 2 | Sul1-XhoI-R | CCGCTCGAGCTAGGCATGATCTAACCCTCGGTC | This work |
| 3 | Sul2-KpnI-F | GGGGTACCATGAATAAATCGCTCATCATTTTC | This work |
| 4 | Sul2-XhoI-R | CCGCTCGAGTTAACGAATTCTTGCGGTTTCTTT | This work |
| 5 | Sul3-KpnI-F | GGGGTACCATGAGCAAGATTTTTGGAATCGTA | This work |
| 6 | Sul3-XhoI-R | CCGCTCGAGCTAACCTAGGGCTTTGGATATTTT | This work |
| 7 | araB-F | TTATAGAGTCGCAACGGCCTGG | This work |
| 8 | araB-R | CCGTGGCGCAATCTGCCGCATC | This work |
| 9 | araB-Cm-F | GTTGGCCGATTCATTAATGCCCGCTCGCCAACGCTCTGTTTGTCG | This work |
| 10 | araB-Cm-R | CTTACGTGCCGATCAAACGCTAACCAACGCCTGAAAGGGGTGATT | This work |
| 11 | cm-araB-F | CAGGCGTTGGTTAGCGTTTGATCGGCACGTAAGAGGTTCCAA | This work |
| 12 | cm-araB-R | CGTTGGCGAGCGGGCATTAATGAATCGGCCAACGCGCGGGGAG | This work |
| 13 | araB-JD-F | CGCCCAGTGCCATCACGTT | This work |
| 14 | araB-JD-R | CACCTGCGACATTCTGATTGCC | This work |
| 15 | sgRNA | ACCGGCAGGTGATCCAGAGA | This work |
| 16 | lacI-F | TCGCAGAGTATGCCGGTGTCTC | This work |
| 17 | lacI-R | CCTGGTCATCCAGCGGATAGTTAA | This work |
| 18 | ybaM-F | GTCACTGGAAAATGCCCCTGACGAT | This work |
| 19 | ybaM-R | ACTTTTCCGCCTCATCATCGCGCGTT | This work |
| 20 | folD-F | GCGCAGCAGGTGCGCTCTGAAGT | This work |
| 21 | folD-R | GGTCATAAGAGCGGGAGACGAAC | This work |
| 22 | ybfP-F | GCCGTGGTGATAACTTACCTGAT | This work |
| 23 | ybfP-R | CTGGCAGCGTCGTAACTCAATAA | This work |
| 24 | ptrB-F | CCGCAGTGAACACCTGTGGATAG | This work |
| 25 | ptrB-R | TGGGCGATAACGCCGTGGAATAA | This work |
| 26 | insH6-F | GGCATGAAGGCCCACATTGGTGT | This work |
| 27 | insH6-R | TCATTTGGTCCGCCCGAAACAGG | This work |
| 28 | YqeF-F | ATGATGATGAGCGAAGCCAAAGC | This work |
| 29 | YqeF-R | CACGTTCAATGGTCAATGCCACA | This work |
| 30 | SpoT-F | AAACCTACCTGCCGGAAGACCAA | This work |
| 31 | SpoT-R | CGCCTCTTTCTTATCGCGGAACT | This work |
| 32 | GlpK-F | TTATGGCAGGCGCATCCATTCAG | This work |
| 33 | GlpK-R | CTCTTGCAGCTCGTCGAGGTTCT | This work |
| 34 | Glk-F | GAAAATCTCAAGCCAAAAGATATTAC | This work |
| 35 | Glk-R | GTATGATTTAAAAGATTATCGGGAGA | This work |

No. 1–6: primers for Sanger sequencing and cloning the *sul1*, *sul2* and *sul3* gene from the isolates. No. 7–10: primers for cloning the homologous sequence of *araB*; No. 11–12: primers for cloning the full-length chloramphenicol resistance gene (including promoter and terminator); No. 13–14: primers for identifying the recombinant strain *E. coli* MG1656 *araB::cat* edited by CRISPR-Cas9 system; No. 15: sgRNA (single guide RNA) for the guidance of CRISPR-Cas9 editing. No. 16–35: primers for the amplification of the fragments which containing the SNP site.

**Table S8** Strains and plasmids

| Plasmids and strains | Description |
| --- | --- |
| Plasmids: |  |
| pZA2^a^ | Contains *aph(3')-Ia* gene (conferring resistance to kanamycin), p15a replicon and multiple cloning site |
| pZA2-*sul1* | *sul1* gene in sulfonamide resistant isolates cloned into pZA2 |
| pZA2-*sul2* | *sul2* gene in sulfonamide resistant isolates cloned into pZA2 |
| pZA2-*sul3* | *sul3* gene in sulfonamide resistant isolates cloned into pZA2 |
| Strains: |  |
| *Escherichia coli* MG1656 ^a^ | MG1655 (K-12F^-^ *λ^-^ ilvG^-^ rfb-50 rph-1*) *ΔlacMluI* |
| *Escherichia coli* MG1656 *araB::cat* ^b^ | MG1656 strain containing the chloramphenicol resistance marker, *cat* gene, inserted into the chromosomal *araB* gene |
| LC: MG1656 *araB::cat* + pZA2 | MG1656 *araB::cat* carrying pZA2 plasmid (used as the control strain) |
| P1: MG1656 *araB::cat* + pZA2-*sul1* | MG1656 *araB::cat* carrying pZA2-*sul1* plasmid |
| P2: MG1656 *araB::cat* + pZA2-*sul2* | MG1656 *araB::cat* carrying pZA2-*sul2* plasmid |
| P3: MG1656 *araB::cat* + pZA2-*sul3* | MG1656 *araB::cat* carrying pZA2-*sul3* plasmid |
| S2-1 | Compensatory mutant *sul2* gene-dependent strain |
| S2-2 | Compensatory mutant *sul2* gene-dependent strain |
| S2-3 | Compensatory mutant *sul2* gene-dependent strain |

Note: ^a^ For a description of the construction of plasmid pZA2 and *Escherichia coli* MG1656, refer to Lutz and Bujard (1997) and Lacotte (2017). ^b^ *araB::cat*: the chloramphenicol resistance marker, *cat* gene, inserted into the chromosomal *araB* gene of the MG1656 strains using the CRISPR-Cas9 system (Jiang et al., 2015).
