# Supplementary material for: In vivo fitness of sul gene-dependent sulfonamide-resistant Escherichia coli in the mammalian gut
Source: mSystems. 2024 Aug 14;9(9):e00836-24. doi: 10.1128/msystems.00836-24 (PMC11406977; doi:10.1128/msystems.00836-24)
Supplement: Legends — Supplemental figure and table legends. [file msystems.00836-24-s0007.docx]

**Supplemental legends**

**Fig S1** Characterization of three sulfonamide resistant test strains P1, P2, and P3 and sulfonamide-sensitive control strain LC. **(a)** Polymerase Chain Reaction products of strains P1, P2, and P3 analyzed by agarose gel electrophoresis. M: Takara DL2,000 DNA Marker; 1: *sul1* gene positive control; 2: *sul1* gene of strain P1; 3: *sul2* gene positive control; 4: *sul2* gene of strain P2; 5: *sul3* gene positive control; 6: *sul3* gene of strain P3; N1: negative control of strain P1; N2: negative control of strain P2; N3: negative control of strain P3. **(b)** Antimicrobial susceptibility tests of strains P1, P2, P3 and LC. (b1): strain P1; (b2): strain P2; (b3): strain P3; (b4): strain LC; susceptibility paper 1: kanamycin; 2: ampicillin; 3: chloramphenicol; 4: sulfisoxazole. **(c)** Minimum inhibitory concentration (MIC) of sulfisoxazole in strains P1, P2, P3, and LC.

**Fig S2** Characterization of the three compensatory mutant strains (CMSs) S2-1, S2-2, S2-3, and the parent strain P2. **(a)** Antimicrobial susceptibility tests of the three CMSs S2-1, S2-2, S2-3, and parent strain P2. (a1): parent strain P2; (a2): CMS S2-1; (a3): CMS S2-2; (a4): CMS S2-3; susceptibility paper 1: kanamycin; 2: sulfisoxazole; 3: chloramphenicol; 4: ampicillin. **(b)** MIC of sulfisoxazole in the three CMSs and the parent strain P2. **(c)** PCR products of three CMSs after agarose gel electrophoresis analysis. M: Takara DL2,000 DNA Marker; 1: *uidA* gene of *Escherichia coli* positive control; 2-4: *uidA* gene of CMSs S2-1, S2-2, and S2-3, respectively; 5: *sul2* gene positive control; 6-8 *sul2* gene of CMSs S2-1, S2-2, and S2-3 respectively; N1: negative control of *E. coli*; N2: negative control of CMSs. **(d)** Antimicrobial resistance genes in the CMSs analyzed by whole-genome sequencing analysis. **(e)** *In vitro* growth curves of sulfonamide-sensitive control strain LC, parent strain P2, and CMSs S2-1, S2-2, and S2-3.

**Fig S3** Top 20 terms from the Kyoto Encyclopedia of Genes and Genomes pathway enrichment analysis of differentially expressed proteins (DEPs). **(a)** Compensatory mutant strain S2-1 *vs.* parent strain P2. **(b)** S2-2 *vs.* P2. **(c)** S2-3 *vs.* P2. The *x*-axis represents the enrichment factor (Rich Factor ≤ 1), which denotes the ratio of the number of annotated DEPs to the number of proteins annotated to these functional categories. The *y*-axis indicates the number of DEPs under each functional category.

**Table S1** The data of the CFU per gram feces of each strain in each mouse from which the competitive index is calculated

**Table S2** Information on differentially expressed proteins of the compensatory mutant strains S2-1, S2-2, and S2-3.

**Table S3** Differentially expressed protein information related to biological process ontology of the compensatory mutant strains S2-1, S2-2, and S2-3.

**Table S4** Differentially expressed protein information related to cellular component ontology of the compensatory mutant strains S2-1, S2-2, and S2-3.

**Table S5** Differentially expressed protein information related to molecular function ontology of the compensatory mutant strains S2-1, S2-2, and S2-3.

**Table S6** Pathway enrichment analysis of differentially expressed proteins in the compensatory mutant strains S2-1, S2-2, and S2-3.

**Table S7** Primer sequences.

**Table S8** Strains and plasmids.
