# Supplementary figures and images for: In vivo fitness of sul gene-dependent sulfonamide-resistant Escherichia coli in the mammalian gut
Source: mSystems. 2024 Aug 14;9(9):e00836-24. doi: 10.1128/msystems.00836-24 (PMC11406977; doi:10.1128/msystems.00836-24)

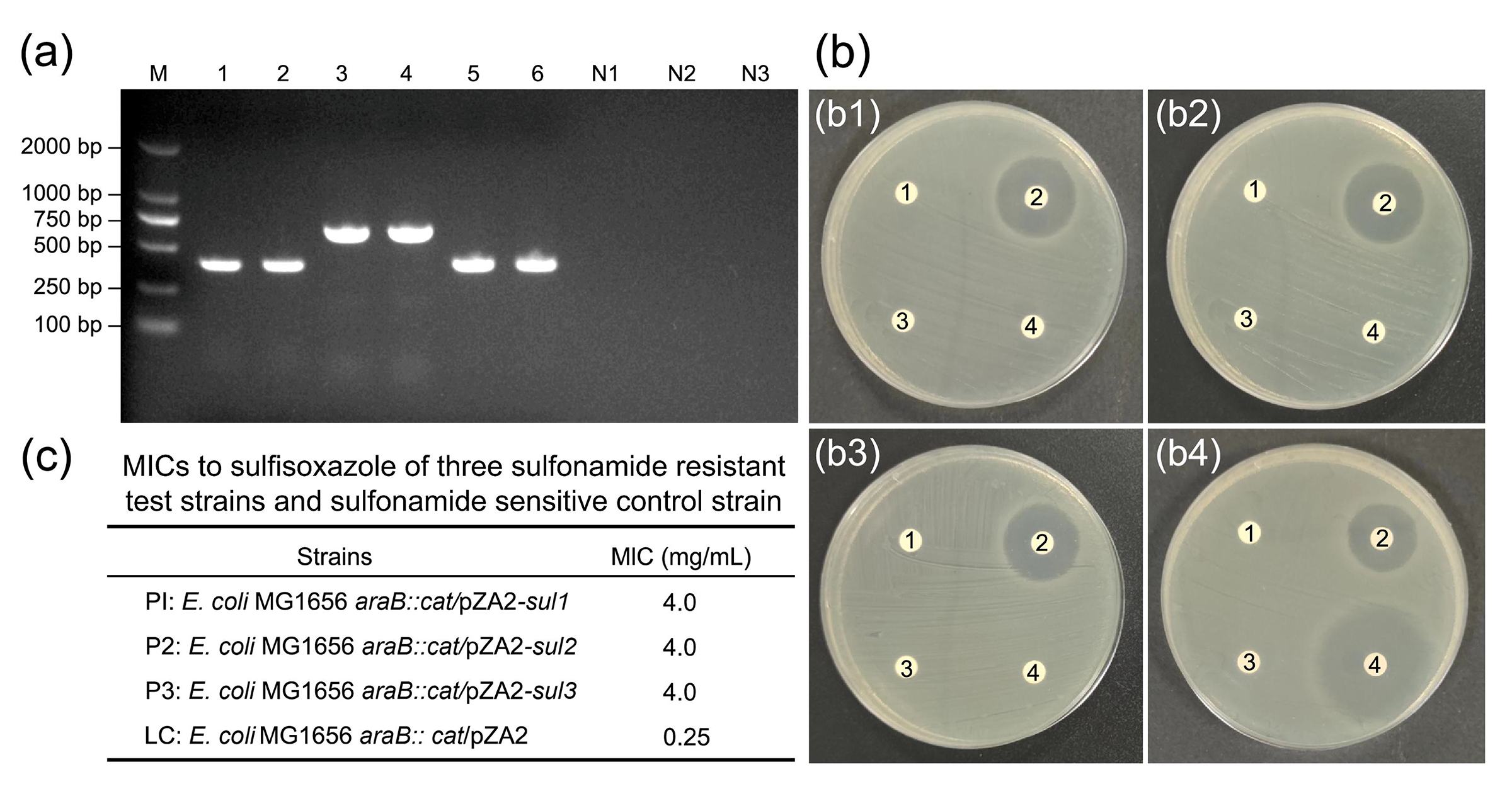

Supplement: Figure S1 — Characterization of sulfonamide-resistant test strains P1, P2, and P3 and sulfonamide-sensitive control strain LC. [file msystems.00836-24-s0008.tif]

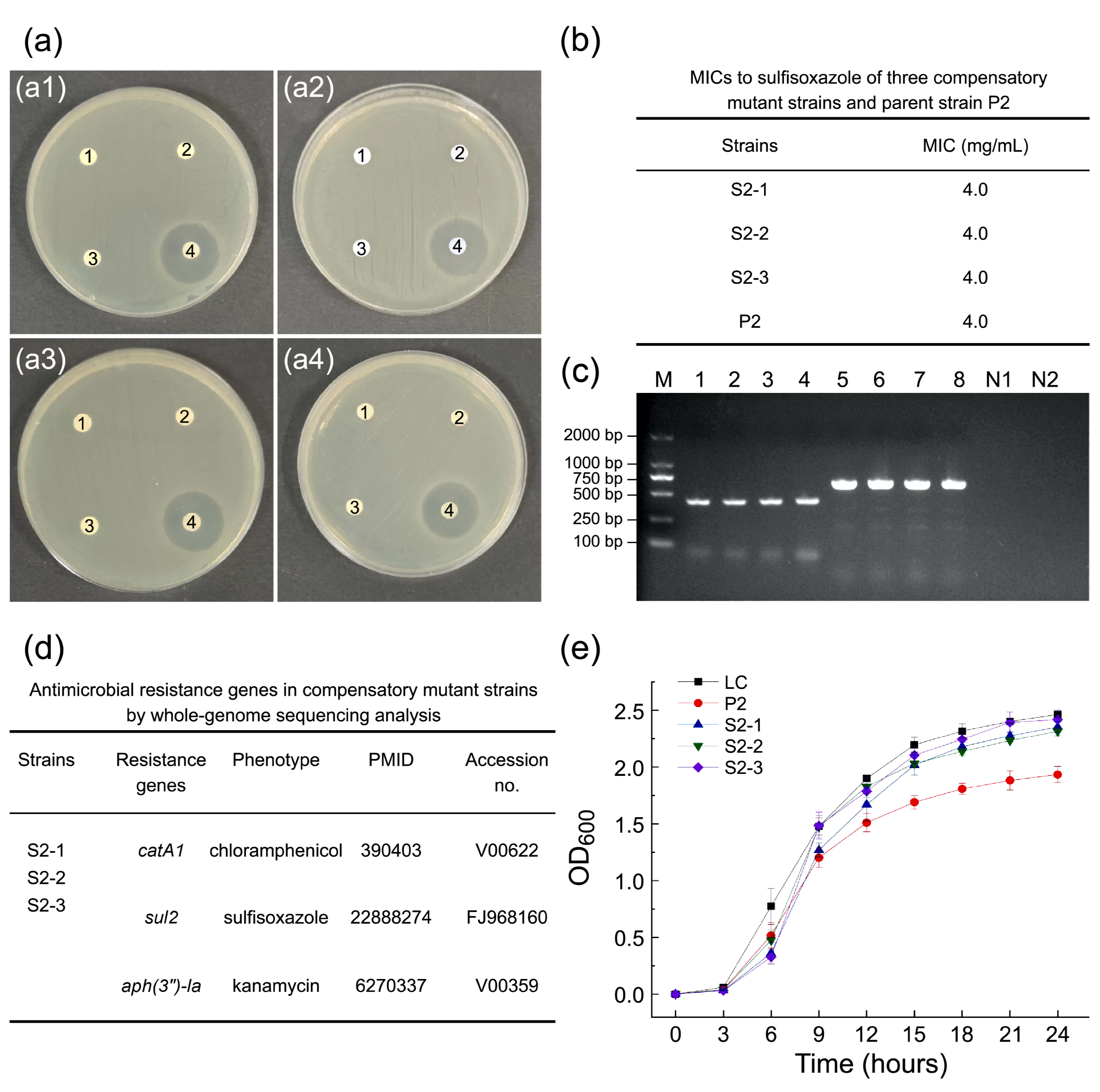

Supplement: Figure S2 — Characterization of CMS S2-1, S2-2, and S2-3 and parent strain P2. [file msystems.00836-24-s0009.tif]

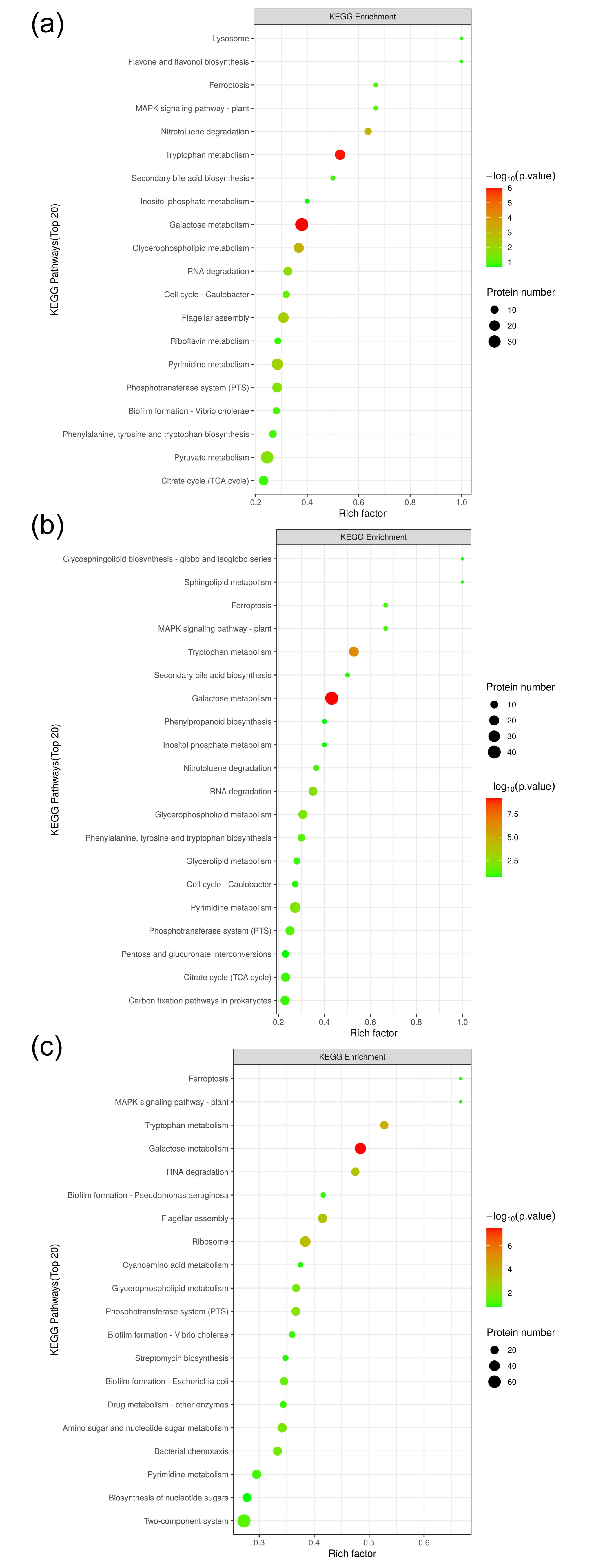

Supplement: Figure S3 — Top 20 terms from the Kyoto Encyclopedia of Genes and Genomes pathway enrichment analysis of differentially expressed proteins (DEPs). [file msystems.00836-24-s0010.tif]
